# Role of the Gut-Brain Axis in the Shared Genetic Etiology Between Gastrointestinal Tract Diseases and Psychiatric Disorders: A Genome-Wide Pleiotropic Analysis

**DOI:** 10.1001/jamapsychiatry.2022.4974

**Published:** 2023-02-08

**Authors:** Weiming Gong, Ping Guo, Yuanming Li, Lu Liu, Ran Yan, Shuai Liu, Shukang Wang, Fuzhong Xue, Xiang Zhou, Zhongshang Yuan

**Affiliations:** 1 Department of Biostatistics, School of Public Health, Cheeloo College of Medicine, Shandong University, Jinan, Shandong, China; 2 Institute for Medical Dataology, Shandong University, Jinan, China; 3 School of Medicine, Cheeloo College of Medicine, Shandong University Jinan, China; 4 Department of Biostatistics, University of Michigan, Ann Arbor; 5 Center for Statistical Genetics, University of Michigan, Ann Arbor

## Abstract

**Importance:**

Comorbidities and genetic correlations between gastrointestinal tract diseases and psychiatric disorders have been widely reported, with the gut-brain axis (GBA) hypothesized as a potential biological basis. However, the degree to which the shared genetic determinants are involved in these associations underlying the GBA is unclear.

**Objective:**

To investigate the shared genetic etiology between gastrointestinal tract diseases and psychiatric disorders and to identify shared genomic loci, genes, and pathways.

**Design, Setting, and Participants:**

This genome-wide pleiotropic association study using genome-wide association summary statistics from publicly available data sources was performed with various statistical genetic approaches to sequentially investigate the pleiotropic associations from genome-wide single-nucleotide variation (SNV; formerly single-nucleotide polymorphism [SNP]), and gene levels and biological pathways to disentangle the underlying shared genetic etiology between 4 gastrointestinal tract diseases (inflammatory bowel disease, irritable bowel syndrome, peptic ulcer disease, and gastroesophageal reflux disease) and 6 psychiatric disorders (schizophrenia, bipolar disorder, major depressive disorder, attention-deficit/hyperactivity disorder, posttraumatic stress disorder, and anorexia nervosa). Data were collected from March 10, 2021, to August 25, 2021, and analysis was performed from January 8 through May 30, 2022.

**Main Outcomes and Measures:**

The primary outcomes consisted of a list of genetic loci, genes, and pathways shared between gastrointestinal tract diseases and psychiatric disorders.

**Results:**

Extensive genetic correlations and genetic overlaps were found among 22 of 24 trait pairs. Pleiotropic analysis under a composite null hypothesis identified 2910 significant potential pleiotropic SNVs in 19 trait pairs, with 83 pleiotropic loci and 24 colocalized loci detected. Gene-based analysis found 158 unique candidate pleiotropic genes, which were highly enriched in certain GBA-related phenotypes and tissues, whereas pathway enrichment analysis further highlighted biological pathways primarily involving cell adhesion, synaptic structure and function, and immune cell differentiation. Several identified pleiotropic loci also shared causal variants with gut microbiomes. Mendelian randomization analysis further illustrated vertical pleiotropy across 8 pairwise traits. Notably, many pleiotropic loci were identified for multiple pairwise traits, such as 1q32.1 (*INAVA*), 19q13.33 (*FUT2*), 11q23.2 (*NCAM1*), and 1p32.3 (*LRP8*).

**Conclusions and Relevance:**

These findings suggest that the pleiotropic genetic determinants between gastrointestinal tract diseases and psychiatric disorders are extensively distributed across the genome. These findings not only support the shared genetic basis underlying the GBA but also have important implications for intervention and treatment targets of these diseases simultaneously.

## Key Points

**Question:**

To what extent are shared genetic determinants in the comorbidities and associations between gastrointestinal tract diseases and psychiatry disorders involved in the gut-brain axis?

**Findings:**

In this genome-wide pleiotropic association study using genome-wide association summary statistics from publicly available data sources, pervasive genetic correlations and genetic overlaps between gastrointestinal tract diseases and psychiatric disorders were found. The pleiotropic genetic determinants between them were extensively distributed across the genome.

**Meaning:**

These findings not only support the shared genetic basis underlying the gut-brain axis but also have important implications for intervention and treatment targets of these 2 types of diseases simultaneously.

## Teaser

This genome-wide pleiotropic association study sequentially investigates the pleiotropic associations from genetic and biological pathways to disentangle the underlying shared genetic etiology between 4 gastrointestinal tract diseases and 6 psychiatric disorders.

## Introduction

The comorbidities and associations between gastrointestinal tract diseases and psychiatric disorders have been widely reported,^1,2,3^ which was likely to be regulated by the gut-brain axis (GBA). The GBA is characterized by bidirectional interactions between the gastrointestinal tract and the central nervous system (CNS) and would link intestinal dysfunction and inflammation with brain function and psychiatric disorders.^4,5^ Various biological mechanisms were involved in the GBA, such as inflammatory immune responses, the autonomic nervous system, and enteric nervous system, where the role of the composition of gut microbiota and related metabolites has been particularly highlighted.^4,5,6,7^ Underlying the conceptual framework of the GBA, the shared genetic etiology might be involved in the associations between gastrointestinal tract diseases and psychiatric disorders.

Genome-wide association studies (GWAS) have identified multiple genetic variants (ie, single-nucleotide variations [SNVs]; formerly single-nucleotide polymorphisms [SNPs]) associated with gastrointestinal tract diseases and psychiatric disorders.^8,9,10^ Genetic correlations have been suggested between these 2 types of diseases using linkage disequilibrium (LD) score regression (LDSC).^10,11,12,13,14^ However, it remains unclear whether the overall genetic correlation would be attributed to a few loci or across the genome.^15^ Indeed, there would be genetic overlap even without any genetic correlation. Although previous studies have investigated genetic overlap,^16^ shared susceptibility genes,^17,18^ and causal relationships^19,20,21^ between these 2 types of diseases, they mainly focused on inflammatory bowel disease (IBD) and psychiatric disorders with limited sample sizes. Recently, 2 studies^10,22^ have conducted GWAS of specific gastrointestinal tract diseases as well as systematic post-GWAS analyses and pointed out the necessity to explore the shared genetic risk across traits to improve the understanding of the disordered brain-gut interactions. Therefore, it is of great importance to further seek out the specific genomic variants or loci accounting for genome-wide genetic correlation and to deeply probe into the shared genetic etiology between these 2 types of diseases. Shared genetic etiology also indicates the potential pleiotropy, which often acts as genetic confounding of the associations between trait pairs.^23,24,25^ Cross-trait analysis has been proposed to investigate the pleiotropic genetic variants or loci among multiple traits by leveraging the correlation of GWAS signals,^23,26,27,28,29^ where the pleiotropic loci could serve as intervention targets with the potential to simultaneously prevent or treat these diseases.

In this genome-wide pleiotropic association study, using large-scale GWAS summary data, we performed a genome-wide pairwise trait pleiotropic analysis between 4 gastrointestinal tract diseases (IBD, irritable bowel syndrome [IBS], peptic ulcer disease [PUD], and gastroesophageal reflux disease [GERD]) and 6 psychiatric disorders (schizophrenia, bipolar disorder [BIP], major depressive disorder [MDD], attention-deficit/hyperactivity disorder [ADHD], posttraumatic stress disorder [PTSD], and anorexia nervosa [AN]) through various statistical genetic approaches to sequentially investigate the pleiotropic associations from genome-wide, SNV, and gene levels and biological pathways to disentangle the underlying shared genetic etiology. Of note, under the framework of pleiotropic analysis, we first performed the SNV-level analysis to detect pleiotropic variants and loci, followed by pairwise colocalization analysis to determine colocalized loci and gene-level analysis to identify candidate pleiotropic genes, based on which we further performed parallel phenotype and tissue-specific enrichment analysis to characterize the phenotype and tissue specificity as well as additional gene-level analysis to identify the tissue-specific and cell type-specific pleiotropic genes. We also highlighted the role of gut microbiomes in interpreting the shared genetic etiology, followed by mendelian randomization analysis to evaluate pairwise causal associations and partly characterize different types of pleiotropy (vertical pleiotropy or horizontal pleiotropy).

## Methods

### GWAS Data Sets

We sought GWAS summary statistics from publicly available data sources with European ancestry, owing to the limited availability of well-powered GWAS with other ancestries, and selected GWAS with sample sizes larger than 50 000 to ensure statistical power. GWAS for GERD, IBD, and PUD were obtained from the same gastrointestinal tract GWAS based on 456 327 individuals from UK Biobank (UKB).^10^ GWAS for IBS were obtained from a larger meta-analysis with 486 601 individuals (53 400 cases and 433 201 controls).^22^ GWAS for the 6 psychiatric disorders were from the Psychiatric Genomics Consortium, including schizophrenia,^30^ BIP,^31^ MDD,^32^ ADHD,^33^ PTSD,^34^ and AN.^35^ In addition, GWAS for early age-related macular degeneration (AMD)^36^ and cataract^37^ were obtained to serve as a common set of negative controls for both gastrointestinal tract diseases and psychiatric disorders, given these 2 disorders are relatively limited to the pathological lesions of intraocular contents, and previous studies also showed no significant genetic correlations between AMD and MDD^38^ as well as among cataract, GERD, and psychiatric symptoms.^39^ All GWAS were approved by relevant ethic committees, and written informed consent was obtained from all participants, with details provided in the eTable 1 in Supplement 1. Data were collected from March 10, 2021, to August 25, 2021, and analyzed from January 8 through May 30, 2022. This genome-wide pleiotropic association study followed the Strengthening the Reporting of Genetic Association Studies (STREGA) reporting guideline.

### Statistical Analysis

**Figure 1.**
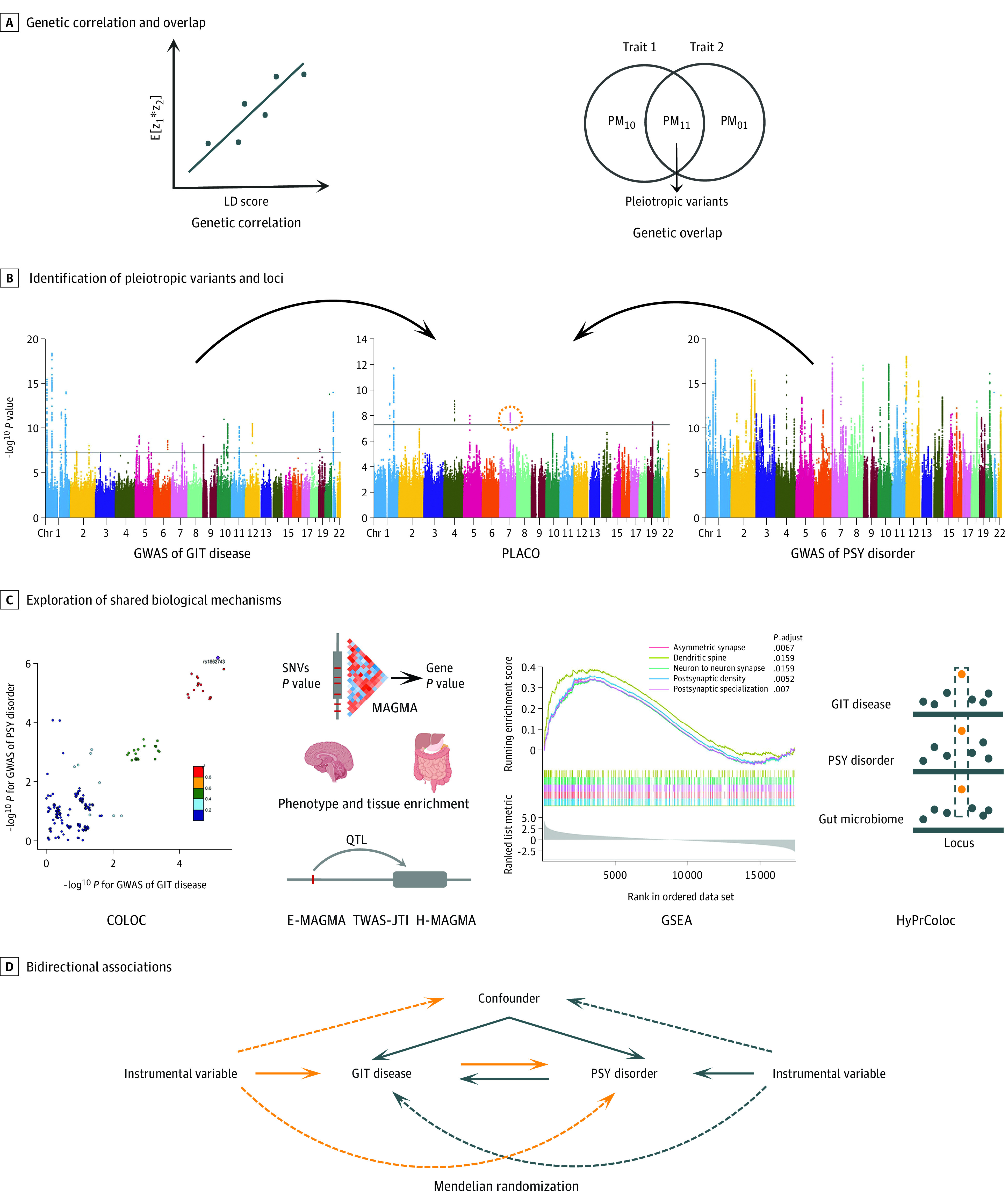
Study Workflow We conducted the comprehensive pleiotropic analysis between 4 gastrointestinal tract (GIT) diseases and 6 psychiatric (PSY) disorders from different perspectives. COLOC indicates colocalization; GSEA, gene set enrichment analysis; GWAS genome-wide association study; E-MAGMA, expression quantitative trait loci–informed multi-marker analysis of GenoMic annotation (MAGMA); H-MAGMA, Hi-C–coupled MAGMA; HyPrColoc, hypothesis prioritization for multi-trait colocalization; LD, linkage disequilibrium; PLACO, pleiotropic analysis under composite null hypothesis; SNV, single-nucleotide variation; and TWAS-JTI, transcriptome-wide association study analysis using joint-tissue imputation.

All analyses were performed after excluding SNVs in the major histocompatibility complex region (chromosome 6: 25-35 megabase [Mb]) due to its complex LD structure and restricted to biallelic SNVs with minor allele frequency larger than 0.01. Details of these methods are provided in Figure 1 and eMethods in Supplement 1.

Both LDSC^40^ and high-definition likelihood^41^ were used to assess genome-wide genetic correlations for 24 pairwise traits. The intercept from LDSC could also indicate potential sample overlap between 2 GWAS. Given that genetic correlation only reflects the overall correlation across the genome, we further applied genetic analysis incorporating pleiotropy and annotation (GPA)^29^ to explore the overall genetic overlap between traits. The Bonferroni-corrected significant threshold was set at *P* < 2.08 × 10^−3^ (.05/24). In addition, negative control analysis was performed through LDSC between AMD and cataract and the 4 gastrointestinal tract diseases as well as the 6 psychiatric disorders, with Bonferroni-corrected significant threshold set at *P* < 2.5 × 10^−3^ (.05/20).

For the union set of pairwise traits with significant genetic correlation or genetic overlap, we used pleiotropic analysis under composite null hypothesis (PLACO) to identify potential pleiotropic SNVs.^26^ Single-nucleotide variations with *P* < 5 × 10^−8^ for PLACO were considered significant pleiotropic variants. Functional mapping and annotation of genetic associations (FUMA)^42^ was applied to characterize potential pleiotropic loci, based on which a bayesian colocalization analysis^43^ was performed to further identify shared causal variants in each pleiotropic locus. We declared a colocalized locus with posterior probability of H4 (PP.H4) larger than 0.7.

Based on PLACO results, we further explored the shared biological mechanisms of these pleiotropic loci. We performed gene-level multimarker analysis of GenoMic annotation (MAGMA)^44^ analysis on the genes located in or overlapped with the pleiotropic loci based on both PLACO outputs and single-trait GWAS to identify candidate pleiotropic genes, with the significance declared at the locus-specific Bonferroni-corrected 2-sided *P* < .05 for MAGMA analysis on PLACO results and 2-sided *P* < .05 for MAGMA analyses based on original single-trait GWAS. Further, we performed phenotype enrichment analysis based on the Mouse Genome Informatics platform^45^ to characterize the phenotype specificity of these pleiotropic genes against that of nonpleotropic genes by examining the differences in the proportions of genes associated with certain phenotypes in the pleiotropic gene group against that in the nonpleotropic gene group using the Fisher exact test. Then, we performed tissue-specific enrichment analyses to illustrate the tissue specificity of these pleiotropic genes using the deTS tissue-specific enrichment method^46^ based on 2 different reference panels, Genotype-Tissue Expression project (GTEx)^47^ and the Encyclopedia of DNA Elements project (ENCODE).^48^ We declared the significance with a nominal threshold (2-sided *P* < .05) for these 2 parallel enrichment analyses. In addition, we used E-MAGMA (expression quantitative trait loci [eQTL]–informed MAGMA)^49^ and transcriptome-wide association study analysis using joint-tissue imputation^50^ to further investigate the tissue-specific genes, parallelized with H-MAGMA (Hi-C–coupled MAGMA)^51^ to indicate the cell-type specificity, in which we declared the significance with locus-specific Bonferroni correction. Gene set enrichment analysis was also performed to identify potential biological pathways using the clusterProfiler package.^52^ Significantly enriched pathways were declared with normalized enrichment score greater than 2 and adjusted *P* < .05. Multitrait colocalization analysis with HyPrColoc (hypothesis prioritization for multi-trait colocalization)^53^ incorporating host-microbiome GWAS was performed to highlight the critical role of gut microbiome.

Last, we conducted bidirectional mendelian randomization analysis for 24 pairwise traits between gastrointestinal tract diseases and psychiatric disorders to investigate the potential causal trait pairs (ie, vertical pleiotropy), along with negative control analysis using AMD and cataract. We used the inverse-variance weighted method^54^ as main analysis and in total 6 alternative mendelian randomization methods with different model assumptions as additional analyses for further validation. For main analysis, we chose a false discovery rate approach for multiple testing correction with the significance threshold being false discovery rate–adjusted *P* < .05, given that the commonly used Bonferroni correction is often too stringent for multiple nonindependent tests. The nominally significant threshold (*P* < .05) was used for alternative mendelian randomization methods. The Latent Heritable Confounder Mendelian Randomization (LHC-MR) method, which could account for sample overlap, was used for pairwise traits with potential sample overlap to further validate the mendelian randomization results.^55^

### Genomic Loci Characterization and Functional Annotation

For significant pleiotropic SNVs from PLACO, we applied FUMA to identify independent variants, characterize genomic risk loci, and annotate the functions of variants using LD information from the 1000 Genome Project phase 3 reference panel of European population.^42^ We characterized independent SNVs with *r*^2^ less than 0.6000 and lead SNVs with *r*^2^ less than 0.1000 within 1 Mb. Genomic risk loci were defined by merging genomic regions if the physical distance between lead SNVs was less than 250 kilobase.^42^

Functional annotations, including ANNOVAR software tool categories (Bioinformatics), combined annotation-dependent depletion (CADD) scores, and RegulomeDB scores, were also provided by FUMA. Single-nucleotide variations with a CADD score larger than 12.37 were considered a potentially deleterious variant.^42^ Single-nucleotide variations with *P* < 5 × 10^−8^ in each single-trait GWAS were also annotated by FUMA for comparison.

## Results

### Genetic Correlations and Genetic Overlaps Between Gastrointestinal Tract Diseases and Psychiatric Disorders

Using genome-wide association summary statistics from publicly available data sources, pervasive significant genome-wide genetic correlations and genetic overlaps were found across 24 pairwise traits, among which 14 were identified from LDSC and 21 were identified from GPA (Table 1). Notably, 8 trait pairs were identified with nonsignificant genetic correlation but with significant genetic overlap, producing a final union set of 22 pairwise traits for subsequent analysis. In addition, the results from the LDSC were highly consistent with those from high-definition likelihood (eTable 2 and eFigure 1 in Supplement 1) and suggested significant sample overlap for 5 trait pairs (Table 1). For negative control analysis, no significant genetic correlations were detected as expected (eTable 3 in Supplement 1).

**Table 1.** Genetic Correlation and Genetic Overlap Estimations Between 24 Pairwise Traits^a^

Trait pair	Genetic correlation				Genetic overlap		
	Genetic correlation (SE)	*P* value for LDSC	Intercept (SE)	*P* value for intercept	PM 11	PAR^b^	*P* value for GPA
IBD-MDD^c^^,^^d^	0.1706 (0.0407)	2.82 × 10^−5^	0.0003 (0.0055)	9.57 × 10^−1^	0.0116	0.0473	1.41 × 10^−3^
IBD-PTSD	0.1742 (0.0377)	7.39 × 10^−2^	0.0049 (0.0051)	3.37 × 10^−1^	0.0038	0.0189	2.27 × 10^−1^
IBD-SCZ^d^	0.0359 (0.0403)	3.73 × 10^−1^	0.0004 (0.0062)	9.49 × 10^−1^	0.0167	0.0605	6.07 × 10^−15^
IBD-ADHD	–0.0045 (0.0599)	9.40 × 10^−1^	0.0121 (0.0057)	3.38 × 10^−2^	0.0121	0.0512	5.16 × 10^−2^
IBD-BIP^d^	0.0221 (0.0478)	6.45 × 10^−1^	0.0064 (0.0063)	3.10 × 10^−1^	0.0194	0.0828	3.80 × 10^−28^
IBD-AN^d^	–0.0296 (0.0630)	6.38 × 10^−1^	0.0019 (0.0063)	7.63 × 10^−1^	0.0206	0.0851	8.85 × 10^−11^
IBS-MDD^c^^,^^d^^,^^e^	0.5705 (0.0256)	1.14 × 10^−109^	0.0795 (0.0066)	2.05 × 10^−33^	0.1571	0.7791	<1 × 10^−300^
IBS-PTSD^c^^,^^d^^,^^e^	0.4720 (0.0795)	2.87 × 10^−9^	0.0275 (0.0049)	2.00 × 10^−8^	0.0422	0.1504	1.27 × 10^−5^
IBS-SCZ^c^^,^^d^	0.1711 (0.0285)	1.85 × 10^−9^	–0.0025 (0.006)	6.77 × 10^−1^	0.1246	0.4130	<1 × 10^−300^
IBS-ADHD^c^^,^^d^	0.2060 (0.0405)	3.73 × 10^−7^	0.0138 (0.0059)	1.93 × 10^−2^	0.1155	0.3895	1.67 × 10^−87^
IBS-BIP^c^^,^^d^^,^^e^	0.1269 (0.0305)	3.12 × 10^−5^	0.0219 (0.0058)	1.59 × 10^−4^	0.1183	0.4579	3.54 × 10^−268^
IBS-AN^c^^,^^d^	0.1536 (0.0413)	2.00 × 10^−4^	0.0003 (0.0064)	9.63 × 10^−1^	0.1207	0.4059	3.98 × 10^−86^
PUD-MDD^c^^,^^d^^,^^e^	0.4438 (0.0437)	3.31 × 10^−24^	0.0333 (0.0059)	1.66 × 10^−8^	0.0987	0.4011	1.04 × 10^−102^
PUD-PTSD^c^	0.5448 (0.1120)	1.15 × 10^−6^	0.0074 (0.0046)	1.08 × 10^−1^	0.0274	0.1052	3.65 × 10^−2^
PUD-SCZ^c^^,^^d^	0.1306 (0.0402)	1.20 × 10^−3^	–0.0007 (0.006)	9.07 × 10^−1^	0.0832	0.2713	7.89 × 10^−55^
PUD-ADHD^c^^,^^d^	0.4771 (0.0579)	1.63 × 10^−16^	0.0072 (0.0058)	2.14 × 10^−1^	0.0886	0.3409	1.27 × 10^−37^
PUD-BIP^d^	0.0691 (0.0437)	1.14 × 10^−1^	0.0023 (0.0054)	6.70 × 10^−1^	0.0679	0.2491	1.31 × 10^−34^
PUD-AN^d^	0.0366 (0.0539)	4.97 × 10^−1^	0.0014 (0.0056)	8.03 × 10^−1^	0.0448	0.1441	8.03 × 10^−4^
GERD–MDD^c^^,^^d^^,^^e^	0.4596 (0.0267)	3.50 × 10^−66^	0.0692 (0.0053)	5.83 × 10^−39^	0.1762	0.6824	<1 × 10^−300^
GERD-PTSD^c^^,^^d^	0.4193 (0.0690)	1.24 × 10^−9^	0.0156 (0.0051)	2.22 × 10^−3^	0.0538	0.1567	3.43 × 10^−4^
GERD-SCZ^d^	0.0313 (0.0273)	2.51 × 10^−1^	0.0107 (0.0062)	8.44 × 10^−2^	0.1299	0.3596	1.07 × 10^−189^
GERD-ADHD^c^^,^^d^	0.4919 (0.0371)	3.32 × 10^−40^	–0.0003 (0.0063)	9.62 × 10^−1^	0.1543	0.4844	6.81 × 10^−132^
GERD-BIP^d^	0.0332 (0.0308)	2.81 × 10^−01^	0.0048 (0.0059)	4.16 × 10^−1^	0.1279	0.4050	1.27 × 10^−174^
GERD-AN^d^	0.0179 (0.0386)	6.43 × 10^−1^	0.0038 (0.0063)	5.46 × 10^−1^	0.1265	0.3597	1.24 × 10^−61^

Abbreviations: ADHD, attention-deficit/hyperactivity disorder; AN, anorexia nervosa; BIP, bipolar disorder; GERD, gastroesophageal reflux disease; GPA, genetic analysis incorporating pleiotropy and annotation method; IBD, inflammatory bowel disease; IBS, irritable bowel syndrome; LDSC, linkage disequilibrium score regression; MDD, major depressive disorder; PAR, pleiotropy association ratio; PM 11, proportion of genetic variants associated with both traits; PTSD, posttraumatic stress disorder; PUD, peptic ulcer disease; SCZ, schizophrenia.

^a^ Genetic correlation and genetic overlap were estimated by LDSC and GPA methods, respectively. Bonferroni-corrected significance threshold was set at *P* < 2.83 × 10^−3^ (.05/24), producing a final union set of 22 pairwise traits with significant genetic correlation or genetic overlap for subsequent analysis.

^b^ We introduced PAR as PM 11/(PM 10 + PM 01 + PM 11) to represent the proportion of pleiotropic single-nucleotide variations (SNVs) associated with both traits against the proportion of SNVs associated with at least 1 trait.

^c^ Pairwise trait with significant genetic correlation.

^d^ Pairwise trait with significant genetic overlap.

^e^ Genome-wide association study summary data with potentially significant sample overlap.

### Shared Loci Between Gastrointestinal Tract Diseases and Psychiatric Disorders

**Figure 2.**
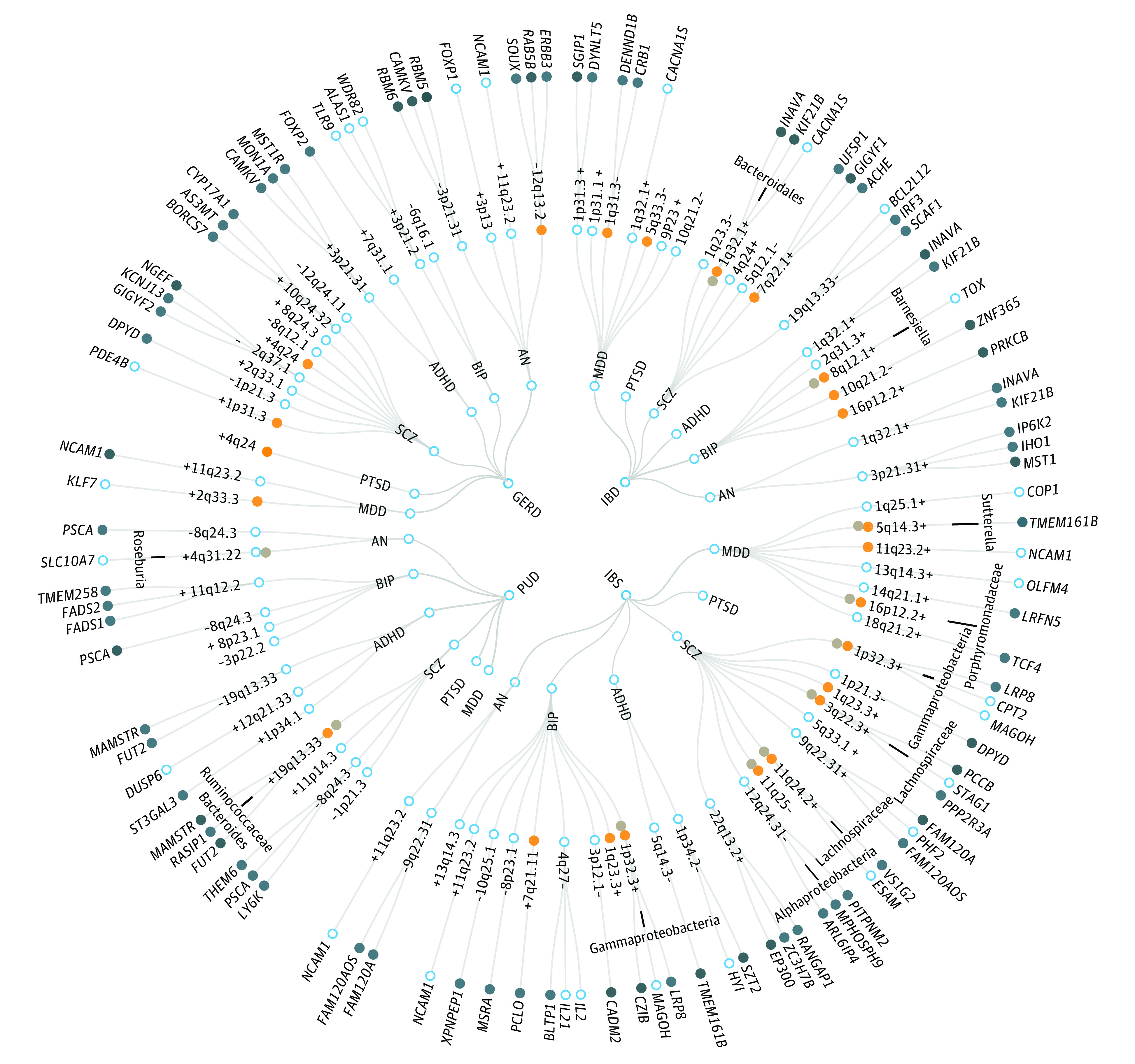
The Overall Landscape of the Pleiotropic Associations Across 4 Gastrointestinal Tract Diseases and 6 Psychiatric Disorders A circular dendrogram included 4 gastrointestinal tract diseases (inner circle, including gastroesophageal reflux disease [GERD], inflammatory bowel disease [IBD], irritable bowel syndrome [IBS], and peptic ulcers disease [PUD]) and 6 psychiatric disorders (second circle; attention-deficit/hyperactivity disorder [ADHD], anorexia nervosa [AN], bipolar disorder [BIP], major depressive disorder [MDD], posttraumatic stress disorder [PTSD], and schizophrenia [SCZ]), resulting in 24 trait pairs. A total of 83 pleiotropic loci were identified across 19 trait pairs (third circle; no pleiotropic loci were identified for IBS-PTSD, PUD-MDD, and PUD-PTSD), in which 53 of 83 top single nucleotide variations (SNVs) showed concordant associations (marked by a plus sign) with a certain pair of traits while the remaining top SNVs showed discordant associations (marked by a minus sign); 24 pleiotropic loci were colocalized for pairwise traits (orange dots, posterior probability of H4 [PP.H4]>0.7); and 11 were colocalized for certain gastrointestinal tract diseases, psychiatric disorders, and gut microorganisms (gray dots, PP>0.7, with the specific microorganism annotated on the lines). A total of 196 significant pleiotropic genes (158 unique) were further identified by multimarker analysis of GenoMic annotation (MAGMA). For the trait pairs with more than 3 pleiotropic genes, we only showed the top 3 pleiotropic genes according to the prioritization of candidate pleiotropic genes, which were shown with the statistical prioritization attenuation manifested by clockwise direction (fourth circle), the genes with the tissue specificity in at least one of gastrointestinal tract or brain tissues and in other tissues identified by E-MAGMA were also highlighted (dark blue dots). Detailed information for 83 pleiotropic loci (eTable 6 in Supplement 1), colocalization results (Table 2), multitrait colocalization results (eTable 17 Supplement 1), significant pleiotropic genes with annotations of tissue specificity (eTable 15 in Supplement 1) was also provided.

A total of 2910 SNVs (2284 unique) were identified as potential pleiotropic variants by PLACO in 19 trait pairs (eFigure 2 in Supplement 1). FUMA identified 83 independent genomic risk loci as pleiotropic loci, involving 54 unique chromosomal regions (Figure 2 and eTables 4 and 5 in Supplement 1). Some pleiotropic regions were identified in multiple pairwise traits, such as 11q23.2 (mapped gene: *NCAM1* [OMIM 116930]) and 1q32.1 (mapped gene: *INAVA* [OMIM 618051]), suggesting the extensive pleiotropic effects of these loci. The top SNVs in 83 FUMA-annotated pleiotropic loci showed mixed directions of allelic associations (eTable 6 in Supplement 1). Overall, 53 of 83 top SNVs (64%) showed concordant associations with a certain pair of traits, indicating these variants could simultaneously decrease or increase the risk of gastrointestinal tract diseases and psychiatric disorders. The remaining 30 of 83 top SNVs (36%) showed discordant associations, suggesting the possibly distinct biological mechanisms.

ANNOVAR category annotation illustrated that 34 of 83 index SNVs (41%) were intronic variants and 27 of 83 (33%) were intergenic variants. Only 7 of 83 index SNVs (8%) (6 unique) were exonic variants, including 5 messenger RNA (mRNA) exonic variants and 2 noncoding RNA exonic variants (eTable 5 in Supplement 1). Of note, 5 mRNA exonic variants (4 unique) were located in 3 loci and mapped to 3 genes. Specifically, the index SNV rs20551 at 22q13.2 locus (PLACO *P* = 5.07 × 10^−11^ for IBS-schizophrenia) was a significant eQTL for *EP300* (OMIM 602700) encoding p300 protein, which plays an important role in cell proliferation and differentiation. The index SNVs rs681343 (PLACO *P* = 1.36 × 10^−12^ for PUD-schizophrenia) and rs601338 (PLACO *P* = 5.07 × 10^−11^ for PUD-ADHD) at 22q13.2 were in high LD (*r*^2^ = 0.996) and had almost identical eQTL regulation information (eTable 7 in Supplement 1), regulating the expression of *FUT2* (OMIM 182100). The index SNV rs13107325 at 4q24 locus (PLACO *P* = 1.78 × 10^−14^ for GERD-schizophrenia; PLACO *P* = 4.19 × 10^−8^ for GERD-PTSD) was a significant eQTL for *SLC39A8* (OMIM 608732), which encodes ZIP8 metal cation transporter. In addition, 7 index SNVs (6 unique) were identified with CADD scores larger than 12.37, in which 2 mRNA exonic variants had higher CADD scores, including rs601338 (CADD score: 52; mapped gene: *FUT2* [OMIM 182100]) and rs13107325 (CADD score: 34; mapped gene: *SLC39A8*).

Further colocalization analysis identified 24 of 83 potential pleiotropic loci (29%) with PP.H4 larger than 0.7, in which 22 top SNVs of corresponding loci were identified as candidate shared causal variants (Table 2, Figure 2, and eFigure 3 in Supplement 1). Interestingly, the 19q13.33 locus, which was identified as pleiotropic loci for 2 pairs of traits, was only colocalized between PUD and schizophrenia (PP.H4 = 0.8927) rather than PUD and ADHD (PP.H4 = 0.3552), with the same potential shared causal variant rs681343 identified (mapped gene: *FUT2*). In addition, 7 pleiotropic loci were identified with PP.H3 larger than 0.7000, indicating there might be different causal variants in these loci (eTable 8 in Supplement 1).

**Table 2.** 24 Colocalized Loci Identified by Colocalization Analysis Performed on 83 Pleiotropic Loci

Trait pair	Top SNV	Locus boundary^a^	Region	Nearest gene	PP.H3	PP.H4	Best causal	SNV.PP.H4
IBD-MDD	rs12118513	1:197342380–197781198	1q31.3	*DENND1B*	0.0395	0.7372	rs12118513^b^	0.2022
IBD-MDD	rs60689680	5:158827769–158856513	5q33.3	*AC008703.1*	0.0614	0.7034	rs60689680^b^	0.1184
IBD-SCZ	rs905634	1:200874229–201027055	1q32.1	*INAVA*	0.0813	0.9139	rs905634^b^	0.4835
IBD-SCZ	rs492430	7:100219167–100523241	7q22.1	*EPO*	0.0801	0.9116	rs492430^b^	0.1452
IBD-BIP	rs56073120	8:59800835–59925249	8q12.1	*TOX*	0.0435	0.9044	rs56073120^b^	0.0632
IBD-BIP	rs7090073	10:64387108–64441247	10q21.2	*ZNF365*	0.0257	0.9719	rs7090073^b^	0.1992
IBD-BIP	rs196001	16:23892887–23962504	16p12.2	*PRKCB*	0.0656	0.8724	rs196001^b^	0.0811
IBS-MDD	rs3099439	5:87514778–87822672	5q14.3	*TMEM161B*	0.0536	0.9428	rs3099439^b^	0.2131
IBS-MDD	rs4937872	11:112826867–112912811	11q23.2	*RP11-629G13.1*	0.0845	0.9030	rs4937872^b^	0.1163
IBS-MDD	rs1862743	16:60665658–60743834	16p12.2	*GNPATP*	0.0108	0.9578	rs1862743^b^	0.4157
IBS-SCZ	rs12031155	1:53658317–53752134	1p32.3	*LRP8*	0.0518	0.9368	rs12031155^b^	0.1577
IBS-SCZ	rs7542202	1:163616199–163766672	1q23.3	*RP4-640E24.1*	0.0550	0.9299	rs7542202^b^	0.0691
IBS-SCZ	rs1280622	3:135807609–136673157	3q22.3	*RP11-731C17.1*	0.1145	0.8386	rs7432375	0.0965
IBS-SCZ	rs11604175	11:124619407–124624854	11q24.2	*VSIG2*	0.0011	0.7717	rs11604175^b^	0.5730
IBS-SCZ	rs12277680	11:134576216–134595774	11q25	*RP11-469N6.2*	0.0106	0.7331	rs12277680^b^	0.4057
IBS-BIP	rs5177	1:53658317–53752134	1p32.3	*LRP8*	0.0553	0.8665	rs5177^b^	0.1081
IBS-BIP	rs2345964	1:163582980–163768927	1q23.3	*RP4-640E24.1*	0.0254	0.9684	rs2345964^b^	0.1043
IBS-BIP	rs13239217	7:82387493–82583609	7q21.11	*PCLO*	0.0939	0.8493	rs13239217^b^	0.1919
PUD-SCZ	rs681343	19:49103447–49254955	19q13.33	*FUT2*	0.0951	0.8927	rs681343^b^	0.2834
GERD-MDD	rs1263674	2:208017033–208088987	2q33.3	*AC007879.1:AC007879.2*	0.0567	0.8596	rs62188630	0.0638
GERD-PTSD	rs13107325	4:102938709–103438709	4q24	*SLC39A8*	0.0111	0.7904	rs13107325^b^	0.7793
GERD-SCZ	rs1892346	1:66304167–66333877	1p31.3	*PDE4B*	0.0210	0.8141	rs1892346^b^	0.0854
GERD-SCZ	rs13107325	4:102702364–103387161	4q24	*SLC39A8*	0.0020	0.9966	rs13107325^b^	0.8466
GERD-AN	rs1873914	12:56368708–56478658	12q13.2	*RAB5B*	0.0484	0.8134	rs1873914^b^	0.1042

Abbreviations: ADHD, attention-deficit/hyperactivity disorder; AN, anorexia nervosa; Best causal, the candidate causal single-nucleotide variation (SNV); BIP, bipolar disorder; GERD, gastroesophageal reflux disease; IBD, inflammatory bowel disease; IBS, irritable bowel syndrome; MDD, major depressive disorder; PP.H3, posterior probability of H3; PP.H4, posterior probability of H4; PTSD, posttraumatic stress disorder; PUD, peptic ulcer disease; SCZ, schizophrenia; SNV.PP.H4, posterior probability of the best causal variant.

^a^ Locus boundary of each pleiotropic genomic risk locus was denoted as “chromosome: start-end” defined by FUMA for the corresponding trait pair.

^b^ The top SNV in this locus was also identified as a candidate causal SNV.

### Prioritization of Candidate Pleiotropic Genes and Characterization of Phenotype and Tissue Specificity

MAGMA analysis based on 295 potential pleiotropic genes that located in or overlapped with 83 pleiotropic loci identified 196 significant pleiotropic genes (158 unique), in which 38 genes were detected in 2 or more trait pairs (eTable 9 in Supplement 1). For example, *NCAM1* was identified as a significant pleiotropic gene in 5 pairs of traits, followed by *INAVA*, *CACNA1S* (OMIM 114208), and *KIF21B* (OMIM 608322) in 4 pairs of traits and *BANK1* (OMIM 610292), *CAMKV* (OMIM 614993), *DPYD* (OMIM 612779), *MON1A* (OMIM 611464), *MST1R* (OMIM 600168), *PSCA* (OMIM 602470), *RBM5* (OMIM 606884), *RBM6* (OMIM 606886), and *SLC39A8* in 3 pairs of traits.

**Figure 3.**
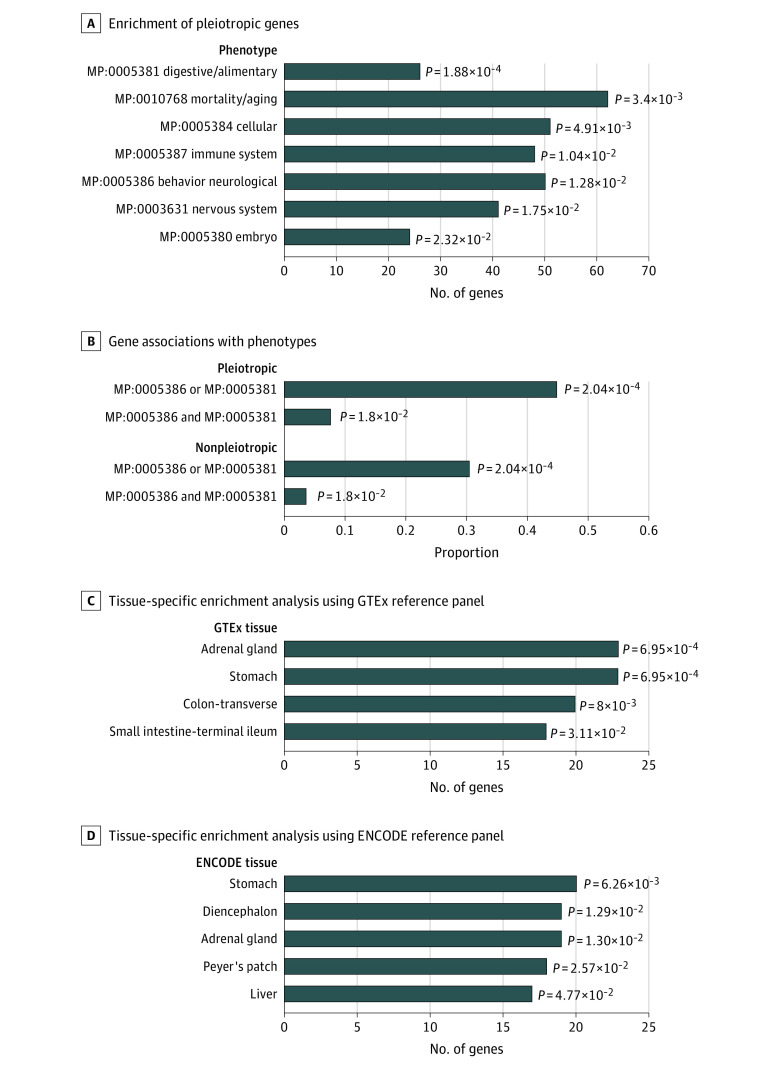
Phenotype and Tissue Specificity for Candidate Pleiotropic Genes Phenotype enrichment analysis based on existing mouse/human orthology with phenotype annotations from the Mouse Genome Informatics platform suggested 7 phenotypes in which the significant pleiotropic genes were enriched (A) and both the proportions of the genes associated with at least 1 of 2 gut-brain axis–related phenotypes or associated with both phenotypes (behavior-neurological phenotype and digestive-alimentary phenotype) in the pleiotropic gene group were higher (B). Detailed information of phenotype annotations for these genes was provided in eTable 10 in Supplement 1. Tissue-specific enrichment analysis using the deTS method based on 2 reference panels showed higher tissue specificity, 4 significantly enriched tissues were identified when using the Genotype-Tissue Expression project (GTEx) reference panel (C), and 5 were identified when using the Encyclopedia of DNA Elements project (ENCODE) reference panel (D). Detailed information of tissue-specific genes was provided in eTables 11 and 12 in Supplement 1. We declared the nominally significant threshold (2-sided *P* < .05) in both analyses.

Further parallel phenotype and tissue-specific enrichment analysis suggested both higher phenotype and tissue specificity (Figure 3). Specifically, we obtained in total 19 326 genes with available phenotype annotations resorting to Mouse Genome Informatics platform, in which 144 of 158 unique pleiotropic genes were included. These pleiotropic genes were associated with several GBA-related phenotypes with the analyses performed on each phenotype (Figure 3A) and were associated with the 2 phenotypes simultaneously (behavior-neurological phenotype and digestive-alimentary phenotype), contrasting with that of nonpleotropic genes with less association with these phenotypes (7.64% vs 3.61%; *P* = 1.80 × 10^−2^) (Figure 3B). Details of these genes were provided in eTable 10 in Supplement 1. Tissue-specific enrichment analysis using deTS based on GTEx and ENCODE reference panels suggested higher tissue specificity (Figure 3) in several gastrointestinal tract and brain tissues, such as stomach, transverse colon, terminal ileum of small intestine, Peyer’s patch, and diencephalon. The hypothalamic-pituitary-adrenal axis is an important component of the GBA and could provide primary biological response to stressful stimuli, with adrenal gland especially highlighted. Details of these tissue-specific genes were also provided (eFigure 4 and eTables 11 and 12 in Supplement 1).

In addition, E-MAGMA and joint-tissue imputation analysis also confirmed the tissue specificity not only in gastrointestinal tract and brain tissues but also in other GBA-related tissues. In total, 52 pleiotropic genes (22 unique) across 8 pleiotropic loci were significantly detected in multiple GBA-related tissues in at least 2 trait pairs, for example, *PSCA* (8q24.3) for 3 pairwise traits (PUD-schizophrenia, PUD-BIP, and schizophrenia-AN), *LPR8* (1p32.3) for 2 pairwise traits (IBS-schizophrenia and IBS-BIP), and *FUT2* (19q13.33) for 2 pairwise traits (PUD-schizophrenia and PUD-ADHD). H-MAGMA analysis further suggested the cell-type specificity of these pleiotropic genes. Details of these gene-level analyses were also provided (eTables 13-15 in Supplement 1).

### Synaptic and Immune-Related Mechanisms Shared Between Gastrointestinal Tract Diseases and Psychiatric Disorders

Gene set enrichment analysis identified 127 significantly enriched pathways (normalized enrichment score >2 and adjusted *P* < .05), including 99 Gene Ontology (GO) terms and 28 Kyoto Encyclopedia of Genes and Genomes (KEGG) pathways (eTable 16 in Supplement 1). These enriched pathways are mainly involved in cell adhesion, synaptic structure and function, and immune cell differentiation. For example, homophilic cell adhesion via plasma membrane adhesion molecules (GO:0007156), which plays important roles in neural and immune mechanisms, was identified in 5 pairs of traits. A γ-aminobutyric acid (GABA)–modifying synapse (GO:0098982), which typically functions as an inhibitory synapse using GABA as neurotransmitter, was significantly enriched in 3 pairs of traits. Notably, both T helper 1 (T_H_1) and T_H_2 cell differentiation (KEGG has04658) and T_H_17 cell differentiation (KEGG has04659), which involve in immunoinflammatory responses, were highlighted in 2 pairs of traits.

### Multitrait Colocalization Analysis to Pinpoint Critical Gut Microbiomes

Multitrait colocalization analysis from HyPrColoc highlighted 15 pleiotropic loci (11 unique) that were colocalized to share a causal variant, supporting the significant role of gut microorganisms (Figure 2 and eTable 17 in Supplement 1). Seven of 11 unique loci were identified to be colocalized among schizophrenia, gastrointestinal tract diseases, and gut microorganisms. For example, 19q13.33 was particularly colocalized between PUD and schizophrenia not only with the prevalence of *Ruminococcaceae* species but with the abundance of *Bacteroides* species, where the index SNV rs681343 (mapped gene: *FUT2*) was also identified as shared causal variant.

### Mendelian Randomization and Associations Between Gastrointestinal Tract Diseases and Psychiatric Disorders

Bidirectional mendelian randomization analyses using inverse-variance weighted method showed 11 significant positive associations (eFigure 5 and eTable 18 in Supplement 1). In addition, no significant bidirectional associations were detected in negative control analysis (eTable 19 in Supplement 1). The results from several alternative mendelian randomization methods were largely consistent with that from main analysis (eTables 18 and 19 in Supplement 1). Notably, among 5 pairwise traits with potential sample overlap, LHC-MR further validated the results (eTable 20 in Supplement 1).

## Discussion

In this genome-wide pleiotropic association study, we found extensive genome-wide genetic correlations and genetic overlaps between gastrointestinal tract diseases and psychiatric disorders. Further comprehensive analyses highlighted the pleiotropic genetic variants and loci, potential shared causal variants, pleiotropic genes, biological pathways, and a potential genetic basis associated with gut microbiome. All these findings supported the role of GBA in the shared genetic etiology underlying these 2 types of diseases.

Using multiple methods with different model assumptions could provide complementary evidence and allow deep investigation of the underlying pleiotropic associations from different perspectives. The GPA identified substantial genetic overlap between several trait pairs even without any significant genetic correlation. The mixed directions across the genomic risk loci, which indicate the existence of concordant and discordant pleiotropy, also help explain the polygenic overlap despite nonsignificant genetic correlation. PLACO analysis further determines the pleiotropic variants, followed by colocalization analysis to identify potential shared causal variants in each pleiotropic locus. Mendelian randomization analysis could detect causal trait pairs and partially reflect vertical pleiotropy. Of note, we conducted LDSC to examine potential sample overlap and performed pleiotropic analysis using PLACO as well as mendelian randomization analysis using LHC-MR to alleviate sample overlap issue. The LDSC and mendelian randomization analyses were of comparable consistency with previous studies, with detailed comparisons provided (eTables 21-23 in Supplement 1).

Overall, pleiotropic variants between gastrointestinal tract diseases and psychiatric disorders were extensively distributed, with several loci especially highlighted between certain trait pairs, such as 1q32.1 (*INAVA*), 19q13.33 (*FUT2*), 11q23.2 (*NCAM1*), and 1p32.3 (*LRP8*). Several loci previously identified to be associated with gastrointestinal tract diseases were illustrated to be potential pleiotropic loci shared with psychiatric disorders and vice versa (eDiscussion in Supplement 1). The pleiotropic genes were more likely to be enriched in GBA-related phenotypes, such as behavioral, neurological, and digestive phenotypes, as well as GBA-related tissues, especially gastrointestinal and brain tissues.

Shared genetic determinants also reflect common biological pathways, among which the T_H_1, T_H_2, and T_H_17 cell differentiation pathways were highlighted between IBD and schizophrenia, BIP, and AN. The T_H_17 cells play important roles in the development of inflammatory responses and autoimmune diseases.^56,57,58^ Previous studies suggested the significant role of dysfunction of T_H_17 cell differentiation and the accumulation of T_H_17-related cytokines in the pathological process of IBD.^59,60^ In addition, T_H_17 cells and related cytokines would provoke CNS neuroinflammation and neurotoxicity under pathological status and disrupt the blood-brain barrier and thus alter the permeability of the blood-brain barrier.^61,62^ Bacterially produced bile acid metabolites have been shown to inhibit T_H_17 cell differentiation, which may be related to the pathophysiology of IBD.^63^ Therefore, T_H_17 cell differentiation and function play a vital role in the regulation of brain-gut-microbiome axis.

### Limitations

Our study is not without limitations. First, we were unable to assess the causal effects of gut microbiome on gastrointestinal tract diseases or psychiatric disorders through mendelian randomization analyses since SNVs with associated gut microorganisms were hard to obtain due to the limited sample size of gut microbiome GWAS. Therefore, caution should be used on the interpretation of the role of gut microorganisms. Second, our study was restricted to European ancestry and may not generalize to other ancestries.

## Conclusions

Given strong evidence of genetic correlation and genetic overlap between gastrointestinal tract diseases and psychiatric disorders, we found that pleiotropic genetic variants, loci, and genes were extensively distributed across the genome, with higher phenotype and tissue specificity. We highlighted some genetic determinants previously associated with gastrointestinal tract diseases that were shared with psychiatric disorders and vice versa. More importantly, shared biological mechanisms concerning immune response, synaptic structure and function, and potential gut microbiome were identified. Our findings not only support the shared genetic basis underlying GBA but provide novel insight into the intervention and treatment targets of these diseases.

## Notes

Supplement 1. **eMethods,** Study Analyses **eDiscussion.** Study Outcomes **eFigure 1.** Fourteen Pairs of Traits With Significant Genetic Correlations Identified by Both HDL and LDSC Methods **eFigure 2.** Quantile-Quantile (Q-Q) Plots of PLACO Results for 22 Pairwise Traits **eFigure 3.** LocusZoom and LocusCompare Plots of 24 Significantly Colocalized Loci **eFigure 4.** Gene Expression Heatmap of 158 Significant Pleiotropic Genes in 25 Tissues **eFigure 5.** The Bidirectional Causal Effects Estimated by IVW Method **eTable 1.** Details of GWAS Summary Data Sources **eTable 2.** Genetic Correlations Between 4 Gastrointestinal Tract Diseases and 6 Psychiatric Disorders Estimated by HDL **eTable 3.** Bivariate LDSC Estimates in Negative Control Analysis **eTable 4.** Summary of Genome-Wide Significant Pleiotropic SNVs and FUMA-Annotated Pleiotropic Genomic Risk Loci for Each Pair of Traits **eTable 5.** 83 Pleiotropic Genomic Loci Identified by FUMA Using PLACO Results **eTable 6.** Effect Sizes and *P* Values of Top SNPs in 83 Pleiotropic Loci From Original GWAS Summary Statistics **eTable 7.** The eQTL Regulatory Information of rs601338 and rs681343 on *FUT2* Gene in Gastrointestinal Tract and Brain Tissues **eTable 8.** The Remaining 59 Loci in Colocalization Analysis **eTable 9.** Candidate Pleiotropic Genes Identified by MAGMA **eTable 10.** Phenotype Enrichment Results With Existing Phenotype Annotations of the Pleiotropic Genes **eTable 11.** Pleiotropic Genes Identified With Tissue Specificity in GTEx Reference Panel **eTable 12.** Pleiotropic Genes Identified With Tissue Specificity in ENCODE Reference Panel **eTable 13.** Twenty-Five Tissue Types Used for E-MAGMA Analysis **eTable 14.** Six Tissue/Cell Types Used for H-MAGMA Analysis **eTable 15.** Tissue Specificity and Cell-Type Specificity of the Identified Pleiotropic Genes in E-MAGMA, JTI, and H-MAGMA Analysis **eTable 16.** Significantly Enriched GO and KEGG Pathways in GSEA Analysis **eTable 17.** Significantly Colocalized Loci Identified by Multitrait Colocalization Using HyPrColoc **eTable 18.** Results of Bidirectional Mendelian Randomization Analysis From Main Analysis and Alternative Methods Between 4 Gastrointestinal Diseases and 6 Psychiatric Disorders **eTable 19.** Results of Bidirectional Mendelian Randomization Analysis From Main Analysis and Alternative Methods for Negative Control Analysis **eTable 20.** Results of Mendelian Randomization Analysis Using LHC-MR Method **eTable 21.** Comparisons of Data Sources of Psychiatric Disorders-Related GWAS in Genetic Correlation and Mendelian Randomization Analysis **eTable 22.** Summary of Genetic Correlation Results Between 4 Gastrointestinal Tract Diseases and Psychiatric Disorders **eTable 23.** Summary of Associations Between Gastrointestinal Tract Diseases and Psychiatric Disorders in Mendelian Randomization Analysis
eReferences.

Supplement 2.
Data Sharing Statement
