# Supplementary material for: Role of the Gut-Brain Axis in the Shared Genetic Etiology Between Gastrointestinal Tract Diseases and Psychiatric Disorders: A Genome-Wide Pleiotropic Analysis
Source: JAMA Psychiatry. 2023 Feb 8;80(4):360–70. doi: 10.1001/jamapsychiatry.2022.4974 (PMC9909581; doi:10.1001/jamapsychiatry.2022.4974)
Supplement: Supplement 1. — eMethods, Study Analyses eDiscussion. Study Outcomes eFigure 1. Fourteen Pairs of Traits With Significant Genetic Correlations Identified by Both HDL and LDSC Methods eFigure 2. Quantile-Quantile (Q-Q) Plots of PLACO Results for 22 Pairwise Traits eFigure 3. LocusZoom and LocusCompare Plots of 24 Significantly Colocalized Loci eFigure 4. Gene Expression Heatmap of 158 Significant Pleiotropic Genes in 25 Tissues eFigure 5. The Bidirectional Causal Effects Estimated by IVW Method eTable 1. Details of GWAS Summary Data Sources eTable 2. Genetic Correlations Between 4 Gastrointestinal Tract Diseases and 6 Psychiatric Disorders Estimated by HDL eTable 3. Bivariate LDSC Estimates in Negative Control Analysis eTable 4. Summary of Genome-Wide Significant Pleiotropic SNVs and FUMA-Annotated Pleiotropic Genomic Risk Loci for Each Pair of Traits eTable 5. 83 Pleiotropic Genomic Loci Identified by FUMA Using PLACO Results eTable 6. Effect Sizes and P Values of Top SNPs in 83 Pleiotropic Loci From Original GWAS Summary Statistics eTable 7. The eQTL Regulatory Information of rs601338 and rs681343 on FUT2 Gene in Gastrointestinal Tract and Brain Tissues eTable 8. The Remaining 59 Loci in Colocalization Analysis eTable 9. Candidate Pleiotropic Genes Identified by MAGMA eTable 10. Phenotype Enrichment Results With Existing Phenotype Annotations of the Pleiotropic Genes eTable 11. Pleiotropic Genes Identified With Tissue Specificity in GTEx Reference Panel eTable 12. Pleiotropic Genes Identified With Tissue Specificity in ENCODE Reference Panel eTable 13. Twenty-Five Tissue Types Used for E-MAGMA Analysis eTable 14. Six Tissue/Cell Types Used for H-MAGMA Analysis eTable 15. Tissue Specificity and Cell-Type Specificity of the Identified Pleiotropic Genes in E-MAGMA, JTI, and H-MAGMA Analysis eTable 16. Significantly Enriched GO and KEGG Pathways in GSEA Analysis eTable 17. Significantly Colocalized Loci Identified by Multitrait Colocalization Using HyPrColoc eTable 18. Results of Bidi [file jamapsychiatry-e224974-s001.pdf]

Supplementary Online Content

Gong W, Guo P, Li Y, et al. Role of the gut-brain axis in the shared genetic etiology between gastrointestinal tract diseases and psychiatric disorders: a genome-wide pleiotropic analysis. *JAMA Psychiatry*. Published online February 8, 2023. doi:10.1001/jamapsychiatry.2022.4974

eMethods, Study Analyses

eDiscussion. Study Outcomes

eFigure 1. Fourteen Pairs of Traits With Significant Genetic Correlations Identified by Both HDL and LDSC Methods

eFigure 2. Quantile-Quantile (Q-Q) Plots of PLACO Results for 22 Pairwise Traits

eFigure 3. LocusZoom and LocusCompare Plots of 24 Significantly Colocalized Loci

eFigure 4. Gene Expression Heatmap of 158 Significant Pleiotropic Genes in 25 Tissues

eFigure 5. The Bidirectional Causal Effects Estimated by IVW Method

eTable 1. Details of GWAS Summary Data Sources

eTable 2. Genetic Correlations Between 4 Gastrointestinal Tract Diseases and 6 Psychiatric Disorders Estimated by HDL

eTable 3. Bivariate LDSC Estimates in Negative Control Analysis

eTable 4. Summary of Genome-Wide Significant Pleiotropic SNVs and FUMA-Annotated Pleiotropic Genomic Risk Loci for Each Pair of Traits

eTable 5. 83 Pleiotropic Genomic Loci Identified by FUMA Using PLACO Results

eTable 6. Effect Sizes and *P* Values of Top SNPs in 83 Pleiotropic Loci From Original GWAS Summary Statistics

eTable 7. The eQTL Regulatory Information of rs601338 and rs681343 on *FUT2* Gene in Gastrointestinal Tract and Brain Tissues

eTable 8. The Remaining 59 Loci in Colocalization Analysis

eTable 9. Candidate Pleiotropic Genes Identified by MAGMA

eTable 10. Phenotype Enrichment Results With Existing Phenotype Annotations of the Pleiotropic Genes

eTable 11. Pleiotropic Genes Identified With Tissue Specificity in GTEx Reference Panel

eTable 12. Pleiotropic Genes Identified With Tissue Specificity in ENCODE Reference Panel

eTable 13. Twenty-Five Tissue Types Used for E-MAGMA Analysis

©2023 Gong W et al. *JAMA Psychiatry*.

eTable 14. Six Tissue/Cell Types Used for H-MAGMA Analysis

eTable 15. Tissue Specificity and Cell-Type Specificity of the Identified Pleiotropic Genes in E-MAGMA, JTI, and H-MAGMA Analysis

eTable 16. Significantly Enriched GO and KEGG Pathways in GSEA Analysis

eTable 17. Significantly Colocalized Loci Identified by Multitrait Colocalization Using HyPrColoc

eTable 18. Results of Bidirectional Mendelian Randomization Analysis From Main Analysis and Alternative Methods Between 4 Gastrointestinal Diseases and 6 Psychiatric Disorders

eTable 19. Results of Bidirectional Mendelian Randomization Analysis From Main Analysis and Alternative Methods for Negative Control Analysis

eTable 20. Results of Mendelian Randomization Analysis Using LHC-MR Method

eTable 21. Comparisons of Data Sources of Psychiatric Disorders-Related GWAS in Genetic Correlation and Mendelian Randomization Analysis

eTable 22. Summary of Genetic Correlation Results Between 4 Gastrointestinal Tract Diseases and Psychiatric Disorders

eTable 23. Summary of Associations Between Gastrointestinal Tract Diseases and Psychiatric Disorders in Mendelian Randomization Analysis

eReferences.

This supplementary material has been provided by the authors to give readers additional information about their work.

eMethods. Study Analyses

GWAS data quality control

Genome-wide association study (GWAS) datasets for 4 gastrointestinal tract (GIT) diseases, 6 psychiatric (PSY) disorders, and 2 eye disorders (a common set of negative controls) used in this study have undergone stringent quality control, detailed descriptions of inclusion criteria and quality control procedures have been provided in the original publications.[1-10] In addition, we performed further quality control on these GWAS summary data by: (i) aligning to hg19 human reference genomes; (ii) filtering single nucleotide polymorphisms (SNPs) without rsID or with duplicated rsID; (iii) excluding SNPs in major histocompatibility complex region (MHC, chr 6: 25–35 Mb) due to its complex LD structure; (iv) keeping biallelic SNPs with minor allele frequency (MAF) > 0.01. Additional data processing procedures were carried out according to the corresponding requirements of different methods in subsequent analyses.

Genetic correlation analysis

We used both linkage disequilibrium score regression (LDSC)[11] and high-definition likelihood (HDL)[12] to assess genome-wide genetic correlations for 24 pairwise traits between 4 GIT diseases and 6 psychiatric (PSY) disorders. The intercept estimated from LDSC could also indicate potential sample overlap between two GWASs. Compared to LDSC only accounting for partial LD information, HDL is able to fully account for LD across the genome and greatly improve the estimation precision. In addition, LDSC was also performed between 2 eye disorders mentioned above and a total of 10 GIT and PSY traits, serving as negative control analysis.

Genetic correlation analyses were performed according to the standard analysis process of LDSC and HDL. We performed LDSC using well-imputed HapMap3 variants (http://ldsc.broadinstitute.org/static/media/w_hm3.noMHC.snplist.zip) and pre-computed LD scores of European ancestry from the 1000 Genomes Project Phase3 (https://data.broadinstitute.org/alkesgroup/LDSCORE/eur_w_ld_chr.tar.bz2).[11] We did not constrain the intercepts in LDSC analysis, which could not only account for residual confounding but also indicate whether there was potential sample overlap between two GWAS studies. We performed HDL using R package HDL-v1.4.0 (https://github.com/zhenin/HDL), taking 1,029,876 well-imputed HapMap3 SNPs as reference panel (https://github.com/zhenin/HDL/wiki/Reference-panels).[12]

Genetic overlap analysis

Given that the genetic correlation analysis only reflects the overall correlation across the genome between traits, we further applied GPA (Genetic analysis incorporating Pleiotropy and Annotation)[13] to explore the overall genetic overlap. For each trait pair, GPA relies on four distinct models to classify SNPs into four categories, aims to estimate the proportions of SNPs in each model, and uses likelihood ratio test to assess the statistical significance for overall genetic overlap.[13] GPA assumes that *p*-values

from null SNPs (not associated with the trait) follow the uniform distribution and non-null SNPs (associated with the trait) follow the Beta distribution, then extends the assumption to two GWASs and proposes four models (M_{00}, M_{10}, M_{01}, and M_{11}) to classify these SNPs into four categories: (i) SNPs associated with neither of traits; (ii) SNPs only associated with the first trait; (iii) SNPs only associated with the second trait; (iv) SNPs associated with both traits. GPA aims to estimate the proportions of SNPs in these models (PM) and uses likelihood ratio test (LRT) to assess the statistical significance for overall genetic overlap.[13] Note that the proportion of risk SNPs should not be extremely small to enable GPA to work well.[13] To alleviate the influence of LD on GPA, we performed LD pruning based on the 1000 Genomes Phase 3 European-ancestry genotypes using PLINK1.9 to obtain relatively independent SNPs.

Pairwise pleiotropic analysis using PLACO

For the union set of pairwise traits with significant genetic correlation or genetic overlap, we used the recently developed pleiotropic analysis under composite null hypothesis (PLACO), which could account for potential correlation between two traits, to identify pleiotropic SNPs.[14] For a given variant, PLACO detects pleiotropic associations by considering a composite null hypothesis, where the null hypothesis H_0 is a composite of the global null {$beta_{trait1} = beta_{trait2} = 0$}, and the sub-null hypotheses are {$beta_{trait1} = 0$, $beta_{trait2} \neq 0$} and {$beta_{trait1} \neq 0$, $beta_{trait2} = 0$}. That is, PLACO tests H_0: $beta_{trait1} \times beta_{trait2} = 0$ vs H_1: $beta_{trait1} \times beta_{trait2} \neq 0$, and the test statistic of PLACO is $T_{PLACO} = Z_{trait1}Z_{trait2}$.[14] For each trait pair, we denote trait1 and trait2 as GIT disease and PSY disorder, $beta_{trait1}$ and $beta_{trait2}$ as the effect sizes of a SNP on two traits, Z_{trait1} and Z_{trait2} as the observed Z-scores of a SNP from corresponding GWAS summary data, respectively. The rejection of H_0 statistically suggests that the SNP would be a potential pleiotropic variant shared between two traits. Overlapped SNPs between GWASs of each pairwise traits were included and the summary statistics were harmonized to align to same effect allele. SNPs with squared Z-scores above 80 were removed since extremely large effect sizes could produce spurious signals.[14] We de-correlated the Z-scores using the correlation matrix estimated from GWAS summary statistics to account for potential sample overlap. SNPs with $P_{PLACO} < 5 \times 10^{-8}$ were declared as significant pleiotropic variants.

Bayesian colocalization analysis using COLOC

For FUMA-annotated pleiotropic loci, we performed a Bayesian colocalization analysis using R package coloc-v5.1.2[15] to further identify potential shared causal variants in each pleiotropic locus for the corresponding pairwise traits. Colocalization analysis relies on single causal variant assumption and the posterior probability (PP) for five hypotheses at each pleiotropic locus would be provided: (i) H0: neither trait has a genetic association in the region; (ii) H1: only trait 1 has a genetic association in the region; (iii) H2: only trait 2 has a genetic association in the region; (iv) H3: both traits are associated, but with different causal variants; (v) H4: both traits are associated and share a single causal variant.[15] We performed colocalization analysis by coloc.abf function under the default setting ($p_1 = p_2 = 1 \times 10^{-4}$,

©2023 Gong W et al. *JAMA Psychiatry*.

$p_{12} = 1 \times 10^{-5}$), where p_1 and p_2 represent the prior probability of a SNP to be associated with trait 1 and trait 2, respectively, and p_{12} represents the prior probability of a SNP to be associated with both traits. With posterior probability (PP) for each mutually exclusive hypothesis provided, we declared a genomic locus with PP.H4 > 0.7 as a colocalized locus with potential shared causal variant. Besides, the SNP with the largest PP.H4 in this locus would be determined as a candidate causal variant.

Gene-level analysis

We first performed gene-level Multi-marker Analysis of GenoMic Annotation (MAGMA)[16] on the genes located in or overlapped with the pleiotropic loci based on both PLACO results and single-trait GWAS to identify candidate pleiotropic genes. MAGMA Gene IDs and locations of 19 427 protein-coding genes based on NCBI build 37.3 were downloaded from https://ctg.cncr.nl/software/magma. The significance was declared at both the locus-specific Bonferroni-corrected *P*-value < 0.05 for MAGMA analysis on PLACO results and *P*-values < 0.05 for both MAGMA analyses based on original single-trait GWAS of corresponding GIT disease and PSY disorder.

To further investigate certain biological implications of these pleiotropic genes, we performed two parallel enrichment analyses, including phenotype enrichment analysis and tissue-specific enrichment analysis. Specifically, phenotype enrichment analysis was performed based on the "Mouse/Human Orthology with Phenotype Annotations" from Mouse Genome Informatics platform[17] (MGI, http://www.informatics.jax.org/), to characterize the phenotype specificity of these pleiotropic genes against that of non-pleotropic genes by examining the differences in the proportions of genes associated with certain phenotypes in the pleiotropic gene group against that in the non-pleotropic gene group using Fisher's exact test. The analyses were first performed in a total of 27 phenotypes, respectively, followed by the analysis focusing on the two phenotypes (behavior/neurological phenotype and digestive/alimentary phenotype) to investigate the phenotype enrichment of the pleiotropic genes associated with at least one of these two phenotypes as well as associated with both phenotypes simultaneously, to further characterize the phenotype specificity. Then, we performed tissue-specific enrichment analyses to illustrate the tissue specificity of these pleiotropic genes using *deTS* method[18] based on two different reference panels, Genotype-Tissue Expression project (GTEx, 14 725 protein-coding, non-housekeeping genes in 47 tissues) and the Encyclopedia of DNA Elements project (ENCODE, 14 031 protein-coding, non-housekeeping genes in 44 tissues). We declared the significance with a nominally significant threshold ($P < 0.05$) for the parallel phenotype and tissue enrichment analysis.

In addition, based on the significantly enriched tissues implicated in tissue-specific enrichment analysis, we leveraged E-MAGMA[19] and transcriptome-wide association study (TWAS) analysis using joint-tissue imputation (JTI)[20] to further investigate the tissue-specific genes, parallelized with H-MAGMA (Hi-C coupled MAGMA)[21] to indicate the cell-type specificity. Specifically, a total of 25 tissues were included (13 brain tissues, 7 gastrointestinal tissues, whole blood, and 4 additional tissues including pituitary, adrenal gland, liver, and EBV-transformed lymphocytes), in which tissue-specific

annotations used for E-MAGMA were obtained from https://github.com/eskederks/eMAGMA-tutorial and the multi-tissue gene expression predictive models used for JTI were downloaded from Zenodo (https://doi.org/10.5281/zenodo.3842289), both were derived from Genotype-Tissue Expression project version 8 (GTEx v8). Additionally, we applied H-MAGMA, which advances MAGMA by exploiting chromatin interaction profiles from human brain tissue to assign noncoding SNPs to their cognate genes based on long-range interactions, to further characterize the cell-type specificity of the pleiotropic genes. Six Hi-C annotation files (fetal brain Hi-C, adult brain Hi-C, iPSC-derived neuron Hi-C, iPSC-derived astrocyte Hi-C, cortical neuronal Hi-C, adult midbrain dopaminergic Hi-C) were obtained from https://github.com/thewonlab/H-MAGMA. We declared the significance with locus-specific Bonferroni correction according to the actual number of tests.

Gene set enrichment analysis

Using gene sets derived from Gene Ontology (GO) and Kyoto Encyclopedia of Genes and Genomes (KEGG) pathway database, gene set enrichment analysis (GSEA) was performed on MAGMA outputs to identify potential biological pathways using clusterProfiler package.[22] GSEA algorithm would determine whether the genes in a gene set are clustered at the top or bottom of or randomly distributed in a ranked gene list.[23] Specifically, GSEA scans the ranked gene list from the top to the bottom to calculate the enrichment score (ES) of a gene set, ES increases when a gene is in the gene set and decreases otherwise, leading to final ES as the maximum deviation from zero in this process. Then, a normalized enrichment score (NES) accounting for the differences in gene set sizes and in correlations between gene sets would be calculated, where positive and negative NES values represent the enrichment at the top and bottom of the ranked gene list, respectively. NES could be used to compare results across gene sets, and the *P*-values were obtained by permutation test. The gene list used for GSEA analysis was sorted by descending order of Z-scores from MAGMA. Significantly enriched pathways were declared with NES > 2 and $P_{adjust} < 0.05$.

Multi-trait colocalization analysis using HyPrColoc

Given the significant role of gut microbiome in GBA regulated mechanisms, we further performed multi-trait colocalization analysis on each FUMA-annotated pleiotropic loci using GWASs of pairwise traits and human host-microbiome GWAS (mGWAS) by HyPrColoc (Hypothesis Prioritisation for multi-trait Colocalization) method[24] to identify potential shared causal variants among these pairwise traits and gut microbiome, thus to indicate the potential biological mechanisms involving certain microorganisms. Again, a genomic locus with PP larger than 0.7 was declared as a colocalized locus. HyPrColoc, as an extension of the colocalization method mentioned above, allows colocalization analysis for multiple traits, which adopts deterministic Bayesian divisive clustering algorithm to identify clusters of colocalized traits and candidate causal variants in a genomic locus and provides the posterior probability of colocalization for each cluster.[24]

Gut microbiome GWASs for 430 microbiome features used in this study were obtained from a recent study performed on 8 956 German individuals,[25] including gut microbiome GWASs performed on 198 univariate microbial features based on presence-absence patterns of microorganisms using logistic

©2023 Gong W et al. *JAMA Psychiatry*.

regression and 232 univariate microbial features based on abundance of microorganisms using linear regression. Univariate microbial features were defined according to taxonomic annotations from phylum to genus, and classifications below the genus level were defined by sequence similarity clustering (97% and 99% similarity) and amplicon sequence variants (ASVs).[25] Specifically, for each pleiotropic loci, we extracted the GWAS summary statistics from corresponding GIT disease PSY disorder and one of the 430 microbial features to perform HyPrColoc, resulting in a total of 35 690 colocalization analyses (83 loci × 430 microbial features).

Mendelian randomization analysis

Using inverse variance weighted method (IVW) as main analysis, several alternative MR methods under different assumptions were also used as sensitivity analyses to further validate the results: (i) MR-Egger,[26] which relies on INstrument Strength Independent of Direct Effect (InSIDE) assumption and can provide the causal effect estimate as well as test for pleiotropy; (ii) Weighted median,[27] which is robust to instrumental outliers; (iii) Weighted mode,[28] which is also outlier-robust; (iv) IVW method using robust regression (MR-Robust),[29] which downweights the outliers to improve the estimation accuracy of causal effect; (v) MR Robust Adjusted Profile Score (MR-RAPS),[30] which is robust to both systematic and idiosyncratic pleiotropy; (vi) MR-Pleiotropy Residual Sum and Outlier (MR-PRESSO),[31] which can identify and remove outliers with horizontal pleiotropic effects. All bidirectional MR analyses implemented for each pair of traits (e.g., IBD–SCZ) using these standard MR methods were based on following procedures to select independent instrumental variants. We first selected the genome-wide significant SNPs with $P < 5 \times 10^{-8}$, then used 1000 Genomes Project phase 3 of European population as LD reference panel to obtain independent instrumental variants with $r^2 < 0.001$ or physical distance > 10,000 kb. For those data sets without at least 2 independent SNPs at a threshold of $P < 5 \times 10^{-8}$, including PTSD and CAT, the threshold was relaxed to $P < 5 \times 10^{-6}$. Then we used phenotype variance explained by genetic variants (PVE) and F statistics to assess the strength of genetic associations of instrumental SNPs and the issue of weak instrument bias.

©2023 Gong W et al. *JAMA Psychiatry*.

eDiscussion. Study Outcomes

Several loci previously identified to be associated with GIT diseases were illustrated to be potential pleiotropic loci shared with PSY disorders. For example, *INAVA* (1q32.1), a critical susceptibility gene of IBD[32,33] was identified to be shared between IBD and SCZ as well as IBD and BIP, which would regulate the stability of epithelial adherens junctions thus influence the intestinal permeability and host susceptibility to pathogen infections.[34,35] An experimental study demonstrated the impairments of intestinal epithelial cell barrier in *INAVA*-deficient mice.[34] To date, no studies have reported the associations of *INAVA* with PSY disorders. This locus was also colocalized with certain gut microbiome *Bacteroidales*, suggesting a possible biological mechanism might be the interaction of defects in intestinal epithelial function regulated by *INAVA* and disturbance of gut microbiome. *FUT2* (19q13.33) was especially highlighted to be shared between PUD and SCZ as well as PUD and ADHD with distinct index SNPs. The genotype of rs601338 determines *FUT2* secretor status (non-secretor: AA; secretor: AG or GG) which encodes the enzyme α-1,2-L-fucosyltransferase 2, influencing the secretion of histo-blood group antigens on mucosal surfaces which could serve as host receptor sites to alter host susceptibility to infections of some pathogens.[36] Interestingly, the index SNP rs681343-T would increase the risk of both PUD and SCZ, while the index SNP rs601338-A would increase the risk PUD but decrease the risk of ADHD. Besides, this locus was not only colocalized between PUD and SCZ but also highlighted as a colocalized locus with the features of *Ruminococcaceae* and *Bacteroides*, in line with previous study that *FUT2* was strongly associated with features of gut microbiome.[25]

Several loci previously identified to be associated with PSY diseases were illustrated to be potential pleiotropic loci shared with GIT disorders. *NCAM1* (11q23.2) was suggested to be shared between GORD and MDD. *NCAM1* encodes for neural cell adhesion molecule 1, which is a member of the immunoglobulin superfamily and implicated in neuron-neuron adhesion and the development of the nervous system. *NCAM1* is highly expressed both in CNS and intestinal nerve fibers and ganglia of ENS.[37] The encoded protein could play an important role in altering the morphology and strength of synaptic connections to regulate neuronal activity.[38] An animal experiment showed that depression-like phenotype in Ncam-deficient mice could be reversed by *NCAM*-derived peptides.[39] The bidirectional causal associations between GORD and MDD also indicates the existence of possible genetic confounder that drive such associations. *LRP8* (1p32.3), which has been reported to be associated with SCZ and BIP, was identified to be shared between IBS and SCZ as well as IBS and BIP. The potential shared genetic variants between IBS and BIP in this locus is rs5177, which is a significant eQTL regulating the expression of *LRP8*. *LRP8* encodes for low-density lipoprotein receptor-related protein 8 which participates in Reelin signaling pathway. Reelin acts via both the very low-density lipoprotein receptor and *LRP8* to regulate *DAB1* tyrosine phosphorylation and microtubule function in neurons.[40] It has been demonstrated that Reelin-*LRP8* pathway modulates synaptic plasticity events central to learning and memory.[40]

©2023 Gong W et al. *JAMA Psychiatry*.

eFigure 1. Fourteen Pairs of Traits With Significant Genetic Correlations Identified by Both HDL and LDSC Methods

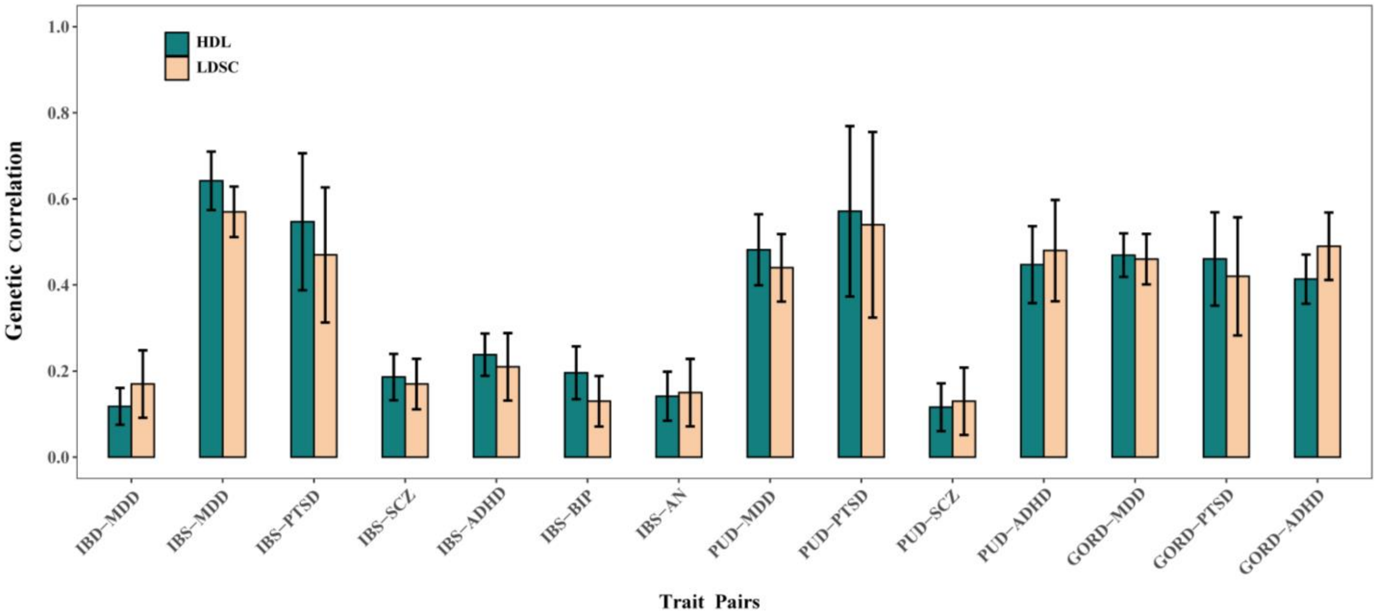

Error bars in black showed the 95% confidence intervals of genetic correlation estimates. HDL indicates high-definition likelihood; LDSC, linkage disequilibrium score regression; IBD, inflammatory bowel disease; IBS, irritable bowel syndrome; PUD, peptic ulcers disease; GORD, gastro-oesophageal reflux disease; MDD, major depressive disorder; PTSD, post-traumatic stress disorder; SCZ, schizophrenia; ADHD, attention deficit hyperactivity disorder; BIP, bipolar disorder; AN, anorexia nervosa.

©2023 Gong W et al. *JAMA Psychiatry*.

eFigure 2. Quantile-Quantile (Q-Q) Plots of PLACO Results for 22 Pairwise Traits

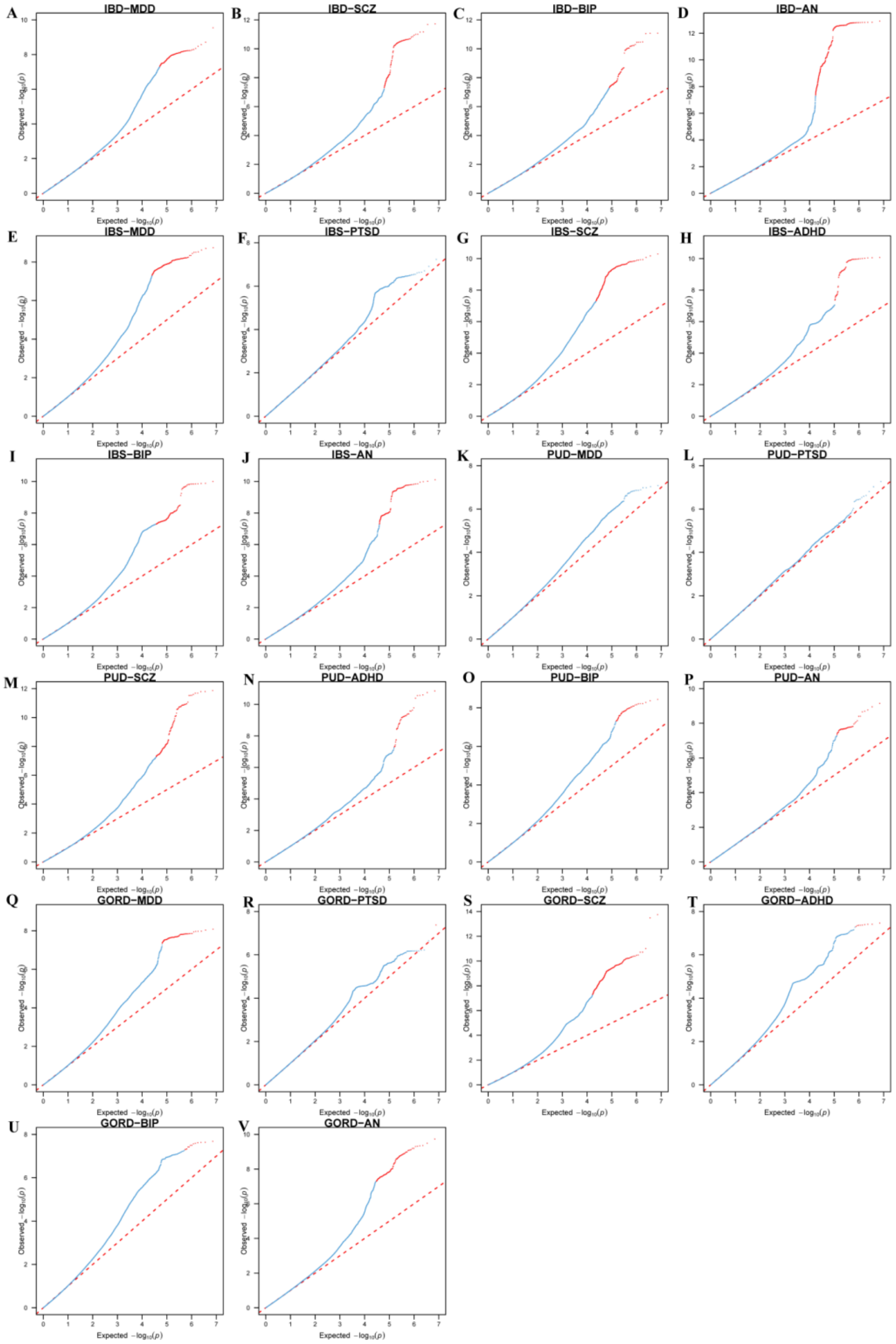

©2023 Gong W et al. *JAMA Psychiatry*.

Q-Q plots depicts expected –log10 P-values (x-axis) against observed –log10 P_{PLACO}-values (y-axis). Red dots indicate significant pleiotropic variants ($P_{PLACO} < 5 \times 10^{-8}$). Note that no pleiotropic loci were identified for three trait pairs, including IBS–PTSD, PUD–MDD, and PUD–PTSD. IBD indicates inflammatory bowel disease; IBS, irritable bowel syndrome; PUD, peptic ulcers disease; GORD, gastro-oesophageal reflux disease; MDD, major depressive disorder; PTSD, post-traumatic stress disorder; SCZ, schizophrenia; ADHD, attention deficit hyperactivity disorder; BIP, bipolar disorder; AN, anorexia nervosa.

eFigure 3. LocusZoom and LocusCompare Plots of 24 Significantly Colocalized Loci

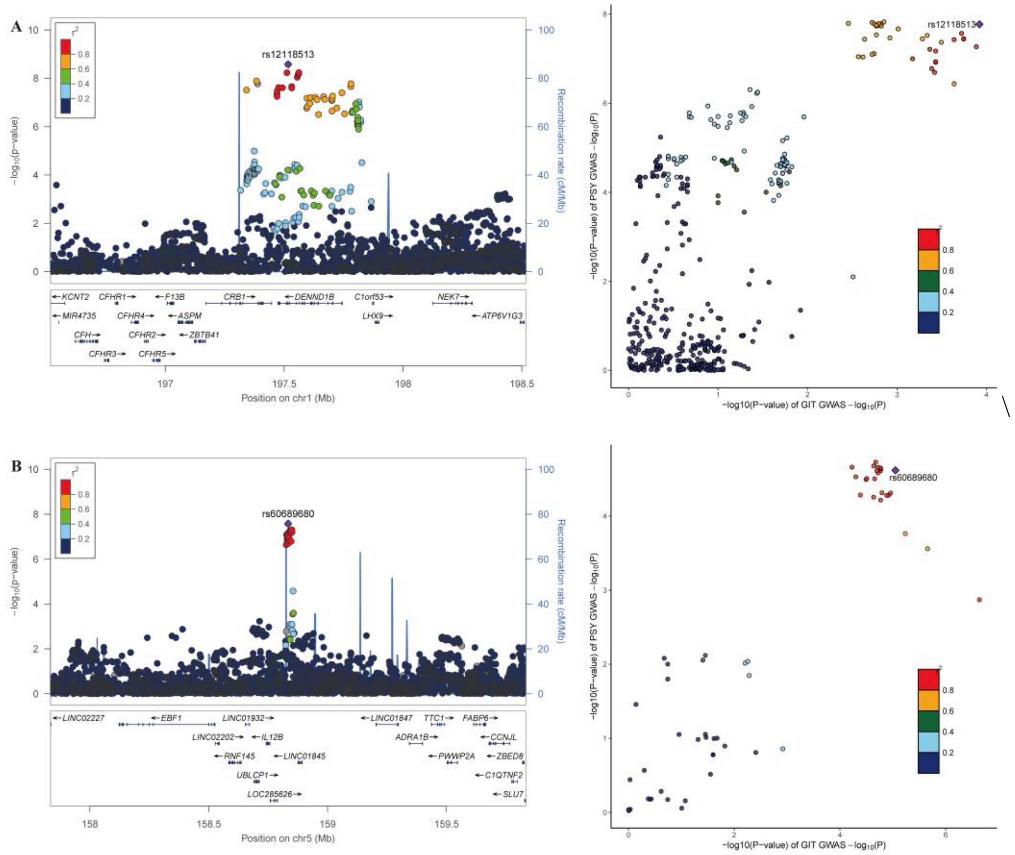

©2023 Gong W et al. *JAMA Psychiatry*.

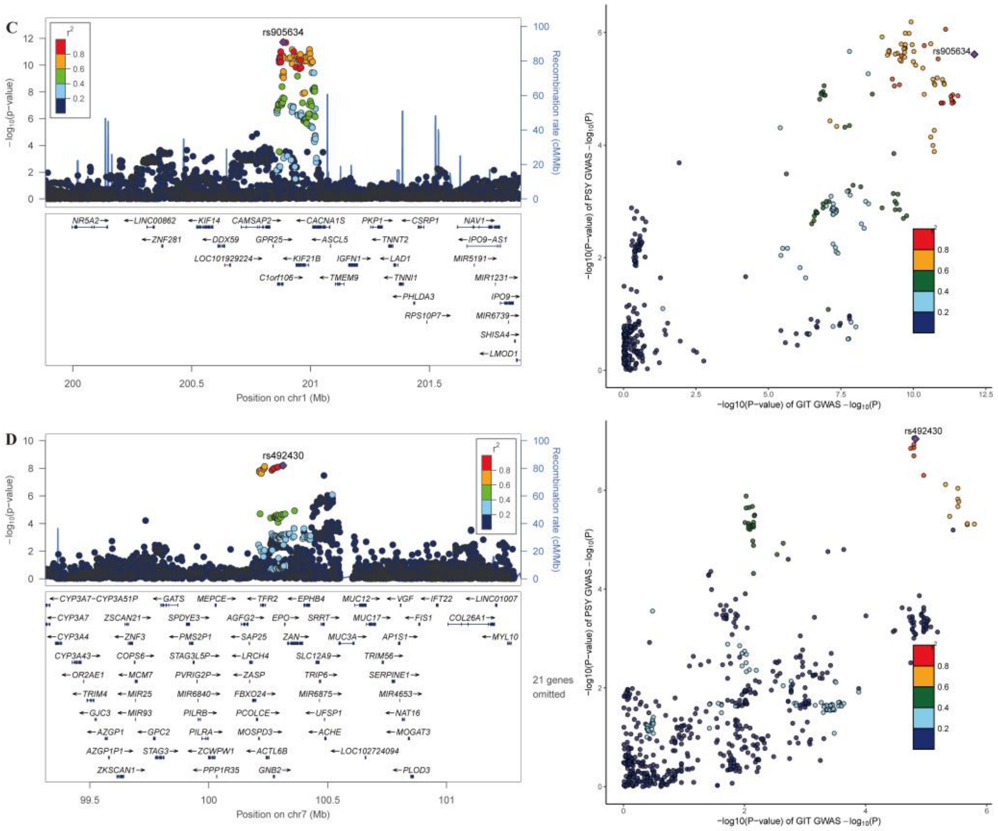

©2023 Gong W et al. *JAMA Psychiatry*.

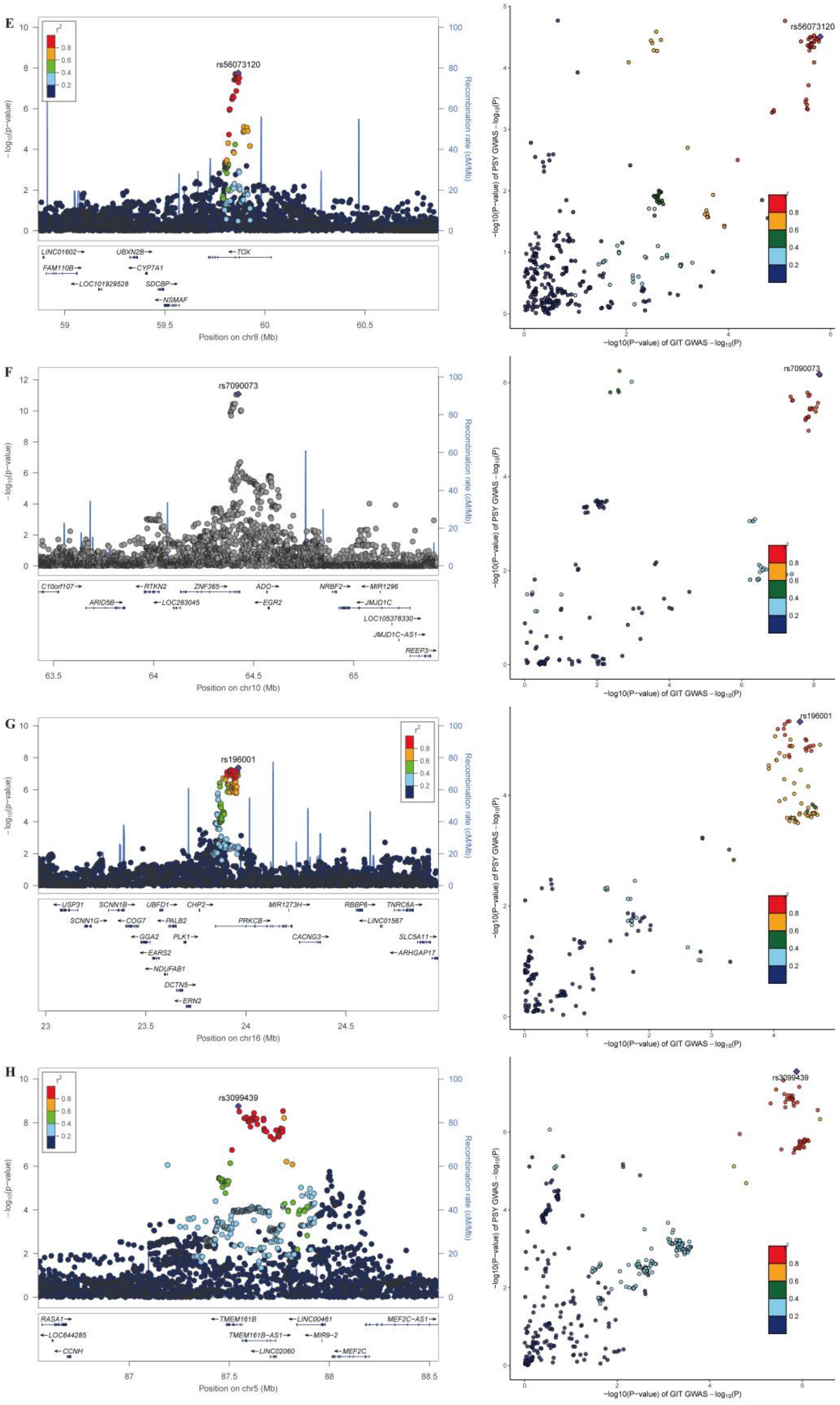

©2023 Gong W et al. *JAMA Psychiatry*.

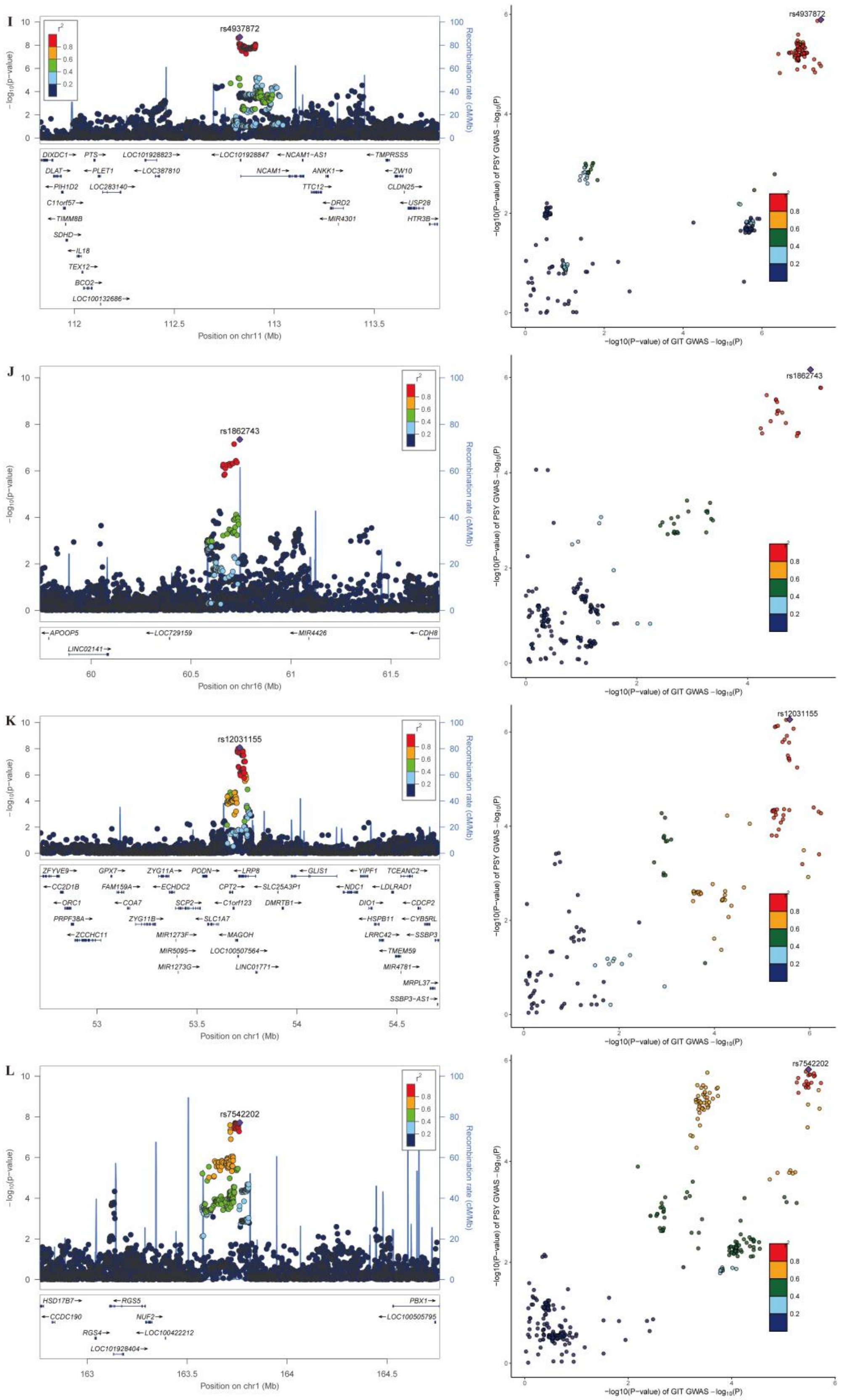

©2023 Gong W et al. *JAMA Psychiatry*.

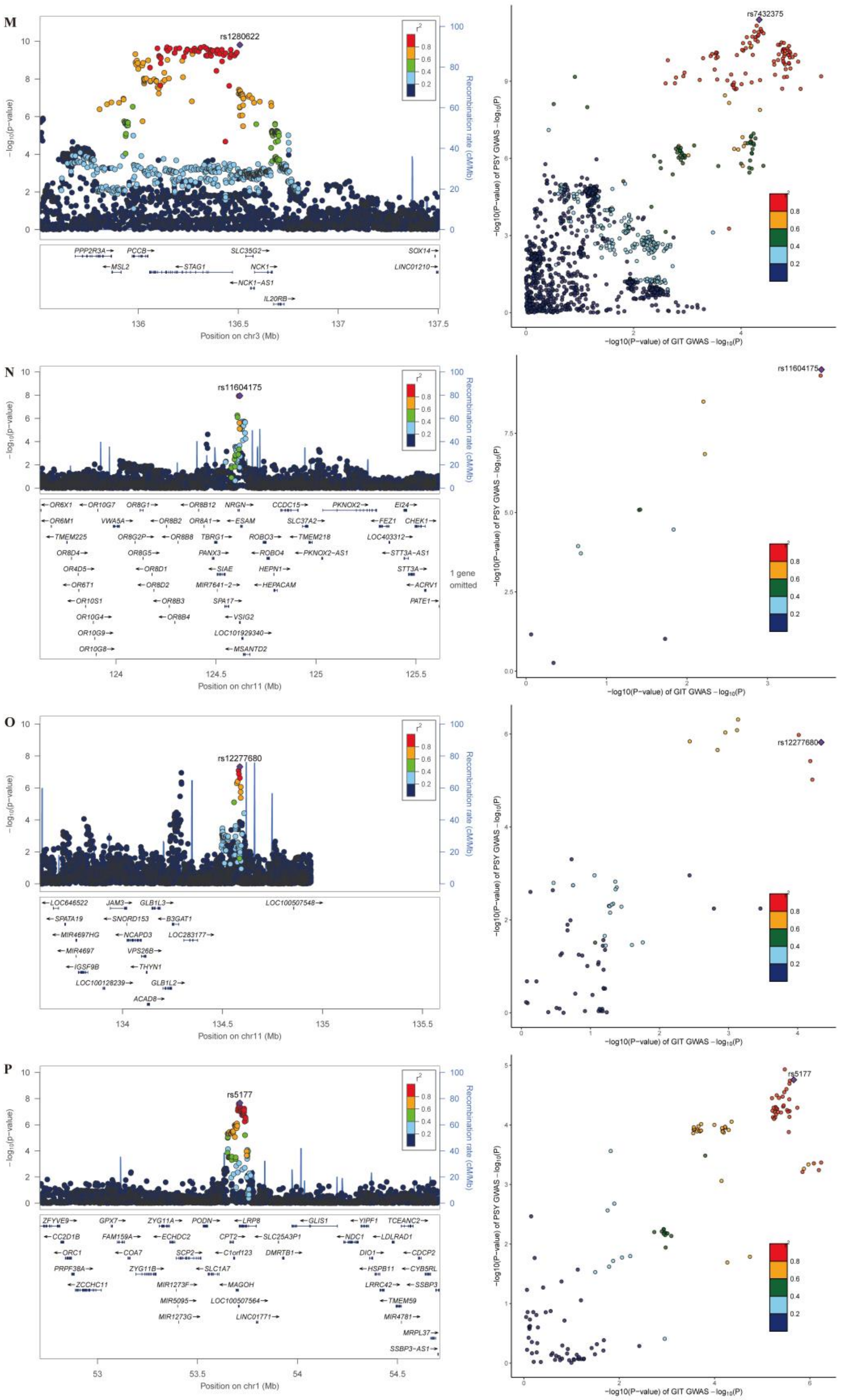

©2023 Gong W et al. *JAMA Psychiatry*.

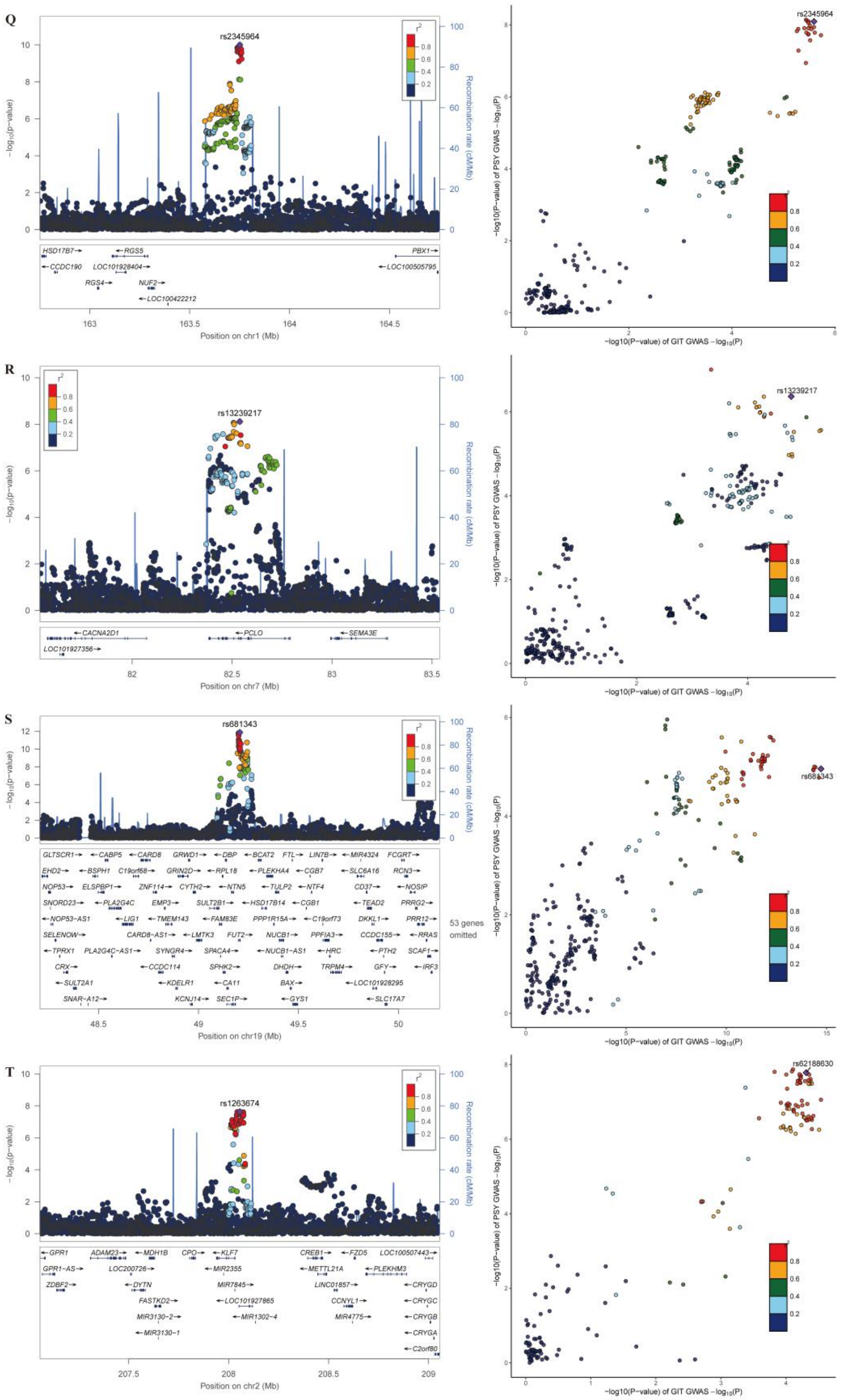

©2023 Gong W et al. *JAMA Psychiatry*.

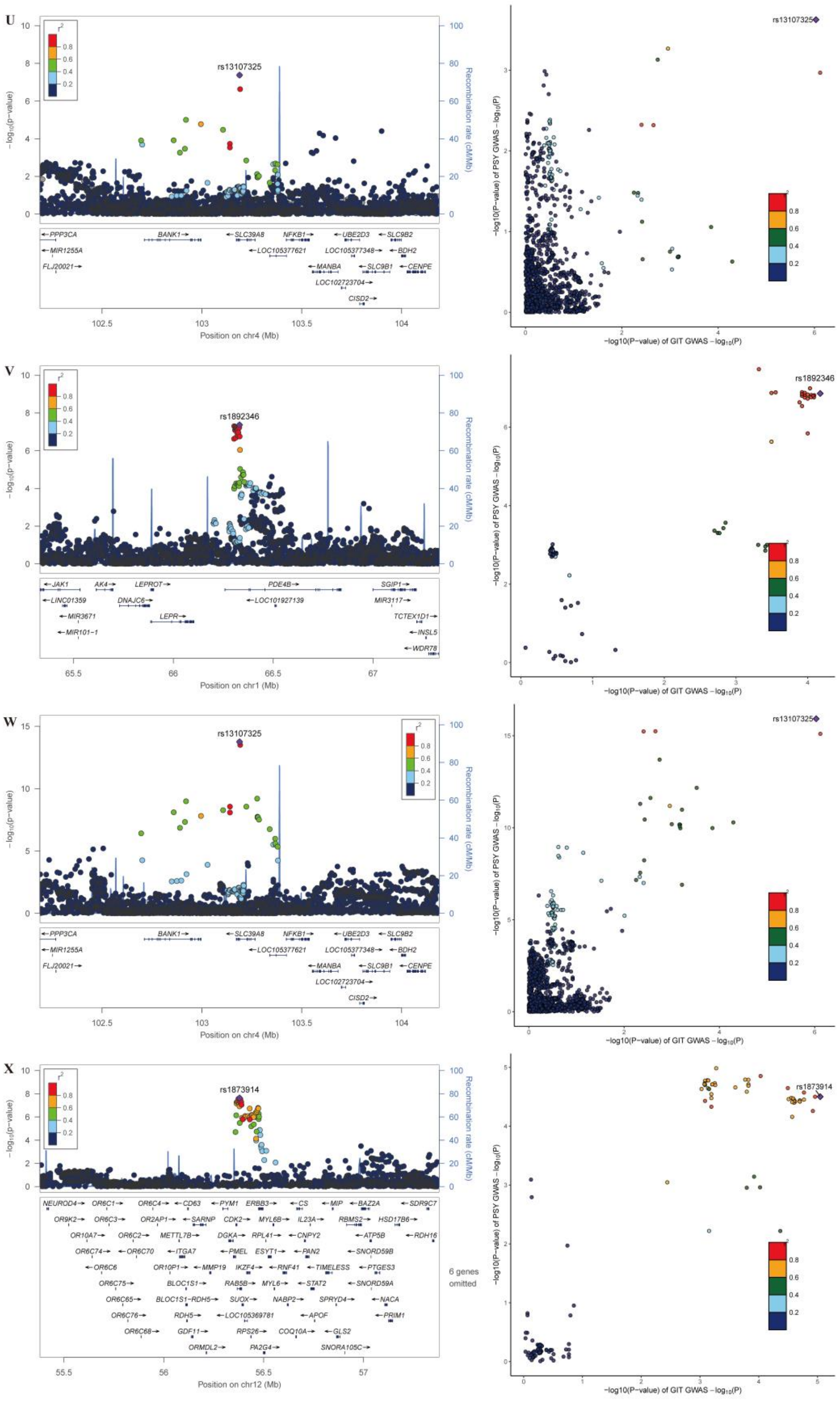

©2023 Gong W et al. *JAMA Psychiatry*.

For each colocalized locus (PP.H4 > 0.7) identified for corresponding trait pair, the left panel depicts the PLACO results using LocusZoom plot, and the right panel compares two single-trait GWAS statistics of corresponding trait pair for each variant using LocusCompare plot. For the LocusZoom plot, the x-axis shows the genomic position for each variant, and the y-axis shows −log10 *P* values from PLCAO results. The top variant with the smallest P_{PLACO} in each locus is indicated in purple diamond. The color of each variant represents its LD relationship with the top variant. For the LocusCompare plot, each dot represents a variant, the x-axis shows the −log10 P_{GWAS} from corresponding GWAS of gastrointestinal disease, and the y-axis shows −log10 P_{GWAS} from corresponding GWAS psychiatric disorder. The candidate shared causal variant identified by pairwise colocalization analysis is also indicated in purple diamond. The color of each variant represents its LD relationship with the candidate shared causal variant. All genomic location is based on reference genome hg19, and LD calculation is based on 1000 Genomes Project of European population. (A) 1q31.3 for IBD-MDD; (B) 5q33.3 for IBD-MDD; (C) 1q32.1 for IBD-SCZ; (D) 7q22.1 for IBD-SCZ; (E) 8q12.1 for IBD-BIP; (F) 10q21.2 for IBD-BIP; (G) 16p12.2 for IBD-BIP; (H) 5q14.3 for IBS-MDD; (I) 11q23.2 for IBS-MDD; (J) 16p12.2 for IBS-MDD; (K) 1p32.3 for IBS-SCZ; (L) 1q23.3 for IBS-SCZ; (M) 3q22.3 for IBS-SCZ; (N) 11q24.2 for IBS-SCZ; (O) 11q25 for IBS-SCZ; (P) 1p32.3 for IBS-BIP; (Q) 1q23.3 for IBS-BIP; (R) 7q21.11 for IBS-BIP; (S) 19q13.33 for PUD-SCZ; (T) 2q33.3 for GORD-MDD; (U) 4q24 for GORD-PTSD; (V) 1p31.3 for GORD-SCZ; (W) 4q24 for GORD-SCZ; (X) 12q13.2 for GORD-AN. Detailed descriptions were provided in Table 3.

©2023 Gong W et al. *JAMA Psychiatry*.

eFigure 4. Gene Expression Heatmap of 158 Significant Pleiotropic Genes in 25 Tissues

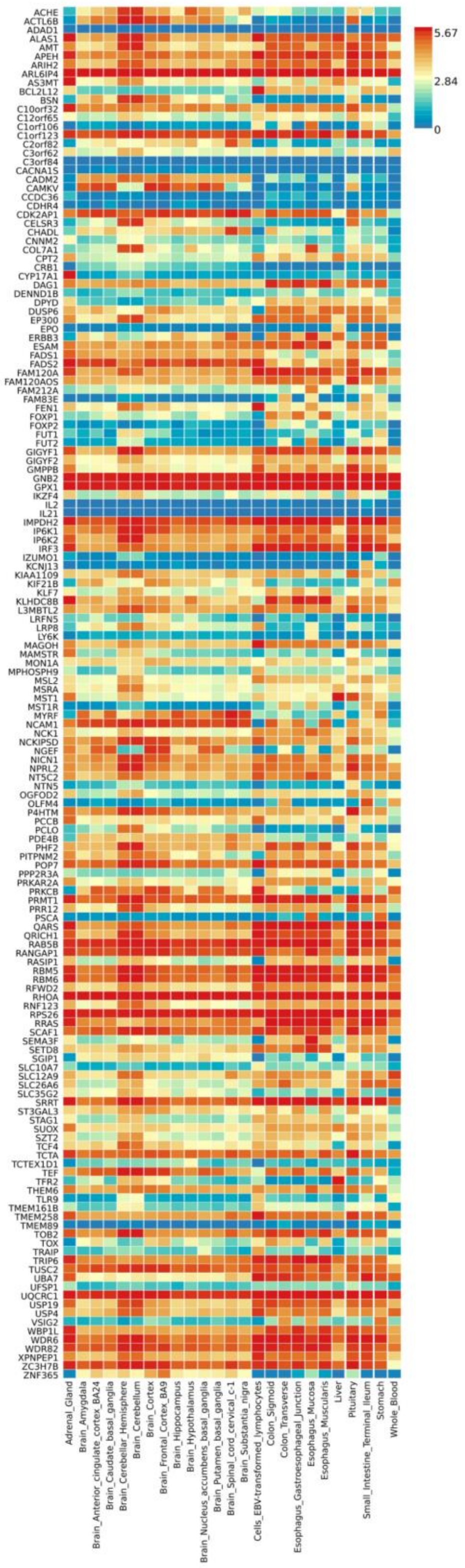

©2023 Gong W et al. *JAMA Psychiatry*.

The color represents log2 transformed average gene expression level.

©2023 Gong W et al. *JAMA Psychiatry*.

eFigure 5. The Bidirectional Causal Effects Estimated by IVW Method

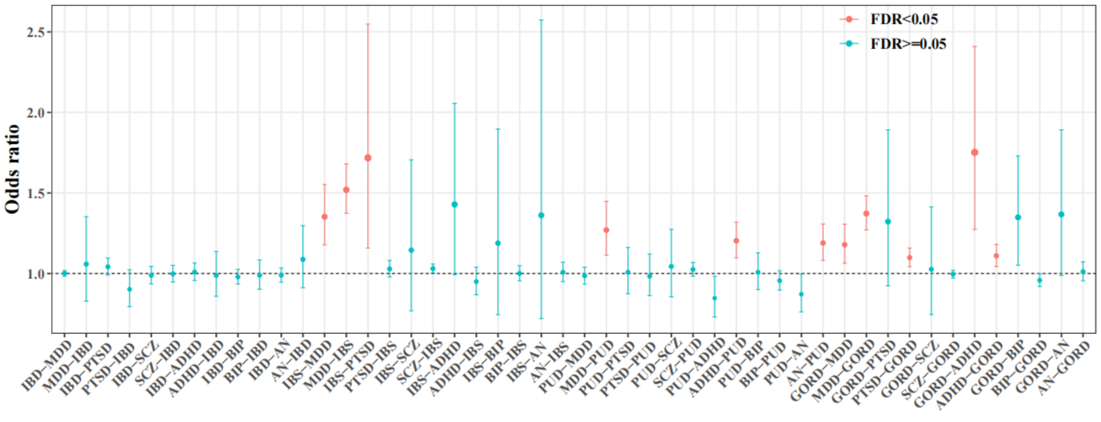

The dots represent point estimates of causal effects. Error bars represent the 95% CI of causal effects. FDR indicates false discovery rate; IBD, inflammatory bowel disease; IBS, irritable bowel syndrome; PUD, peptic ulcers disease; GORD, gastro-oesophageal reflux disease; MDD, major depressive disorder; PTSD, post-traumatic stress disorder; SCZ, schizophrenia; ADHD, attention deficit hyperactivity disorder; BIP, bipolar disorder; AN, anorexia nervosa.

©2023 Gong W et al. *JAMA Psychiatry*.

eTable 1. Details of GWAS Summary Data Sources

Diseases	Abbreviations	PMID	Year	N_cases	N_total	Ancestry	h^2 (se)[a]	Intercept (se)[a]
Inflammatory bowel disease	IBD	33608531	2021	7 045	456 327	EUR	0.0107 (0.0016)	1.0309 (0.0079)
Irritable bowel syndrome	IBS	34741163	2021	53 400	486 601	EUR	0.0246 (0.0015)	0.9974 (0.0069)
Peptic ulcer disease	PUD	33608531	2021	16 666	456 327	EUR	0.0105 (0.0013)	1.0139 (0.0072)
Gastro-oesophageal reflux disease	GORD	33608531	2021	54 854	456 327	EUR	0.0290 (0.0015)	1.0222 (0.0077)
Major depressive disorder	MDD	30718901	2019	170 756	500 199	EUR	0.0599 (0.0023)	1.0010 (0.0097)
Post-traumatic stress disorder	PTSD	31594949	2019	23 212	174 659	EUR	0.0414 (0.0075)	1.0219 (0.0065)
Schizophrenia	SCZ	29483656	2018	40 675	105 318	EUR	0.4101 (0.0139)	1.0698 (0.0114)
Attention deficit hyperactivity disorder	ADHD	30478444	2019	19 099	53 293	EUR	0.5534 (0.0354)	1.0328 (0.0098)
Bipolar disorder	BIP	34002096	2021	41 917	413 466	EUR	0.0708 (0.0027)	1.0246 (0.0088)
Anorexia nervosa	AN	31308545	2019	16 992	72 517	EUR	0.1772 (0.0120)	1.0270 (0.0101)
Early age-related macular degeneration	early AMD	32843070	2020	14 034	105 248	EUR	0.0353 (0.0119)	0.9949 (0.0079)
Cataract	CAT	31427789	2019	11 986	127 603	EUR	0.0188 (0.0038)	1.0088 (0.0063)

Abbreviations: PMID, PubMed unique identifier; EUR, European; h^2, heritability; se, standard error.

[a] The heritability and intercept were estimated by univariate LDSC using single-trait GWAS summary statistics.

©2023 Gong W et al. *JAMA Psychiatry*.

eTable 2. Genetic Correlations Between 4 Gastrointestinal Tract Diseases and 6 Psychiatric Disorders Estimated by HDL[a]

Trait pair	rg	se	P_{HDL}
IBD–MDD	0.1181	0.0218	$\mathbf{6.48{\times}10^{-8}}$
IBD–PTSD	0.1837	0.0704	$9.10{\times}10^{-3}$
IBD–SCZ	0.0445	0.0322	$1.67{\times}10^{-1}$
IBD–ADHD	0.0586	0.0399	$1.41{\times}10^{-1}$
IBD–BIP	0.0613	0.0309	$4.72{\times}10^{-2}$
IBD–AN	-0.0242	0.0460	$5.99{\times}10^{-1}$
IBS–MDD	0.6421	0.0346	$\mathbf{8.44{\times}10^{-77}}$
IBS–PTSD	0.5470	0.0812	$\mathbf{1.62{\times}10^{-11}}$
IBS–SCZ	0.1863	0.0275	$\mathbf{1.36{\times}10^{-11}}$
IBS–ADHD	0.2383	0.0251	$\mathbf{2.60{\times}10^{-21}}$
IBS–BIP	0.1963	0.0313	$\mathbf{3.66{\times}10^{-10}}$
IBS–AN	0.1416	0.0291	$\mathbf{1.17{\times}10^{-6}}$
PUD–MDD	0.4818	0.0422	$\mathbf{3.81{\times}10^{-30}}$
PUD–PTSD	0.5714	0.1010	$\mathbf{1.52{\times}10^{-8}}$
PUD–SCZ	0.1162	0.0283	$\mathbf{4.01{\times}10^{-5}}$
PUD–ADHD	0.4473	0.0455	$\mathbf{8.08{\times}10^{-23}}$
PUD–BIP	0.0996	0.0338	$3.24{\times}10^{-3}$
PUD–AN	0.0384	0.0468	$4.12{\times}10^{-1}$
GORD–MDD	0.4693	0.0258	$\mathbf{4.04{\times}10^{-74}}$
GORD–PTSD	0.4605	0.0553	$\mathbf{8.51{\times}10^{-17}}$
GORD–SCZ	0.0462	0.0185	$1.25{\times}10^{-2}$
GORD–ADHD	0.4137	0.0290	$\mathbf{4.59{\times}10^{-46}}$
GORD–BIP	0.0385	0.0224	$8.56{\times}10^{-2}$
GORD–AN	0.0424	0.0289	$1.43{\times}10^{-1}$

Abbreviations: HDL, high-definition likelihood method; rg, genetic correlation; se, standard error; IBD, inflammatory bowel disease; IBS, irritable bowel syndrome; PUD, peptic ulcers disease; GORD, gastro-oesophageal reflux disease; MDD, major depressive disorder; PTSD, post-traumatic stress disorder; SCZ, schizophrenia; ADHD, attention deficit hyperactivity disorder; BIP, bipolar disorder; AN, anorexia nervosa.

[a] HDL was used to estimate the genetic correlation between two traits. Significant results with *P* value reaching the Bonferroni corrected threshold ($P < 2.83{\times}10^{-3}$ [0.05/24]) are highlighted in bold.

©2023 Gong W et al. *JAMA Psychiatry*.

eTable 3. Bivariate LDSC Estimates in Negative Control Analysis[a]

Trait pair	rg (se)	P_{rg}	Intercept (se)	$P_{intercept}$
CAT–IBD	0.0629 (0.1097)	0.5663	0.0012 (0.0047)	0.7985
CAT–IBS	0.0948 (0.0756)	0.2104	–0.0045 (0.0050)	0.3681
CAT–PUD	0.0644 (0.1023)	0.5292	0.0004 (0.0049)	0.9349
CAT–GORD	0.1679 (0.0679)	0.0135	0.0077 (0.0047)	0.1014
CAT–MDD	0.0786 (0.0531)	0.1384	0.0093 (0.0052)	0.0737
CAT–PTSD	–0.0867 (0.1217)	0.4761	–0.0005 (0.0044)	0.9095
CAT–SCZ	–0.0067 (0.0490)	0.8907	–0.0117 (0.0059)	0.0474
CAT–ADHD	–0.0339 (0.0807)	0.6741	0.0065 (0.0058)	0.2624
CAT–BIP	0.0054 (0.0618)	0.9306	–0.0009 (0.0059)	0.8788
CAT–AN	0.0001 (0.0788)	0.9987	0.0023 (0.0058)	0.6917
early AMD–IBD	0.0686 (0.0884)	0.4375	–0.0018 (0.0049)	0.7134
early AMD–IBS	–0.0792 (0.0731)	0.2788	0.0030 (0.0056)	0.5922
early AMD–PUD	0.0325 (0.1003)	0.7461	–0.0033 (0.0052)	0.5257
early AMD–GORD	–0.0060 (0.0596)	0.9192	–0.0098 (0.0051)	0.0547
early AMD–MDD	–0.0592 (0.0448)	0.1857	0.0067 (0.0055)	0.2232
early AMD–PTSD	0.0598 (0.0975)	0.5395	–0.0024 (0.0046)	0.6019
early AMD–SCZ	–0.0069 (0.0471)	0.8827	–0.0036 (0.0061)	0.5551
early AMD–ADHD	–0.0784 (0.0591)	0.1845	–0.0004 (0.0051)	0.9375
early AMD–BIP	0.0454 (0.0431)	0.2916	–0.0063 (0.0053)	0.2346
early AMD–AN	0.0003 (0.0641)	0.9967	–0.0023 (0.0056)	0.6813

Abbreviations: LDSC, linkage disequilibrium score regression; rg, genetic correlation; se, standard error; CAT, cartaract; AMD, age-related macular degeneration; IBD, inflammatory bowel disease; IBS, irritable bowel syndrome; PUD, peptic ulcers disease; GORD, gastro-oesophageal reflux disease; MDD, major depressive disorder; PTSD, post-traumatic stress disorder; SCZ, schizophrenia; ADHD, attention deficit hyperactivity disorder; BIP, bipolar disorder; AN, anorexia nervosa.

[a] Bivariate LDSC was used to estimate the genetic correlation between negative controls (2 eye disorders) and a total of 10 traits (4 gastrointestinal tract diseases and 6 psychiatric disorders) with the intercept indicating potential sample overlap between two GWASs. Bonferroni corrected significant threshold was set at $P < 2.5\times10^{-3}$ (0.05/20). As expected, no significant genetic correlations were detected in this negative control analysis.

©2023 Gong W et al. *JAMA Psychiatry*.

eTable 4. Summary of Genome-Wide Significant Pleiotropic SNVs and FUMA-Annotated Pleiotropic Genomic Risk Loci for Each Pair of Traits

Trait pair	Total SNPs	No. SNP[b]	No. loci[c]	Chromosomal region
IBD–MDD	7 361 159	142	7	1p31.1, 1p31.3, 1q31.3, 1q32.1, 5q33.3, 9p23, 10q21.2
IBD–SCZ	7 078 011	113	6	1q23.3, 1q32.1, 4q24, 5q12.1, 7q22.1, 19q13.33
IBD–BIP	7 130 578	89	5	1q32.1, 2q31.3, 8q12.1, 10q21.2, 16p12.2
IBD–AN	6 839 114	388	2	1q32.1, 3p21.31
IBS–MDD	7 496 417	299	7	1q25.1, 5q14.3, 11q23.2, 13q14.3, 14q21.1, 16p12.2, 18q21.2
IBS–PTSD[a]	7 948 182	NA	NA	NA
IBS–SCZ	7 259 187	315	10	1p21.3, 1p32.3, 1q23.3, 3q22.3, 5q33.1, 9q22.31, 11q24.2, 11q25, 12q24.31, 22q13.2
IBS–ADHD	6 844 670	65	2	1p34.2, 5q14.3
IBS–BIP	7 259 266	197	9	1p32.3, 1q23.3, 3p12.1, 4q27, 7q21.11, 8p23.1, 10q25.1, 11q23.2, 13q14.3
IBS–AN	7 061 035	177	2	9q22.31, 11q23.2
PUD–MDD[a]	7 361 223	NA	NA	NA
PUD–PTSD[a]	7 754 811	NA	NA	NA
PUD–SCZ	7 078 062	192	4	1p21.3, 8q24.3, 11p14.3, 19q13.33
PUD–ADHD	6 845 733	43	3	1p34.1, 12q21.33, 19q13.33
PUD–BIP	7 130 644	47	4	3p22.2, 8p23.1, 8q24.3, 11q12.2
PUD–AN	6 839 179	53	2	4q31.22, 8q24.3
GORD–MDD	7 361 223	114	2	2q33.3, 11q23.2
GORD–PTSD	7 754 811	1	1	4q24
GORD–SCZ	7 078 062	424	9	1p21.3, 1p31.3, 2q33.1, 2q37.1, 4q24, 8q12.1, 8q24.3, 10q24.32, 12q24.11
GORD–ADHD	6 845 733	9	2	3p21.31, 7q31.1
GORD–BIP	7 130 644	13	2	3p21.2, 6q16.1
GORD–AN	6 839 179	229	4	3p21.31, 3p13, 11q23.2, 12q13.2
Total		2 910	83	

Abbreviations: IBD, inflammatory bowel disease; IBS, irritable bowel syndrome; PUD, peptic ulcers disease; GORD, gastro-oesophageal reflux disease; MDD, major depressive disorder; PTSD, post-traumatic stress disorder; SCZ, schizophrenia; ADHD, attention deficit hyperactivity disorder; BIP, bipolar disorder; AN, anorexia nervosa. NA, not applicable.

[a] No significant pleiotropic SNPs were identified, thus no pleiotropic loci were detected.

[b] Number of significant pleiotropic SNPs identified by PLACO.

[c] Number of pleiotropic genomic risk loci defined by FUMA.

eTable 5. 83 Pleiotropic Genomic Loci Identified by FUMA Using PLACO Results

No.	Trait pair	Top SNP	CHR	BP	Locus boundary	Region	P_{PLACO}	A1	A2	Nearest gene	Functional annotation	CADD	RDB	G-sig	P-sig
1	IBD–MDD	rs6690186	1	67003479	66991492–67250607	1p31.3	3.35×10^{-9}	T	C	*SGIP1*	intronic	0.855	5	1	1
2	IBD–MDD	rs7531118	1	72837239	72748669–72956535	1p31.1	2.86×10^{-10}	T	C	*RPL31P12*	intergenic	11.35	4	0	1
3	IBD–MDD	rs12118513	1	197517086	197342380–197781198	1q31.3	2.66×10^{-9}	T	A	*DENND1B*	intronic	9.82	6	0	1
4	IBD–MDD	rs169850	1	200929144	200874229–201014966	1q32.1	4.58×10^{-8}	T	C	*MROH3P*	ncRNA intronic	5.232	7	1	0
5	IBD–MDD	rs60689680	5	158834367	158827769–158856513	5q33.3	2.65×10^{-8}	G	T	*AC008703.1*	intergenic	1.096	7	0	0
6	IBD–MDD	rs10756219	9	11276005	11265159–11623147	9p23	1.60×10^{-8}	C	T	*RP11-23D5.1*	ncRNA intronic	1.807	6	0	1
7	IBD–MDD	rs10822050	10	64438771	64387108–64441247	10q21.2	9.11×10^{-9}	T	C	*ZNF365*	intergenic	0.457	7	1	0
8	IBD–SCZ	rs6658353	1	161469054	161463601–161479745	1q23.3	1.07×10^{-9}	G	C	*FCGR2A*	intergenic	0.756	5	1	0
9	IBD–SCZ	rs905634	1	200884985	200874229–201027055	1q32.1	1.89×10^{-12}	C	T	*INAVA*	downstream	2.128	NA	1	1
10	IBD–SCZ	rs6855246	4	103112470	102702364–103198082	4q24	6.88×10^{-10}	A	G	*SLC39A8*	intergenic	0.313	7	0	1
11	IBD–SCZ	rs7719676	5	60736949	60696323–60800336	5q12.1	9.93×10^{-9}	A	G	*ZSWIM6*	intronic	2.745	6	0	1
12	IBD–SCZ	rs492430	7	100313099	100219167–100523241	7q22.1	6.27×10^{-9}	T	G	*EPO*	intergenic	0.583	5	0	0
13	IBD–SCZ	rs2304204	19	50169020	50106208–50182697	19q13.33	3.24×10^{-8}	T	C	*IRF3:BCL2L12*	UTR5	11.47	NA	0	1
14	IBD–BIP	rs12132349	1	200875242	200870754–201024059	1q32.1	2.06×10^{-9}	T	A	*INAVA*	intronic	2.845	4	1	0
15	IBD–BIP	rs13029144	2	182334684	182308352–182334753	2q31.3	4.78×10^{-8}	C	T	*ITGA4*	intronic	4.22	7	1	0
16	IBD–BIP	rs56073120	8	59867177	59800835–59925249	8q12.1	1.76×10^{-8}	G	A	*TOX*	intronic	4.604	5	0	0
17	IBD–BIP	rs7090073	10	64423504	64387108–64441247	10q21.2	8.30×10^{-12}	G	T	*ZNF365*	intronic	8.613	5	1	1
18	IBD–BIP	rs196001	16	23962504	23892887–23962504	16p12.2	4.30×10^{-8}	G	A	*PRKCB*	intronic	2.377	4	0	0
19	IBD–AN	rs6427868	1	200929482	200864267–201024059	1q32.1	4.92×10^{-10}	A	G	*MROH3P*	ncRNA exonic	9.724	6	1	0
20	IBD–AN	rs11717978	3	48969036	48446237–50519141	3p21.31	1.24×10^{-13}	T	A	*ARIH2*	intronic	13.03	5	0	1
21	IBS–MDD	rs12755507	1	176164865	175902660–176406835	1q25.1	4.58×10^{-9}	T	C	*COP1*	intronic	6.038	4	0	1
22	IBS–MDD	rs3099439	5	87545318	87514778–87822672	5q14.3	1.77×10^{-9}	T	C	*TMEM161B*	intronic	1.562	NA	0	1

©2023 Gong W et al. *JAMA Psychiatry*.

No.	Trait pair	Top SNP	CHR	BP	Locus boundary	Region	P_{PLACO}	A1	A2	Nearest gene	Functional annotation	CADD	RDB	G-sig	P-sig
23	IBS–MDD	rs4937872	11	112827715	112826867–112912811	11q23.2	1.96×10^{-9}	A	G	*RP11-629G13.1*	intergenic	0.044	6	1	1
24	IBS–MDD	rs2806933	13	53643370	53617781–54049489	13q14.3	7.28×10^{-9}	A	C	*OLFM4*	intergenic	15.8	4	1	1
25	IBS–MDD	rs67505447	14	42143231	41969803–42183025	14q21.1	3.04×10^{-9}	A	G	*LRFN5*	intronic	2.016	6	0	1
26	IBS–MDD	rs1862743	16	60743834	60665658–60743834	16p12.2	4.45×10^{-8}	A	C	*GNPATP*	intergenic	1.06	6	0	0
27	IBS–MDD	rs12969536	18	53101860	53077795–53125364	18q21.2	1.44×10^{-8}	C	G	*TCF4*	intronic	0.054	7	0	1
28	IBS–SCZ	rs12031155	1	53714139	53658317–53752134	1p32.3	8.18×10^{-9}	T	C	*LRP8*	intronic	4.164	6	0	0
29	IBS–SCZ	rs1198572	1	98497176	98325796–98559093	1p21.3	3.04×10^{-10}	A	C	*MIR137HG*	ncRNA intronic	10.02	NA	0	1
30	IBS–SCZ	rs7542202	1	163763578	163616199–163766672	1q23.3	1.92×10^{-8}	T	C	*RP4-640E24.1*	intergenic	8.481	7	0	0
31	IBS–SCZ	rs1280622	3	136508008	135807609–136673157	3q22.3	1.56×10^{-10}	A	C	*RP11-731C17.1*	intergenic	3.161	NA	0	1
32	IBS–SCZ	rs12187419	5	151971392	151887779–152323236	5q33.1	8.19×10^{-9}	A	G	*AC091969.1*	intergenic	0.093	6	0	1
33	IBS–SCZ	rs12379660	9	96182487	96161300–96358301	9q22.31	3.13×10^{-8}	A	G	*Y_RNA*	intergenic	10.09	6	1	0
34	IBS–SCZ	rs11604175	11	124619407	124619407–124624854	11q24.2	1.12×10^{-8}	T	C	*VSIG2*	intronic	9.987	5	0	1
35	IBS–SCZ	rs12277680	11	134586708	134576216–134595774	11q25	4.68×10^{-8}	A	G	*RP11-469N6.2*	downstream	0.825	5	0	1
36	IBS–SCZ	rs2851443	12	123694250	123447928–123897177	12q24.31	3.60×10^{-9}	T	C	*MPHOSPH9*	intronic	1.395	NA	0	1
37	IBS–SCZ	rs20551	22	41548008	41408754–41854446	22q13.2	5.07×10^{-11}	A	G	*EP300*	exonic	12.01	5	0	1
38	IBS–ADHD	rs2782657	1	43929988	43788858–43949810	1p34.2	4.30×10^{-8}	C	G	*HYI-AS1*	intergenic	0.723	7	0	1
39	IBS–ADHD	rs6452785	5	87685500	87514778–87932809	5q14.3	8.60×10^{-11}	T	C	*TMEM161B-DT*	ncRNA intronic	14.18	5	0	1
40	IBS–BIP	rs5177	1	53711734	53658317–53752134	1p32.3	2.16×10^{-8}	C	G	*LRP8*	UTR3	6.557	1f	0	0
41	IBS–BIP	rs2345964	1	163750904	163582980–163768927	1q23.3	9.98×10^{-11}	A	G	*RP4-640E24.1*	intergenic	1.682	6	0	1
42	IBS–BIP	rs4301023	3	85057281	85002871–85671909	3p12.1	3.18×10^{-9}	T	C	*CADM2*	intronic	3.889	NA	1	0
43	IBS–BIP	rs77087420	4	123122856	123122856–123558330	4q27	3.45×10^{-9}	A	G	*BLTP1*	intronic	4.579	7	0	1
44	IBS–BIP	rs13239217	7	82538636	82387493–82583609	7q21.11	7.60×10^{-9}	A	G	*PCLO*	intronic	0.515	6	0	0
45	IBS–BIP	rs4840461	8	9904788	9881136–10006664	8p23.1	9.87×10^{-9}	T	C	*RP11-1E4.1*	intergenic	0.709	7	1	1

©2023 Gong W et al. *JAMA Psychiatry*.

No.	Trait pair	Top SNP	CHR	BP	Locus boundary	Region	P_{PLACO}	A1	A2	Nearest gene	Functional annotation	CADD	RDB	G-sig	P-sig
46	IBS–BIP	rs59042914	10	111734169	111648659–111928784	10q25.1	1.89×10^{-8}	A	G	*RP11-451M19.3*	intergenic	4.63	7	0	1
47	IBS–BIP	rs11214436	11	112830782	112826867–113034787	11q23.2	2.01×10^{-8}	T	G	*RP11-629G13.1*	ncRNA exonic	0.357	7	1	0
48	IBS–BIP	rs4886394	13	53907653	53879062–54054920	13q14.3	7.77×10^{-9}	A	C	*AL450423.1*	intergenic	0.793	6	1	0
49	IBS–AN	rs7021689	9	96229562	96163260–96356004	9q22.31	8.62×10^{-9}	T	C	*FAM120A*	intronic	1.456	4	1	0
50	IBS–AN	rs55694714	11	112829329	112826311–113062983	11q23.2	7.87×10^{-11}	A	T	*RP11-629G13.1*	downstream	3.516	5	1	0
51	PUD–SCZ	rs4322261	1	98405856	98327133–98559093	1p21.3	5.65×10^{-9}	G	A	*RP11-272L13.3*	ncRNA intronic	3.7	7	0	1
52	PUD–SCZ	rs2920281	8	143760444	143752994–143809193	8q24.3	3.69×10^{-8}	C	T	*JRK:PSCA*	ncRNA intronic	5.291	NA	1	1
53	PUD–SCZ	rs1808034	11	24367339	24367339–24412992	11p14.3	1.15×10^{-8}	A	G	*RP11-2F20.1*	intergenic	3.429	7	0	1
54	PUD–SCZ	rs681343	19	49206462	49103447–49254955	19q13.33	1.36×10^{-12}	C	T	*FUT2*	exonic	0.063	5	1	1
55	PUD–ADHD	rs3011217	1	44303266	44197228–44480093	1p34.1	3.59×10^{-8}	A	G	*ST3GAL3*	intronic	1.481	1f	0	0
56	PUD–ADHD	rs770082	12	89776485	89726027–89776845	12q21.33	1.74×10^{-8}	G	A	*RP11-1109F11.5*	intergenic	2.133	5	0	1
57	PUD–ADHD	rs601338	19	49206674	49168942–49250239	19q13.33	1.41×10^{-11}	G	A	*FUT2*	exonic	52	5	1	0
58	PUD–BIP	rs9834970	3	36856030	36834099–36870230	3p22.2	1.44×10^{-8}	T	C	*TRANK1*	intergenic	11.17	4	0	1
59	PUD–BIP	rs688245	8	9793646	9703795–9829379	8p23.1	1.86×10^{-8}	C	T	*snoU13*	intergenic	1.533	7	0	1
60	PUD–BIP	rs2717609	8	143769252	143752994–143780261	8q24.3	2.47×10^{-8}	A	T	*PSCA*	intergenic	0.997	6	1	1
61	PUD–BIP	rs102275	11	61557803	61542006–61624181	11q12.2	3.63×10^{-9}	T	C	*TMEM258*	intronic	8.908	7	0	1
62	PUD–AN	rs9784437	4	147216089	147216084–147337374	4q31.22	3.13×10^{-8}	A	G	*SLC10A7*	intronic	14.62	7	0	0
63	PUD–AN	rs2978977	8	143755720	143752994–143809193	8q24.3	7.18×10^{-10}	C	A	*JRK:PSCA*	ncRNA intronic	0.621	4	1	0
64	GORD–MDD	rs1263674	2	208055723	208017033–208088987	2q33.3	2.30×10^{-8}	T	C	*AC007879.1:AC007879.2*	ncRNA intronic	10.94	NA	0	1
65	GORD–MDD	rs3802850	11	112912518	112826867–112938783	11q23.2	8.19×10^{-9}	A	C	*NCAM1*	intronic	2.213	6	1	1
66	GORD–PTSD	rs13107325	4	103188709	102938709–103438709	4q24	4.19×10^{-8}	C	T	*SLC39A8*	exonic	34		0	0
67	GORD–SCZ	rs1892346	1	66331478	66304167–66333877	1p31.3	4.27×10^{-8}	T	A	*PDE4B*	intronic	2.812	7	0	1
68	GORD–SCZ	rs12073487	1	98315061	98298371–98559093	1p21.3	4.39×10^{-11}	T	A	*DPYD*	intronic	1.6	2b	0	1

©2023 Gong W et al. *JAMA Psychiatry*.

No.	Trait pair	Top SNP	CHR	BP	Locus boundary	Region	P_{PLACO}	A1	A2	Nearest gene	Functional annotation	CADD	RDB	G-sig	P-sig
69	GORD–SCZ	rs2084217	2	200127614	199908378–200131695	2q33.1	7.24×10^{-9}	G	C	*SATB2*	intergenic	11.77	5	0	1
70	GORD–SCZ	rs4973563	2	233716421	233559312–233806771	2q37.1	5.85×10^{-11}	C	T	*GIGYF2*	intronic	0.102	6	0	1
71	GORD–SCZ	rs13107325	4	103188709	102702364–103387161	4q24	1.78×10^{-14}	C	T	*SLC39A8*	exonic	34	5	0	1
72	GORD–SCZ	rs7836602	8	60675434	60485588–60954059	8q12.1	6.46×10^{-9}	C	G	*RP11-960H2.2*	intergenic	2.372	6	0	1
73	GORD–SCZ	rs4129585	8	143312933	143308772–143349510	8q24.3	4.70×10^{-9}	A	C	*TSNARE1*	intronic	6.646	NA	0	1
74	GORD–SCZ	rs11191424	10	104625886	104571436–104962011	10q24.32	9.86×10^{-12}	G	A	*BORCS7-ASMT*	intronic	4.392	7	0	1
75	GORD–SCZ	rs4766428	12	110723245	110473245–110973245	12q24.11	4.44×10^{-9}	C	T	*ATP2A2*	intronic	2.012	5	0	1
76	GORD–ADHD	rs7613360	3	49916710	49897830–50167424	3p21.31	3.87×10^{-8}	C	T	*ACTBP13*	intergenic	2.031	5	0	0
77	GORD–ADHD	rs1229758	7	114229139	114104389–114287116	7q31.1	3.43×10^{-8}	G	A	*FOXP2*	intronic	0.047	NA	0	1
78	GORD–BIP	rs352139	3	52258372	52217088–52467263	3p21.2	3.74×10^{-8}	T	C	*TLR9:TLR9*	intronic	4.804	NA	0	0
79	GORD–BIP	rs2388334	6	98591622	98547979–98591622	6q16.1	2.09×10^{-8}	A	G	*RP11-436D23.1*	ncRNA intronic	5.756	7	0	1
80	GORD–AN	rs199956414	3	50022089	49734229–50209053	3p21.31	2.95×10^{-8}	G	A	*RBM6*	intronic	1.093	5	0	1
81	GORD–AN	rs13097265	3	70943143	70795054–71018894	3p13	1.87×10^{-10}	G	A	*AC096971.1*	intergenic	0.03	7	0	1
82	GORD–AN	rs7105462	11	112912048	112826867–112922254	11q23.2	7.40×10^{-9}	G	A	*NCAM1*	intronic	1.862	7	1	0
83	GORD–AN	rs1873914	12	56379427	56368708–56478658	12q13.2	2.32×10^{-8}	G	C	*RAB5B*	UTR5	6.6	6	0	0

Abbreviations: No., the numerical order of pleiotropic genomic risk locus; CHR, chromosome; BP, base pair; CADD, Combined Annotation-Dependent depletion scores; RDB, RegulomeDB scores.

Pleiotropic loci were identified by FUMA using significant pleiotropic variants ($P_{PLACO} < 5\times10^{-8}$) and top variants were annotated. Top SNP is the genetic variant with the smallest *P* value in each pleiotropic locus. Locus boundary displays the region (start-end) of FUMA-annotated pleiotropic genomic locus. We also annotated the SNPs with $P < 5 \times 10^{-8}$ in each single-trait GWAS by FUMA for comparison. These results were shown in G-sig and P-sig columns, respectively. For a certain pairwise trait, 0 denotes that the FUMA-annotated genomic risk locus from single GWAS did not locate in the same chromosomal region as that annotated from PLACO results, and 1 denotes that the locus locates in the same chromosomal region as that annotated from PLACO results.

©2023 Gong W et al. *JAMA Psychiatry*.

eTable 6. Effect Sizes and *P* Values of Top SNPs in 83 Pleiotropic Loci From Original GWAS Summary Statistics

No.	Trait pair	Top SNP	Region	A1	A2	OR_{GIT}	OR_{PSY}	P_{GIT}	P_{PSY}
1	IBD–MDD	rs6690186	1p31.3	T	C	0.8917	0.9704	2.30×10^{-6}	2.24×10^{-6}
2	IBD–MDD	rs7531118	1p31.1	T	C	0.9492	0.9637	2.50×10^{-3}	1.59×10^{-17}
3	IBD–MDD	rs12118513	1q31.3	T	A	0.9249	1.0302	1.20×10^{-4}	1.70×10^{-8}
4	IBD–MDD	rs169850	1q32.1	T	C	0.8844	0.9865	4.20×10^{-11}	3.02×10^{-3}
5	IBD–MDD	rs60689680	5q33.3	G	T	0.9244	1.0194	8.90×10^{-6}	2.41×10^{-5}
6	IBD–MDD	rs10756219	9p23	C	T	0.9367	0.9758	2.10×10^{-4}	2.26×10^{-8}
7	IBD–MDD	rs10822050	10q21.2	T	C	0.9100	1.0163	4.40×10^{-8}	2.34×10^{-4}
8	IBD–SCZ	rs6658353	1q23.3	G	C	1.1245	0.9652	6.90×10^{-12}	2.26×10^{-4}
9	IBD–SCZ	rs905634	1q32.1	C	T	1.1449	1.0508	7.80×10^{-13}	2.45×10^{-6}
10	IBD–SCZ	rs6855246	4q24	A	G	0.8966	0.8535	6.70×10^{-4}	1.99×10^{-14}
11	IBD–SCZ	rs7719676	5q12.1	A	G	1.0622	0.9296	8.40×10^{-4}	2.02×10^{-11}
12	IBD–SCZ	rs492430	7q22.1	T	G	1.1036	1.0732	1.50×10^{-5}	9.16×10^{-8}
13	IBD–SCZ	rs2304204	19q13.33	T	C	0.9401	1.0792	1.50×10^{-3}	4.43×10^{-11}
14	IBD–BIP	rs12132349	1q32.1	T	A	1.1430	1.0344	4.10×10^{-12}	1.21×10^{-3}
15	IBD–BIP	rs13029144	2q31.3	C	T	1.0949	1.0347	1.70×10^{-7}	3.17×10^{-4}
16	IBD–BIP	rs56073120	8q12.1	G	A	0.9186	0.9597	1.60×10^{-6}	3.13×10^{-5}
17	IBD–BIP	rs7090073	10q21.2	G	T	0.9041	1.0495	7.10×10^{-9}	6.68×10^{-7}
18	IBD–BIP	rs196001	16p12.2	G	A	0.9294	0.9561	3.80×10^{-5}	4.71×10^{-6}
19	IBD–AN	rs6427868	1q32.1	A	G	1.1354	1.0505	2.50×10^{-11}	8.50×10^{-4}
20	IBD–AN	rs11717978	3p21.31	T	A	0.8916	0.8606	1.20×10^{-5}	8.66×10^{-13}
21	IBS–MDD	rs12755507	1q25.1	T	C	1.0325	1.0259	8.14×10^{-6}	8.80×10^{-9}
22	IBS–MDD	rs3099439	5q14.3	T	C	0.9672	0.9762	1.34×10^{-6}	2.78×10^{-8}
23	IBS–MDD	rs4937872	11q23.2	A	G	0.9620	0.9788	3.21×10^{-8}	1.28×10^{-6}
24	IBS–MDD	rs2806933	13q14.3	A	C	1.0328	1.0242	4.41×10^{-6}	7.33×10^{-8}
25	IBS–MDD	rs67505447	14q21.1	A	G	0.9689	0.9722	4.29×10^{-5}	8.21×10^{-11}
26	IBS–MDD	rs1862743	16p12.2	A	C	0.9698	0.9788	7.36×10^{-6}	6.86×10^{-7}
27	IBS–MDD	rs12969536	18q21.2	C	G	1.0347	1.0300	9.45×10^{-5}	3.27×10^{-10}
28	IBS–SCZ	rs12031155	1p32.3	T	C	0.9682	0.9527	2.63×10^{-6}	5.44×10^{-7}
29	IBS–SCZ	rs1198572	1p21.3	A	C	1.0260	0.8980	3.60×10^{-3}	2.52×10^{-18}
30	IBS–SCZ	rs7542202	1q23.3	T	C	1.0330	1.0476	3.27×10^{-6}	1.51×10^{-6}
31	IBS–SCZ	rs1280622	3q22.3	A	C	1.0327	1.0634	3.24×10^{-6}	6.44×10^{-10}
32	IBS–SCZ	rs12187419	5q33.1	A	G	0.9635	0.9384	3.03×10^{-5}	1.85×10^{-8}
33	IBS–SCZ	rs12379660	9q22.31	A	G	0.9608	0.9614	3.05×10^{-8}	9.25×10^{-5}
34	IBS–SCZ	rs11604175	11q24.2	T	C	0.9713	0.9334	2.14×10^{-4}	3.13×10^{-10}
35	IBS–SCZ	rs12277680	11q25	A	G	1.0287	0.9540	4.57×10^{-5}	1.51×10^{-6}
36	IBS–SCZ	rs2851443	12q24.31	T	C	1.0223	0.9167	5.11×10^{-3}	8.83×10^{-16}
37	IBS–SCZ	rs20551	22q13.2	A	G	0.9632	0.9381	6.16×10^{-7}	1.30×10^{-9}
38	IBS–ADHD	rs2782657	1p34.2	C	G	1.0204	0.9228	3.73×10^{-3}	6.86×10^{-9}
39	IBS–ADHD	rs6452785	5q14.3	T	C	1.0342	0.9377	9.24×10^{-7}	2.10×10^{-6}

©2023 Gong W et al. *JAMA Psychiatry*.

No.	Trait pair	Top SNP	Region	A1	A2	OR_{GIT}	OR_{PSY}	P_{GIT}	P_{PSY}
40	IBS–BIP	rs5177	1p32.3	C	G	0.9679	0.9602	2.19×10^{-6}	1.75×10^{-5}
41	IBS–BIP	rs2345964	1q23.3	A	G	1.0333	1.0557	2.56×10^{-6}	8.05×10^{-9}
42	IBS–BIP	rs4301023	3p12.1	T	C	0.9701	1.0454	9.12×10^{-6}	2.12×10^{-6}
43	IBS–BIP	rs77087420	4q27	A	G	1.0648	0.9013	2.97×10^{-5}	5.45×10^{-7}
44	IBS–BIP	rs13239217	7q21.11	A	G	0.9617	0.9398	1.64×10^{-5}	4.31×10^{-7}
45	IBS–BIP	rs4840461	8p23.1	T	C	1.0297	0.9405	3.39×10^{-4}	3.35×10^{-8}
46	IBS–BIP	rs59042914	10q25.1	A	G	1.0280	0.9210	3.52×10^{-3}	1.38×10^{-10}
47	IBS–BIP	rs11214436	11q23.2	T	G	1.0381	1.0376	1.12×10^{-7}	1.30×10^{-4}
48	IBS–BIP	rs4886394	13q14.3	A	C	0.9710	0.9540	1.66×10^{-5}	5.06×10^{-7}
49	IBS–AN	rs7021689	9q22.31	T	C	1.0366	0.9516	1.59×10^{-7}	3.19×10^{-4}
50	IBS–AN	rs55694714	11q23.2	A	T	1.0492	1.1187	1.91×10^{-6}	1.29×10^{-7}
51	PUD–SCZ	rs4322261	1p21.3	G	A	1.0444	0.9043	3.40×10^{-3}	1.62×10^{-14}
52	PUD–SCZ	rs2920281	8q24.3	C	T	1.0905	0.9748	2.10×10^{-14}	8.70×10^{-3}
53	PUD–SCZ	rs1808034	11p14.3	A	G	0.9541	0.9491	2.80×10^{-5}	5.53×10^{-8}
54	PUD–SCZ	rs681343	19q13.33	C	T	0.9151	0.9570	1.90×10^{-15}	1.10×10^{-5}
55	PUD–ADHD	rs3011217	1p34.1	A	G	1.0423	1.0889	8.30×10^{-4}	1.51×10^{-8}
56	PUD–ADHD	rs770082	12q21.33	G	A	0.9622	0.9239	6.50×10^{-4}	8.25×10^{-9}
57	PUD–ADHD	rs601338	19q13.33	G	A	0.9153	1.0426	2.40×10^{-15}	2.52×10^{-3}
58	PUD–BIP	rs9834970	3p22.2	T	C	1.0248	0.9203	2.70×10^{-2}	6.63×10^{-19}
59	PUD–BIP	rs688245	8p23.1	C	T	1.0534	1.0624	1.50×10^{-4}	1.33×10^{-7}
60	PUD–BIP	rs2717609	8q24.3	A	T	1.0835	0.9728	2.10×10^{-12}	6.17×10^{-3}
61	PUD–BIP	rs102275	11q12.2	T	C	0.9652	0.9326	2.30×10^{-3}	6.20×10^{-13}
62	PUD–AN	rs9784437	4q31.22	A	G	0.9521	0.9200	3.10×10^{-4}	8.01×10^{-7}
63	PUD–AN	rs2978977	8q24.3	C	A	1.0886	0.9603	8.80×10^{-14}	3.72×10^{-3}
64	GORD–MDD	rs1263674	2q33.3	T	C	0.9723	0.9743	4.40×10^{-5}	1.87×10^{-8}
65	GORD–MDD	rs3802850	11q23.2	A	C	0.9667	0.9795	2.40×10^{-7}	2.56×10^{-6}
66	GORD–PTSD	rs13107325	4q24	C	T	0.9430	0.9029	9.30×10^{-7}	2.37×10^{-4}
67	GORD–SCZ	rs1892346	1p31.3	T	A	0.9743	0.9503	6.70×10^{-5}	1.39×10^{-7}
68	GORD–SCZ	rs12073487	1p21.3	T	A	0.9677	1.0813	1.80×10^{-5}	1.41×10^{-11}
69	GORD–SCZ	rs2084217	2q33.1	G	C	0.9773	0.9392	3.40×10^{-4}	6.67×10^{-11}
70	GORD–SCZ	rs4973563	2q37.1	C	T	1.0249	0.9249	2.60×10^{-4}	6.00×10^{-15}
71	GORD–SCZ	rs13107325	4q24	C	T	0.9430	0.8525	9.30×10^{-7}	1.19×10^{-16}
72	GORD–SCZ	rs7836602	8q12.1	C	G	0.9760	1.0589	1.80×10^{-4}	1.77×10^{-9}
73	GORD–SCZ	rs4129585	8q24.3	A	C	1.0183	1.0855	4.80×10^{-3}	9.26×10^{-18}
74	GORD–SCZ	rs11191424	10q24.32	G	A	1.0275	1.0845	7.20×10^{-5}	4.78×10^{-16}
75	GORD–SCZ	rs4766428	12q24.11	C	T	1.0198	0.9267	2.60×10^{-3}	2.68×10^{-14}
76	GORD–ADHD	rs7613360	3p21.31	C	T	0.9692	0.9437	1.60×10^{-6}	3.71×10^{-5}
77	GORD–ADHD	rs1229758	7q31.1	G	A	0.9754	0.9310	1.20×10^{-4}	2.29×10^{-7}
78	GORD–BIP	rs352139	3p21.2	T	C	1.0213	1.0575	1.10×10^{-3}	2.67×10^{-9}
79	GORD–BIP	rs2388334	6q16.1	A	G	1.0157	0.9286	1.50×10^{-2}	1.79×10^{-15}
80	GORD–AN	rs199956414	3p21.31	G	A	0.9727	1.0596	1.90×10^{-5}	2.07×10^{-5}

©2023 Gong W et al. *JAMA Psychiatry*.

No.	Trait pair	Top SNP	Region	A1	A2	OR_{GIT}	OR_{PSY}	P_{GIT}	P_{PSY}
81	GORD–AN	rs13097265	3p13	G	A	0.9635	0.9352	1.30×10^{-7}	6.05×10^{-6}
82	GORD–AN	rs7105462	11q23.2	G	A	1.0320	1.0583	1.40×10^{-6}	3.82×10^{-5}
83	GORD–AN	rs1873914	12q13.2	G	C	1.0292	0.9438	9.00×10^{-6}	3.16×10^{-5}

Abbreviations: No., the numerical order of pleiotropic genomic risk locus; A1, effect allele; A2, non-effect allele; OR, odds ratio. OR and *P* value were obtained from single-trait GWAS of corresponding pairwise traits.

©2023 Gong W et al. *JAMA Psychiatry*.

eTable 7. The eQTL Regulatory Information of rs601338 and rs681343 on *FUT2* Gene in Gastrointestinal Tract and Brain Tissues[a]

SNP	CHR		BP	Gene Symbol	A1	A2	Tissue	NES	*P* value
rs601338	19		49206674	*FUT2*	A	G	Esophagus - Mucosa	–0.73	1.80×10^{-159}
rs601338	19		49206674	*FUT2*	A	G	Stomach	–0.29	2.00×10^{-26}
rs601338	19		49206674	*FUT2*	A	G	Colon - Transverse	–0.25	1.80×10^{-24}
rs601338	19		49206674	*FUT2*	A	G	Small Intestine - Terminal Ileum	–0.34	2.50×10^{-17}
rs601338	19		49206674	*FUT2*	A	G	Brain - Caudate (basal ganglia)	0.41	3.60×10^{-11}
rs601338	19		49206674	*FUT2*	A	G	Brain - Nucleus accumbens (basal ganglia)	0.37	2.40×10^{-8}
rs601338	19		49206674	*FUT2*	A	G	Brain - Putamen (basal ganglia)	0.47	2.90×10^{-8}
rs601338	19		49206674	*FUT2*	A	G	Brain - Anterior cingulate cortex (BA24)	0.35	7.80×10^{-6}
rs681343	19		49206462	*FUT2*	T	C	Esophagus - Mucosa	–0.73	1.80×10^{-159}
rs681343	19		49206462	*FUT2*	T	C	Stomach	–0.29	2.00×10^{-26}
rs681343	19		49206462	*FUT2*	T	C	Colon - Transverse	–0.25	1.80×10^{-24}
rs681343	19		49206462	*FUT2*	T	C	Small Intestine - Terminal Ileum	–0.34	2.50×10^{-17}
rs681343	19		49206462	*FUT2*	T	C	Brain - Caudate (basal ganglia)	0.41	3.60×10^{-11}
rs681343	19		49206462	*FUT2*	T	C	Brain - Nucleus accumbens (basal ganglia)	0.37	2.40×10^{-8}
rs681343	19		49206462	*FUT2*	T	C	Brain - Putamen (basal ganglia)	0.47	2.90×10^{-8}
rs681343	19		49206462	*FUT2*	T	C	Brain - Anterior cingulate cortex (BA24)	0.35	7.80×10^{-6}

Abbreviations: eQTL, expression quantitative trait locus; SNP, single nucleotide polymorphisms; CHR, chromosome; BP, base pair; A1, effect allele; A2, non-effect allele; NES, normalized effect size.

[a] The single-trait eQTL information in gastrointestinal and brain tissues was obtained from GTEx portal.

©2023 Gong W et al. *JAMA Psychiatry*.

eTable 8. The Remaining 59 Loci in Colocalization Analysis

No.[a]	Trait pair	Top SNP	Locus boundary	Region	Nearest gene	PP.H3	PP.H4	Best causal SNP	SNP.PP.H4
1	IBD–MDD	rs6690186	1:66991492–67250607	1p31.3	*SGIP1*	0.6679	0.2650	rs6690186	0.0350
2	IBD–MDD	rs7531118	1:72748669–72956535	1p31.1	*RPL31P12*	0.0360	0.1050	rs7531118	0.2361
4	IBD–MDD	rs169850	1:200874229–201014966	1q32.1	*MROH3P*	0.0059	0.0213	rs905634	0.2788
6	IBD–MDD	rs10756219	9:11265159–11623147	9p23	*RP11-23D5.1*	0.1731	0.3177	rs10756219	0.0296
7	IBD–MDD	rs10822050	10:64387108–64441247	10q21.2	*ZNF365*	0.0109	0.1406	rs10995271	0.1295
8	IBD–SCZ	rs6658353	1:161463601–161479745	1q23.3	*FCGR2A*	0.0098	0.6234	rs4657041	0.3199
10	IBD–SCZ	rs6855246	4:102702364–103198082	4q24	*SLC39A8*	0.0287	0.5346	rs13107325	0.6173
11	IBD–SCZ	rs7719676	5:60696323–60800336	5q12.1	*ZSWIM6*	0.0124	0.0506	rs7709645	0.3056
13	IBD–SCZ	rs2304204	19:50106208–50182697	19q13.33	*IRF3:BCL2L12*	0.0233	0.5066	rs2304204	0.2422
14	IBD–BIP	rs12132349	1:200870754–201024059	1q32.1	*INAVA*	0.0274	0.3815	rs905634	0.1807
15	IBD–BIP	rs13029144	2:182308352–182334753	2q31.3	*ITGA4*	0.0085	0.2604	rs13029144	0.3863
19	IBD–AN	rs6427868	1:200864267–201024059	1q32.1	*MROH3P*	0.0395	0.2753	rs905634	0.2818
20	IBD–AN	rs11717978	3:48446237–50519141	3p21.31	*ARIH2*	0.9910	0.0076	rs34326553	0.0274
21	IBS–MDD	rs12755507	1:175902660–176406835	1q25.1	*COP1*	0.6093	0.3822	rs12755507	0.0698
24	IBS–MDD	rs2806933	13:53617781–54049489	13q14.3	*OLFM4*	0.7899	0.1960	rs2806933	0.2541
25	IBS–MDD	rs67505447	14:41969803–42183025	14q21.1	*LRFN5*	0.0491	0.5501	rs4356390	0.1655
27	IBS–MDD	rs12969536	18:53077795–53125364	18q21.2	*TCF4*	0.0187	0.1201	rs12967143	0.7954
29	IBS–SCZ	rs1198572	1:98325796–98559093	1p21.3	*MIR137HG*	0.0251	0.1920	rs2660304	0.1974
32	IBS–SCZ	rs12187419	5:151887779–152323236	5q33.1	*AC091969.1*	0.1200	0.1281	rs111294930	0.0587
33	IBS–SCZ	rs12379660	9:96161300–96358301	9q22.31	*Y_RNA*	0.3514	0.4968	rs10992729	0.0346
36	IBS–SCZ	rs2851443	12:123447928–123897177	12q24.31	*MPHOSPH9*	0.0505	0.1325	rs2851447	0.1657
37	IBS–SCZ	rs20551	22:41408754–41854446	22q13.2	*EP300*	0.5406	0.4320	rs9607782	0.7557

©2023 Gong W et al. *JAMA Psychiatry*.

No.[a]	Trait pair	Top SNP	Locus boundary	Region	Nearest gene	PP.H3	PP.H4	Best causal SNP	SNP.PP.H4
38	IBS–ADHD	rs2782657	1:43788858–43949810	1p34.2	*HYI-AS1*	0.0078	0.0817	rs2842198	0.2720
39	IBS–ADHD	rs6452785	5:87514778–87932809	5q14.3	*TMEM161B-DT*	0.5525	0.4175	rs4916723	0.0499
42	IBS–BIP	rs4301023	3:85002871–85671909	3p12.1	*CADM2*	0.8914	0.0371	rs1248860	0.0618
43	IBS–BIP	rs77087420	4:123122856–123558330	4q27	*BLTP1*	0.1775	0.3192	rs77087420	0.3700
45	IBS–BIP	rs4840461	8:9881136–10006664	8p23.1	*RP11-1E4.1*	0.0299	0.0534	rs17150993	0.3685
46	IBS–BIP	rs59042914	10:111648659–111928784	10q25.1	*RP11-451M19.3*	0.0249	0.1361	rs17095350	0.0395
47	IBS–BIP	rs11214436	11:112826867–113034787	11q23.2	*RP11-629G13.1*	0.8294	0.1279	rs10891481	0.0274
48	IBS–BIP	rs4886394	13:53879062–54054920	13q14.3	*AL450423.1*	0.7945	0.1811	rs4886394	0.0505
49	IBS–AN	rs7021689	9:96163260–96356004	9q22.31	*FAM120A*	0.1269	0.1789	rs12156439	0.0476
50	IBS–AN	rs55694714	11:112826311–113062983	11q23.2	*RP11-629G13.1*	0.5932	0.4026	rs55694714	0.0632
51	PUD–SCZ	rs4322261	1:98327133–98559093	1p21.3	*RP11-272L13.3*	0.0411	0.0269	rs1198572	0.1489
52	PUD–SCZ	rs2920281	8:143752994–143809193	8q24.3	*JRK:PSCA*	0.2698	0.0743	rs2976388	0.2106
53	PUD–SCZ	rs1808034	11:24367339–24412992	11p14.3	*RP11-2F20.1*	0.0826	0.6002	rs11027827	0.0314
55	PUD–ADHD	rs3011217	1:44197228–44480093	1p34.1	*ST3GAL3*	0.0836	0.0141	rs12354267	0.1463
56	PUD–ADHD	rs770082	12:89726027–89776845	12q21.33	*RP11-1109F11.5*	0.0324	0.2511	rs770082	0.1676
57	PUD–ADHD	rs601338	19:49168942–49250239	19q13.33	*FUT2*	0.0120	0.3552	rs681343	0.2729
58	PUD–BIP	rs9834970	3:36834099–36870230	3p22.2	*TRANK1*	0.0016	0.0587	rs9834970	0.9907
59	PUD–BIP	rs688245	8:9703795–9829379	8p23.1	*snoU13*	0.0774	0.1117	rs62489493	0.4237
60	PUD–BIP	rs2717609	8:143752994–143780261	8q24.3	*PSCA*	0.0381	0.0484	rs2920281	0.2542
61	PUD–BIP	rs102275	11:61542006–61624181	11q12.2	*TMEM258*	0.0181	0.2625	rs174592	0.1803
62	PUD–AN	rs9784437	4:147216084–147337374	4q31.22	*SLC10A7*	0.0279	0.6463	rs9784437	0.1152
63	PUD–AN	rs2978977	8:143752994–143809193	8q24.3	*JRK:PSCA*	0.0132	0.1853	rs2920281	0.1855
65	GORD–MDD	rs3802850	11:112826867–112938783	11q23.2	*NCAM1*	0.3269	0.6307	rs3802850	0.0343

©2023 Gong W et al. *JAMA Psychiatry*.

No.[a]	Trait pair	Top SNP	Locus boundary	Region	Nearest gene	PP.H3	PP.H4	Best causal SNP	SNP.PP.H4
68	GORD–SCZ	rs12073487	1:98298371–98559093	1p21.3	*DPYD*	0.4509	0.0596	rs2660304	0.1884
69	GORD–SCZ	rs2084217	2:199908378–200131695	2q33.1	*SATB2*	0.0597	0.3353	rs260755	0.0480
70	GORD–SCZ	rs4973563	2:233559312–233806771	2q37.1	*GIGYF2*	0.0527	0.4745	rs4144797	0.1680
72	GORD–SCZ	rs7836602	8:60485588–60954059	8q12.1	*RP11-960H2.2*	0.2058	0.2623	rs1473594	0.0636
73	GORD–SCZ	rs4129585	8:143308772–143349510	8q24.3	*TSNARE1*	0.0036	0.1122	rs4129585	0.6357
74	GORD–SCZ	rs11191424	10:104571436–104962011	10q24.32	*BORCS7-ASMT*	0.2439	0.0648	rs11191419	0.0704
75	GORD–SCZ	rs4766428	12:110473245–110973245	12q24.11	*ATP2A2*	0.0127	0.2274	rs4766428	0.9998
76	GORD–ADHD	rs7613360	3:49897830–50167424	3p21.31	*ACTL11P*	0.6050	0.1486	rs7613360	0.8207
77	GORD–ADHD	rs1229758	7:114104389–114287116	7q31.1	*FOXP2*	0.0186	0.6227	rs9969232	0.1768
78	GORD–BIP	rs352139	3:52217088–52467263	3p21.2	*TLR9:TLR9*	0.0112	0.1432	rs7622851	0.1639
79	GORD–BIP	rs2388334	6:98547979–98591622	6q16.1	*RP11-436D23.1*	0.0023	0.0534	rs9372734	0.1742
80	GORD–AN	rs199956414	3:49734229–50209053	3p21.31	*RBM6*	0.9655	0.0002	rs73077200	0.2805
81	GORD–AN	rs13097265	3:70795054–71018894	3p13	*AC096971.1*	0.5159	0.4686	rs13097265	0.1535
82	GORD–AN	rs7105462	11:112826867–112922254	11q23.2	*NCAM1*	0.9851	0.0076	rs2186874	0.0233

Abbreviations: No., the numerical order of pleiotropic genomic risk locus; PP.H3, posterior probability of the model H3, indicating that both traits are associated with the locus but with different causal variants; PP.H4, posterior probability of the model H4, indicating that both traits are associated with the locus and share a single causal variant; SNP.PP.H4, posterior probability of the best causal variant conditioned on H4 being true.

[a] The details of the these pleiotropic genomic risk loci were provided in eTable 7.

©2023 Gong W et al. *JAMA Psychiatry*.

eTable 9. Candidate Pleiotropic Genes Identified by MAGMA

Trait pairs	No loci	locus	Locus position	Gene symbol	EntrezID	Gene position	P_{PLACO}	Sig$_{PLACO}$	P_{GIT}	P_{PSY}
IBD–MDD	1	1p31.3	66991492-67250607	*SGIP1*	84251	66998066–67211768	$1.26×10^{-9}$	TRUE	$7.96×10^{-5}$	$5.02×10^{-11}$
IBD–MDD	1	1p31.3	66991492-67250607	*DYNLT5*	200132	67216978–67245730	$4.06×10^{-6}$	TRUE	$2.53×10^{-3}$	$6.98×10^{-7}$
IBD–MDD	2	1p31.1	72748669-72956535	*NEGR1*	257194	71867625–72749533	$3.27×10^{-2}$	TRUE	$7.67×10^{-1}$	$1.20×10^{-10}$
IBD–MDD	3	1q31.3	197342380-197781198	*DENND1B*	163486	197472878–197745623	$1.47×10^{-3}$	TRUE	$3.19×10^{-2}$	$2.05×10^{-4}$
IBD–MDD	3	1q31.3	197342380-197781198	*CRB1*	23418	197169592–197448585	$4.45×10^{-3}$	TRUE	$2.98×10^{-2}$	$5.36×10^{-4}$
IBD–MDD	4	1q32.1	200874229-201014966	*KIF21B*	23046	200937514–200993828	$2.27×10^{-5}$	TRUE	$2.48×10^{-8}$	$6.08×10^{-2}$
IBD–MDD	4	1q32.1	200874229-201014966	*INAVA*	55765	200859627–200885864	$5.74×10^{-5}$	TRUE	$3.71×10^{-13}$	$1.14×10^{-1}$
IBD–MDD	4	1q32.1	200874229-201014966	*CACNA1S*	779	201007635–201082694	$1.81×10^{-4}$	TRUE	$5.28×10^{-6}$	$6.31×10^{-3}$
IBD–MDD	7	10q21.2	64387108-64441247	*ZNF365*	22891	64132916–64432771	$9.97×10^{-5}$	TRUE	$1.23×10^{-9}$	$7.05×10^{-2}$
IBD–SCZ	8	1q23.3	161463601-161479745	*FCGR2A*	2212	161474205–161490360	$1.08×10^{-3}$	TRUE	$9.88×10^{-7}$	$5.47×10^{-2}$
IBD–SCZ	9	1q32.1	200874229-201027055	*INAVA*	55765	200859627–200885864	$1.35×10^{-10}$	TRUE	$3.71×10–^{13}$	$3.57×10^{-5}$
IBD–SCZ	9	1q32.1	200874229-201027055	*KIF21B*	23046	200937514–200993828	$4.94×10^{-8}$	TRUE	$2.48×10^{-8}$	$1.02×10^{-4}$
IBD–SCZ	9	1q32.1	200874229-201027055	*CACNA1S*	779	201007635–201082694	$7.12×10^{-6}$	TRUE	$5.28×10^{-6}$	$5.40×10^{-4}$
IBD–SCZ	10	4q24	102702364-103198082	*SLC39A8*	64116	103171198–103267655	$9.72×10^{-3}$	TRUE	$8.91×10^{-2}$	$6.30×10^{-6}$
IBD–SCZ	10	4q24	102702364-103198082	*BANK1*	55024	102710764–102996969	$1.64×10^{-2}$	TRUE	$1.74×10^{-1}$	$1.36×10^{-4}$
IBD–SCZ	11	5q12.1	60696323-60800336	*ZSWIM6*	57688	60627100–60842999	$1.70×10^{-4}$	TRUE	$9.45×10^{-2}$	$3.64×10^{-10}$
IBD–SCZ	12	7q22.1	100219167-100523241	*UFSP1*	402682	100485344–100488339	$3.03×10^{-8}$	TRUE	$3.74×10^{-6}$	$7.65×10^{-6}$
IBD–SCZ	12	7q22.1	100219167-100523241	*GIGYF1*	64599	100276130–100287870	$7.08×10^{-8}$	TRUE	$1.33×10^{-5}$	$5.28×10^{-7}$
IBD–SCZ	12	7q22.1	100219167-100523241	*ACHE*	43	100486615–100494754	$1.68×10^{-7}$	TRUE	$4.86×10^{-6}$	$1.88×10^{-5}$
IBD–SCZ	12	7q22.1	100219167-100523241	*GNB2*	2783	100270363–100277792	$9.36×10^{-7}$	TRUE	$2.87×10^{-5}$	$3.10×10^{-5}$
IBD–SCZ	12	7q22.1	100219167-100523241	*TFR2*	7036	100217039–100241332	$9.88×10^{-7}$	TRUE	$7.83×10^{-5}$	$2.45×10^{-5}$
IBD–SCZ	12	7q22.1	100219167-100523241	*SLC12A9*	56996	100449337–100465634	$1.06×10^{-6}$	TRUE	$1.24×10^{-5}$	$6.66×10^{-5}$
IBD–SCZ	12	7q22.1	100219167-100523241	*TRIP6*	7205	100463950–100472076	$1.27×10^{-6}$	TRUE	$2.09×10^{-5}$	$3.36×10^{-5}$
IBD–SCZ	12	7q22.1	100219167-100523241	*SRRT*	51593	100471701–100487285	$6.43×10^{-6}$	TRUE	$6.33×10^{-5}$	$1.35×10^{-4}$
IBD–SCZ	12	7q22.1	100219167-100523241	*EPO*	2056	100317423–100322323	$2.25×10^{-5}$	TRUE	$7.03×10^{-5}$	$1.86×10^{-4}$

©2023 Gong W et al. *JAMA Psychiatry*.

IBD–SCZ	12	7q22.1	100219167-100523241	*ACTL6B*	51412	100239720–100255084	2.70×10^{-4}	TRUE	2.46×10^{-3}	1.25×10^{-3}
IBD–SCZ	12	7q22.1	100219167-100523241	*POP7*	10248	100302676–100306123	9.26×10^{-4}	TRUE	3.73×10^{-4}	2.75×10^{-2}
IBD–SCZ	12	7q22.1	100219167-100523241	*ZAN*	7455	100330249–100396419	9.16×10^{-3}	FALSE	3.67×10^{-3}	4.79×10^{-2}
IBD–SCZ	12	7q22.1	100219167-100523241	*EPHB4*	2050	100399187–100426143	1.14×10^{-2}	FALSE	1.21×10^{-2}	1.42×10^{-2}
IBD–SCZ	13	19q13.33	50106208-50182697	*BCL2L12*	83596	50167399–50178173	4.15×10^{-8}	TRUE	1.55×10^{-3}	5.96×10^{-11}
IBD–SCZ	13	19q13.33	50106208-50182697	*IRF3*	3661	50161826–50170132	1.09×10^{-7}	TRUE	2.28×10^{-3}	1.96×10^{-10}
IBD–SCZ	13	19q13.33	50106208-50182697	*SCAF1*	58506	50144382–50162906	1.21×10^{-7}	TRUE	2.24×10^{-3}	1.29×10^{-10}
IBD–SCZ	13	19q13.33	50106208-50182697	*PRMT1*	3276	50179409–50192707	8.29×10^{-7}	TRUE	2.83×10^{-3}	2.76×10^{-9}
IBD–SCZ	13	19q13.33	50106208-50182697	*PRR12*	57479	50093912–50130696	2.57×10^{-5}	TRUE	1.48×10^{-2}	6.42×10^{-7}
IBD–SCZ	13	19q13.33	50106208-50182697	*RRAS*	6237	50137552–50144400	4.93×10^{-4}	TRUE	3.88×10^{-2}	4.57×10^{-6}
IBD–BIP	14	1q32.1	200870754-201024059	*INAVA*	55765	200859627–200885864	8.24×10^{-9}	TRUE	3.71×10–13	1.68×10^{-3}
IBD–BIP	14	1q32.1	200870754-201024059	*KIF21B*	23046	200937514–200993828	1.36×10^{-5}	TRUE	2.48×10^{-8}	8.20×10^{-3}
IBD–BIP	14	1q32.1	200870754-201024059	*CACNA1S*	779	201007635–201082694	8.29×10^{-4}	TRUE	5.28×10^{-6}	6.68×10^{-2}
IBD–BIP	15	2q31.3	182308352-182334753	*ITGA4*	3676	182320619–182403474	5.81×10^{-3}	TRUE	4.39×10^{-4}	9.99×10^{-2}
IBD–BIP	16	8q12.1	59800835-59925249	*TOX*	9760	59716977–60032767	2.35×10^{-5}	TRUE	3.09×10^{-5}	1.02×10^{-3}
IBD–BIP	17	10q21.2	64387108-64441247	*ZNF365*	22891	64132916–64432771	2.39×10^{-9}	TRUE	1.23×10^{-9}	7.20×10^{-7}
IBD–BIP	18	16p12.2	23892887-23962504	*PRKCB*	5579	23846300–24232932	3.15×10^{-7}	TRUE	1.17×10^{-4}	7.43×10^{-7}
IBD–AN	19	1q32.1	200864267-201024059	*INAVA*	55765	200859627–200885864	1.13×10^{-8}	TRUE	3.71×10–13	1.75×10^{-3}
IBD–AN	19	1q32.1	200864267-201024059	*KIF21B*	23046	200937514–200993828	3.48×10^{-6}	TRUE	2.48×10^{-8}	9.25×10^{-3}
IBD–AN	19	1q32.1	200864267-201024059	*CACNA1S*	779	201007635–201082694	2.22×10^{-3}	TRUE	5.28×10^{-6}	3.78×10^{-1}
IBD–AN	20	3p21.31	48446237-50519141	*IP6K2*	51447	48724436–48755711	3.63×10^{-11}	TRUE	4.34×10^{-4}	3.45×10^{-13}
IBD–AN	20	3p21.31	48446237-50519141	*IHO1*	339834	49234861–49296636	6.79×10^{-11}	TRUE	6.99×10^{-5}	2.29×10^{-12}
IBD–AN	20	3p21.31	48446237-50519141	*MST1*	4485	49720380–49727196	9.26×10^{-11}	TRUE	1.23×10^{-7}	2.46×10^{-6}
IBD–AN	20	3p21.31	48446237-50519141	*C3orf62*	375341	49305030–49315864	1.28×10^{-10}	TRUE	1.35×10^{-5}	3.75×10^{-13}
IBD–AN	20	3p21.31	48446237-50519141	*BSN*	8927	49590922–49709982	3.09×10^{-10}	TRUE	3.30×10^{-7}	4.28×10^{-7}
IBD–AN	20	3p21.31	48446237-50519141	*KLHDC8B*	200942	49207987–49214919	3.24×10^{-10}	TRUE	2.85×10^{-4}	1.02×10^{-8}
IBD–AN	20	3p21.31	48446237-50519141	*APEH*	327	49710427–49721936	4.01×10^{-10}	TRUE	3.62×10^{-7}	6.38×10^{-7}

©2023 Gong W et al. *JAMA Psychiatry*.

IBD–AN	20	3p21.31	48446237-50519141	*AMT*	275	49453211–49461111	5.13×10^{-10}	TRUE	9.36×10^{-7}	7.08×10^{-7}
IBD–AN	20	3p21.31	48446237-50519141	*DAG1*	1605	49505136–49574051	7.02×10^{-10}	TRUE	1.32×10^{-6}	2.33×10^{-7}
IBD–AN	20	3p21.31	48446237-50519141	*GPX1*	2876	49393609–49396791	8.37×10^{-10}	TRUE	2.28×10^{-7}	2.22×10^{-5}
IBD–AN	20	3p21.31	48446237-50519141	*NCKIPSD*	51517	48699419–48724366	1.26×10^{-9}	TRUE	4.28×10^{-3}	1.45×10^{-14}
IBD–AN	20	3p21.31	48446237-50519141	*TCTA*	6988	49448639–49454909	1.41×10^{-9}	TRUE	2.73×10^{-7}	1.21×10^{-5}
IBD–AN	20	3p21.31	48446237-50519141	*EPRS1*	5859	49132365–49143562	1.61×10^{-9}	TRUE	1.00×10^{-3}	7.26×10^{-12}
IBD–AN	20	3p21.31	48446237-50519141	*IMPDH2*	3615	49060758–49067875	1.74×10^{-9}	TRUE	2.07×10^{-4}	3.81×10^{-9}
IBD–AN	20	3p21.31	48446237-50519141	*USP4*	7375	49313577–49378536	1.78×10^{-9}	TRUE	1.89×10^{-5}	5.10×10^{-9}
IBD–AN	20	3p21.31	48446237-50519141	*CELSR3*	1951	48672896–48701348	1.94×10^{-9}	TRUE	2.24×10^{-3}	1.71×10^{-12}
IBD–AN	20	3p21.31	48446237-50519141	*SLC26A6*	65010	48662156–48673926	1.19×10^{-8}	TRUE	2.61×10^{-3}	1.44×10^{-10}
IBD–AN	20	3p21.31	48446237-50519141	*QRICH1*	54870	49066140–49132504	1.71×10^{-8}	TRUE	1.11×10^{-3}	1.88×10^{-10}
IBD–AN	20	3p21.31	48446237-50519141	*RHOA*	387	49395569–49450526	4.23×10^{-8}	TRUE	5.59×10^{-6}	5.47×10^{-6}
IBD–AN	20	3p21.31	48446237-50519141	*RNF123*	63891	49725950–49759962	8.97×10^{-8}	TRUE	2.94×10^{-5}	2.20×10^{-6}
IBD–AN	20	3p21.31	48446237-50519141	*NICN1*	84276	49458766–49467777	1.76×10^{-7}	TRUE	2.19×10^{-4}	5.48×10^{-7}
IBD–AN	20	3p21.31	48446237-50519141	*C3orf84*	646498	49214069–49230291	3.31×10^{-7}	TRUE	3.52×10^{-4}	5.81×10^{-8}
IBD–AN	20	3p21.31	48446237-50519141	*CAMKV*	79012	49894414–49908655	9.00×10^{-7}	TRUE	2.74×10^{-3}	4.55×10^{-7}
IBD–AN	20	3p21.31	48446237-50519141	*GMPPB*	29925	49757909–49762407	1.18×10^{-6}	TRUE	6.00×10^{-4}	2.57×10^{-5}
IBD–AN	20	3p21.31	48446237-50519141	*TRAIP*	10293	49865028–49894992	1.37×10^{-6}	TRUE	5.61×10^{-4}	2.42×10^{-6}
IBD–AN	20	3p21.31	48446237-50519141	*IP6K1*	9807	49760728–49824973	1.45×10^{-6}	TRUE	2.62×10^{-4}	2.10×10^{-5}
IBD–AN	20	3p21.31	48446237-50519141	*PRKAR2A*	5576	48783017–48886270	1.46×10^{-6}	TRUE	4.18×10^{-3}	8.62×10^{-9}
IBD–AN	20	3p21.31	48446237-50519141	*INKA1*	389119	49839687–49843463	2.02×10^{-6}	TRUE	8.25×10^{-4}	7.74×10^{-5}
IBD–AN	20	3p21.31	48446237-50519141	*WDR6*	11180	49043637–49054386	2.38×10^{-6}	TRUE	7.61×10^{-3}	3.33×10^{-8}
IBD–AN	20	3p21.31	48446237-50519141	*ARIH2*	10425	48955265–49023974	3.30×10^{-6}	TRUE	8.31×10^{-3}	1.48×10^{-8}
IBD–AN	20	3p21.31	48446237-50519141	*USP19*	10869	49144479–49159371	8.34×10^{-6}	TRUE	1.12×10^{-2}	2.93×10^{-8}
IBD–AN	20	3p21.31	48446237-50519141	*NPRL2*	10641	50383918–50389486	1.07×10^{-5}	TRUE	1.68×10^{-2}	2.76×10^{-6}
IBD–AN	20	3p21.31	48446237-50519141	*P4HTM*	54681	49026304–49045582	1.08×10^{-5}	TRUE	8.26×10^{-3}	1.66×10^{-7}
IBD–AN	20	3p21.31	48446237-50519141	*TUSC2*	11334	50361341–50366668	1.23×10^{-5}	TRUE	1.89×10^{-4}	1.15×10^{-6}

©2023 Gong W et al. *JAMA Psychiatry*.

IBD–AN	20	3p21.31	48446237-50519141	*RBM6*	10180	49976474–50115685	1.83×10^{-5}	TRUE	4.87×10^{-3}	1.91×10^{-5}
IBD–AN	20	3p21.31	48446237-50519141	*CDHR4*	389118	49827165–49838254	2.02×10^{-5}	TRUE	1.36×10^{-4}	1.22×10^{-4}
IBD–AN	20	3p21.31	48446237-50519141	*UBA7*	7318	49841638–49852391	2.52×10^{-5}	TRUE	2.43×10^{-3}	3.49×10^{-5}
IBD–AN	20	3p21.31	48446237-50519141	*TMEM89*	440955	48657275–48660189	3.80×10^{-5}	TRUE	7.68×10^{-3}	3.39×10^{-4}
IBD–AN	20	3p21.31	48446237-50519141	*RBM5*	10181	50125341–50157397	3.89×10^{-5}	TRUE	2.00×10^{-3}	2.34×10^{-4}
IBD–AN	20	3p21.31	48446237-50519141	*CYB561D2*	11068	50387265–50392500	9.47×10^{-5}	TRUE	8.22×10^{-2}	4.47×10^{-5}
IBD–AN	20	3p21.31	48446237-50519141	*MST1R*	4486	49923435–49942311	1.68×10^{-4}	TRUE	2.43×10^{-2}	1.29×10^{-4}
IBD–AN	20	3p21.31	48446237-50519141	*UQCRC1*	7384	48635432–48648391	2.00×10^{-4}	TRUE	2.51×10^{-2}	7.57×10^{-5}
IBD–AN	20	3p21.31	48446237-50519141	*MON1A*	84315	49945302–49968445	2.05×10^{-4}	TRUE	7.31×10^{-3}	6.69×10^{-4}
IBD–AN	20	3p21.31	48446237-50519141	*SLC25A20*	788	48893356–48937426	2.77×10^{-4}	TRUE	5.62×10^{-2}	1.51×10^{-7}
IBD–AN	20	3p21.31	48446237-50519141	*LSMEM2*	132228	50315458–50326545	2.85×10^{-4}	TRUE	7.37×10^{-2}	1.23×10^{-5}
IBD–AN	20	3p21.31	48446237-50519141	*COL7A1*	1294	48600506–48633593	6.63×10^{-4}	TRUE	2.21×10^{-2}	6.94×10^{-5}
IBD–AN	20	3p21.31	48446237-50519141	*CCDC71*	64925	49198968–49204785	6.87×10^{-4}	FALSE	1.16×10^{-1}	6.59×10^{-9}
IBD–AN	20	3p21.31	48446237-50519141	*GNAT1*	2779	50228043–50236129	7.62×10^{-4}	FALSE	5.13×10^{-3}	1.95×10^{-3}
IBD–AN	20	3p21.31	48446237-50519141	*SEMA3F*	6405	50191562–50227508	2.71×10^{-3}	FALSE	6.97×10^{-3}	8.74×10^{-3}
IBD–AN	20	3p21.31	48446237-50519141	*LAMB2*	3913	49157547–49171599	5.35×10^{-3}	FALSE	3.71×10^{-3}	6.37×10^{-4}
IBD–AN	20	3p21.31	48446237-50519141	*ARIH2OS*	646450	48954221–48957818	5.54×10^{-3}	FALSE	1.96×10^{-1}	4.00×10^{-6}
IBD–AN	20	3p21.31	48446237-50519141	*PFKFB4*	5210	48554117–48599607	8.89×10^{-3}	FALSE	2.67×10^{-2}	3.98×10^{-2}
IBD–AN	20	3p21.31	48446237-50519141	*SHISA5*	51246	48508197–48543259	1.39×10^{-2}	FALSE	3.53×10^{-2}	3.58×10^{-2}
IBD–AN	20	3p21.31	48446237-50519141	*CACNA2D2*	9254	50399230–50541892	2.87×10^{-2}	FALSE	2.42×10^{-1}	1.31×10^{-2}
IBD–AN	20	3p21.31	48446237-50519141	*UCN2*	90226	48598151–48602201	3.03×10^{-2}	FALSE	1.28×10^{-1}	1.57×10^{-1}
IBD–AN	20	3p21.31	48446237-50519141	*PLXNB1*	5364	48444261–48472460	5.91×10^{-2}	FALSE	2.32×10^{-1}	4.11×10^{-3}
IBD–AN	20	3p21.31	48446237-50519141	*ATRIP*	84126	48487114–48510044	8.73×10^{-2}	FALSE	3.58×10^{-1}	1.44×10^{-2}
IBD–AN	20	3p21.31	48446237-50519141	*SLC38A3*	10991	50241692–50259406	1.21×10^{-1}	FALSE	3.66×10^{-2}	3.41×10^{-1}
IBD–AN	20	3p21.31	48446237-50519141	*TMA7*	51372	48480686–48486537	1.43×10^{-1}	FALSE	4.78×10^{-1}	1.68×10^{-2}
IBD–AN	20	3p21.31	48446237-50519141	*DALRD3*	55152	49051832–49059504	1.47×10^{-1}	FALSE	1.43×10^{-1}	5.32×10^{-3}
IBD–AN	20	3p21.31	48446237-50519141	*CCDC51*	79714	48472580–48482529	1.64×10^{-1}	FALSE	3.78×10^{-1}	2.40×10^{-2}

©2023 Gong W et al. *JAMA Psychiatry*.

IBD–AN	20	3p21.31	48446237-50519141	*TREX1*	11277	48505919–48510044	2.56×10^{-1}	FALSE	7.46×10^{-1}	1.96×10^{-2}
IBD–AN	20	3p21.31	48446237-50519141	*IFRD2*	7866	50324163–50331648	2.64×10^{-1}	FALSE	6.13×10^{-1}	3.64×10^{-2}
IBD–AN	20	3p21.31	48446237-50519141	*GNAI2*	2771	50263120–50297786	2.95×10^{-1}	FALSE	3.64×10^{-1}	4.52×10^{-2}
IBD–AN	20	3p21.31	48446237-50519141	*SEMA3B*	7869	50304040–50315573	3.50×10^{-1}	FALSE	4.02×10^{-2}	4.86×10^{-1}
IBD–AN	20	3p21.31	48446237-50519141	*NAA80*	24142	50332833–50337852	3.73×10^{-1}	FALSE	3.99×10^{-2}	5.49×10^{-1}
IBD–AN	20	3p21.31	48446237-50519141	*AMIGO3*	386724	49753267–49758238	4.65×10^{-1}	FALSE	1.11×10^{-2}	8.45×10^{-1}
IBD–AN	20	3p21.31	48446237-50519141	*HYAL3*	8372	50329259–50337899	5.33×10^{-1}	FALSE	1.40×10^{-1}	6.16×10^{-2}
IBD–AN	20	3p21.31	48446237-50519141	*RASSF1*	11186	50366217–50379367	6.02×10^{-1}	FALSE	6.36×10^{-1}	2.81×10^{-1}
IBD–AN	20	3p21.31	48446237-50519141	*HYAL2*	8692	50354221–50361281	6.83×10^{-1}	FALSE	8.85×10^{-1}	1.42×10^{-1}
IBD–AN	20	3p21.31	48446237-50519141	*ZMYND10*	51364	50377537–50384459	7.84×10^{-1}	FALSE	7.30×10^{-1}	7.28×10^{-1}
IBD–AN	20	3p21.31	48446237-50519141	*HYAL1*	3373	50336320–50350812	8.28×10^{-1}	FALSE	7.55×10^{-1}	4.04×10^{-1}
IBD–AN	20	3p21.31	48446237-50519141	*NDUFAF3*	25915	49056908–49061928	9.05×10^{-1}	FALSE	6.60×10^{-2}	9.85×10^{-1}
IBD–AN	20	3p21.31	48446237-50519141	*TMEM115*	11070	50391180–50397939	9.14×10^{-1}	FALSE	9.86×10^{-1}	4.74×10^{-1}
IBS–MDD	21	1q25.1	175902660-176406835	*COP1*	64326	175912967–176177386	2.02×10^{-5}	TRUE	5.90×10^{-5}	1.18×10^{-5}
IBS–MDD	22	5q14.3	87514778-87822672	*TMEM161B*	153396	87484450–87565665	1.22×10^{-6}	TRUE	7.86×10^{-6}	1.12×10^{-5}
IBS–MDD	23	11q23.2	112826867-112912811	*NCAM1*	4684	112830969–113150158	3.46×10^{-7}	TRUE	8.71×10^{-8}	9.97×10^{-6}
IBS–MDD	24	13q14.3	53617781-54049489	*OLFM4*	10562	53601876–53627196	3.73×10^{-4}	TRUE	2.26×10^{-3}	1.74×10^{-4}
IBS–MDD	25	14q21.1	41969803-42183025	*LRFN5*	145581	42075764–42374752	1.84×10^{-7}	TRUE	8.74×10^{-4}	2.03×10^{-10}
IBS–MDD	27	18q21.2	53077795-53125364	*TCF4*	6925	52888562–53304252	1.04×10^{-4}	TRUE	4.49×10^{-2}	2.82×10^{-13}
IBS–SCZ	28	1p32.3	53658317-53752134	*LRP8*	7804	53707036–53794821	1.74×10^{-7}	TRUE	4.25×10^{-6}	3.09×10^{-6}
IBS–SCZ	28	1p32.3	53658317-53752134	*CPT2*	1376	53661101–53680869	2.49×10^{-5}	TRUE	1.87×10^{-4}	1.53×10^{-4}
IBS–SCZ	28	1p32.3	53658317-53752134	*MAGOH*	4116	53691564–53705282	4.87×10^{-5}	TRUE	4.66×10^{-5}	1.07×10^{-3}
IBS–SCZ	28	1p32.3	53658317-53752134	*CZIB*	54987	53678771–53687289	8.73×10^{-5}	TRUE	2.58×10^{-4}	9.49×10^{-4}
IBS–SCZ	29	1p21.3	98325796-98559093	*DPYD*	1806	97542299–98387615	8.19×10^{-6}	TRUE	4.55×10^{-2}	5.26×10^{-18}
IBS–SCZ	31	3q22.3	135807609-136673157	*PCCB*	5096	135968167–136057737	8.65×10^{-7}	TRUE	8.84×10^{-4}	1.37×10^{-9}
IBS–SCZ	31	3q22.3	135807609-136673157	*STAG1*	10274	136054077–136472245	8.71×10^{-7}	TRUE	2.22×10^{-4}	5.81×10^{-8}
IBS–SCZ	31	3q22.3	135807609-136673157	*PPP2R3A*	5523	135683515–135867752	4.62×10^{-5}	TRUE	7.65×10^{-4}	9.86×10^{-6}

©2023 Gong W et al. *JAMA Psychiatry*.

IBS–SCZ	31	3q22.3	135807609-136673157	*MSL2*	55167	135866760–135916522	7.78×10^{-4}	TRUE	3.89×10^{-3}	9.38×10^{-6}
IBS–SCZ	31	3q22.3	135807609-136673157	*NCK1*	4690	136580050–136671446	7.92×10^{-4}	TRUE	3.72×10^{-2}	1.59×10^{-5}
IBS–SCZ	31	3q22.3	135807609-136673157	*SLC35G2*	80723	136536861–136575734	9.33×10^{-4}	TRUE	3.78×10^{-2}	1.22×10^{-5}
IBS–SCZ	33	9q22.31	96161300-96358301	*FAM120A*	23196	96213173–96329397	2.55×10^{-7}	TRUE	1.09×10^{-8}	1.01×10^{-5}
IBS–SCZ	33	9q22.31	96161300-96358301	*PHF2*	5253	96337909–96442869	5.98×10^{-6}	TRUE	6.55×10^{-8}	5.64×10^{-5}
IBS–SCZ	33	9q22.31	96161300-96358301	*FAM120AOS*	158293	96207776–96216874	7.50×10^{-4}	TRUE	1.06×10^{-4}	1.86×10^{-5}
IBS–SCZ	34	11q24.2	124619407-124624854	*VSIG2*	23584	124616370–124623109	2.04×10^{-6}	TRUE	3.96×10^{-3}	1.45×10^{-8}
IBS–SCZ	34	11q24.2	124619407-124624854	*ESAM*	90952	124622019–124633223	5.91×10^{-6}	TRUE	5.08×10^{-3}	1.99×10^{-9}
IBS–SCZ	36	12q24.31	123447928-123897177	*PITPNM2*	57605	123467027–123636376	6.30×10^{-8}	TRUE	7.52×10^{-3}	2.73×10^{-13}
IBS–SCZ	36	12q24.31	123447928-123897177	*MPHOSPH9*	10198	123639943–123718785	1.23×10^{-7}	TRUE	6.81×10^{-3}	1.85×10^{-12}
IBS–SCZ	36	12q24.31	123447928-123897177	*ARL6IP4*	51329	123463607–123468460	1.53×10^{-7}	TRUE	6.85×10^{-3}	2.88×10^{-11}
IBS–SCZ	36	12q24.31	123447928-123897177	*OGFOD2*	79676	123458354–123465588	1.65×10^{-7}	TRUE	8.33×10^{-3}	3.77×10^{-11}
IBS–SCZ	36	12q24.31	123447928-123897177	*MTRFR*	91574	123716844–123743651	2.08×10^{-7}	TRUE	1.52×10^{-2}	1.15×10^{-14}
IBS–SCZ	36	12q24.31	123447928-123897177	*CDK2AP1*	8099	123744517–123757863	1.52×10^{-6}	TRUE	3.68×10^{-2}	4.55×10^{-13}
IBS–SCZ	36	12q24.31	123447928-123897177	*KMT5A*	387893	123867704–123894900	2.32×10^{-5}	TRUE	4.94×10^{-2}	2.68×10^{-9}
IBS–SCZ	36	12q24.31	123447928-123897177	*SBNO1*	55206	123772656–123850756	2.08×10^{-3}	TRUE	1.51×10^{-1}	1.73×10^{-7}
IBS–SCZ	36	12q24.31	123447928-123897177	*ABCB9*	23457	123404498–123452056	2.17×10^{-3}	TRUE	4.01×10^{-1}	2.02×10^{-7}
IBS–SCZ	36	12q24.31	123447928-123897177	*LOC100293704*	100293704	123847046–123851336	1.08×10^{-2}	FALSE	3.55×10^{-1}	1.04×10^{-6}
IBS–SCZ	37	22q13.2	41408754-41854446	*RANGAP1*	5905	41639781–41683255	4.70×10^{-7}	TRUE	4.85×10^{-5}	1.86×10^{-6}
IBS–SCZ	37	22q13.2	41408754-41854446	*ZC3H7B*	23264	41696507–41757151	6.25×10^{-6}	TRUE	1.37×10^{-5}	8.08×10^{-5}
IBS–SCZ	37	22q13.2	41408754-41854446	*EP300*	2033	41487614–41577081	1.08×10^{-4}	TRUE	1.94×10^{-3}	4.89×10^{-5}
IBS–SCZ	37	22q13.2	41408754-41854446	*LOC100996598*	100996598	41764086–41767400	1.81×10^{-4}	TRUE	1.02×10^{-4}	9.22×10^{-3}
IBS–SCZ	37	22q13.2	41408754-41854446	*TEF*	7008	41762337–41796332	4.07×10^{-4}	TRUE	5.61×10^{-5}	3.44×10^{-3}
IBS–SCZ	37	22q13.2	41408754-41854446	*CHADL*	150356	41604861–41637935	5.09×10^{-4}	TRUE	5.94×10^{-3}	2.24×10^{-4}
IBS–SCZ	37	22q13.2	41408754-41854446	*L3MBTL2*	83746	41600312–41628276	6.62×10^{-4}	TRUE	6.94×10^{-3}	2.50×10^{-4}
IBS–SCZ	37	22q13.2	41408754-41854446	*TOB2*	10766	41828492–41845234	7.50×10^{-4}	TRUE	6.82×10^{-5}	8.82×10^{-3}
IBS–ADHD	38	1p34.2	43788858-43949810	*SZT2*	23334	43854556–43920918	1.28×10^{-6}	TRUE	1.19×10^{-2}	5.03×10^{-8}

©2023 Gong W et al. *JAMA Psychiatry*.

IBS–ADHD	38	1p34.2	43788858-43949810	*HYI*	81888	43915674–43920938	8.45×10^{-6}	TRUE	1.01×10^{-2}	1.34×10^{-6}
IBS–ADHD	38	1p34.2	43788858-43949810	*MED8*	112950	43848579–43856483	1.00×10^{-4}	TRUE	1.57×10^{-1}	1.76×10^{-8}
IBS–ADHD	38	1p34.2	43788858-43949810	*TIE1*	7075	43765566–43789781	1.21×10^{-4}	TRUE	7.24×10^{-2}	6.30×10^{-8}
IBS–ADHD	38	1p34.2	43788858-43949810	*MPL*	4352	43802475–43821135	3.36×10^{-4}	TRUE	1.46×10^{-1}	8.46×10^{-8}
IBS–ADHD	38	1p34.2	43788858-43949810	*CDC20*	991	43823626–43829874	4.92×10^{-4}	TRUE	1.42×10^{-1}	1.76×10^{-7}
IBS–ADHD	38	1p34.2	43788858-43949810	*ELOVL1*	64834	43828068–43834745	7.59×10^{-3}	FALSE	5.11×10^{-1}	1.31×10^{-6}
IBS–ADHD	39	5q14.3	87514778-87932809	*TMEM161B*	153396	87484450–87565665	7.53×10^{-8}	TRUE	7.86×10^{-6}	1.46×10^{-4}
IBS–BIP	40	1p32.3	53658317-53752134	*LRP8*	7804	53707036–53794821	7.10×10^{-7}	TRUE	4.25×10^{-6}	1.42×10^{-4}
IBS–BIP	40	1p32.3	53658317-53752134	*MAGOH*	4116	53691564–53705282	2.26×10^{-6}	TRUE	4.66×10^{-5}	1.88×10^{-4}
IBS–BIP	40	1p32.3	53658317-53752134	*CZIB*	54987	53678771–53687289	9.00×10^{-6}	TRUE	2.58×10^{-4}	2.23×10^{-4}
IBS–BIP	40	1p32.3	53658317-53752134	*CPT2*	1376	53661101–53680869	1.84×10^{-5}	TRUE	1.87×10^{-4}	4.40×10^{-4}
IBS–BIP	42	3p12.1	85002871-85671909	*CADM2*	253559	85007133–86124579	1.38×10-10	TRUE	4.56×10^{-8}	3.51×10^{-7}
IBS–BIP	43	4q27	123122856-123558330	*IL2*	3558	123371625–123378650	1.48×10^{-5}	TRUE	3.54×10^{-3}	1.94×10^{-6}
IBS–BIP	43	4q27	123122856-123558330	*IL21*	59067	123532783–123543212	1.29×10^{-3}	TRUE	1.06×10^{-2}	1.41×10^{-4}
IBS–BIP	43	4q27	123122856-123558330	*BLTP1*	84162	123072488–123284914	1.72×10^{-3}	TRUE	1.08×10^{-2}	1.61×10^{-4}
IBS–BIP	43	4q27	123122856-123558330	*ADAD1*	132612	123299121–123351957	5.30×10^{-3}	TRUE	1.38×10^{-2}	3.07×10^{-4}
IBS–BIP	44	7q21.11	82387493-82583609	*PCLO*	27445	82382321–82793197	4.49×10^{-9}	TRUE	1.95×10^{-6}	2.72×10^{-6}
IBS–BIP	45	8p23.1	9881136-10006664	*MSRA*	4482	9910830–10287401	2.34×10^{-7}	TRUE	5.91×10^{-3}	1.11×10^{-10}
IBS–BIP	46	10q25.1	111648659-111928784	*XPNPEP1*	7511	111623524–111684311	7.78×10^{-6}	TRUE	5.63×10^{-3}	4.93×10^{-7}
IBS–BIP	46	10q25.1	111648659-111928784	*ADD3*	120	111764627–111896323	2.62×10^{-5}	TRUE	3.34×10^{-1}	1.74×10^{-9}
IBS–BIP	47	11q23.2	112826867-113034787	*NCAM1*	4684	112830969–113150158	2.08×10^{-9}	TRUE	8.71×10^{-8}	2.86×10^{-6}
IBS–AN	49	9q22.31	96163260-96356004	*FAM120A*	23196	96213173–96329397	6.34×10^{-8}	TRUE	1.09×10^{-8}	1.60×10^{-3}
IBS–AN	49	9q22.31	96163260-96356004	*FAM120AOS*	158293	96207776–96216874	2.19×10^{-4}	TRUE	1.06×10^{-4}	2.36×10^{-2}
IBS–AN	49	9q22.31	96163260-96356004	*PHF2*	5253	96337909–96442869	3.09×10^{-3}	TRUE	6.55×10^{-8}	1.97×10^{-1}
IBS–AN	50	11q23.2	112826311-113062983	*NCAM1*	4684	112830969–113150158	6.57×10-11	TRUE	8.71×10^{-8}	2.15×10^{-7}
PUD–SCZ	51	1p21.3	98327133-98559093	*DPYD*	1806	97542299–98387615	1.48×10^{-4}	TRUE	1.94×10^{-1}	5.26×10^{-18}
PUD–SCZ	52	8q24.3	143752994-143809193	*LY6K*	54742	143780529–143786588	2.99×10^{-7}	TRUE	1.42×10–10	5.45×10^{-3}

©2023 Gong W et al. *JAMA Psychiatry*.

PUD–SCZ	52	8q24.3	143752994-143809193	*PSCA*	8000	143750726–143765145	4.08×10^{-7}	TRUE	1.43×10–13	1.50×10^{-2}
PUD–SCZ	52	8q24.3	143752994-143809193	*THEM6*	51337	143807621–143819350	2.39×10^{-5}	TRUE	1.56×10–10	9.47×10^{-3}
PUD–SCZ	54	19q13.33	49103447-49254955	*FUT2*	2524	49198228–49210191	3.13×10-12	TRUE	4.86×10–14	6.24×10^{-6}
PUD–SCZ	54	19q13.33	49103447-49254955	*RASIP1*	54922	49222842–49245136	1.06×10-10	TRUE	1.32×10–11	1.32×10^{-5}
PUD–SCZ	54	19q13.33	49103447-49254955	*MAMSTR*	284358	49214999–49223976	2.96×10-10	TRUE	2.44×10–11	2.51×10^{-5}
PUD–SCZ	54	19q13.33	49103447-49254955	*IZUMO1*	284359	49243073–49251831	2.79×10^{-9}	TRUE	8.28×10^{-9}	1.40×10^{-5}
PUD–SCZ	54	19q13.33	49103447-49254955	*FUT1*	2523	49250268–49259647	3.89×10^{-8}	TRUE	3.29×10^{-7}	2.68×10^{-5}
PUD–SCZ	54	19q13.33	49103447-49254955	*NTN5*	126147	49163664–49177264	9.01×10^{-6}	TRUE	1.39×10^{-6}	6.41×10^{-4}
PUD–SCZ	54	19q13.33	49103447-49254955	*FAM83E*	54854	49102857–49119098	3.08×10^{-4}	TRUE	1.40×10^{-4}	3.96×10^{-3}
PUD–SCZ	54	19q13.33	49103447-49254955	*SPHK2*	56848	49121548–49134974	9.81×10^{-3}	FALSE	7.05×10^{-3}	1.05×10^{-2}
PUD–SCZ	54	19q13.33	49103447-49254955	*SPACA4*	171169	49109000–49111971	1.50×10^{-2}	FALSE	2.19×10^{-1}	5.10×10^{-3}
PUD–SCZ	54	19q13.33	49103447-49254955	*RPL18*	6141	49117584–49123675	1.51×10^{-2}	FALSE	8.29×10^{-3}	5.59×10^{-2}
PUD–SCZ	54	19q13.33	49103447-49254955	*DBP*	1628	49132817–49141639	1.65×10^{-2}	FALSE	1.05×10^{-2}	4.49×10^{-2}
PUD–SCZ	54	19q13.33	49103447-49254955	*SULT2B1*	6820	49054429–49103684	2.02×10^{-2}	FALSE	1.55×10^{-2}	2.13×10^{-2}
PUD–SCZ	54	19q13.33	49103447-49254955	*CA11*	770	49140272–49150451	2.30×10^{-2}	FALSE	1.53×10^{-2}	5.47×10^{-2}
PUD–ADHD	55	1p34.1	44197228-44480093	*ST3GAL3*	6487	44172204–44397837	2.98×10^{-6}	TRUE	1.71×10^{-2}	1.64×10^{-11}
PUD–ADHD	55	1p34.1	44197228-44480093	*ARTN*	9048	44397992–44403912	1.06×10^{-3}	TRUE	1.77×10^{-1}	4.46×10^{-6}
PUD–ADHD	55	1p34.1	44197228-44480093	*CCDC24*	149473	44456280–44463200	1.86×10^{-3}	TRUE	2.06×10^{-1}	9.46×10^{-6}
PUD–ADHD	55	1p34.1	44197228-44480093	*SLC6A9*	6536	44461155–44498171	4.18×10^{-3}	TRUE	1.90×10^{-1}	5.97×10^{-6}
PUD–ADHD	55	1p34.1	44197228-44480093	*B4GALT2*	8704	44443874–44457843	7.75×10^{-3}	FALSE	1.93×10^{-1}	4.04×10^{-5}
PUD–ADHD	55	1p34.1	44197228-44480093	*IPO13*	9670	44411478–44434694	2.16×10^{-2}	FALSE	2.85×10^{-1}	4.97×10^{-4}
PUD–ADHD	55	1p34.1	44197228-44480093	*DPH2*	1802	44434653–44440043	2.86×10^{-2}	FALSE	3.28×10^{-1}	7.98×10^{-4}
PUD–ADHD	55	1p34.1	44197228-44480093	*ATP6V0B*	533	44439118–44444972	3.26×10^{-2}	FALSE	3.87×10^{-1}	3.36×10^{-5}
PUD–ADHD	56	12q21.33	89726027-89776845	*DUSP6*	1848	89740837–89747296	2.53×10^{-7}	TRUE	1.87×10^{-3}	7.08×10^{-9}
PUD–ADHD	57	19q13.33	49168942-49250239	*FUT2*	2524	49198228–49210191	2.16×10-10	TRUE	4.86×10–14	4.92×10^{-3}
PUD–ADHD	57	19q13.33	49168942-49250239	*MAMSTR*	284358	49214999–49223976	1.69×10^{-7}	TRUE	2.44×10–11	4.41×10^{-2}
PUD–ADHD	57	19q13.33	49168942-49250239	*RASIP1*	54922	49222842–49245136	1.36×10^{-6}	TRUE	1.32×10–11	9.32×10^{-2}

©2023 Gong W et al. *JAMA Psychiatry*.

PUD–ADHD	57	19q13.33	49168942-49250239	*NTN5*	126147	49163664–49177264	2.39×10^{-4}	TRUE	1.39×10^{-6}	7.32×10^{-2}
PUD–ADHD	57	19q13.33	49168942-49250239	*IZUMO1*	284359	49243073–49251831	4.87×10^{-4}	TRUE	8.28×10^{-9}	2.62×10^{-1}
PUD–BIP	58	3p22.2	36834099-36870230	*TRANK1*	9881	36867308–36987548	6.63×10^{-3}	TRUE	7.13×10^{-1}	3.75×10^{-9}
PUD–BIP	60	8q24.3	143752994-143780261	*PSCA*	8000	143750726–143765145	4.26×10^{-7}	TRUE	1.43×10–13	2.33×10^{-2}
PUD–BIP	61	11q12.2	61542006-61624181	*FADS1*	3992	61566097–61585529	3.34×10^{-8}	TRUE	5.53×10^{-3}	2.76×10^{-12}
PUD–BIP	61	11q12.2	61542006-61624181	*FADS2*	9415	61582675–61635826	3.54×10^{-8}	TRUE	2.96×10^{-3}	2.63×10^{-12}
PUD–BIP	61	11q12.2	61542006-61624181	*TMEM258*	746	61555602–61561085	2.96×10^{-7}	TRUE	1.96×10^{-2}	8.21×10^{-12}
PUD–BIP	61	11q12.2	61542006-61624181	*FEN1*	2237	61559109–61565716	7.98×10^{-7}	TRUE	1.70×10^{-2}	1.16×10^{-9}
PUD–BIP	61	11q12.2	61542006-61624181	*MYRF*	745	61519121–61556990	4.34×10^{-6}	TRUE	4.61×10^{-2}	1.68×10^{-11}
PUD–AN	62	4q31.22	147216084-147337374	*SLC10A7*	84068	147174137–147444123	1.79×10^{-5}	TRUE	2.12×10^{-3}	9.29×10^{-5}
PUD–AN	63	8q24.3	143752994-143809193	*PSCA*	8000	143750726–143765145	7.55×10^{-9}	TRUE	1.43×10–13	8.61×10^{-3}
PUD–AN	63	8q24.3	143752994-143809193	*LY6K*	54742	143780529–143786588	4.09×10^{-6}	TRUE	1.42×10–10	5.65×10^{-2}
PUD–AN	63	8q24.3	143752994-143809193	*THEM6*	51337	143807621–143819350	8.61×10^{-6}	TRUE	1.56×10–10	9.56×10^{-2}
GORD–MDD	64	2q33.3	208017033-208088987	*KLF7*	8609	207937861–208032970	1.17×10^{-4}	TRUE	1.63×10^{-4}	1.09×10^{-4}
GORD–MDD	65	11q23.2	112826867-112938783	*NCAM1*	4684	112830969–113150158	6.80×10^{-7}	TRUE	2.89×10^{-7}	9.97×10^{-6}
GORD–PTSD	66	4q24	102938709-103438709	*SLC39A8*	64116	103171198–103267655	2.22×10^{-1}	FALSE	3.91×10^{-1}	3.51×10^{-2}
GORD–PTSD	66	4q24	102938709-103438709	*NFKB1*	4790	103421486–103539459	3.67×10^{-1}	FALSE	6.43×10^{-1}	1.63×10^{-1}
GORD–PTSD	66	4q24	102938709-103438709	*BANK1*	55024	102710764–102996969	7.25×10^{-1}	FALSE	5.91×10^{-1}	7.08×10^{-1}
GORD–SCZ	67	1p31.3	66304167-66333877	*PDE4B*	5142	66257193–66841262	1.43×10^{-4}	TRUE	9.63×10^{-3}	3.54×10^{-7}
GORD–SCZ	68	1p21.3	98298371-98559093	*DPYD*	1806	97542299–98387615	3.12×10^{-7}	TRUE	8.73×10^{-4}	5.26×10^{-18}
GORD–SCZ	70	2q37.1	233559312-233806771	*GIGYF2*	26058	233561015–233726287	1.41×10^{-7}	TRUE	1.11×10^{-3}	3.29×10^{-11}
GORD–SCZ	70	2q37.1	233559312-233806771	*KCNJ13*	3769	233629512–233642275	1.81×10^{-7}	TRUE	3.43×10^{-3}	1.14×10^{-13}
GORD–SCZ	70	2q37.1	233559312-233806771	*NGEF*	25791	233742396–233878951	2.87×10^{-6}	TRUE	2.49×10^{-2}	1.40×10^{-13}
GORD–SCZ	70	2q37.1	233559312-233806771	*SNORC*	389084	233732724–233742111	1.30×10^{-4}	TRUE	1.29×10^{-2}	2.02×10^{-6}
GORD–SCZ	71	4q24	102702364-103387161	*SLC39A8*	64116	103171198–103267655	2.64×10^{-2}	FALSE	3.91×10^{-1}	6.30×10^{-6}
GORD–SCZ	71	4q24	102702364-103387161	*BANK1*	55024	102710764–102996969	3.60×10^{-1}	FALSE	5.91×10^{-1}	1.36×10^{-4}
GORD–SCZ	73	8q24.3	143308772-143349510	*TSNARE1*	203062	143292441–143485543	2.99×10^{-3}	TRUE	3.88×10^{-1}	2.40×10^{-10}

©2023 Gong W et al. *JAMA Psychiatry*.

GORD–SCZ	74	10q24.32	104571436-104962011	*BORCS7*	119032	104612967–104625718	2.42×10-10	TRUE	3.33×10^{-4}	2.72×10^{-15}
GORD–SCZ	74	10q24.32	104571436-104962011	*AS3MT*	57412	104628183–104662656	1.78×10^{-7}	TRUE	1.41×10^{-3}	5.00×10^{-10}
GORD–SCZ	74	10q24.32	104571436-104962011	*CYP17A1*	1586	104589288–104598290	7.68×10^{-7}	TRUE	2.79×10^{-3}	1.25×10^{-9}
GORD–SCZ	74	10q24.32	104571436-104962011	*CNNM2*	54805	104677075–104839344	4.69×10^{-6}	TRUE	4.22×10^{-3}	9.12×10^{-13}
GORD–SCZ	74	10q24.32	104571436-104962011	*NT5C2*	22978	104846774–104954063	7.25×10^{-6}	TRUE	3.57×10^{-2}	5.48×10^{-14}
GORD–SCZ	74	10q24.32	104571436-104962011	*WBP1L*	54838	104502727–104577022	3.39×10^{-4}	TRUE	2.25×10^{-2}	5.53×10^{-6}
GORD–SCZ	75	12q24.11	110473245-110973245	*C12orf76*	400073	110464401–110506500	2.20×10^{-2}	FALSE	4.14×10^{-1}	1.62×10^{-6}
GORD–SCZ	75	12q24.11	110473245-110973245	*ARPC3*	10094	110871695–110889222	4.32×10^{-2}	FALSE	6.03×10^{-1}	1.31×10^{-7}
GORD–SCZ	75	12q24.11	110473245-110973245	*ATP2A2*	488	110718032–110789898	4.41×10^{-2}	FALSE	7.63×10^{-1}	1.61×10^{-8}
GORD–SCZ	75	12q24.11	110473245-110973245	*PPTC7*	160760	110971237–111022064	4.79×10^{-2}	FALSE	4.97×10^{-1}	3.30×10^{-8}
GORD–SCZ	75	12q24.11	110473245-110973245	*IFT81*	28981	110561140–110657600	4.86×10^{-2}	FALSE	6.42×10^{-1}	8.77×10^{-9}
GORD–SCZ	75	12q24.11	110473245-110973245	*ANKRD13A*	88455	110435974–110478237	5.28×10^{-2}	FALSE	2.46×10^{-1}	5.70×10^{-4}
GORD–SCZ	75	12q24.11	110473245-110973245	*GPN3*	51184	110889291–110907667	6.77×10^{-2}	FALSE	6.04×10^{-1}	2.62×10^{-8}
GORD–SCZ	75	12q24.11	110473245-110973245	*RAD9B*	144715	110939005–110974952	1.03×10^{-1}	FALSE	6.49×10^{-1}	8.50×10^{-7}
GORD–SCZ	75	12q24.11	110473245-110973245	*FAM216A*	29902	110905232–110929192	1.06×10^{-1}	FALSE	7.71×10^{-1}	1.78×10^{-7}
GORD–SCZ	75	12q24.11	110473245-110973245	*ANAPC7*	51434	110809705–110842535	1.44×10^{-1}	FALSE	8.12×10^{-1}	6.57×10^{-7}
GORD–SCZ	75	12q24.11	110473245-110973245	*VPS29*	51699	110928330–110940916	2.17×10^{-1}	FALSE	9.30×10^{-1}	6.66×10^{-8}
GORD–ADHD	76	3p21.31	49897830-50167424	*CAMKV*	79012	49894414–49908655	2.37×10^{-6}	TRUE	3.01×10^{-6}	3.90×10^{-4}
GORD–ADHD	76	3p21.31	49897830-50167424	*MON1A*	84315	49945302–49968445	4.84×10^{-5}	TRUE	2.23×10^{-6}	2.11×10^{-2}
GORD–ADHD	76	3p21.31	49897830-50167424	*MST1R*	4486	49923435–49942311	1.32×10^{-4}	TRUE	7.12×10^{-6}	2.52×10^{-2}
GORD–ADHD	76	3p21.31	49897830-50167424	*RBM5*	10181	50125341–50157397	1.78×10^{-4}	TRUE	2.58×10^{-6}	2.27×10^{-2}
GORD–ADHD	76	3p21.31	49897830-50167424	*RBM6*	10180	49976474–50115685	2.91×10^{-4}	TRUE	6.16×10^{-6}	2.90×10^{-2}
GORD–ADHD	77	7q31.1	114104389-114287116	*FOXP2*	93986	113725365–114334827	1.92×10^{-5}	TRUE	1.21×10^{-3}	4.25×10^{-6}
GORD–BIP	78	3p21.2	52217088-52467263	*TLR9*	54106	52254096–52261179	5.33×10^{-7}	TRUE	3.23×10^{-3}	1.35×10^{-9}
GORD–BIP	78	3p21.2	52217088-52467263	*ALAS1*	211	52231099–52249343	5.39×10^{-7}	TRUE	2.90×10^{-3}	3.73×10^{-8}
GORD–BIP	78	3p21.2	52217088-52467263	*WDR82*	80335	52287438–52313659	4.31×10^{-4}	TRUE	1.88×10^{-2}	1.95×10^{-5}
GORD–BIP	78	3p21.2	52217088-52467263	*SEMA3G*	56920	52466268–52480112	9.74×10^{-4}	TRUE	1.13×10^{-1}	2.85×10^{-6}

©2023 Gong W et al. *JAMA Psychiatry*.

GORD–BIP	78	3p21.2	52217088-52467263	*GLYCTK*	132158	52320836–52330272	1.81×10^{-3}	TRUE	7.62×10^{-2}	2.31×10^{-5}
GORD–BIP	78	3p21.2	52217088-52467263	*TWF2*	11344	52261626–52274183	2.99×10^{-3}	TRUE	6.59×10^{-2}	4.99×10^{-5}
GORD–BIP	78	3p21.2	52217088-52467263	*PPM1M*	132160	52278782–52285615	4.38×10^{-3}	TRUE	7.78×10^{-2}	1.11×10^{-4}
GORD–BIP	78	3p21.2	52217088-52467263	*DNAH1*	25981	52349335–52435513	1.47×10^{-2}	FALSE	6.98×10^{-2}	5.67×10^{-3}
GORD–BIP	78	3p21.2	52217088-52467263	*PHF7*	51533	52443577–52458657	2.28×10^{-2}	FALSE	3.81×10^{-1}	3.79×10^{-6}
GORD–BIP	78	3p21.2	52217088-52467263	*BAP1*	8314	52434020–52445121	3.72×10^{-2}	FALSE	7.13×10^{-2}	1.79×10^{-2}
GORD–AN	80	3p21.31	49734229-50209053	*RBM6*	10180	49976474–50115685	8.28×10^{-8}	TRUE	6.16×10^{-6}	1.91×10^{-5}
GORD–AN	80	3p21.31	49734229-50209053	*CAMKV*	79012	49894414–49908655	8.97×10^{-8}	TRUE	3.01×10^{-6}	4.55×10^{-7}
GORD–AN	80	3p21.31	49734229-50209053	*RBM5*	10181	50125341–50157397	1.25×10^{-7}	TRUE	2.58×10^{-6}	2.34×10^{-4}
GORD–AN	80	3p21.31	49734229-50209053	*MST1R*	4486	49923435–49942311	1.66×10^{-7}	TRUE	7.12×10^{-6}	1.29×10^{-4}
GORD–AN	80	3p21.31	49734229-50209053	*MON1A*	84315	49945302–49968445	5.68×10^{-7}	TRUE	2.23×10^{-6}	6.69×10^{-4}
GORD–AN	80	3p21.31	49734229-50209053	*RNF123*	63891	49725950–49759962	5.79×10^{-7}	TRUE	2.62×10^{-5}	2.20×10^{-6}
GORD–AN	80	3p21.31	49734229-50209053	*GMPPB*	29925	49757909–49762407	6.53×10^{-7}	TRUE	1.82×10^{-4}	2.57×10^{-5}
GORD–AN	80	3p21.31	49734229-50209053	*IP6K1*	9807	49760728–49824973	1.08×10^{-6}	TRUE	3.39×10^{-5}	2.10×10^{-5}
GORD–AN	80	3p21.31	49734229-50209053	*TRAIP*	10293	49865028–49894992	1.42×10^{-6}	TRUE	5.75×10^{-5}	2.42×10^{-6}
GORD–AN	80	3p21.31	49734229-50209053	*INKA1*	389119	49839687–49843463	2.80×10^{-6}	TRUE	1.33×10^{-3}	7.74×10^{-5}
GORD–AN	80	3p21.31	49734229-50209053	*UBA7*	7318	49841638–49852391	1.17×10^{-5}	TRUE	2.81×10^{-4}	3.49×10^{-5}
GORD–AN	80	3p21.31	49734229-50209053	*SEMA3F*	6405	50191562–50227508	7.57×10^{-5}	TRUE	2.75×10^{-6}	8.74×10^{-3}
GORD–AN	80	3p21.31	49734229-50209053	*CDHR4*	389118	49827165–49838254	2.74×10^{-4}	TRUE	5.10×10^{-5}	1.22×10^{-4}
GORD–AN	80	3p21.31	49734229-50209053	*AMIGO3*	386724	49753267–49758238	4.27×10^{-1}	FALSE	2.88×10^{-3}	8.45×10^{-1}
GORD–AN	81	3p13	70795054-71018894	*FOXP1*	27086	71002865–71634140	1.14×10^{-2}	TRUE	4.38×10^{-2}	4.32×10^{-3}
GORD–AN	82	11q23.2	112826867-112922254	*NCAM1*	4684	112830969–113150158	2.40×10^{-7}	TRUE	2.89×10^{-7}	2.15×10^{-7}
GORD–AN	83	12q13.2	56368708-56478658	*SUOX*	6821	56390043–56400309	7.06×10^{-8}	TRUE	6.81×10^{-5}	8.33×10^{-6}
GORD–AN	83	12q13.2	56368708-56478658	*RAB5B*	5869	56366697–56391467	1.29×10^{-7}	TRUE	4.79×10^{-5}	1.17×10^{-4}
GORD–AN	83	12q13.2	56368708-56478658	*ERBB3*	2065	56472809–56498291	3.09×10^{-7}	TRUE	1.84×10^{-4}	2.38×10^{-5}
GORD–AN	83	12q13.2	56368708-56478658	*RPS26*	6231	56434686–56439007	1.58×10^{-6}	TRUE	1.39×10^{-3}	1.83×10^{-5}
GORD–AN	83	12q13.2	56368708-56478658	*IKZF4*	64375	56400268–56433219	5.13×10^{-3}	TRUE	3.91×10^{-2}	1.69×10^{-2}

©2023 Gong W et al. *JAMA Psychiatry*.

Gene position information was obtained from NCBI build 37.3. P_{PLACO} represents P values for candidate pleiotropic genes from MAGMA analysis performing on PLACO statistics for corresponding pair of traits. Sig_{PLACO} represents whether the genes were identified under the locus-specific Bonferroni correction (0.05/the number of genes in each locus). P_{GIT} and P_{PSY}, P values of genes from MAGMA analysis performing on corresponding single-trait GIT and PSY GWAS, respectively.

©2023 Gong W et al. *JAMA Psychiatry*.

eTable 10. Phenotype Enrichment Results With Existing Phenotype Annotations of the Pleiotropic Genes

ID	Phenotype	Pleiotropic gene group		Non-pleiotropic gene group		*P*	Gene
		1	0	1	0		
MP:0005381	digestive/alimentary phenotype	26	118	1617	17565	1.88×10^{-4}	*CACNA1S, CAMKV, CELSR3, COL7A1, DPYD, EP300, ERBB3, FADS1, FADS2, FEN1, FUT1, FUT2, GPX1, IKZF4, IL2, IP6K2, KCNJ13, MST1, OLFM4, QRICH1, SLC26A6, TFR2, TLR9, TMEM258, TOX, XPNPEP1*
MP:0010768	mortality/aging	62	82	6124	13058	3.40×10^{-3}	*ACHE, ACTL6B, ADAD1, ALAS1, AMT, ARIH2, BSN, CACNA1S, CDK2AP1, CELSR3, CNNM2, COL7A1, CPT2, CRB1, CYP17A1, DAG1, DUSP6, EP300, EPO, ERBB3, FADS1, FADS2, FEN1, FOXP1, FOXP2, FUT2, GIGYF2, IL2, IMPDH2, IP6K2, KCNJ13, KLF7, L3MBTL2, MAGOH, MSRA, MST1R, MYRF, NCAM1, PCCB, PCLO, PDE4B, PHF2, POP7, PRMT1, RANGAP1, RASIP1, RHOA, RPS26, SEMA3F, SRRT, STAG1, SZT2, TCF4, TLR9, TMEM161B, TMEM258, TRAIP, TUSC2, UQCRC1, USP19, WBP1L, XPNPEP1*
MP:0005384	cellular phenotype	51	93	4868	14314	4.91×10^{-3}	*ADAD1, ARIH2, C3orf62, CACNA1S, CDK2AP1, CELSR3, CYP17A1, DAG1, DUSP6, EP300, EPO, ERBB3, ESAM, FADS1, FADS2, FEN1, FOXP1, FOXP2, GIGYF2, GPX1, IKZF4, IL2, IP6K1, IP6K2, KLF7, LRP8, LY6K, MAGOH, MSRA, MST1R, NCAM1, NCKIPSD, NGEF, PDE4B, PRKCB, PRMT1, RASIP1, RBM5, RHOA, RPS26, SEMA3F, SRRT, STAG1, TLR9, TMEM258, TOB2, TRAIP, TRIP6, TUSC2, USP19, USP4*
MP:0005387	immune system phenotype	48	96	4687	14495	1.04×10^{-2}	*ACHE, ARIH2, BCL2L12, COL7A1, CPT2, DENND1B, DUSP6, EP300, EPO, ERBB3, ESAM, FADS1, FADS2, FEN1, FOXP1, FOXP2, FUT2, GIGYF2, GPX1, IKZF4, IL2, IL21, IMPDH2, IP6K2, IRF3, MST1, MST1R, OLFM4, PDE4B, PRKCB, PSCA, QRICH1, RHOA, RRAS, SEMA3F, SRRT, STAG1, TCF4, TFR2, TLR9, TMEM258, TOB2, TOX, TRIP6, TUSC2, USP4, WBP1L, XPNPEP1*
MP:0005386	behavior/neurological phenotype	50	94	4983	14199	1.28×10^{-2}	*ACHE, ACTL6B, APEH, BSN, C3orf84, CACNA1S, CAMKV, CELSR3, CNNM2, CYP17A1, DAG1, DPYD, ERBB3, FADS1, FADS2, FAM120A, FOXP1, FOXP2, GIGYF2, GNB2, IKZF4, IL2, IRF3, KCNJ13, KIF21B, KLF7, LRP8, MON1A, MSRA, MYRF, NCAM1, NT5C2, PDE4B, PPP2R3A, PRKCB, RAB5B, RBM6, RPS26, RRAS, SEMA3F, SGIP1, SZT2, TCF4, TEF, TOX, TUSC2, UQCRC1, USP19, WBP1L, WDR6*
MP:0003631	nervous system phenotype	41	103	3981	15201	1.75×10^{-2}	*ACHE, ACTL6B, AMT, APEH, BSN, CACNA1S, CELSR3, CRB1, DAG1, EP300, ERBB3, FADS1, FEN1, FOXP2, GIGYF2, GPX1, IP6K1, KIF21B, KLF7, LRP8, MAGOH, MPHOSPH9, MYRF, NCAM1, NCKIPSD, NGEF, NTN5, PCLO, PDE4B, PHF2, RHOA, SEMA3F, SZT2, TCF4, TEF, TOX, TRIP6, UQCRC1, WBP1L, XPNPEP1, ZNF365*

©2023 Gong W et al. *JAMA Psychiatry*.

MP:0005380	embryo phenotype	24	120	2087	17095	2.32×10^{-2}	*ALAS1, AMT, ARIH2, CACNA1S, CNNM2, CPT2, DAG1, EP300, EPO, ERBB3, FEN1, KCNJ13, L3MBTL2, MAGOH, NPRL2, PRMT1, RANGAP1, RASIP1, RHOA, SRRT, STAG1, TRAIP, WBP1L, XPNPEP1*
MP:0005378	growth/size/body region phenotype	54	90	5960	13222	5.99×10^{-2}	*ACHE, ALAS1, ARIH2, BCL2L12, CACNA1S, CDK2AP1, CELSR3, CNNM2, COL7A1, CYP17A1, DAG1, DENND1B, DUSP6, EP300, EPO, ERBB3, ESAM, FADS1, FADS2, FAM120A, FEN1, FOXP1, FOXP2, FUT2, GIGYF2, GPX1, IKZF4, IL2, IP6K1, IP6K2, KCNJ13, L3MBTL2, MAGOH, MST1R, NCK1, NPRL2, PCCB, PCLO, PHF2, QRICH1, RANGAP1, RASIP1, SEMA3F, SLC10A7, STAG1, TCF4, TFR2, TMEM258, TOX, TRAIP, TRIP6, TUSC2, USP19, XPNPEP1*
MP:0005385	cardiovascular system phenotype	35	109	3611	15571	6.17×10^{-2}	*ACHE, ALAS1, ARIH2, CACNA1S, CNNM2, CPT2, CRB1, CYP17A1, DAG1, DUSP6, EP300, EPO, ERBB3, ESAM, FAM120A, FOXP1, FOXP2, FUT2, GNB2, GPX1, IL2, IRF3, KCNJ13, NCK1, PRKCB, QRICH1, RASIP1, RRAS, SGIP1, STAG1, TFR2, TOX, TUSC2, WBP1L, XPNPEP1*
MP:0005397	hematopoietic system phenotype	44	100	4874	14308	9.57×10^{-2}	*ACHE, BCL2L12, COL7A1, DENND1B, DUSP6, EP300, EPO, ESAM, FADS1, FADS2, FEN1, FOXP1, FOXP2, GIGYF2, GPX1, IKZF4, IL2, IL21, IMPDH2, IP6K2, KLF7, MST1, MST1R, NPRL2, NT5C2, PDE4B, PRKCB, PSCA, QRICH1, RHOA, RRAS, SRRT, STAG1, TCF4, TFR2, TLR9, TOB2, TOX, TRIP6, TUSC2, USP19, USP4, WBP1L, XPNPEP1*
MP:0005376	homeostasis/metabolism phenotype	57	87	6561	12621	1.03×10^{-1}	*ACHE, ADAD1, AS3MT, C3orf84, CACNA1S, CELSR3, CNNM2, CPT2, CRB1, CYP17A1, DAG1, DUSP6, EP300, EPO, ERBB3, FADS1, FADS2, FAM120A, FEN1, FOXP1, FOXP2, GIGYF2, GNB2, GPX1, IL2, IL21, IP6K1, IP6K2, KIF21B, KLF7, MON1A, MPHOSPH9, MSRA, MST1R, NPRL2, OLFM4, P4HTM, PPP2R3A, PRKCB, PRMT1, RASIP1, RBM6, RPS26, RRAS, SEMA3F, SGIP1, SLC26A6, STAG1, TFR2, TMEM161B, TOX, TRIP6, TUSC2, UQCRC1, USP19, WBP1L, XPNPEP1*
MP:0005377	hearing/vestibular/ear phenotype	10	134	889	18293	1.34×10^{-1}	*ACHE, ACTL6B, BSN, CYP17A1, DUSP6, FAM120A, FOXP2, GPX1, RNF123, TOX*
MP:0005382	craniofacial phenotype	14	130	1347	17835	1.37×10^{-1}	*ACHE, CACNA1S, CDK2AP1, CNNM2, COL7A1, DUSP6, EP300, FOXP2, KCNJ13, QRICH1, SLC10A7, STAG1, TRIP6, XPNPEP1*
MP:0005388	respiratory system phenotype	15	129	1515	17667	1.67×10^{-1}	*ACHE, ARIH2, CELSR3, CPT2, DENND1B, DUSP6, EP300, EPO, ERBB3, FEN1, FOXP2, IL2, KCNJ13, KLF7, XPNPEP1*
MP:0010771	integument phenotype	20	124	2141	17041	1.82×10^{-1}	*ACHE, APEH, CPT2, CYP17A1, EP300, FADS2, FEN1, IL2, IMPDH2, MST1R, NCAM1, NCK1, PSCA, RANGAP1, RASIP1, SGIP1, STAG1, TRAIP, TRIP6, WDR6*

©2023 Gong W et al. *JAMA Psychiatry*.

MP:0005391	vision/eye phenotype	24	120	2687	16495	2.10×10^{-1}	*ACHE, ALAS1, BSN, CRB1, DAG1, EP300, FOXP2, GNB2, GPX1, IL2, KLF7, MON1A, MPHOSPH9, MYRF, NCAM1, NCK1, NPRL2, P4HTM, SEMA3F, SGIP1, STAG1, TCF4, WBP1L, XPNPEP1*
MP:0005389	reproductive system phenotype	25	119	2887	16295	2.51×10^{-1}	*ACHE, ACTL6B, ADAD1, C3orf62, CAMKV, CYP17A1, DPYD, FADS1, FADS2, FEN1, FUT1, FUT2, GIGYF2, GPX1, IP6K1, IZUMO1, LRP8, LY6K, MST1R, PRMT1, RBM5, RPS26, STAG1, TUSC2, USP19*
MP:0005369	muscle phenotype	15	129	1693	17489	2.91×10^{-1}	*ACHE, BSN, CACNA1S, DAG1, DUSP6, EP300, EPO, ERBB3, FOXP1, GPX1, MAMSTR, QRICH1, STAG1, USP19, XPNPEP1*
MP:0005370	liver/biliary system phenotype	14	130	1652	17530	3.59×10^{-1}	*ARIH2, AS3MT, BCL2L12, EPO, FADS2, FEN1, IL2, MST1, MST1R, NPRL2, NT5C2, STAG1, TFR2, XPNPEP1*
MP:0005367	renal/urinary system phenotype	15	129	1819	17363	3.92×10^{-1}	*ARIH2, EP300, FADS1, FADS2, FEN1, IKZF4, IRF3, NT5C2, P4HTM, PCCB, QRICH1, SLC26A6, STAG1, TUSC2, XPNPEP1*
MP:0001186	pigmentation phenotype	5	139	564	18618	4.19×10^{-1}	*CRB1, MAGOH, MYRF, SZT2, WDR6*
MP:0002006	neoplasm	7	137	839	18343	4.44×10^{-1}	*ESAM, FEN1, IP6K2, RRAS, STAG1, TUSC2, XPNPEP1*
MP:0005379	endocrine/exocrine gland phenotype	23	121	2933	16249	4.46×10^{-1}	*C3orf62, EP300, ERBB3, FADS1, FADS2, FEN1, FUT1, GIGYF2, GPX1, IL2, IP6K1, MST1R, PRMT1, QRICH1, RBM5, RPS26, SLC26A6, SRRT, TCF4, TMEM258, TUSC2, USP4, XPNPEP1*
MP:0005375	adipose tissue phenotype	10	134	1329	17853	5.44×10^{-1}	*ACHE, CACNA1S, CYP17A1, FAM120A, FEN1, FOXP2, PHF2, STAG1, TMEM161B, TUSC2*
MP:0005390	skeleton phenotype	18	126	2747	16435	7.67×10^{-1}	*BCL2L12, CACNA1S, CNNM2, COL7A1, CPT2, CYP17A1, DUSP6, FAM120A, KCNJ13, KLF7, L3MBTL2, SLC10A7, STAG1, TOB2, TOX, TRIP6, TUSC2, XPNPEP1*
MP:0005371	limbs/digits/tail phenotype	4	140	1144	18038	9.75×10^{-1}	*CACNA1S, COL7A1, SGIP1, SLC10A7*
MP:0005394	taste/olfaction phenotype	0	144	129	19053	1.00	
MP:0005381 or MP:0005386	digestive/alimentary phenotype or behavior/neurological phenotype	65	79	5894	13288	2.04×10^{-4}	*ACHE, ACTL6B, APEH, BSN, C3orf84, CACNA1S, CAMKV, CELSR3, CNNM2, COL7A1, CYP17A1, DAG1, DPYD, EP300, ERBB3, FADS1, FADS2, FAM120A, FEN1, FOXP1, FOXP2, FUT1, FUT2, GIGYF2, GNB2, GPX1, IKZF4, IL2, IP6K2, IRF3, KCNJ13, KIF21B, KLF7, LRP8, MON1A, MSRA, MST1, MYRF, NCAM1, NT5C2, OLFM4, PDE4B, PPP2R3A, PRKCB, QRICH1, RAB5B, RBM6, RPS26, RRAS, SEMA3F, SGIP1, SLC26A6, SZT2, TCF4, TEF, TFR2, TLR9, TMEM258, TOX, TUSC2, UQCRC1, USP19, WBP1L, WDR6, XPNPEP1*

©2023 Gong W et al. *JAMA Psychiatry*.

MP:0005381 and MP:0005386	digestive/alimentary phenotype and behavior/neurological phenotype	11	133	692	18490	1.80×10^{-2}	*CACNA1S, CAMKV, CELSR3, DPYD, ERBB3, FADS1, FADS2, IKZF4, IL2, KCNJ13, TOX*

Note: The phenotype annotations were obtained from "Mouse/Human Orthology with Phenotype Annotations" from Mouse Genome Informatics platform. In the pleiotropic gene group and non-pleiotropic gene group, 0 and 1 represent the number of genes associated with certain phenotype, respectively.

©2023 Gong W et al. *JAMA Psychiatry*.

eTable 11. Pleiotropic Genes Identified With Tissue Specificity in GTEx Reference Panel

Tissues	Num	*P*	Genes
Adrenal Gland	23	6.95×10^{-4}	*UFSP1, GMPPB, GPX1, KLHDC8B, PRKAR2A, TCTA, CPT2, PCCB, SLC35G2, OGFOD2, PITPNM2, THEM6, MAMSTR, FADS1, FADS2, NGEF, AS3MT, BORCS7, CYP17A1, ALAS1, IKZF4, RPS26, SUOX*
Stomach	23	6.95×10^{-4}	*INAVA, IRF3, TOX, GMPPB, MST1R, SLC26A6, TMEM161B, CPT2, VSIG2, OGFOD2, HYI, PSCA, THEM6, FAM83E, FUT1, FUT2, IZUMO1, MYRF, SLC10A7, FOXP2, FOXP1, ERBB3, SUOX*
Colon - Transverse	20	8.00×10^{-3}	*DENND1B, INAVA, TOX, AMT, GMPPB, MST1R, SLC26A6, UBA7, TMEM161B, OLFM4, CPT2, VSIG2, HYI, FAM83E, FUT2, SNORC, FOXP2, FOXP1, ERBB3, SUOX*
Small Intestine - Terminal Ileum	18	3.11×10^{-2}	*DENND1B, INAVA, IRF3, TOX, MST1R, SLC26A6, UBA7, OLFM4, CPT2, MSL2, OGFOD2, SZT2, IL2, FAM83E, FUT2, KCNJ13, TLR9, ERBB3*
Thyroid	17	5.64×10^{-2}	*GIGYF1, TRIP6, IRF3, PRR12, GMPPB, NICN1, TCTA, UBA7, TMEM161B, LRP8, ESAM, OGFOD2, SZT2, NTN5, SLC10A7, BORCS7, NT5C2*
Pituitary	16	9.60×10^{-2}	*ACTL6B, GIGYF1, PRR12, AMT, CELSR3, GMPPB, TCTA, SLC35G2, PHF2, MPHOSPH9, OGFOD2, SZT2, BLTP1, PCLO, NTN5, IKZF4*
Brain - Caudate (basal ganglia)	16	9.64×10^{-2}	*SGIP1, DYNLT5, CRB1, ACHE, ACTL6B, ZNF365, PRKCB, BSN, CAMKV, NCAM1, CHADL, CADM2, PCLO, MYRF, SNORC, NGEF*
Liver	16	9.64×10^{-2}	*EPO, TFR2, AMT, GPX1, MST1, RNF123, CPT2, PCCB, PITPNM2, HYI, THEM6, DUSP6, BORCS7, ALAS1, ERBB3, SUOX*
Lung	16	9.64×10^{-2}	*DYNLT5, CDHR4, GPX1, UBA7, TCF4, DPYD, ESAM, PITPNM2, IL2, FAM83E, FUT1, RASIP1, DUSP6, SLC10A7, KLF7, SEMA3F*
Uterus	16	9.64×10^{-2}	*GIGYF1, SRRT, TRIP6, PRR12, AMT, NICN1, NCAM1, MSL2, STAG1, PHF2, HYI, MAMSTR, NTN5, KLF7, FOXP2, WDR82*
Brain - Amygdala	15	1.56×10^{-1}	*SGIP1, DYNLT5, CRB1, KIF21B, ACTL6B, ZNF365, BSN, CAMKV, NCAM1, LRFN5, CHADL, CADM2, PCLO, MYRF, SNORC*
Brain - Hippocampus	15	1.56×10^{-1}	*DYNLT5, CRB1, KIF21B, ACTL6B, ZNF365, PRKCB, BSN, CAMKV, NCAM1, LRFN5, CHADL, CADM2, PCLO, MYRF, SNORC*
Brain - Putamen (basal ganglia)	15	1.56×10^{-1}	*SGIP1, CRB1, ACHE, ACTL6B, ZNF365, PRKCB, BSN, CAMKV, NCAM1, CHADL, CADM2, PCLO, MYRF, SNORC, NGEF*
Esophagus - Muscularis	15	1.56×10^{-1}	*TRIP6, UFSP1, RRAS, TOX, KLHDC8B, PHF2, PITPNM2, EP300, TOB2, ST3GAL3, KLF7, FOXP2, WDR82, FOXP1, IKZF4*
Muscle - Skeletal	15	1.56×10^{-1}	*CACNA1S, ACHE, UFSP1, IMPDH2, PRKAR2A, RNF123, CPT2, PCCB, PPP2R3A, TOB2, MAMSTR, ST3GAL3, PDE4B, GIGYF2, NT5C2*

©2023 Gong W et al. *JAMA Psychiatry*.

Pancreas	15	1.56×10^{-1}	*DENND1B, EPO, UFSP1, GMPPB, IMPDH2, OLFM4, OGFOD2, HYI, THEM6, FUT1, IZUMO1, MYRF, SLC10A7, ERBB3, RPS26*
Prostate	15	1.56×10^{-1}	*SRRT, TRIP6, IRF3, AMT, COL7A1, GMPPB, TCTA, UBA7, PHF2, OGFOD2, HYI, SZT2, NTN5, FOXP1, SUOX*
Vagina	15	1.56×10^{-1}	*DENND1B, INAVA, TRIP6, IRF3, AMT, COL7A1, INKA1, TMEM161B, OLFM4, LRFN5, PHF2, FUT2, SLC10A7, NT5C2, SEMA3F*
Brain - Cortex	14	2.36×10^{-1}	*CRB1, KIF21B, ACTL6B, ZNF365, PRKCB, BSN, CAMKV, CELSR3, NCAM1, LRFN5, CADM2, PCLO, SNORC, NGEF*
Brain - Hypothalamus	14	2.36×10^{-1}	*DYNLT5, CRB1, ACHE, ACTL6B, ZNF365, BSN, CAMKV, NCAM1, LRFN5, CHADL, CADM2, PCLO, MYRF, SNORC*
Brain - Nucleus accumbens (basal ganglia)	14	2.36×10^{-1}	*SGIP1, CRB1, ACHE, ACTL6B, ZNF365, PRKCB, BSN, CAMKV, NCAM1, LRFN5, CHADL, CADM2, PCLO, NGEF*
Brain - Substantia nigra	14	2.36×10^{-1}	*CRB1, ACHE, ACTL6B, ZNF365, BSN, CAMKV, NCAM1, LRFN5, CHADL, CADM2, PCLO, MYRF, PDE4B, SNORC*
Colon - Sigmoid	14	2.36×10^{-1}	*RRAS, TOX, AMT, KLHDC8B, NCAM1, PPP2R3A, PHF2, CHADL, HYI, KLF7, FOXP2, FOXP1, IKZF4, SUOX*
Esophagus - Gastroesophageal Junction	14	2.36×10^{-1}	*TRIP6, RRAS, TOX, KLHDC8B, PHF2, PITPNM2, TOB2, HYI, ST3GAL3, KLF7, FOXP2, WDR82, FOXP1, IKZF4*
Artery - Tibial	13	3.38×10^{-1}	*SGIP1, TRIP6, RRAS, TOX, PRKAR2A, SLC26A6, STAG1, ESAM, TOB2, HYI, BLTP1, KLF7, FOXP1*
Esophagus - Mucosa	13	3.38×10^{-1}	*DENND1B, INAVA, TRIP6, COL7A1, INKA1, IMPDH2, MST1R, THEM6, FUT2, SLC10A7, NT5C2, SEMA3F, ERBB3*
Heart - Left Ventricle	13	3.38×10^{-1}	*DYNLT5, DENND1B, KLHDC8B, NCAM1, CPT2, PCCB, PPP2R3A, ESAM, CHADL, RASIP1, AS3MT, ALAS1, SUOX*
Kidney - Cortex	13	3.38×10^{-1}	*EPO, TRIP6, IRF3, MST1, SLC26A6, TCTA, CPT2, PCCB, PPP2R3A, PITPNM2, HYI, THEM6, SUOX*
Ovary	13	3.38×10^{-1}	*GIGYF1, AMT, IMPDH2, KLHDC8B, NICN1, TMEM161B, PHF2, PITPNM2, HYI, BLTP1, NTN5, IKZF4, RPS26*
Brain - Anterior cingulate cortex (BA24)	12	4.57×10^{-1}	*CRB1, KIF21B, ACTL6B, ZNF365, PRKCB, BSN, CAMKV, NCAM1, LRFN5, CADM2, PCLO, NGEF*
Brain - Frontal Cortex (BA9)	12	4.57×10^{-1}	*CRB1, KIF21B, ACTL6B, ZNF365, PRKCB, BSN, CAMKV, NCAM1, LRFN5, CADM2, PCLO, NGEF*
Nerve - Tibial	12	4.57×10^{-1}	*GIGYF1, TRIP6, TOX, TMEM161B, MSL2, STAG1, PHF2, CHADL, BLTP1, SLC10A7, KLF7, ERBB3*
Spleen	12	4.57×10^{-1}	*KIF21B, GIGYF1, BCL2L12, IRF3, C3orf62, UBA7, MSL2, OGFOD2, RASIP1, DUSP6, TLR9, SEMA3F*
Artery - Coronary	11	5.82×10^{-1}	*SGIP1, TRIP6, RRAS, SLC26A6, TCTA, DPYD, ESAM, TOB2, HYI, DUSP6, FOXP1*
Adipose - Visceral (Omentum)	11	5.83×10^{-1}	*GPX1, TCF4, DPYD, SLC35G2, STAG1, ESAM, IL2, RASIP1, DUSP6, SLC10A7, KLF7*
Adipose - Subcutaneous	10	7.03×10^{-1}	*GPX1, TCF4, DPYD, SLC35G2, STAG1, ESAM, RASIP1, DUSP6, SLC10A7, KLF7*
Brain - Spinal cord (cervical c-1)	10	7.03×10^{-1}	*CRB1, ZNF365, CAMKV, NCAM1, CHADL, CADM2, PCLO, MYRF, PDE4B, SNORC*
Breast - Mammary Tissue	10	7.03×10^{-1}	*TCF4, SLC35G2, STAG1, ESAM, THEM6, NTN5, RASIP1, DUSP6, SLC10A7, KLF7*
Minor Salivary Gland	10	7.03×10^{-1}	*DENND1B, GMPPB, TMEM161B, THEM6, FAM83E, FUT2, DUSP6, SLC10A7, NT5C2, ERBB3*
Skin - Not Sun Exposed (Suprapubic)	10	7.03×10^{-1}	*INAVA, PRR12, COL7A1, INKA1, IMPDH2, MST1R, PPP2R3A, FADS2, SEMA3F, ERBB3*

©2023 Gong W et al. *JAMA Psychiatry*.

Skin - Sun Exposed (Lower leg)	10	7.03×10^{-1}	*INAVA, PRR12, COL7A1, INKA1, IMPDH2, MST1R, PPP2R3A, GIGYF2, SEMA3F, ERBB3*
Brain - Cerebellar Hemisphere	9	8.08×10^{-1}	*CRB1, ACHE, ACTL6B, ZNF365, BSN, CELSR3, CADM2, PCLO, WDR82*
Brain - Cerebellum	9	8.08×10^{-1}	*CRB1, ACHE, ACTL6B, PRR12, ZNF365, BSN, CELSR3, CADM2, PCLO*
Artery - Aorta	7	9.43×10^{-1}	*SGIP1, TRIP6, RRAS, NICN1, SLC26A6, HYI, FOXP1*
Heart - Atrial Appendage	7	9.43×10^{-1}	*KLHDC8B, NCAM1, PCCB, PPP2R3A, CHADL, PDE4B, AS3MT*
Whole Blood	7	9.43×10^{-1}	*KIF21B, SLC12A9, PRKCB, C3orf62, GPX1, DPYD, TLR9*
Testis	6	9.75×10^{-1}	*C3orf84, IHO1, TMEM89, ADAD1, IL21, IZUMO1*

eTable 12. Pleiotropic Genes Identified With Tissue Specificity in ENCODE Reference Panel

**Tissues**	**Num**	***P***	**Genes**
Stomach	20	6.26×10^{-3}	*DENND1B, INAVA, IRF3, TOX, AMT, MST1R, SLC26A6, UBA7, TMEM161B, VSIG2, MPHOSPH9, IL2, PSCA, THEM6, FAM83E, FUT1, FUT2, IZUMO1, MYRF, ERBB3*
Diencephalon	19	1.29×10^{-2}	*DYNLT5, KIF21B, ACHE, ACTL6B, ZNF365, BSN, CAMKV, NCAM1, LRFN5, SLC35G2, CADM2, BLTP1, PCLO, ST3GAL3, KLF7, PDE4B, NGEF, FOXP2, WDR82*
Adrenal Gland	19	1.30×10^{-2}	*UFSP1, GPX1, KLHDC8B, NICN1, PRKAR2A, TCTA, PCCB, PITPNM2, THEM6, MAMSTR, FADS1, FADS2, NGEF, AS3MT, CYP17A1, ALAS1, IKZF4, RPS26, SUOX*
Peyer's Patch	18	2.57×10^{-2}	*DENND1B, INAVA, AMT, MST1R, RNF123, SLC26A6, UBA7, OLFM4, HYI, SZT2, IL2, FAM83E, FUT2, NTN5, MYRF, KCNJ13, NGEF, ERBB3*
Liver	17	4.77×10^{-2}	*TFR2, AMT, GMPPB, MST1, RNF123, CPT2, DPYD, PCCB, MPHOSPH9, PITPNM2, THEM6, FADS1, FADS2, SLC10A7, ALAS1, ERBB3, SUOX*
Temporal Lobe	16	8.33×10^{-2}	*SGIP1, KIF21B, ACTL6B, ZNF365, BSN, CAMKV, CELSR3, NCAM1, LRFN5, TCF4, CADM2, PCLO, KLF7, PDE4B, NGEF, WDR82*
Body of Pancreas	15	1.37×10^{-1}	*GIGYF1, SLC12A9, UFSP1, IRF3, AMT, GMPPB, SLC26A6, TCTA, OLFM4, HYI, THEM6, FUT1, IZUMO1, RASIP1, MYRF*
Parietal Lobe	15	1.37×10^{-1}	*SGIP1, KIF21B, ACTL6B, ZNF365, BSN, CAMKV, CELSR3, NCAM1, TCF4, LRP8, CADM2, BLTP1, PCLO, KLF7, NGEF*
Heart Left Ventricle	14	1.91×10^{-1}	*RRAS, KLHDC8B, NCAM1, CPT2, PCCB, PPP2R3A, ESAM, PITPNM2, CHADL, TOB2, RASIP1, AS3MT, ALAS1, SUOX*
Cerebellum	14	2.13×10^{-1}	*SGIP1, ACTL6B, PRKCB, CELSR3, TMEM161B, LRFN5, TCF4, SLC35G2, MPHOSPH9, BLTP1, PCLO, KLF7, PDE4B, FOXP2*
Prostate Gland	14	2.13×10^{-1}	*GIGYF1, SRRT, UFSP1, BCL2L12, PRR12, GMPPB, TCTA, OGFOD2, CHADL, EP300, TOB2, HYI, NTN5, SUOX*
Suprapubic Skin	14	2.13×10^{-1}	*INAVA, BCL2L12, IRF3, PRR12, COL7A1, INKA1, MST1R, CHADL, SZT2, LY6K, FADS2, SEMA3F, ERBB3, RPS26*

©2023 Gong W et al. *JAMA Psychiatry*.

Spinal Cord	13	3.09×10^{-1}	*DYNLT5, ACHE, ZNF365, BSN, LRFN5, SLC35G2, CHADL, CADM2, BLTP1, PCLO, FADS1, PDE4B, WDR82*
Lung	13	3.10×10^{-1}	*DYNLT5, IMPDH2, TMEM161B, DPYD, ESAM, BLTP1, DUSP6, SLC10A7, PDE4B, GIGYF2, NT5C2, FOXP1, ERBB3*
Esophagus Squamous Epithelium	13	3.11×10^{-1}	*INAVA, TRIP6, UFSP1, BCL2L12, INKA1, GMPPB, IMPDH2, PSCA, THEM6, FUT2, IZUMO1, SEMA3F, RPS26*
Gastrocnemius Medialis	13	3.11×10^{-1}	*CACNA1S, ACHE, UFSP1, BCL2L12, IMPDH2, PRKAR2A, RNF123, TOB2, MAMSTR, ST3GAL3, FADS1, ALAS1, RPS26*
Occipital Lobe	13	3.11×10^{-1}	*SGIP1, DENND1B, KIF21B, ACTL6B, ZNF365, CAMKV, CELSR3, TMEM161B, NCAM1, LRFN5, TCF4, KLF7, WDR82*
Esophagus Muscularis Mucosa	12	4.24×10^{-1}	*UFSP1, RRAS, KLHDC8B, PHF2, PITPNM2, CHADL, TOB2, ST3GAL3, KLF7, FOXP2, FOXP1, IKZF4*
Gastroesophageal Sphincter	12	4.26×10^{-1}	*TRIP6, UFSP1, RRAS, TOX, KLHDC8B, PHF2, PITPNM2, TOB2, ST3GAL3, KLF7, FOXP2, FOXP1*
Right Atrium Auricular Region	12	4.27×10^{-1}	*ACHE, RRAS, KLHDC8B, NCAM1, PCCB, PPP2R3A, ESAM, CHADL, TOB2, ALAS1, RPS26, SUOX*
Sigmoid Colon	12	4.27×10^{-1}	*RRAS, TOX, GPX1, KLHDC8B, CHADL, HYI, IL2, FOXP2, FOXP1, IKZF4, RPS26, SUOX*
Skeletal Muscle Tissue	12	4.27×10^{-1}	*CACNA1S, ACHE, UFSP1, IHO1, IMPDH2, KLHDC8B, TCF4, PPP2R3A, STAG1, MAMSTR, NT5C2, ERBB3*
Right Lobe of Liver	11	5.51×10^{-1}	*EPO, TFR2, GMPPB, GPX1, MST1, CPT2, PCCB, THEM6, DUSP6, NGEF, SUOX*
Tibial Nerve	11	5.51×10^{-1}	*GIGYF1, UFSP1, RRAS, AMT, PHF2, CHADL, HYI, IL2, LY6K, NTN5, NGEF*
Ovary	11	5.52×10^{-1}	*BCL2L12, IHO1, CDHR4, IMPDH2, KLHDC8B, NICN1, HYI, NTN5, MYRF, IKZF4, RPS26*
Camera-type Eye	11	5.53×10^{-1}	*SGIP1, CRB1, DENND1B, TOX, TMEM161B, STAG1, MPHOSPH9, CADM2, GIGYF2, NT5C2, FOXP2*
Frontal Cortex	11	5.53×10^{-1}	*KIF21B, ACTL6B, ZNF365, CAMKV, CELSR3, NCAM1, LRFN5, TCF4, KLF7, NGEF, WDR82*
Upper Lobe of Left Lung	11	5.53×10^{-1}	*DYNLT5, PRR12, RRAS, CDHR4, UBA7, ESAM, PITPNM2, HYI, FAM83E, RASIP1, MYRF*
Thyroid Gland	10	6.76×10^{-1}	*UFSP1, GMPPB, TCTA, LRP8, ESAM, TOB2, SZT2, NT5C2, FOXP1, IKZF4*
Thoracic Aorta	10	6.78×10^{-1}	*SGIP1, RRAS, GMPPB, NICN1, SLC26A6, TMEM89, SZT2, NTN5, FOXP1, IKZF4*
Tongue	10	6.78×10^{-1}	*CACNA1S, ACHE, BCL2L12, IHO1, NICN1, RNF123, PPP2R3A, STAG1, NT5C2, ERBB3*
Transverse Colon	10	6.78×10^{-1}	*INAVA, MST1R, SLC26A6, TMEM89, VSIG2, FAM83E, FUT2, NGEF, ERBB3, RPS26*
Omental Fat Pad	9	7.85×10^{-1}	*UFSP1, RRAS, GPX1, SLC35G2, TOB2, IL2, RASIP1, DUSP6, MYRF*
Urinary Bladder	9	7.87×10^{-1}	*DENND1B, IMPDH2, STAG1, IL2, IL21, BLTP1, SLC10A7, NT5C2, FOXP2*
Heart	9	7.88×10^{-1}	*DYNLT5, DENND1B, TMEM161B, NCAM1, PPP2R3A, STAG1, MPHOSPH9, GIGYF2, NT5C2*
Lower Leg Skin	9	7.88×10^{-1}	*INAVA, IRF3, PRR12, COL7A1, INKA1, MST1R, FADS2, SEMA3F, ERBB3*
Metanephros	8	8.73×10^{-1}	*SLC12A9, UFSP1, BCL2L12, AMT, NICN1, PHF2, EP300, SZT2*

©2023 Gong W et al. *JAMA Psychiatry*.

Breast Epithelium	8	8.73×10^{-1}	*GPX1*, *DPYD*, *HYI*, *IL2*, *NTN5*, *PDE4B*, *ERBB3*, *RPS26*
Spleen	8	8.74×10^{-1}	*SLC12A9*, *PRKCB*, *C3orf62*, *UBA7*, *DPYD*, *MSL2*, *IL21*, *TLR9*
Skin of Body	3	9.33×10^{-1}	*IHO1*, *COL7A1*, *IMPDH2*, *TCF4*, *STAG1*, *IL2*, *SLC10A7*
Subcutaneous Adipose Tissue	3	9.34×10^{-1}	*RRAS*, *C3orf62*, *IHO1*, *GPX1*, *DPYD*, *SLC35G2*, *LY6K*
Testis	3	9.34×10^{-1}	*SRRT*, *C3orf84*, *IHO1*, *TMEM89*, *LRP8*, *ADAD1*, *LY6K*
Uterus	6	9.70×10^{-1}	*TRAIP*, *TMEM161B*, *STAG1*, *MPHOSPH9*, *OGFOD2*, *RASIP1*
Umbilical Cord	3	9.99×10^{-1}	*IRF3*, *STAG1*, *IL2*

©2023 Gong W et al. *JAMA Psychiatry*.

eTable 13. Twenty-Five Tissue Types Used for E-MAGMA Analysis

Tissue type	Code
Brain_Amygdala	E1
Brain_Anterior_cingulate_cortex_BA24	E2
Brain_Caudate_basal_ganglia	E3
Brain_Cerebellar_Hemisphere	E4
Brain_Cerebellum	E5
Brain_Cortex	E6
Brain_Frontal_Cortex_BA9	E7
Brain_Hippocampus	E8
Brain_Hypothalamus	E9
Brain_Nucleus_accumbens_basal_ganglia	E10
Brain_Putamen_basal_ganglia	E11
Brain_Spinal_cord_cervical_c-1	E12
Brain_Substantia_nigra	E13
Colon_Sigmoid	E14
Colon_Transverse	E15
Esophagus_Gastroesophageal_Junction	E16
Esophagus_Mucosa	E17
Esophagus_Muscularis	E18
Small_Intestine_Terminal_Ileum	E19
Stomach	E20
Adrenal_Gland	E21
Pituitary	E22
Liver	E23
Cells_EBV-transformed_lymphocytes	E24
Whole_Blood	E25

©2023 Gong W et al. *JAMA Psychiatry*.

eTable 14. Six Tissue/Cell Types Used for H-MAGMA Analysis

**Cell type**	**Code**
Adult brain	H1
Cortical Neuron	H2
Fetal brain	H3
iPSC derived astro	H4
iPSC derived neuro	H5
Midbrain DA	H6

©2023 Gong W et al. *JAMA Psychiatry*.

eTable 15. Tissue Specificity and Cell-Type Specificity of the Identified Pleiotropic Genes in E-MAGMA, JTI, and H-MAGMA Analysis

Trait pairs	No. loci	Locus	Gene symbol	E-MAGMA$_{PLACO}$	JTI$_{GIT}$	JTI$_{PSY}$	H-MAGMA $_{PLACO}$
IBD-MDD	1	1p31.3	*SGIP1*	E5, E12, E13, E15, E17, E19, E22, E25	E1, E5, E12, E14, E16, E17, E19–E21	E1, E4, E5, E12, E15–E17, E19–E22, E25	H1–H6
IBD-MDD	1	1p31.3	*DYNLT5*	E3–E11, E14–E20, E21, E22, E25	E13, E19	E2–E11, E13, E22	H2, H4, H6
IBD-MDD	3	1q31.3	*CRB1*	E6, E10	E4–E8, E11		H1, H2, H4, H5
IBD-MDD	3	1q31.3	*DENND1B*	E5, E16–E18, E20, E25	E4, E5, E8, E14, E16–E18, E20, E21		H1
IBD-MDD	4	1q32.1	*CACNA1S*				H1–H5
IBD-SCZ	9	1q32.1	*INAVA*	E14, E17, E25	E2–E4, E6–E11, E13, E14, E17	E17	H1, H2, H4, H6
IBD-SCZ	9	1q32.1	*CACNA1S*		E14		H1–H5
IBD-SCZ	9	1q32.1	*KIF21B*	E7, E16, E18	E4, E5, E15–E18, E21	E1, E16–E18	H1, H2, H6
IBD-SCZ	12	7q22.1	*ACHE*	E16–E18, E21–E23	E16, E18, E20, E22		H2
IBD-SCZ	12	7q22.1	*ACTL6B*				H2–H6
IBD-SCZ	12	7q22.1	*EPO*				H1, H4
IBD-SCZ	12	7q22.1	*GIGYF1*	E1, E3, E5–E22, E25	E1–E18, E20–E23, E25	E1–E25	H2–H6
IBD-SCZ	12	7q22.1	*GNB2*	E15, E18, E23	E12, E14–E16, E23	E23	H1–H6
IBD-SCZ	12	7q22.1	*POP7*		E23		H2, H4–H6
IBD-SCZ	12	7q22.1	*SLC12A9*	E1–E7, E10, E11, E15, E17, E22	E1–E12, E15, E17, E19, E20, E22, E23	E1–E12, E15, E16, E19, E20, E22, E23	H2, H4, H6
IBD-SCZ	12	7q22.1	*SRRT*	E5, E23	E5, E8, E9		H1–H3, H5
IBD-SCZ	12	7q22.1	*TFR2*	E10, E20	E10, E16, E18, E20	E10, E16, E18, E20	H1, H2, H5, H6
IBD-SCZ	12	7q22.1	*TRIP6*	E14–E18, E20	E5, E14–E16, E19, E20	E5, E14–E16, E18–E20	H2, H3
IBD-SCZ	12	7q22.1	*UFSP1*	E10, E14–E18, E22, E25			H1–H6
IBD-SCZ	13	19q13.33	*BCL2L12*				H1–H6
IBD-SCZ	13	19q13.33	*IRF3*	E4, E5, E7, E9, E10, E14, E15, E17–E22	E14–E19, E21, E24	E4–E7, E9–E11, E14–E24	H1–H6

©2023 Gong W et al. *JAMA Psychiatry*.

IBD-SCZ	13	19q13.33	*PRMT1*				H1, H3, H4
IBD-SCZ	13	19q13.33	*PRR12*	E2, E4–E7, E9, E11, E14–E23, E25	E12	E1–E21, E23–E25	H1–H6
IBD-SCZ	13	19q13.33	*RRAS*	E17	E2, E17		H2, H4–H6
IBD-SCZ	13	19q13.33	*SCAF1*	E17, E18	E8, E14–E18		H2, H3, H5
IBD-BIP	14	1q32.1	*INAVA*	E14, E17	E2–E4, E6–E11, E13, E14, E17	E17	H2, H4, H6
IBD-BIP	14	1q32.1	*KIF21B*	E7, E16, E18	E4, E5, E15–E18, E21		H1, H2, H6
IBD-BIP	16	8q12.1	*TOX*		E17		
IBD-BIP	17	10q21.2	*ZNF365*	E24	E12, E14, E18	E14, E18, E24	H1, H3–H6
IBD-BIP	18	16p12.2	*PRKCB*	E14–E16, E18, E21, E25	E21, E25	E21, E23	H1, H2, H4, H6
IBD-AN	19	1q32.1	*INAVA*	E14, E17, E25	E2–E4, E6–E11, E13, E14, E17		H2, H4, H6
IBD-AN	19	1q32.1	*KIF21B*	E6, E7, E16, E18	E4, E5, E15–E18, E21		H1, H2
IBD-AN	20	3p21.31	*AMT*	E5–E7, E10, E14–E18, E22, E25			H1–H6
IBD-AN	20	3p21.31	*APEH*	E12, E17, E18, E25	E8, E11, E12, E14–E16, E18, E19, E23–E25	E11, E12, E16, E23, E24	H1–H6
IBD-AN	20	3p21.31	*ARIH2*	E8, E15, E21	E3, E5–E9, E11, E14–E17, E19–E23, E25		H1–H6
IBD-AN	20	3p21.31	*BSN*	E6			H1–H6
IBD-AN	20	3p21.31	*C3orf62*	E5, E14, E17	E4, E6	E5, E11, E14, E16, E19, E21	H1–H6
IBD-AN	20	3p21.31	*C3orf84*				H1–H6
IBD-AN	20	3p21.31	*CAMKV*	E3, E19, E20	E4, E6, E7		H1–H6
IBD-AN	20	3p21.31	*IHO1*	E14–E18	E2, E3, E5, E7, E8, E10–E22		H1–H6
IBD-AN	20	3p21.31	*CDHR4*	E14, E17	E14	E15	H3–H6
IBD-AN	20	3p21.31	*CELSR3*		E15, E16, E18		H1–H6
IBD-AN	20	3p21.31	*COL7A1*		E7		H1, H3–H6

©2023 Gong W et al. *JAMA Psychiatry*.

IBD-AN	20	3p21.31	*DAG1*	E8, E17, E25	E2, E3, E6, E11, E14–E16, E18, E20, E22, E24, E25	E2, E3, E6, E8, E11, E14–E18, E20–E22, E24, E25	H1–H6
IBD-AN	20	3p21.31	*INKA1*	E4, E5, E17	E4, E5, E17		H4, H6
IBD-AN	20	3p21.31	*GMPPB*	E4–E6, E8, E11, E14–E18, E20, E23, E25	E21		H1–H6
IBD-AN	20	3p21.31	*GPX1*	E2–E8, E10, E17, E21			H1–H6
IBD-AN	20	3p21.31	*IMPDH2*		E7	E2, E4, E5, E17	H1–H6
IBD-AN	20	3p21.31	*IP6K1*	E17			H1–H6
IBD-AN	20	3p21.31	*IP6K2*	E4–E6, E8, E14–E21, E25			H1–H6
IBD-AN	20	3p21.31	*KLHDC8B*	E10, E17	E17		H1–H6
IBD-AN	20	3p21.31	*MON1A*	E22, E25	E18, E22		H2, H3, H5
IBD-AN	20	3p21.31	*MST1*	E1–E3, E5–E11, E14–E18, E20–E22, E25	E1, E8, E14, E20, E21	E1–E18, E20, E22	H1–H6
IBD-AN	20	3p21.31	*MST1R*	E2–E6, E10, E12, E14–E18, E25	E18, E19		H1, H2, H4, H6
IBD-AN	20	3p21.31	*NCKIPSD*	E2, E3, E5–E7, E10, E11, E14–E18, E20, E21, E25	E2, E4, E7, E10, E13–E17, E19–E21, E23–E25		H1–H6
IBD-AN	20	3p21.31	*NICN1*	E3, E14–E18	E5, E9, E15–E20, E22		H1–H6
IBD-AN	20	3p21.31	*NPRL2*				H1–H6
IBD-AN	20	3p21.31	*P4HTM*	E20			H1, H2–H5
IBD-AN	20	3p21.31	*PRKAR2A*	E18			H1–H6
IBD-AN	20	3p21.31	*EPRS1*				H1–H6
IBD-AN	20	3p21.31	*QRICH1*	E7, E22, E25			H1–H6
IBD-AN	20	3p21.31	*RBM5*	E16, E17	E16, E18, E19		H1, H2
IBD-AN	20	3p21.31	*RBM6*	E1–E25	E1–E25	E11	H2–H6
IBD-AN	20	3p21.31	*RHOA*				H1–H6
IBD-AN	20	3p21.31	*RNF123*	E1–E7, E9–E12, E14–E20, E22, E24	E14	E3, E9–E12, E14, E16–E18, E20, E22–E25	H1–H6

©2023 Gong W et al. *JAMA Psychiatry*.

IBD-AN	20	3p21.31	*SLC26A6*	E14, E16–E18, E22–E25	E18		H1–H6
IBD-AN	20	3p21.31	*TCTA*				H1–H6
IBD-AN	20	3p21.31	*TMEM89*	E25			H1–H6
IBD-AN	20	3p21.31	*TRAIP*				H2–H6
IBD-AN	20	3p21.31	*TUSC2*				H1–H6
IBD-AN	20	3p21.31	*UBA7*	E14, E15, E17–E20, E23, E25	E14–E20, E23, E25	E1, E3, E11	H1–H6
IBD-AN	20	3p21.31	*UQCRC1*				H1, H2, H3, H6
IBD-AN	20	3p21.31	*USP19*				H1–H6
IBD-AN	20	3p21.31	*USP4*		E1		H1–H6
IBD-AN	20	3p21.31	*WDR6*	E3, E5–E7, E14–E21, E23–E25	E1–E25	E4, E5	H1–H6
IBS-MDD	21	1q25.1	*COP1*				H6
IBS-MDD	22	5q14.3	*TMEM161B*	E5, E17	E4, E5		H1–H6
IBS-MDD	23	11q23.2	*NCAM1*		E23	E19	H1–H6
IBS-MDD	24	13q14.3	*OLFM4*		E19	E4–E6, E17–E20	H1, H4–H6
IBS-MDD	25	14q21.1	*LRFN5*	E4–E6, E18	E4–E6, E19		H1–H6
IBS-MDD	27	18q21.2	*TCF4*	E17	E17, E18		H1–H6
IBS-SCZ	28	1p32.3	*CZIB*	E1–E12, E14–E25	E1–E25	E1, E3–E7, E14–E16	H1, H2, H3, H5, H6
IBS-SCZ	28	1p32.3	*CPT2*				H1, H2–H4, H6
IBS-SCZ	28	1p32.3	*LRP8*	E4–E6, E16, E18	E1, E3–E7, E14, E16, E18		H1–H3
IBS-SCZ	28	1p32.3	*MAGOH*		E21	E15–E18, E20, E21, E23, E25	
IBS-SCZ	29	1p21.3	*DPYD*	E15, E17	E15	E1, E6, E13, E16, E23, E25	H1–H3, H5, H6
IBS-SCZ	31	3q22.3	*MSL2*		E25		H1, H2, H4, H6
IBS-SCZ	31	3q22.3	*NCK1*	E23	E2–E7, E9, E10, E22	E1–E22, E24, E25	H1–H3
IBS-SCZ	31	3q22.3	*PCCB*	E1–E11, E14–E25	E1–E6, E8–E11, E13, E14, E17–E19, E21, E22, E24, E25	E3, E6	H1–H6

©2023 Gong W et al. *JAMA Psychiatry*.

IBS-SCZ	31	3q22.3	*PPP2R3A*	E5, E17, E20, E23, E25	E5, E11, E17, E22, E25		H1–H6
IBS-SCZ	31	3q22.3	*SLC35G2*	E3	E1–E7, E10–E12, E25		H1–H5
IBS-SCZ	31	3q22.3	*STAG1*				H1–H6
IBS-SCZ	33	9q22.31	*FAM120A*	E18	E17, E19	E4, E5, E7, E15–E20	H1–H3
IBS-SCZ	33	9q22.31	*FAM120AOS*	E5, E14, E15, E17, E18	E4–E7, E15–E17, E19, E20		H1–H6
IBS-SCZ	33	9q22.31	*PHF2*		E6		H3
IBS-SCZ	34	11q24.2	*ESAM*		E3		H1–H6
IBS-SCZ	34	11q24.2	*VSIG2*	E6, E7, E10, E17, E18	E1–E3, E6–E11, E14–E23, E25		H4, H6
IBS-SCZ	36	12q24.31	*ARL6IP4*	E15, E17, E20, E21, E25	E15, E17–E23, E25		H1–H6
IBS-SCZ	36	12q24.31	*MTRFR*	E14–E18, E20	E4, E6, E13–E18, E20–E22		H1–H6
IBS-SCZ	36	12q24.31	*CDK2AP1*	E14–E20, E23, E25	E2, E3, E9, E14–E21, E23, E24		H1
IBS-SCZ	36	12q24.31	*MPHOSPH9*	E14–E16, E18, E19, E22, E25	E3, E5, E8–E10, E14–E16, E18–E20, E22, E25		H1, H2, H4–H6
IBS-SCZ	36	12q24.31	*OGFOD2*	E5, E15, E17, E18, E24, E25	E2, E4–E7, E9, E10, E12, E15, E17, E18, E22, E24, E25		H1–H6
IBS-SCZ	36	12q24.31	*PITPNM2*	E4, E5, E17	E4, E5, E8, E17		H1–H6
IBS-SCZ	36	12q24.31	*KMT5A*	E3–E6, E16–E18, E22, E25	E1–E8, E10–E12, E16–E18, E25	E3–E11, E14, E15, E18, E22	
IBS-SCZ	37	22q13.2	*CHADL*	E14			H1–H6
IBS-SCZ	37	22q13.2	*EP300*	E5, E9, E18, E25	E3–E11, E14, E15, E18, E22	E18	H1–H6
IBS-SCZ	37	22q13.2	*L3MBTL2*	E2, E7, E17, E18	E18	E5	H1–H6
IBS-SCZ	37	22q13.2	*LOC100996598*				
IBS-SCZ	37	22q13.2	*RANGAP1*	E4, E5, E17	E5, E17		H1–H6
IBS-SCZ	37	22q13.2	*TEF*	E25	E24, E25	E2–E7, E9–E11, E23	H1–H6
IBS-SCZ	37	22q13.2	*TOB2*				H3–H6
IBS-SCZ	37	22q13.2	*ZC3H7B*	E5, E17	E2–E7, E9–E11, E17		H1–H6

©2023 Gong W et al. *JAMA Psychiatry*.

IBS-ADHD	38	1p34.2	*HYI*				H1–H6
IBS-ADHD	38	1p34.2	*SZT2*	E4, E5, E14, E15, E17, E25	E4, E25	E4, E5	H1–H6
IBS-ADHD	39	5q14.3	*TMEM161B*	E5, E17, E22	E4, E5	E1–E25	H1–H6
IBS-BIP	40	1p32.3	*CZIB*	E1–E12, E14–E25	E1–E25	E1, E3, E6, E7, E14, E16	H1–H3, H5, H6
IBS-BIP	40	1p32.3	*CPT2*				H1–H4, H6
IBS-BIP	40	1p32.3	*LRP8*	E4–E6, E16, E18	E1, E3–E7, E14, E16, E18		H1, H3
IBS-BIP	40	1p32.3	*MAGOH*		E21	E4, E8, E9, E12	H1, H3, H4
IBS-BIP	42	3p12.1	*CADM2*	E3, E8–E13	E4–E6, E8, E9, E12, E13		H1, H6
IBS-BIP	43	4q27	*ADAD1*				H4
IBS-BIP	43	4q27	*IL2*				H1, H3, H5, H6
IBS-BIP	43	4q27	*IL21*				H2–H5
IBS-BIP	43	4q27	*BLTP1*	E3, E6, E16, E18	E16	E6, E10, E20	
IBS-BIP	44	7q21.11	*PCLO*	E10, E21	E6, E10, E20, E21		H1–H6
IBS-BIP	45	8p23.1	*MSRA*	E12, E17, E18	E8, E12, E13	E3–E5, E8, E17, E20, E21, E24, E25	H1–H6
IBS-BIP	46	10q25.1	*XPNPEP1*	E21, E22, E25	E20		H1, H2, H4–H6
IBS-BIP	47	11q23.2	*NCAM1*		E21, E23		H1–H3, H6
IBS-AN	49	9q22.31	*FAM120A*	E18	E19		H1–H3
IBS-AN	49	9q22.31	*FAM120AOS*	E5, E14, E15, E17, E18	E4–E7, E15–E17, E19, E20		H1–H6
IBS-AN	50	11q23.2	*NCAM1*				H1–H6
PUD-SCZ	52	8q24.3	*LY6K*	E1–E11, E13–E18, E20, E21	E1–E22		H1–H6
PUD-SCZ	52	8q24.3	*PSCA*	E1, E3–E11, E14–E20	E1–E23, E25		H1–H6
PUD-SCZ	52	8q24.3	*THEM6*	E1–E11, E14–E18, E20–E22, E24	E1–E13, E15–E18, E20–E22, E24	E23	H1, H3–H6
PUD-SCZ	54	19q13.33	*FAM83E*	E5, E6, E17	E1–E8, E10, E11, E23		H1–H6
PUD-SCZ	54	19q13.33	*FUT1*	E4, E15, E18, E22	E14, E16, E18, E22	E1–E3, E6–E11, E15, E17, E19, E20, E22	H1–H6

©2023 Gong W et al. *JAMA Psychiatry*.

PUD-SCZ	54	19q13.33	*FUT2*	E2, E3, E8, E10, E11, E15, E17, E19, E20, E22	E1–E3, E6–E11, E15, E17, E19–E22	E21	H1–H6
PUD-SCZ	54	19q13.33	*IZUMO1*	E4, E9, E14–E18, E20–E22	E14, E21	E1–E3, E5–E16, E18, E19, E21–E23	H1–H6
PUD-SCZ	54	19q13.33	*MAMSTR*	E1–E3, E5–E12, E14, E16, E18, E21	E1–E3, E5–E16, E18, E19, E21–E23	E1–E4, E6–E10, E12, E13, E19, E21–E24	H1–H6
PUD-SCZ	54	19q13.33	*NTN5*	E2–E7, E9–E22, E25	E1–E25	E6, E14–E18, E20, E21, E23, E25	H1–H3, H5, H6
PUD-SCZ	54	19q13.33	*RASIP1*	E9, E14–E18, E20	E6, E9, E14–E18, E21, E23, E25		H1–H5
PUD-ADHD	55	1p34.1	*ST3GAL3*	E6, E16, E18, E25	E6, E14, E16, E18, E21		H1–H6
PUD-ADHD	56	12q21.33	*DUSP6*		E15		H1–H6
PUD-ADHD	57	19q13.33	*FUT2*	E2, E3, E8, E10, E11, E15, E17, E19, E20, E22	E1–E3, E6–E11, E15, E17, E19–E22		H1–H6
PUD-ADHD	57	19q13.33	*MAMSTR*	E2, E3, E5–E12, E14, E16, E18, E21	E1–E3, E5–E16, E18, E19, E21–E23		H1–H6
PUD-BIP	60	8q24.3	*PSCA*	E1, E3–E11, E14–E20	E1–E23, E25		H1–H6
PUD-BIP	61	11q12.2	*FADS1*	E2–E9, E11, E12, E14, E16–E18, E20, E23, E25	E1–E18, E20–E23, E25		H1–H6
PUD-BIP	61	11q12.2	*FADS2*	E14–E20, E22, E25	E10, E14–E20, E22, E24, E25	E4, E21	H1–H4
PUD-BIP	61	11q12.2	*FEN1*		E21		H1–H6
PUD-BIP	61	11q12.2	*MYRF*	E17, E25	E16, E18, E23–E25		H6
PUD-BIP	61	11q12.2	*TMEM258*	E7, E11, E14, E16–E18, E25	E1, E3, E5–E7, E11, E12, E14–E20, E24, E25		H1, H3, H4, H6
PUD-AN	62	4q31.22	*SLC10A7*		E17		
PUD-AN	63	8q24.3	*PSCA*	E1, E3–E11, E14–E20	E1–E23, E25	E15, E19	H1–H6
GORD-MDD	64	2q33.3	*KLF7*		E19	E23	H1–H3, H4, H6
GORD-MDD	65	11q23.2	*NCAM1*	E18	E21, E23	E15–E18, E20, E21, E23, E25	H1, H2, H5, H6
GORD-SCZ	67	1p31.3	*PDE4B*				

©2023 Gong W et al. *JAMA Psychiatry*.

GORD-SCZ	68	1p21.3	*DPYD*	E17	E17		H1–H6
GORD-SCZ	70	2q37.1	*SNORC*	E1–E13	E1–E13	E5–E7, E9, E10, E19, E23	H1–H4
GORD-SCZ	70	2q37.1	*GIGYF2*	E6, E7, E14, E17, E18, E23	E23	E19	H1, H4–H6
GORD-SCZ	70	2q37.1	*KCNJ13*	E19	E19		H1–H6
GORD-SCZ	70	2q37.1	*NGEF*	E21, E14, E16–E18	E3, E8, E9, E11, E12, E14–E20	E1–E20, E22–E25	H1–H5
GORD-SCZ	74	10q24.32	*AS3MT*	E1–E19, E22, E25	E1–E20, E22–E25		H1–H6
GORD-SCZ	74	10q24.32	*BORCS7*	E1–E25	E1, E2, E4–E7, E9–E25		H1–H6
GORD-SCZ	74	10q24.32	*CNNM2*	E1, E5, E7, E25	E1–E8, E10, E11, E25	E6–E9, E18, E22	H1–H6
GORD-SCZ	74	10q24.32	*CYP17A1*	E6	E6, E18, E22		H1–H6
GORD-SCZ	74	10q24.32	*NT5C2*	E8, E18, E25	E11, E13, E19, E21, E24, E25		H1–H6
GORD-SCZ	74	10q24.32	*WBP1L*	E15, E17	E15, E20, E25		H1–H6
GORD-ADHD	76	3p21.31	*CAMKV*	E19, E20	E15, E19, E20		H1–H6
GORD-ADHD	76	3p21.31	*MON1A*	E18, E21	E18, E21, E25		H1, H2, H3, H5
GORD-ADHD	76	3p21.31	*MST1R*	E2, E4–E6, E10, E12, E15–E18, E25	E1–E18, E20, E22, E23, E25		H1–H6
GORD-ADHD	76	3p21.31	*RBM5*	E17	E6, E16–E19		H1–H6
GORD-ADHD	76	3p21.31	*RBM6*	E1–E25	E1–E25	E3, E22	H3–H6
GORD-ADHD	77	7q31.1	*FOXP2*	E21, E22	E3, E22		H1–H4, H6
GORD-BIP	78	3p21.2	*ALAS1*				H1–H6
GORD-BIP	78	3p21.2	*TLR9*		E3, E4, E6, E11, E14		H1–H6
GORD-BIP	78	3p21.2	*WDR82*		E8, E25	E4, E6, E7	H1, H2, H4, H5
GORD-AN	80	3p21.31	*CAMKV*	E3, E19, E20	E15, E19, E20	E14, E15	H1–H6

©2023 Gong W et al. *JAMA Psychiatry*.

GORD-AN	80	3p21.31	*CDHR4*	E14, E17	E14, E16, E18	E4, E5, E17	H1, H3–H6
GORD-AN	80	3p21.31	*INKA1*	E4, E5, E17	E4, E5, E17	E21	H4–H6
GORD-AN	80	3p21.31	*GMPPB*	E3–E9, E11, E14–E21, E23, E25	E21		H1–H6
GORD-AN	80	3p21.31	*IP6K1*	E17	E14, E17, E18, E21, E23	E18, E22, E25	H1–H6
GORD-AN	80	3p21.31	*MON1A*	E18, E21, E25	E18, E21	E1–E18, E20, E22, E23	H1–H6
GORD-AN	80	3p21.31	*MST1R*	E2–E6, E9, E10, E12, E14–E18, E25	E1–E18, E20, E22, E23, E25	E6, E16–E19	H1–H6
GORD-AN	80	3p21.31	*RBM5*	E17	E6, E16–E19	E1–E25	H1–H6
GORD-AN	80	3p21.31	*RBM6*	E1–E25	E1–E25	E1, E5, E11	H2–H6
GORD-AN	80	3p21.31	*RNF123*	E1–E7, E9–E12, E14–E20, E22, E24	E1–E13, E15–E25	E8, E18	H1–H6
GORD-AN	80	3p21.31	*SEMA3F*	E6, E8, E18	E6, E8, E13, E20	E14–E20, E23, E25	H1, H2, H5
GORD-AN	80	3p21.31	*TRAIP*				H2–H6
GORD-AN	80	3p21.31	*UBA7*	E14, E15, E17–E20, E23, E25	E14–E21, E23, E25		H1–H6
GORD-AN	81	3p13	*FOXP1*				H2–H5
GORD-AN	82	11q23.2	*NCAM1*		E21, E23		H1, H6
GORD-AN	83	12q13.2	*ERBB3*	E11	E8, E11	E22	H1–H6
GORD-AN	83	12q13.2	*IKZF4*				H2–H5
GORD-AN	83	12q13.2	*RAB5B*	E22	E22	E1–E25	H1–H6
GORD-AN	83	12q13.2	*RPS26*	E1–E25	E1–E25	E1–E6, E8–E16, E18–E25	H1–H6
GORD-AN	83	12q13.2	*SUOX*	E1–E23, E25	E1–E23, E25		H1–H6

©2023 Gong W et al. *JAMA Psychiatry*.

eTable 16. Significantly Enriched GO and KEGG Pathways in GSEA Analysis

Trait pair	Category	ID	Description	NES	*P* value	P_{adjust}
IBD–MDD	BP	GO:0043488	regulation of mRNA stability	2.1021	1.55×10^{-5}	1.93×10^{-2}
IBD–MDD	BP	GO:0001580	detection of chemical stimulus involved in sensory perception of bitter taste	3.0012	1.69×10^{-5}	1.93×10^{-2}
IBD–MDD	BP	GO:0061013	regulation of mRNA catabolic process	2.0508	2.39×10^{-5}	1.93×10^{-2}
IBD–MDD	BP	GO:0050913	sensory perception of bitter taste	2.9124	2.39×10^{-5}	1.93×10^{-2}
IBD–MDD	BP	GO:0000380	alternative mRNA splicing, via spliceosome	2.5708	2.78×10^{-5}	1.93×10^{-2}
IBD–MDD	BP	GO:0043487	regulation of RNA stability	2.0978	2.83×10^{-5}	1.93×10^{-2}
IBD–MDD	BP	GO:0050912	detection of chemical stimulus involved in sensory perception of taste	2.8463	2.83×10^{-5}	1.93×10^{-2}
IBD–MDD	BP	GO:0000381	regulation of alternative mRNA splicing, via spliceosome	2.6502	5.02×10^{-5}	2.68×10^{-2}
IBD–MDD	BP	GO:0016239	positive regulation of macroautophagy	2.5500	6.94×10^{-5}	3.37×10^{-2}
IBD–MDD	BP	GO:0090317	negative regulation of intracellular protein transport	2.7911	7.16×10^{-5}	3.37×10^{-2}
IBD–SCZ	BP	GO:0007156	homophilic cell adhesion via plasma membrane adhesion molecules	2.1921	4.09×10^{-7}	1.88×10^{-3}
IBD–SCZ	BP	GO:0098742	cell-cell adhesion via plasma-membrane adhesion molecules	2.0025	4.73×10^{-7}	1.88×10^{-3}
IBD–SCZ	BP	GO:0007259	receptor signaling pathway via JAK-STAT	2.0393	1.80×10^{-5}	1.98×10^{-2}
IBD–SCZ	BP	GO:0097696	receptor signaling pathway via STAT	2.0103	2.16×10^{-5}	1.98×10^{-2}
IBD–SCZ	BP	GO:0042501	serine phosphorylation of STAT protein	2.7906	3.28×10^{-5}	1.98×10^{-2}
IBD–SCZ	BP	GO:0034969	histone arginine methylation	2.9333	4.01×10^{-5}	1.98×10^{-2}
IBD–SCZ	BP	GO:0035246	peptidyl-arginine N-methylation	2.9319	4.13×10^{-5}	1.98×10^{-2}
IBD–SCZ	BP	GO:0033139	regulation of peptidyl-serine phosphorylation of STAT protein	2.7680	4.97×10^{-5}	1.98×10^{-2}
IBD–SCZ	BP	GO:0033141	positive regulation of peptidyl-serine phosphorylation of STAT protein	2.8185	6.04×10^{-5}	2.19×10^{-2}
IBD–SCZ	BP	GO:0046425	regulation of receptor signaling pathway via JAK-STAT	2.1696	8.63×10^{-5}	2.59×10^{-2}
IBD–SCZ	BP	GO:1904892	regulation of receptor signaling pathway via STAT	2.1171	1.33×10^{-4}	3.41×10^{-2}
IBD–SCZ	BP	GO:0018216	peptidyl-arginine methylation	2.8251	1.80×10^{-4}	4.49×10^{-2}
IBD–SCZ	BP	GO:0043330	response to exogenous dsRNA	2.4207	1.92×10^{-4}	4.63×10^{-2}
IBS–MDD	BP	GO:0007416	synapse assembly	2.0913	4.57×10^{-5}	3.27×10^{-2}

©2023 Gong W et al. *JAMA Psychiatry*.

Trait pair	Category	ID	Description	NES	*P* value	P_{adjust}
IBS–MDD	BP	GO:1905268	negative regulation of chromatin organization	2.6126	5.30×10^{-5}	3.27×10^{-2}
IBS–MDD	BP	GO:0043487	regulation of RNA stability	2.0059	6.49×10^{-5}	3.58×10^{-2}
IBS–MDD	BP	GO:0099054	presynapse assembly	2.7411	1.01×10^{-4}	4.25×10^{-2}
IBS–MDD	BP	GO:0043488	regulation of mRNA stability	2.0059	1.24×10^{-4}	4.50×10^{-2}
IBS–MDD	BP	GO:0099172	presynapse organization	2.6228	1.53×10^{-4}	4.92×10^{-2}
IBS–MDD	BP	GO:0000466	maturation of 5.8S rRNA from tricistronic rRNA transcript	2.7884	1.60×10^{-4}	4.92×10^{-2}
IBS–BIP	BP	GO:0099504	synaptic vesicle cycle	2.1183	1.19×10^{-6}	9.53×10^{-3}
IBS–BIP	BP	GO:0097479	synaptic vesicle localization	2.6173	4.94×10^{-6}	1.98×10^{-2}
IBS–BIP	BP	GO:0016079	synaptic vesicle exocytosis	2.3011	7.85×10^{-6}	2.10×10^{-2}
PUD–MDD	BP	GO:0048670	regulation of collateral sprouting	3.2503	2.14×10^{-5}	2.19×10^{-2}
PUD–MDD	BP	GO:0007416	synapse assembly	2.1474	5.36×10^{-5}	4.29×10^{-2}
GORD–MDD	BP	GO:0007416	synapse assembly	2.5777	8.59×10^{-9}	6.87×10^{-5}
GORD–MDD	BP	GO:0050807	regulation of synapse organization	2.2483	1.55×10^{-6}	2.22×10^{-3}
GORD–MDD	BP	GO:0050803	regulation of synapse structure or activity	2.1901	1.67×10^{-6}	2.22×10^{-3}
GORD–MDD	BP	GO:0007638	mechanosensory behavior	3.2861	6.12×10^{-6}	4.08×10^{-3}
GORD–MDD	BP	GO:0031223	auditory behavior	3.2762	9.24×10^{-6}	5.08×10^{-3}
GORD–MDD	BP	GO:0007156	homophilic cell adhesion via plasma membrane adhesion molecules	2.2105	9.52×10^{-6}	5.08×10^{-3}
GORD–MDD	BP	GO:0050919	negative chemotaxis	2.6625	9.19×10^{-5}	3.16×10^{-2}
GORD–MDD	BP	GO:0000380	alternative mRNA splicing, via spliceosome	2.5164	1.19×10^{-4}	3.40×10^{-2}
GORD–MDD	BP	GO:0021889	olfactory bulb interneuron differentiation	3.0371	1.24×10^{-4}	3.41×10^{-2}
GORD–MDD	BP	GO:0006376	mRNA splice site selection	2.7768	1.53×10^{-4}	3.95×10^{-2}
GORD–SCZ	BP	GO:0007156	homophilic cell adhesion via plasma membrane adhesion molecules	2.4280	1.39×10^{-9}	1.11×10^{-5}
GORD–SCZ	BP	GO:0021772	olfactory bulb development	2.7304	6.26×10^{-5}	4.89×10^{-2}
GORD–BIP	BP	GO:0007156	homophilic cell adhesion via plasma membrane adhesion molecules	2.4593	1.00×10^{-10}	8.01×10^{-7}
GORD–BIP	BP	GO:0098742	cell-cell adhesion via plasma-membrane adhesion molecules	2.0279	5.03×10^{-8}	2.02×10^{-4}

©2023 Gong W et al. *JAMA Psychiatry*.

Trait pair	Category	ID	Description	NES	*P* value	P_{adjust}
IBD–MDD	CC	GO:0098982	GABA-ergic synapse	2.7061	1.72×10^{-5}	1.93×10^{-2}
IBD–SCZ	CC	GO:0097346	INO80-type complex	2.7462	7.28×10^{-5}	2.32×10^{-2}
IBD–SCZ	CC	GO:0000932	P-body	2.1635	9.08×10^{-5}	2.59×10^{-2}
IBS–MDD	CC	GO:0014069	postsynaptic density	2.0472	4.22×10^{-7}	1.49×10^{-3}
IBS–MDD	CC	GO:0099572	postsynaptic specialization	2.0121	5.58×10^{-7}	1.49×10^{-3}
IBS–MDD	CC	GO:0032279	asymmetric synapse	2.0338	1.22×10^{-6}	1.96×10^{-3}
IBS–MDD	CC	GO:0098982	GABA-ergic synapse	2.7384	1.15×10^{-5}	1.31×10^{-2}
IBS–MDD	CC	GO:0099061	integral component of postsynaptic density membrane	2.6642	5.19×10^{-5}	3.27×10^{-2}
IBS–MDD	CC	GO:0099146	intrinsic component of postsynaptic density membrane	2.6021	8.52×10^{-5}	4.01×10^{-2}
IBS–MDD	CC	GO:0099634	postsynaptic specialization membrane	2.2470	9.08×10^{-5}	4.04×10^{-2}
IBS–MDD	CC	GO:0099060	integral component of postsynaptic specialization membrane	2.4435	1.39×10^{-4}	4.85×10^{-2}
IBS–MDD	CC	GO:0098839	postsynaptic density membrane	2.3766	1.60×10^{-4}	4.92×10^{-2}
IBS–BIP	CC	GO:0098831	presynaptic active zone cytoplasmic component	3.0289	1.91×10^{-5}	2.55×10^{-2}
PUD–MDD	CC	GO:0014069	postsynaptic density	2.0369	1.51×10^{-6}	6.47×10^{-3}
PUD–MDD	CC	GO:0032279	asymmetric synapse	2.0240	2.32×10^{-6}	6.47×10^{-3}
PUD–MDD	CC	GO:0098984	neuron to neuron synapse	2.0029	2.42×10^{-6}	6.47×10^{-3}
PUD–MDD	CC	GO:0099699	integral component of synaptic membrane	2.3153	1.10×10^{-5}	1.47×10^{-2}
PUD–MDD	CC	GO:0099240	intrinsic component of synaptic membrane	2.2502	2.19×10^{-5}	2.19×10^{-2}
PUD–MDD	CC	GO:0043197	dendritic spine	2.0887	4.41×10^{-5}	3.92×10^{-2}
PUD–MDD	CC	GO:0098936	intrinsic component of postsynaptic membrane	2.2955	6.37×10^{-5}	4.64×10^{-2}
PUD–MDD	CC	GO:0044309	neuron spine	2.0698	7.16×10^{-5}	4.78×10^{-2}
PUD–MDD	CC	GO:0099061	integral component of postsynaptic density membrane	2.6743	8.29×10^{-5}	4.90×10^{-2}
PUD–MDD	CC	GO:0099055	integral component of postsynaptic membrane	2.3150	9.19×10^{-5}	4.90×10^{-2}
PUD–SCZ	CC	GO:0043197	dendritic spine	2.0639	9.93×10^{-6}	1.59×10^{-2}
PUD–SCZ	CC	GO:0044309	neuron spine	2.0625	1.91×10^{-5}	2.31×10^{-2}

©2023 Gong W et al. *JAMA Psychiatry*.

Trait pair	Category	ID	Description	NES	*P* value	P_{adjust}
PUD–ADHD	CC	GO:0072686	mitotic spindle	2.2013	4.04×10^{-6}	3.22×10^{-2}
GORD–MDD	CC	GO:0098982	GABA-ergic synapse	3.0898	1.82×10^{-7}	6.04×10^{-4}
GORD–MDD	CC	GO:0099572	postsynaptic specialization	2.0036	1.22×10^{-6}	2.22×10^{-3}
GORD–MDD	CC	GO:0098936	intrinsic component of postsynaptic membrane	2.5325	2.22×10^{-6}	2.52×10^{-3}
GORD–MDD	CC	GO:0099055	integral component of postsynaptic membrane	2.5528	3.01×10^{-6}	2.52×10^{-3}
GORD–MDD	CC	GO:0099699	integral component of synaptic membrane	2.3021	1.47×10^{-5}	7.33×10^{-3}
GORD–MDD	CC	GO:0099240	intrinsic component of synaptic membrane	2.2564	1.69×10^{-5}	7.95×10^{-3}
GORD–MDD	CC	GO:0099146	intrinsic component of postsynaptic density membrane	2.7446	2.72×10^{-5}	1.15×10^{-2}
GORD–MDD	CC	GO:0098948	intrinsic component of postsynaptic specialization membrane	2.5157	3.96×10^{-5}	1.58×10^{-2}
GORD–MDD	CC	GO:0099061	integral component of postsynaptic density membrane	2.6930	5.30×10^{-5}	1.95×10^{-2}
GORD–MDD	CC	GO:0098831	presynaptic active zone cytoplasmic component	3.1856	5.36×10^{-5}	1.95×10^{-2}
GORD–MDD	CC	GO:0098839	postsynaptic density membrane	2.4043	9.87×10^{-5}	3.16×10^{-2}
GORD–MDD	CC	GO:0099634	postsynaptic specialization membrane	2.2644	1.01×10^{-4}	3.16×10^{-2}
GORD–MDD	CC	GO:0099738	cell cortex region	2.7170	1.05×10^{-4}	3.16×10^{-2}
GORD–MDD	CC	GO:0032589	neuron projection membrane	2.5697	1.51×10^{-4}	3.95×10^{-2}
GORD–MDD	CC	GO:0099060	integral component of postsynaptic specialization membrane	2.4695	1.73×10^{-4}	4.08×10^{-2}
IBD–MDD	MF	GO:0003730	mRNA 3'-UTR binding	2.9242	3.07×10^{-7}	2.46×10^{-3}
IBD–MDD	MF	GO:0050681	androgen receptor binding	3.0829	3.62×10^{-5}	2.23×10^{-2}
IBD–SCZ	MF	GO:0016273	arginine N-methyltransferase activity	2.9319	4.13×10^{-5}	1.98×10^{-2}
IBD–SCZ	MF	GO:0016274	protein-arginine N-methyltransferase activity	2.9319	4.13×10^{-5}	1.98×10^{-2}
IBS–MDD	MF	GO:0003730	mRNA 3'-UTR binding	2.8708	1.06×10^{-7}	8.52×10^{-4}
IBS–BIP	MF	GO:0050321	tau-protein kinase activity	2.8695	2.75×10^{-5}	3.15×10^{-2}
IBS–BIP	MF	GO:0048156	tau protein binding	2.4948	5.36×10^{-5}	4.29×10^{-2}
PUD–SCZ	MF	GO:0033130	acetylcholine receptor binding	3.0392	2.02×10^{-5}	2.31×10^{-2}
GORD–ADHD	MF	GO:0042826	histone deacetylase binding	2.4218	5.63×10^{-6}	4.49×10^{-2}

©2023 Gong W et al. *JAMA Psychiatry*.

Trait pair	Category	ID	Description	NES	*P* value	P_{adjust}
IBD–SCZ	KEGG	hsa04658	Th1 and Th2 cell differentiation	2.6775	4.51×10^{-8}	1.51×10^{-5}
IBD–SCZ	KEGG	hsa04659	Th17 cell differentiation	2.4316	2.46×10^{-6}	2.51×10^{-4}
IBD–SCZ	KEGG	hsa04660	T cell receptor signaling pathway	2.3493	2.91×10^{-6}	2.51×10^{-4}
IBD–SCZ	KEGG	hsa05235	PD-L1 expression and PD-1 checkpoint pathway in cancer	2.3878	3.01×10^{-6}	2.51×10^{-4}
IBD–SCZ	KEGG	hsa05161	Hepatitis B	2.0732	8.27×10^{-6}	4.76×10^{-4}
IBD–SCZ	KEGG	hsa04622	RIG-I-like receptor signaling pathway	2.4820	8.54×10^{-6}	4.76×10^{-4}
IBD–SCZ	KEGG	hsa05152	Tuberculosis	2.0908	1.44×10^{-5}	6.86×10^{-4}
IBD–SCZ	KEGG	hsa05418	Fluid shear stress and atherosclerosis	2.0273	1.08×10^{-4}	4.33×10^{-3}
IBD–SCZ	KEGG	hsa04620	Toll-like receptor signaling pathway	2.1333	1.17×10^{-4}	4.33×10^{-3}
IBD–SCZ	KEGG	hsa05140	Leishmaniasis	2.2352	5.91×10^{-4}	1.41×10^{-2}
IBD–SCZ	KEGG	hsa04930	Type II diabetes mellitus	2.2425	1.11×10^{-3}	2.17×10^{-2}
IBD–SCZ	KEGG	hsa05320	Autoimmune thyroid disease	2.3005	2.98×10^{-3}	3.61×10^{-2}
IBD–BIP	KEGG	hsa04659	Th17 cell differentiation	2.1633	3.00×10^{-5}	6.42×10^{-3}
IBD–BIP	KEGG	hsa04658	Th1 and Th2 cell differentiation	2.2273	3.84×10^{-5}	6.42×10^{-3}
IBD–AN	KEGG	hsa05145	Toxoplasmosis	2.2355	5.08×10^{-5}	9.03×10^{-3}
IBD–AN	KEGG	hsa04917	Prolactin signaling pathway	2.2255	1.42×10^{-4}	9.03×10^{-3}
IBD–AN	KEGG	hsa05140	Leishmaniasis	2.3478	1.83×10^{-4}	9.03×10^{-3}
IBD–AN	KEGG	hsa05412	Arrhythmogenic right ventricular cardiomyopathy	2.1600	1.89×10^{-4}	9.03×10^{-3}
IBD–AN	KEGG	hsa04659	Th17 cell differentiation	2.0502	3.30×10^{-4}	1.38×10^{-2}
IBD–AN	KEGG	hsa05321	Inflammatory bowel disease	2.3214	4.49×10^{-4}	1.50×10^{-2}
IBD–AN	KEGG	hsa05133	Pertussis	2.0350	9.48×10^{-4}	2.32×10^{-2}
PUD–SCZ	KEGG	hsa00601	Glycosphingolipid biosynthesis - lacto and neolacto series	2.5901	3.35×10^{-4}	3.34×10^{-2}
PUD–SCZ	KEGG	hsa05030	Cocaine addiction	2.3229	5.17×10^{-4}	3.34×10^{-2}
PUD–SCZ	KEGG	hsa04620	Toll-like receptor signaling pathway	2.0556	6.00×10^{-4}	3.34×10^{-2}
GORD–SCZ	KEGG	hsa05161	Hepatitis B	2.0102	1.49×10^{-5}	4.17×10^{-3}

©2023 Gong W et al. *JAMA Psychiatry*.

Trait pair	Category	ID	Description	NES	*P* value	P_{adjust}
GORD–SCZ	KEGG	hsa04622	RIG-I-like receptor signaling pathway	2.3956	2.50×10^{-5}	4.17×10^{-3}
GORD–SCZ	KEGG	hsa04623	Cytosolic DNA-sensing pathway	2.2261	2.12×10^{-4}	1.77×10^{-2}
GORD–SCZ	KEGG	hsa04620	Toll-like receptor signaling pathway	2.0443	2.76×10^{-4}	1.84×10^{-2}

Abbreviations: GSEA, gene set enrichment analysis; BP, biological process; CC, cellular component; MF, molecular function; KEGG, Kyoto Encyclopedia of Genes and Genomes; NES, normalized enrichment score.

©2023 Gong W et al. *JAMA Psychiatry*.

eTable 17. Significantly Colocalized Loci Identified by Multitrait Colocalization Using HyPrColoc

No.[a]	Trait pair	Top SNP	Region	Nearest gene	Microorganism	Feature	PP	PR	Candidate SNP	PP.SNP
9	IBD–SCZ	rs905634	1q32.1	*INAVA*	OTU99_558 (Bacteroidales)	Prevalence	0.8342	0.9326	rs905634[b]	0.8353
16	IBD–BIP	rs56073120	8q12.1	*TOX*	TestASV_29 (Barnesiella)	Abundance	0.8111	0.8713	rs56073120[b]	0.0646
22	IBS–MDD	rs3099439	5q14.3	*TMEM161B*	OTU97_101 (Sutterella)	Prevalence	0.7037	0.7984	rs3099439[b]	0.2918
22	IBS–MDD	rs3099439	5q14.3	*TMEM161B*	OTU99_116 (Sutterella)	Prevalence	0.7037	0.7984	rs3099439[b]	0.2918
26	IBS–MDD	rs1862743	16p12.2	*GNPATP*	OTU97_165 (Porphyromonadaceae)	Prevalence	0.7122	0.7261	rs1862743[b]	0.7098
28	IBS–SCZ	rs12031155	1p32.3	*LRP8*	C_Gammaproteobacteria	Abundance	0.8093	0.8515	rs17785382	0.3752
31	IBS–SCZ	rs1280622	3q22.3	*RP11-731C17.1*	TestASV_14 (Lachnospiraceae)	Prevalence	0.7429	0.8696	rs7432375	0.1306
34	IBS–SCZ	rs11604175	11q24.2	*VSIG2*	G_Ruminococcus2	Abundance	0.7116	0.7131	rs11604175[b]	0.5572
35	IBS–SCZ	rs12277680	11q25	*RP11-469N6.2*	OTU97_38 (Alphaproteobacteria)	Abundance	0.7817	0.7949	rs6590760	0.5667
35	IBS–SCZ	rs12277680	11q25	*RP11-469N6.2*	OTU99_39 (Alphaproteobacteria)	Abundance	0.7889	0.8030	rs6590760	0.5664
40	IBS–BIP	rs5177	1p32.3	*LRP8*	C_Gammaproteobacteria	Abundance	0.7389	0.7904	rs5177[b]	0.1784
54	PUD–SCZ	rs681343	19q13.33	*FUT2*	OTU97_106 (Ruminococcaceae)	Prevalence	0.7180	0.7792	rs681343[b]	0.2677
54	PUD–SCZ	rs681343	19q13.33	*FUT2*	OTU99_121 (Ruminococcaceae)	Prevalence	0.7708	0.8342	rs681343[b]	0.2671
54	PUD–SCZ	rs681343	19q13.33	*FUT2*	OTU97_27 (Bacteroides)	Abundance	0.8677	0.9638	rs681343[b]	0.3299
62	PUD–AN	rs9784437	4q31.22	*SLC10A7*	G_Roseburia	Abundance	0.7434	0.7749	rs9784437[b]	0.1572

Abbreviations: HyPrColoc, Hypothesis Prioritisation for multi-trait Colocalization; No., the numerical order of pleiotropic genomic risk locus; SNP, single nucleotide polymorphisms; PP, posterior probability; PR, posterior of region.

[a] The details of the these pleiotropic genomic risk locus were provided in eTable 7.

[b] The top SNP was also identified as candidate casual SNP in corresponding locus for a certain trait pair.

©2023 Gong W et al. *JAMA Psychiatry*.

eTable 18. Results of Bidirectional Mendelian Randomization Analysis From Main Analysis and Alternative Methods Between 4 Gastrointestinal Diseases and 6 Psychiatric Disorders

Exposure	Outcome	Method	IV num	PVE	*F* statistic	OR	OR.lower	OR.upper	*P* value	*FDR adjusted P*
IBD	MDD	Inverse-variance weighted	26	0.0026	45.6128	1.0007	0.9834	1.0183	9.35×10^{-1}	9.55×10^{-1}
MDD	IBD	Inverse-variance weighted	47	0.0035	37.5924	1.0583	0.8285	1.3520	6.50×10^{-1}	9.19×10^{-1}
IBD	PTSD	Inverse-variance weighted	26	0.0026	45.6128	1.0416	0.9902	1.0957	1.15×10^{-1}	2.75×10^{-1}
PTSD	IBD	Inverse-variance weighted	12	0.0048	28.1393	0.9018	0.7945	1.0235	1.10×10^{-1}	2.75×10^{-1}
IBD	SCZ	Inverse-variance weighted	24	0.0023	44.0766	0.9878	0.9351	1.0435	6.61×10^{-1}	9.19×10^{-1}
SCZ	IBD	Inverse-variance weighted	112	0.0463	45.5883	0.9971	0.9467	1.0502	9.14×10^{-1}	9.55×10^{-1}
IBD	ADHD	Inverse-variance weighted	25	0.0025	45.7931	1.0089	0.9563	1.0644	7.46×10^{-1}	9.42×10^{-1}
ADHD	IBD	Inverse-variance weighted	9	0.0138	35.4358	0.9878	0.8586	1.1363	8.63×10^{-1}	9.55×10^{-1}
IBD	BIP	Inverse-variance weighted	24	0.0024	46.2685	0.9787	0.9342	1.0254	3.65×10^{-1}	6.26×10^{-1}
BIP	IBD	Inverse-variance weighted	49	0.0050	42.0306	0.9889	0.9021	1.0842	8.13×10^{-1}	9.51×10^{-1}
IBD	AN	Inverse-variance weighted	26	0.0026	45.6128	0.9891	0.9458	1.0345	6.33×10^{-1}	9.19×10^{-1}
AN	IBD	Inverse-variance weighted	8	0.0040	36.5780	1.0872	0.9114	1.2969	3.53×10^{-1}	6.26×10^{-1}
IBS	MDD	Inverse-variance weighted	5	0.0003	32.8922	1.3522	1.1783	1.5517	1.73×10^{-5}	**2.77×10^{-4}**
MDD	IBS	Inverse-variance weighted	48	0.0036	37.7522	1.5193	1.3742	1.6797	3.13×10^{-16}	**1.50×10^{-14}**
IBS	PTSD	Inverse-variance weighted	6	0.0004	34.3919	1.7176	1.1580	2.5477	7.16×10^{-3}	**3.13×10^{-2}**
PTSD	IBS	Inverse-variance weighted	14	0.0054	27.3724	1.0284	0.9784	1.0810	2.70×10^{-1}	5.19×10^{-1}
IBS	SCZ	Inverse-variance weighted	6	0.0004	34.3919	1.1449	0.7685	1.7056	5.06×10^{-1}	8.09×10^{-1}
SCZ	IBS	Inverse-variance weighted	119	0.0485	45.0464	1.0301	1.0028	1.0582	3.04×10^{-2}	1.04×10^{-1}
IBS	ADHD	Inverse-variance weighted	5	0.0003	32.8922	1.4285	0.9929	2.0553	5.47×10^{-2}	1.54×10^{-1}
ADHD	IBS	Inverse-variance weighted	11	0.0171	36.0333	0.9497	0.8679	1.0392	2.61×10^{-1}	5.19×10^{-1}
IBS	BIP	Inverse-variance weighted	6	0.0004	34.3919	1.1879	0.7441	1.8965	4.71×10^{-1}	7.79×10^{-1}
BIP	IBS	Inverse-variance weighted	51	0.0051	41.6786	1.0004	0.9548	1.0482	9.85×10^{-1}	9.85×10^{-1}

©2023 Gong W et al. *JAMA Psychiatry*.

Exposure	Outcome	Method	IV num	PVE	*F* statistic	OR	OR.lower	OR.upper	*P* value	*FDR adjusted P*
IBS	AN	Inverse-variance weighted	5	0.0003	32.8922	1.3611	0.7200	2.5733	3.43×10^{-1}	6.26×10^{-1}
AN	IBS	Inverse-variance weighted	8	0.0040	36.5780	1.0077	0.9494	1.0696	8.00×10^{-1}	9.51×10^{-1}
PUD	MDD	Inverse-variance weighted	7	0.0007	47.2585	0.9848	0.9338	1.0387	5.74×10^{-1}	8.89×10^{-1}
MDD	PUD	Inverse-variance weighted	47	0.0035	37.5924	1.2698	1.1135	1.4480	3.65×10^{-4}	**2.92×10^{-3}**
PUD	PTSD	Inverse-variance weighted	7	0.0007	47.2585	1.0077	0.8743	1.1614	9.16×10^{-1}	9.55×10^{-1}
PTSD	PUD	Inverse-variance weighted	12	0.0048	28.1393	0.9834	0.8628	1.1208	8.02×10^{-1}	9.51×10^{-1}
PUD	SCZ	Inverse-variance weighted	7	0.0007	47.2585	1.0439	0.8553	1.2739	6.73×10^{-1}	9.19×10^{-1}
SCZ	PUD	Inverse-variance weighted	112	0.0463	45.5883	1.0248	0.9835	1.0679	2.43×10^{-1}	5.07×10^{-1}
PUD	ADHD	Inverse-variance weighted	7	0.0007	47.2585	0.8469	0.7297	0.9829	2.88×10^{-2}	1.04×10^{-1}
ADHD	PUD	Inverse-variance weighted	9	0.0138	35.4358	1.2031	1.0976	1.3188	7.85×10^{-5}	**9.42×10^{-4}**
PUD	BIP	Inverse-variance weighted	7	0.0007	47.2585	1.0075	0.9001	1.1277	8.97×10^{-1}	9.55×10^{-1}
BIP	PUD	Inverse-variance weighted	49	0.0050	42.0306	0.9556	0.8972	1.0178	1.58×10^{-1}	3.45×10^{-1}
PUD	AN	Inverse-variance weighted	7	0.0007	47.2585	0.8713	0.7620	0.9962	4.38×10^{-2}	1.31×10^{-1}
AN	PUD	Inverse-variance weighted	8	0.0040	36.5780	1.1889	1.0812	1.3073	3.55×10^{-4}	**2.92×10^{-3}**
GORD	MDD	Inverse-variance weighted	5	0.0004	33.0918	1.1787	1.0638	1.3061	1.68×10^{-3}	**8.09×10^{-3}**
MDD	GORD	Inverse-variance weighted	47	0.0035	37.5924	1.3721	1.2707	1.4815	6.52×10^{-16}	**1.56×10^{-14}**
GORD	PTSD	Inverse-variance weighted	5	0.0004	33.0918	1.3219	0.9240	1.8910	1.27×10^{-1}	2.89×10^{-1}
PTSD	GORD	Inverse-variance weighted	12	0.0048	28.1393	1.0985	1.0421	1.1579	4.73×10^{-4}	**3.24×10^{-3}**
GORD	SCZ	Inverse-variance weighted	5	0.0004	33.0918	1.0258	0.7449	1.4127	8.76×10^{-1}	9.55×10^{-1}
SCZ	GORD	Inverse-variance weighted	112	0.0463	45.5883	0.9955	0.9720	1.0195	7.08×10^{-1}	9.19×10^{-1}
GORD	ADHD	Inverse-variance weighted	5	0.0004	33.0918	1.7518	1.2737	2.4093	5.65×10^{-4}	**3.39×10^{-3}**
ADHD	GORD	Inverse-variance weighted	9	0.0138	35.4358	1.1096	1.0430	1.1804	9.96×10^{-4}	**5.31×10^{-3}**
GORD	BIP	Inverse-variance weighted	5	0.0004	33.0918	1.3485	1.0516	1.7291	1.84×10^{-2}	7.38×10^{-2}

©2023 Gong W et al. *JAMA Psychiatry*.

Exposure	Outcome	Method	IV num	PVE	*F* statistic	OR	OR.lower	OR.upper	*P* value	*FDR adjusted P*
BIP	GORD	Inverse-variance weighted	49	0.0050	42.0306	0.9576	0.9193	0.9976	3.78×10^{-2}	1.21×10^{-1}
GORD	AN	Inverse-variance weighted	5	0.0004	33.0918	1.3669	0.9878	1.8914	5.93×10^{-2}	1.58×10^{-1}
AN	GORD	Inverse-variance weighted	8	0.0040	36.5780	1.0118	0.9553	1.0717	6.89×10^{-1}	9.19×10^{-1}
IBD	MDD	MR-Egger	26	0.0026	45.6128	0.9532	0.9012	1.0083	9.44×10^{-2}	-
MDD	IBD	MR-Egger	47	0.0035	37.5924	0.5976	0.1028	3.4735	5.66×10^{-1}	-
IBD	PTSD	MR-Egger	26	0.0026	45.6128	0.9852	0.8298	1.1698	8.65×10^{-1}	-
PTSD	IBD	MR-Egger	12	0.0048	28.1393	0.7645	0.3458	1.6900	5.07×10^{-1}	-
IBD	SCZ	MR-Egger	24	0.0023	44.0766	1.0876	0.9000	1.3142	3.85×10^{-1}	-
SCZ	IBD	MR-Egger	112	0.0463	45.5883	1.0043	0.7819	1.2900	9.73×10^{-1}	-
IBD	ADHD	MR-Egger	25	0.0025	45.7931	1.0368	0.8618	1.2475	7.01×10^{-1}	-
ADHD	IBD	MR-Egger	9	0.0138	35.4358	1.0162	0.5246	1.9687	9.62×10^{-1}	-
IBD	BIP	MR-Egger	24	0.0024	46.2685	0.9293	0.7952	1.0861	3.57×10^{-1}	-
BIP	IBD	MR-Egger	49	0.0050	42.0306	0.7477	0.4443	1.2582	2.74×10^{-1}	-
IBD	AN	MR-Egger	26	0.0026	45.6128	0.9593	0.8242	1.1165	5.91×10^{-1}	-
AN	IBD	MR-Egger	8	0.0040	36.5780	1.2779	0.6119	2.6688	5.14×10^{-1}	-
IBS	MDD	MR-Egger	5	0.0003	32.8922	0.2440	0.0413	1.4413	1.20×10^{-1}	-
MDD	IBS	MR-Egger	48	0.0036	37.7522	0.6394	0.3217	1.2705	2.02×10^{-1}	-
IBS	PTSD	MR-Egger	6	0.0004	34.3919	0.0428	0.0008	2.3347	1.22×10^{-1}	-
PTSD	IBS	MR-Egger	14	0.0054	27.3724	1.0999	0.7941	1.5236	5.67×10^{-1}	-
IBS	SCZ	MR-Egger	6	0.0004	34.3919	0.5671	0.0025	127.8142	8.37×10^{-1}	-
SCZ	IBS	MR-Egger	119	0.0485	45.0464	1.0552	0.9259	1.2026	4.20×10^{-1}	-
IBS	ADHD	MR-Egger	5	0.0003	32.8922	9.4161	0.0125	7098.1187	5.07×10^{-1}	-
ADHD	IBS	MR-Egger	11	0.0171	36.0333	0.7848	0.4878	1.2624	3.18×10^{-1}	-

©2023 Gong W et al. *JAMA Psychiatry*.

Exposure	Outcome	Method	IV num	PVE	*F* statistic	OR	OR.lower	OR.upper	*P* value	*FDR adjusted P*
IBS	BIP	MR-Egger	6	0.0004	34.3919	0.0047	0.0001	0.1603	2.90×10^{-3}	-
BIP	IBS	MR-Egger	51	0.0051	41.6786	0.7844	0.6064	1.0146	6.44×10^{-2}	-
IBS	AN	MR-Egger	5	0.0003	32.8922	0.0090	0.0000	400.2670	3.88×10^{-1}	-
AN	IBS	MR-Egger	8	0.0040	36.5780	0.9725	0.7577	1.2482	8.27×10^{-1}	-
PUD	MDD	MR-Egger	7	0.0007	47.2585	0.6856	0.5264	0.8931	5.13×10^{-3}	-
MDD	PUD	MR-Egger	47	0.0035	37.5924	0.9782	0.3797	2.5197	9.64×10^{-1}	-
PUD	PTSD	MR-Egger	7	0.0007	47.2585	0.6037	0.2459	1.4824	2.71×10^{-1}	-
PTSD	PUD	MR-Egger	12	0.0048	28.1393	0.7508	0.3230	1.7450	5.05×10^{-1}	-
PUD	SCZ	MR-Egger	7	0.0007	47.2585	1.0155	0.2562	4.0261	9.83×10^{-1}	-
SCZ	PUD	MR-Egger	112	0.0463	45.5883	0.9740	0.7985	1.1882	7.95×10^{-1}	-
PUD	ADHD	MR-Egger	7	0.0007	47.2585	1.4822	0.6054	3.6287	3.89×10^{-1}	-
ADHD	PUD	MR-Egger	9	0.0138	35.4358	0.9178	0.5946	1.4165	6.98×10^{-1}	-
PUD	BIP	MR-Egger	7	0.0007	47.2585	0.9797	0.4443	2.1602	9.60×10^{-1}	-
BIP	PUD	MR-Egger	49	0.0050	42.0306	0.9798	0.6821	1.4075	9.12×10^{-1}	-
PUD	AN	MR-Egger	7	0.0007	47.2585	0.4851	0.1991	1.1822	1.11×10^{-1}	-
AN	PUD	MR-Egger	8	0.0040	36.5780	1.2932	0.8897	1.8798	1.78×10^{-1}	-
GORD	MDD	MR-Egger	5	0.0004	33.0918	1.1189	0.5774	2.1681	7.39×10^{-1}	-
MDD	GORD	MR-Egger	47	0.0035	37.5924	0.9807	0.5684	1.6920	9.44×10^{-1}	-
GORD	PTSD	MR-Egger	5	0.0004	33.0918	0.2674	0.0275	2.5964	2.55×10^{-1}	-
PTSD	GORD	MR-Egger	12	0.0048	28.1393	0.9878	0.7035	1.3869	9.43×10^{-1}	-
GORD	SCZ	MR-Egger	5	0.0004	33.0918	2.8295	0.3586	22.3285	3.24×10^{-1}	-
SCZ	GORD	MR-Egger	112	0.0463	45.5883	1.0046	0.8951	1.1274	9.38×10^{-1}	-
GORD	ADHD	MR-Egger	5	0.0004	33.0918	2.2894	0.2493	21.0269	4.64×10^{-1}	-

©2023 Gong W et al. *JAMA Psychiatry*.

Exposure	Outcome	Method	IV num	PVE	*F* statistic	OR	OR.lower	OR.upper	*P* value	*FDR adjusted P*
ADHD	GORD	MR-Egger	9	0.0138	35.4358	0.9104	0.6918	1.1980	5.03×10^{-1}	-
GORD	BIP	MR-Egger	5	0.0004	33.0918	1.8366	0.2961	11.3925	5.14×10^{-1}	-
BIP	GORD	MR-Egger	49	0.0050	42.0306	0.9966	0.7881	1.2602	9.77×10^{-1}	-
GORD	AN	MR-Egger	5	0.0004	33.0918	4.8684	0.5900	40.1688	1.42×10^{-1}	-
AN	GORD	MR-Egger	8	0.0040	36.5780	1.0220	0.7992	1.3069	8.62×10^{-1}	-
IBD	MDD	MR-Egger intercept	26	0.0026	45.6128	1.0066	0.9993	1.0139	7.52×10^{-2}	-
MDD	IBD	MR-Egger intercept	47	0.0035	37.5924	1.0170	0.9660	1.0708	5.20×10^{-1}	-
IBD	PTSD	MR-Egger intercept	26	0.0026	45.6128	1.0075	0.9855	1.0301	5.06×10^{-1}	-
PTSD	IBD	MR-Egger intercept	12	0.0048	28.1393	1.0165	0.9405	1.0988	6.79×10^{-1}	-
IBD	SCZ	MR-Egger intercept	24	0.0023	44.0766	0.9874	0.9641	1.0113	2.98×10^{-1}	-
SCZ	IBD	MR-Egger intercept	112	0.0463	45.5883	0.9995	0.9815	1.0178	9.54×10^{-1}	-
IBD	ADHD	MR-Egger intercept	25	0.0025	45.7931	0.9963	0.9728	1.0204	7.62×10^{-1}	-
ADHD	IBD	MR-Egger intercept	9	0.0138	35.4358	0.9974	0.9394	1.0589	9.31×10^{-1}	-
IBD	BIP	MR-Egger intercept	24	0.0024	46.2685	1.0071	0.9868	1.0278	4.95×10^{-1}	-
BIP	IBD	MR-Egger intercept	49	0.0050	42.0306	1.0190	0.9845	1.0548	2.85×10^{-1}	-
IBD	AN	MR-Egger intercept	26	0.0026	45.6128	1.0041	0.9847	1.0239	6.79×10^{-1}	-
AN	IBD	MR-Egger intercept	8	0.0040	36.5780	0.9848	0.9203	1.0537	6.56×10^{-1}	-
IBS	MDD	MR-Egger intercept	5	0.0003	32.8922	1.0714	0.9976	1.1507	5.84×10^{-2}	-
MDD	IBS	MR-Egger intercept	48	0.0036	37.7522	1.0259	1.0055	1.0467	1.26×10^{-2}	-
IBS	PTSD	MR-Egger intercept	6	0.0004	34.3919	1.1651	0.9879	1.3740	6.94×10^{-2}	-
PTSD	IBS	MR-Egger intercept	14	0.0054	27.3724	0.9935	0.9627	1.0252	6.83×10^{-1}	-
IBS	SCZ	MR-Egger intercept	6	0.0004	34.3919	1.0295	0.8234	1.2872	7.99×10^{-1}	-
SCZ	IBS	MR-Egger intercept	119	0.0485	45.0464	0.9982	0.9889	1.0077	7.12×10^{-1}	-

©2023 Gong W et al. *JAMA Psychiatry*.

Exposure	Outcome	Method	IV num	PVE	*F* statistic	OR	OR.lower	OR.upper	*P* value	*FDR adjusted P*
IBS	ADHD	MR-Egger intercept	5	0.0003	32.8922	0.9268	0.7100	1.2098	5.76×10^{-1}	-
ADHD	IBS	MR-Egger intercept	11	0.0171	36.0333	1.0177	0.9749	1.0624	4.23×10^{-1}	-
IBS	BIP	MR-Egger intercept	6	0.0004	34.3919	1.2564	1.0868	1.4525	2.04×10^{-3}	-
BIP	IBS	MR-Egger intercept	51	0.0051	41.6786	1.0164	0.9993	1.0338	5.97×10^{-2}	-
IBS	AN	MR-Egger intercept	5	0.0003	32.8922	1.2245	0.7957	1.8843	3.57×10^{-1}	-
AN	IBS	MR-Egger intercept	8	0.0040	36.5780	1.0034	0.9805	1.0269	7.73×10^{-1}	-
PUD	MDD	MR-Egger intercept	7	0.0007	47.2585	1.0315	1.0087	1.0549	6.54×10^{-3}	-
MDD	PUD	MR-Egger intercept	47	0.0035	37.5924	1.0077	0.9802	1.0360	5.85×10^{-1}	-
PUD	PTSD	MR-Egger intercept	7	0.0007	47.2585	1.0449	0.9684	1.1276	2.58×10^{-1}	-
PTSD	PUD	MR-Egger intercept	12	0.0048	28.1393	1.0272	0.9457	1.1157	5.25×10^{-1}	-
PUD	SCZ	MR-Egger intercept	7	0.0007	47.2585	1.0024	0.8917	1.1268	9.68×10^{-1}	-
SCZ	PUD	MR-Egger intercept	112	0.0463	45.5883	1.0038	0.9894	1.0184	6.08×10^{-1}	-
PUD	ADHD	MR-Egger intercept	7	0.0007	47.2585	0.9529	0.8831	1.0283	2.15×10^{-1}	-
ADHD	PUD	MR-Egger intercept	9	0.0138	35.4358	1.0254	0.9859	1.0665	2.11×10^{-1}	-
PUD	BIP	MR-Egger intercept	7	0.0007	47.2585	1.0024	0.9375	1.0718	9.44×10^{-1}	-
BIP	PUD	MR-Egger intercept	49	0.0050	42.0306	0.9983	0.9747	1.0226	8.91×10^{-1}	-
PUD	AN	MR-Egger intercept	7	0.0007	47.2585	1.0511	0.9752	1.1329	1.92×10^{-1}	-
AN	PUD	MR-Egger intercept	8	0.0040	36.5780	0.9920	0.9585	1.0267	6.49×10^{-1}	-
GORD	MDD	MR-Egger intercept	5	0.0004	33.0918	1.0023	0.9738	1.0316	8.76×10^{-1}	-
MDD	GORD	MR-Egger intercept	47	0.0035	37.5924	1.0100	0.9940	1.0262	2.23×10^{-1}	-
GORD	PTSD	MR-Egger intercept	5	0.0004	33.0918	1.0731	0.9718	1.1848	1.63×10^{-1}	-
PTSD	GORD	MR-Egger intercept	12	0.0048	28.1393	1.0106	0.9775	1.0448	5.34×10^{-1}	-
GORD	SCZ	MR-Egger intercept	5	0.0004	33.0918	0.9561	0.8736	1.0464	3.30×10^{-1}	-

©2023 Gong W et al. *JAMA Psychiatry*.

Exposure	Outcome	Method	IV num	PVE	*F* statistic	OR	OR.lower	OR.upper	*P* value	*FDR adjusted P*
SCZ	GORD	MR-Egger intercept	112	0.0463	45.5883	0.9993	0.9910	1.0077	8.74×10^{-1}	-
GORD	ADHD	MR-Egger intercept	5	0.0004	33.0918	0.9882	0.8969	1.0888	8.11×10^{-1}	-
ADHD	GORD	MR-Egger intercept	9	0.0138	35.4358	1.0185	0.9935	1.0441	1.48×10^{-1}	-
GORD	BIP	MR-Egger intercept	5	0.0004	33.0918	0.9865	0.9111	1.0680	7.37×10^{-1}	-
BIP	GORD	MR-Egger intercept	49	0.0050	42.0306	0.9973	0.9819	1.0129	7.35×10^{-1}	-
GORD	AN	MR-Egger intercept	5	0.0004	33.0918	0.9456	0.8625	1.0366	2.32×10^{-1}	-
AN	GORD	MR-Egger intercept	8	0.0040	36.5780	0.9991	0.9768	1.0218	9.34×10^{-1}	-
IBD	MDD	MR-Robust	26	0.0026	45.6128	1.0006	0.9820	1.0196	9.50×10^{-1}	-
MDD	IBD	MR-Robust	47	0.0035	37.5924	1.0639	0.8156	1.3878	6.48×10^{-1}	-
IBD	PTSD	MR-Robust	26	0.0026	45.6128	1.0666	1.0052	1.1318	3.29×10^{-2}	-
PTSD	IBD	MR-Robust	12	0.0048	28.1393	0.9011	0.8046	1.0092	7.16×10^{-2}	-
IBD	SCZ	MR-Robust	24	0.0023	44.0766	0.9815	0.9373	1.0277	4.26×10^{-1}	-
SCZ	IBD	MR-Robust	112	0.0463	45.5883	0.9984	0.9471	1.0525	9.53×10^{-1}	-
IBD	ADHD	MR-Robust	25	0.0025	45.7931	1.0143	0.9687	1.0620	5.45×10^{-1}	-
ADHD	IBD	MR-Robust	9	0.0138	35.4358	0.9916	0.8590	1.1447	9.08×10^{-1}	-
IBD	BIP	MR-Robust	24	0.0024	46.2685	0.9720	0.9271	1.0190	2.38×10^{-1}	-
BIP	IBD	MR-Robust	49	0.0050	42.0306	0.9835	0.8992	1.0757	7.16×10^{-1}	-
IBD	AN	MR-Robust	26	0.0026	45.6128	0.9819	0.9450	1.0203	3.51×10^{-1}	-
AN	IBD	MR-Robust	8	0.0040	36.5780	1.1065	0.8666	1.4129	4.17×10^{-1}	-
IBS	MDD	MR-Robust	5	0.0003	32.8922	1.3557	1.1824	1.5542	1.29×10^{-5}	-
MDD	IBS	MR-Robust	48	0.0036	37.7522	1.5085	1.3631	1.6693	1.84×10^{-15}	-
IBS	PTSD	MR-Robust	6	0.0004	34.3919	1.7186	1.1617	2.5425	6.73×10^{-3}	-
PTSD	IBS	MR-Robust	14	0.0054	27.3724	1.0332	0.9729	1.0973	2.87×10^{-1}	-

©2023 Gong W et al. *JAMA Psychiatry*.

Exposure	Outcome	Method	IV num	PVE	*F* statistic	OR	OR.lower	OR.upper	*P* value	*FDR adjusted P*
IBS	SCZ	MR-Robust	6	0.0004	34.3919	1.1365	0.7612	1.6969	5.31×10^{-1}	-
SCZ	IBS	MR-Robust	119	0.0485	45.0464	1.0326	1.0025	1.0635	3.36×10^{-2}	-
IBS	ADHD	MR-Robust	5	0.0003	32.8922	1.4321	1.0240	2.0030	3.59×10^{-2}	-
ADHD	IBS	MR-Robust	11	0.0171	36.0333	0.9524	0.8724	1.0398	2.76×10^{-1}	-
IBS	BIP	MR-Robust	6	0.0004	34.3919	1.1910	0.7522	1.8859	4.56×10^{-1}	-
BIP	IBS	MR-Robust	51	0.0051	41.6786	0.9965	0.9531	1.0418	8.77×10^{-1}	-
IBS	AN	MR-Robust	5	0.0003	32.8922	1.3432	0.7488	2.4094	3.22×10^{-1}	-
AN	IBS	MR-Robust	8	0.0040	36.5780	0.9995	0.8767	1.1394	9.94×10^{-1}	-
PUD	MDD	MR-Robust	7	0.0007	47.2585	0.9838	0.9356	1.0345	5.25×10^{-1}	-
MDD	PUD	MR-Robust	47	0.0035	37.5924	1.2846	1.1272	1.4640	1.73×10^{-4}	-
PUD	PTSD	MR-Robust	7	0.0007	47.2585	1.0102	0.8983	1.1360	8.66×10^{-1}	-
PTSD	PUD	MR-Robust	12	0.0048	28.1393	0.9802	0.8515	1.1283	7.80×10^{-1}	-
PUD	SCZ	MR-Robust	7	0.0007	47.2585	0.9930	0.8291	1.1894	9.39×10^{-1}	-
SCZ	PUD	MR-Robust	112	0.0463	45.5883	1.0266	0.9823	1.0730	2.43×10^{-1}	-
PUD	ADHD	MR-Robust	7	0.0007	47.2585	0.8544	0.7158	1.0199	8.15×10^{-2}	-
ADHD	PUD	MR-Robust	9	0.0138	35.4358	1.2005	1.0945	1.3167	1.06×10^{-4}	-
PUD	BIP	MR-Robust	7	0.0007	47.2585	1.0171	0.8900	1.1624	8.03×10^{-1}	-
BIP	PUD	MR-Robust	49	0.0050	42.0306	0.9472	0.8840	1.0149	1.23×10^{-1}	-
PUD	AN	MR-Robust	7	0.0007	47.2585	0.8850	0.7639	1.0253	1.04×10^{-1}	-
AN	PUD	MR-Robust	8	0.0040	36.5780	1.2349	0.9893	1.5415	6.22×10^{-2}	-
GORD	MDD	MR-Robust	5	0.0004	33.0918	1.1780	1.1006	1.2609	2.30×10^{-6}	-
MDD	GORD	MR-Robust	47	0.0035	37.5924	1.3671	1.2645	1.4781	4.11×10^{-15}	-
GORD	PTSD	MR-Robust	5	0.0004	33.0918	1.3384	0.9657	1.8550	8.01×10^{-2}	-

©2023 Gong W et al. *JAMA Psychiatry*.

**Exposure**	**Outcome**	**Method**	**IV num**	**PVE**	***F* statistic**	**OR**	**OR.lower**	**OR.upper**	***P* value**	***FDR adjusted P***
PTSD	GORD	MR-Robust	12	0.0048	28.1393	1.0997	1.0416	1.1611	5.98×10^{-4}	-
GORD	SCZ	MR-Robust	5	0.0004	33.0918	1.0536	0.7514	1.4772	7.62×10^{-1}	-
SCZ	GORD	MR-Robust	112	0.0463	45.5883	0.9984	0.9746	1.0228	8.97×10^{-1}	-
GORD	ADHD	MR-Robust	5	0.0004	33.0918	1.8055	1.1735	2.7777	7.19×10^{-3}	-
ADHD	GORD	MR-Robust	9	0.0138	35.4358	1.1116	1.0415	1.1863	1.44×10^{-3}	-
GORD	BIP	MR-Robust	5	0.0004	33.0918	1.3494	1.0810	1.6844	8.09×10^{-3}	-
BIP	GORD	MR-Robust	49	0.0050	42.0306	0.9527	0.9114	0.9959	3.21×10^{-2}	-
GORD	AN	MR-Robust	5	0.0004	33.0918	1.3782	1.0253	1.8526	3.36×10^{-2}	-
AN	GORD	MR-Robust	8	0.0040	36.5780	0.9888	0.9593	1.0192	4.65×10^{-1}	-
IBD	MDD	Weighted median	26	0.0026	45.6128	0.9814	0.9602	1.0030	9.14×10^{-2}	-
MDD	IBD	Weighted median	47	0.0035	37.5924	1.0177	0.7590	1.3645	9.07×10^{-1}	-
IBD	PTSD	Weighted median	26	0.0026	45.6128	1.0580	0.9877	1.1333	1.08×10^{-1}	-
PTSD	IBD	Weighted median	12	0.0048	28.1393	0.9318	0.7803	1.1129	4.36×10^{-1}	-
IBD	SCZ	Weighted median	24	0.0023	44.0766	1.0167	0.9643	1.0718	5.40×10^{-1}	-
SCZ	IBD	Weighted median	112	0.0463	45.5883	0.9832	0.9134	1.0584	6.53×10^{-1}	-
IBD	ADHD	Weighted median	25	0.0025	45.7931	1.0044	0.9403	1.0729	8.96×10^{-1}	-
ADHD	IBD	Weighted median	9	0.0138	35.4358	1.0173	0.8531	1.2132	8.48×10^{-1}	-
IBD	BIP	Weighted median	24	0.0024	46.2685	0.9504	0.9055	0.9977	4.00×10^{-2}	-
BIP	IBD	Weighted median	49	0.0050	42.0306	0.9718	0.8625	1.0949	6.38×10^{-1}	-
IBD	AN	Weighted median	26	0.0026	45.6128	0.9887	0.9297	1.0515	7.18×10^{-1}	-
AN	IBD	Weighted median	8	0.0040	36.5780	1.1018	0.9010	1.3473	3.45×10^{-1}	-
IBS	MDD	Weighted median	5	0.0003	32.8922	1.3951	1.1987	1.6237	1.70×10^{-5}	-
MDD	IBS	Weighted median	48	0.0036	37.7522	1.4827	1.3183	1.6676	5.06×10^{-11}	-

©2023 Gong W et al. *JAMA Psychiatry*.

Exposure	Outcome	Method	IV num	PVE	*F* statistic	OR	OR.lower	OR.upper	*P* value	*FDR adjusted P*
IBS	PTSD	Weighted median	6	0.0004	34.3919	1.6994	1.1192	2.5803	1.28×10^{-2}	-
PTSD	IBS	Weighted median	14	0.0054	27.3724	1.0369	0.9712	1.1071	2.78×10^{-1}	-
IBS	SCZ	Weighted median	6	0.0004	34.3919	1.0399	0.7808	1.3850	7.89×10^{-1}	-
SCZ	IBS	Weighted median	119	0.0485	45.0464	1.0385	1.0063	1.0717	1.87×10^{-2}	-
IBS	ADHD	Weighted median	5	0.0003	32.8922	1.5168	0.9810	2.3452	6.10×10^{-2}	-
ADHD	IBS	Weighted median	11	0.0171	36.0333	0.9763	0.8986	1.0607	5.71×10^{-1}	-
IBS	BIP	Weighted median	6	0.0004	34.3919	1.2223	0.9071	1.6470	1.87×10^{-1}	-
BIP	IBS	Weighted median	51	0.0051	41.6786	0.9950	0.9459	1.0467	8.48×10^{-1}	-
IBS	AN	Weighted median	5	0.0003	32.8922	1.2831	0.8197	2.0085	2.76×10^{-1}	-
AN	IBS	Weighted median	8	0.0040	36.5780	0.9762	0.9030	1.0552	5.44×10^{-1}	-
PUD	MDD	Weighted median	7	0.0007	47.2585	0.9775	0.9230	1.0351	4.36×10^{-1}	-
MDD	PUD	Weighted median	47	0.0035	37.5924	1.3209	1.1068	1.5764	2.04×10^{-3}	-
PUD	PTSD	Weighted median	7	0.0007	47.2585	1.0208	0.8529	1.2217	8.22×10^{-1}	-
PTSD	PUD	Weighted median	12	0.0048	28.1393	0.9278	0.8122	1.0598	2.69×10^{-1}	-
PUD	SCZ	Weighted median	7	0.0007	47.2585	1.0464	0.9060	1.2085	5.37×10^{-1}	-
SCZ	PUD	Weighted median	112	0.0463	45.5883	1.0294	0.9785	1.0829	2.63×10^{-1}	-
PUD	ADHD	Weighted median	7	0.0007	47.2585	0.8679	0.7209	1.0449	1.35×10^{-1}	-
ADHD	PUD	Weighted median	9	0.0138	35.4358	1.1716	1.0322	1.3298	1.42×10^{-2}	-
PUD	BIP	Weighted median	7	0.0007	47.2585	1.0702	0.9385	1.2204	3.11×10^{-1}	-
BIP	PUD	Weighted median	49	0.0050	42.0306	0.9249	0.8531	1.0028	5.86×10^{-2}	-
PUD	AN	Weighted median	7	0.0007	47.2585	0.8686	0.7271	1.0376	1.20×10^{-1}	-
AN	PUD	Weighted median	8	0.0040	36.5780	1.2367	1.0946	1.3974	6.48×10^{-4}	-
GORD	MDD	Weighted median	5	0.0004	33.0918	1.1524	1.0086	1.3166	3.70×10^{-2}	-

©2023 Gong W et al. *JAMA Psychiatry*.

Exposure	Outcome	Method	IV num	PVE	*F* statistic	OR	OR.lower	OR.upper	*P* value	*FDR adjusted P*
MDD	GORD	Weighted median	47	0.0035	37.5924	1.3361	1.2023	1.4848	7.37×10^{-8}	-
GORD	PTSD	Weighted median	5	0.0004	33.0918	1.3834	0.8740	2.1895	1.66×10^{-1}	-
PTSD	GORD	Weighted median	12	0.0048	28.1393	1.1157	1.0399	1.1972	2.30×10^{-3}	-
GORD	SCZ	Weighted median	5	0.0004	33.0918	1.0618	0.7787	1.4479	7.05×10^{-1}	-
SCZ	GORD	Weighted median	112	0.0463	45.5883	1.0109	0.9829	1.0396	4.50×10^{-1}	-
GORD	ADHD	Weighted median	5	0.0004	33.0918	2.0030	1.3066	3.0706	1.44×10^{-3}	-
ADHD	GORD	Weighted median	9	0.0138	35.4358	1.1088	1.0287	1.1952	6.96×10^{-3}	-
GORD	BIP	Weighted median	5	0.0004	33.0918	1.4637	1.0774	1.9885	1.48×10^{-2}	-
BIP	GORD	Weighted median	49	0.0050	42.0306	0.9369	0.8933	0.9827	7.48×10^{-3}	-
GORD	AN	Weighted median	5	0.0004	33.0918	1.4097	0.9230	2.1530	1.12×10^{-1}	-
AN	GORD	Weighted median	8	0.0040	36.5780	1.0053	0.9361	1.0798	8.84×10^{-1}	-
IBD	MDD	Weighted mode-based	26	0.0026	45.6128	0.9694	0.9345	1.0056	9.68×10^{-2}	-
MDD	IBD	Weighted mode-based	47	0.0035	37.5924	0.8807	0.3988	1.9446	7.53×10^{-1}	-
IBD	PTSD	Weighted mode-based	26	0.0026	45.6128	1.0503	0.9452	1.1672	3.61×10^{-1}	-
PTSD	IBD	Weighted mode-based	12	0.0048	28.1393	0.9758	0.7372	1.2915	8.64×10^{-1}	-
IBD	SCZ	Weighted mode-based	24	0.0023	44.0766	1.0242	0.9534	1.1002	5.13×10^{-1}	-
SCZ	IBD	Weighted mode-based	112	0.0463	45.5883	0.9312	0.7626	1.1371	4.84×10^{-1}	-
IBD	ADHD	Weighted mode-based	25	0.0025	45.7931	1.0015	0.9101	1.1022	9.75×10^{-1}	-
ADHD	IBD	Weighted mode-based	9	0.0138	35.4358	1.0908	0.8218	1.4477	5.48×10^{-1}	-
IBD	BIP	Weighted mode-based	24	0.0024	46.2685	0.9418	0.8774	1.0110	9.75×10^{-2}	-
BIP	IBD	Weighted mode-based	49	0.0050	42.0306	0.9571	0.7428	1.2333	7.35×10^{-1}	-
IBD	AN	Weighted mode-based	26	0.0026	45.6128	0.9821	0.8959	1.0765	6.99×10^{-1}	-
AN	IBD	Weighted mode-based	8	0.0040	36.5780	1.1020	0.7728	1.5716	5.92×10^{-1}	-

©2023 Gong W et al. *JAMA Psychiatry*.

Exposure	Outcome	Method	IV num	PVE	*F* statistic	OR	OR.lower	OR.upper	*P* value	*FDR adjusted P*
IBS	MDD	Weighted mode-based	5	0.0003	32.8922	1.3882	1.0834	1.7786	9.50×10^{-3}	-
MDD	IBS	Weighted mode-based	48	0.0036	37.7522	1.2734	0.9904	1.6373	5.95×10^{-2}	-
IBS	PTSD	Weighted mode-based	6	0.0004	34.3919	1.2446	0.6019	2.5737	5.55×10^{-1}	-
PTSD	IBS	Weighted mode-based	14	0.0054	27.3724	1.0326	0.9293	1.1474	5.50×10^{-1}	-
IBS	SCZ	Weighted mode-based	6	0.0004	34.3919	0.8374	0.5301	1.3227	4.47×10^{-1}	-
SCZ	IBS	Weighted mode-based	119	0.0485	45.0464	1.1219	1.0102	1.2460	3.16×10^{-2}	-
IBS	ADHD	Weighted mode-based	5	0.0003	32.8922	1.6080	0.8683	2.9778	1.31×10^{-1}	-
ADHD	IBS	Weighted mode-based	11	0.0171	36.0333	1.0084	0.8797	1.1558	9.05×10^{-1}	-
IBS	BIP	Weighted mode-based	6	0.0004	34.3919	0.7791	0.3389	1.7912	5.57×10^{-1}	-
BIP	IBS	Weighted mode-based	51	0.0051	41.6786	0.9855	0.8772	1.1072	8.05×10^{-1}	-
IBS	AN	Weighted mode-based	5	0.0003	32.8922	1.3270	0.7737	2.2760	3.04×10^{-1}	-
AN	IBS	Weighted mode-based	8	0.0040	36.5780	0.9511	0.8413	1.0751	4.22×10^{-1}	-
PUD	MDD	Weighted mode-based	7	0.0007	47.2585	0.9525	0.8764	1.0352	2.52×10^{-1}	-
MDD	PUD	Weighted mode-based	47	0.0035	37.5924	1.3196	0.8990	1.9371	1.57×10^{-1}	-
PUD	PTSD	Weighted mode-based	7	0.0007	47.2585	1.0454	0.8086	1.3515	7.35×10^{-1}	-
PTSD	PUD	Weighted mode-based	12	0.0048	28.1393	0.8528	0.7068	1.0289	9.63×10^{-2}	-
PUD	SCZ	Weighted mode-based	7	0.0007	47.2585	1.0279	0.8291	1.2743	8.02×10^{-1}	-
SCZ	PUD	Weighted mode-based	112	0.0463	45.5883	1.0838	0.9393	1.2505	2.70×10^{-1}	-
PUD	ADHD	Weighted mode-based	7	0.0007	47.2585	0.9858	0.7382	1.3165	9.23×10^{-1}	-
ADHD	PUD	Weighted mode-based	9	0.0138	35.4358	1.1337	0.9310	1.3806	2.12×10^{-1}	-
PUD	BIP	Weighted mode-based	7	0.0007	47.2585	1.0881	0.8826	1.3415	4.29×10^{-1}	-
BIP	PUD	Weighted mode-based	49	0.0050	42.0306	0.8098	0.6517	1.0064	5.71×10^{-2}	-
PUD	AN	Weighted mode-based	7	0.0007	47.2585	0.8962	0.6850	1.1725	4.24×10^{-1}	-

©2023 Gong W et al. *JAMA Psychiatry*.

Exposure	Outcome	Method	IV num	PVE	*F* statistic	OR	OR.lower	OR.upper	*P* value	*FDR adjusted P*
AN	PUD	Weighted mode-based	8	0.0040	36.5780	1.2425	1.0404	1.4839	1.65×10^{-2}	-
GORD	MDD	Weighted mode-based	5	0.0004	33.0918	1.1275	0.9480	1.3410	1.75×10^{-1}	-
MDD	GORD	Weighted mode-based	47	0.0035	37.5924	1.2979	1.0362	1.6256	2.32×10^{-2}	-
GORD	PTSD	Weighted mode-based	5	0.0004	33.0918	1.3814	0.7383	2.5847	3.12×10^{-1}	-
PTSD	GORD	Weighted mode-based	12	0.0048	28.1393	1.0956	0.9816	1.2229	1.03×10^{-1}	-
GORD	SCZ	Weighted mode-based	5	0.0004	33.0918	1.1875	0.7880	1.7894	4.11×10^{-1}	-
SCZ	GORD	Weighted mode-based	112	0.0463	45.5883	1.0257	0.9528	1.1041	5.01×10^{-1}	-
GORD	ADHD	Weighted mode-based	5	0.0004	33.0918	2.0986	1.1327	3.8880	1.85×10^{-2}	-
ADHD	GORD	Weighted mode-based	9	0.0138	35.4358	1.1828	1.0330	1.3543	1.51×10^{-2}	-
GORD	BIP	Weighted mode-based	5	0.0004	33.0918	1.4248	0.9275	2.1888	1.06×10^{-1}	-
BIP	GORD	Weighted mode-based	49	0.0050	42.0306	0.9024	0.8125	1.0023	5.52×10^{-2}	-
GORD	AN	Weighted mode-based	5	0.0004	33.0918	1.5717	0.8527	2.8969	1.47×10^{-1}	-
AN	GORD	Weighted mode-based	8	0.0040	36.5780	1.0040	0.9105	1.1071	9.36×10^{-1}	-
IBD	MDD	MR-RAPS	26	0.0026	45.6128	0.9989	0.9798	1.0184	9.10×10^{-1}	-
MDD	IBD	MR-RAPS	47	0.0035	37.5924	1.0452	0.8010	1.3638	7.45×10^{-1}	-
IBD	PTSD	MR-RAPS	26	0.0026	45.6128	1.0558	1.0029	1.1115	3.83×10^{-2}	-
PTSD	IBD	MR-RAPS	12	0.0048	28.1393	0.8939	0.7794	1.0252	1.09×10^{-1}	-
IBD	SCZ	MR-RAPS	24	0.0023	44.0766	0.9872	0.9396	1.0373	6.10×10^{-1}	-
SCZ	IBD	MR-RAPS	112	0.0463	45.5883	0.9991	0.9459	1.0553	9.75×10^{-1}	-
IBD	ADHD	MR-RAPS	25	0.0025	45.7931	1.0181	0.9653	1.0738	5.09×10^{-1}	-
ADHD	IBD	MR-RAPS	9	0.0138	35.4358	0.9876	0.8505	1.1468	8.70×10^{-1}	-
IBD	BIP	MR-RAPS	24	0.0024	46.2685	0.9667	0.9252	1.0100	1.30×10^{-1}	-
BIP	IBD	MR-RAPS	49	0.0050	42.0306	0.9831	0.8904	1.0855	7.37×10^{-1}	-

©2023 Gong W et al. *JAMA Psychiatry*.

Exposure	Outcome	Method	IV num	PVE	*F* statistic	OR	OR.lower	OR.upper	*P* value	*FDR adjusted P*
IBD	AN	MR-RAPS	26	0.0026	45.6128	0.9851	0.9397	1.0326	5.32×10^{-1}	-
AN	IBD	MR-RAPS	8	0.0040	36.5780	1.0972	0.9413	1.2789	2.36×10^{-1}	-
IBS	MDD	MR-RAPS	5	0.0003	32.8922	1.3719	1.1922	1.5787	1.01×10^{-5}	-
MDD	IBS	MR-RAPS	48	0.0036	37.7522	1.5143	1.3620	1.6836	1.69×10^{-14}	-
IBS	PTSD	MR-RAPS	6	0.0004	34.3919	1.7233	1.1258	2.6379	1.22×10^{-2}	-
PTSD	IBS	MR-RAPS	14	0.0054	27.3724	1.0289	0.9746	1.0862	3.03×10^{-1}	-
IBS	SCZ	MR-RAPS	6	0.0004	34.3919	1.1397	0.7632	1.7021	5.23×10^{-1}	-
SCZ	IBS	MR-RAPS	119	0.0485	45.0464	1.0330	1.0041	1.0628	2.47×10^{-2}	-
IBS	ADHD	MR-RAPS	5	0.0003	32.8922	1.4431	0.9898	2.1041	5.66×10^{-2}	-
ADHD	IBS	MR-RAPS	11	0.0171	36.0333	0.9399	0.8617	1.0251	1.62×10^{-1}	-
IBS	BIP	MR-RAPS	6	0.0004	34.3919	1.1351	0.6747	1.9099	6.33×10^{-1}	-
BIP	IBS	MR-RAPS	51	0.0051	41.6786	0.9972	0.9516	1.0449	9.05×10^{-1}	-
IBS	AN	MR-RAPS	5	0.0003	32.8922	1.3211	0.7102	2.4576	3.79×10^{-1}	-
AN	IBS	MR-RAPS	8	0.0040	36.5780	1.0020	0.9408	1.0671	9.51×10^{-1}	-
PUD	MDD	MR-RAPS	7	0.0007	47.2585	0.9802	0.9245	1.0394	5.05×10^{-1}	-
MDD	PUD	MR-RAPS	47	0.0035	37.5924	1.2870	1.1298	1.4661	1.47×10^{-4}	-
PUD	PTSD	MR-RAPS	7	0.0007	47.2585	1.0078	0.8675	1.1708	9.19×10^{-1}	-
PTSD	PUD	MR-RAPS	12	0.0048	28.1393	0.9946	0.8562	1.1554	9.43×10^{-1}	-
PUD	SCZ	MR-RAPS	7	0.0007	47.2585	1.0142	0.8710	1.1809	8.56×10^{-1}	-
SCZ	PUD	MR-RAPS	112	0.0463	45.5883	1.0226	0.9784	1.0687	3.22×10^{-1}	-
PUD	ADHD	MR-RAPS	7	0.0007	47.2585	0.8610	0.7426	0.9982	4.73×10^{-2}	-
ADHD	PUD	MR-RAPS	9	0.0138	35.4358	1.2086	1.0937	1.3355	2.01×10^{-4}	-
PUD	BIP	MR-RAPS	7	0.0007	47.2585	1.0240	0.9207	1.1389	6.62×10^{-1}	-

Exposure	Outcome	Method	IV num	PVE	*F* statistic	OR	OR.lower	OR.upper	*P* value	*FDR adjusted P*
BIP	PUD	MR-RAPS	49	0.0050	42.0306	0.9430	0.8824	1.0077	8.29×10^{-2}	-
PUD	AN	MR-RAPS	7	0.0007	47.2585	0.8841	0.7678	1.0181	8.72×10^{-2}	-
AN	PUD	MR-RAPS	8	0.0040	36.5780	1.1906	1.0737	1.3203	9.40×10^{-4}	-
GORD	MDD	MR-RAPS	5	0.0004	33.0918	1.1808	1.0547	1.3219	3.91×10^{-3}	-
MDD	GORD	MR-RAPS	47	0.0035	37.5924	1.3786	1.2693	1.4973	2.53×10^{-14}	-
GORD	PTSD	MR-RAPS	5	0.0004	33.0918	1.3575	0.9317	1.9779	1.11×10^{-1}	-
PTSD	GORD	MR-RAPS	12	0.0048	28.1393	1.1077	1.0444	1.1749	6.57×10^{-4}	-
GORD	SCZ	MR-RAPS	5	0.0004	33.0918	1.0801	0.8119	1.4369	5.97×10^{-1}	-
SCZ	GORD	MR-RAPS	112	0.0463	45.5883	0.9960	0.9724	1.0201	7.40×10^{-1}	-
GORD	ADHD	MR-RAPS	5	0.0004	33.0918	1.7952	1.2619	2.5539	1.14×10^{-3}	-
ADHD	GORD	MR-RAPS	9	0.0138	35.4358	1.1098	1.0366	1.1881	2.76×10^{-3}	-
GORD	BIP	MR-RAPS	5	0.0004	33.0918	1.3593	1.0301	1.7938	3.01×10^{-2}	-
BIP	GORD	MR-RAPS	49	0.0050	42.0306	0.9536	0.9138	0.9951	2.89×10^{-2}	-
GORD	AN	MR-RAPS	5	0.0004	33.0918	1.3777	0.9713	1.9542	7.24×10^{-2}	-
AN	GORD	MR-RAPS	8	0.0040	36.5780	1.0009	0.9446	1.0605	9.76×10^{-1}	-
IBD	MDD	MR-PRESSO	26	0.0026	45.6128	1.0007	0.9834	1.0183	9.36×10^{-1}	-
MDD	IBD	MR-PRESSO	47	0.0035	37.5924	1.0583	0.8284	1.3520	6.52×10^{-1}	-
IBD	PTSD	MR-PRESSO	26	0.0026	45.6128	1.0416	0.9902	1.0957	1.27×10^{-1}	-
PTSD	IBD	MR-PRESSO	12	0.0048	28.1393	0.9018	0.8058	1.0092	9.92×10^{-2}	-
IBD	SCZ	MR-PRESSO	24	0.0023	44.0766	0.9878	0.9351	1.0435	6.65×10^{-1}	-
SCZ	IBD	MR-PRESSO	112	0.0463	45.5883	0.9971	0.9467	1.0502	9.14×10^{-1}	-
IBD	ADHD	MR-PRESSO	25	0.0025	45.7931	1.0089	0.9563	1.0644	7.49×10^{-1}	-
ADHD	IBD	MR-PRESSO	9	0.0138	35.4358	0.9878	0.9029	1.0805	7.95×10^{-1}	-

©2023 Gong W et al. *JAMA Psychiatry*.

Exposure	Outcome	Method	IV num	PVE	*F* statistic	OR	OR.lower	OR.upper	*P* value	*FDR adjusted P*
IBD	BIP	MR-PRESSO	24	0.0024	46.2685	0.9787	0.9342	1.0254	3.74×10^{-1}	-
BIP	IBD	MR-PRESSO	49	0.0050	42.0306	0.9889	0.9021	1.0842	8.14×10^{-1}	-
IBD	AN	MR-PRESSO	26	0.0026	45.6128	0.9891	0.9503	1.0296	5.98×10^{-1}	-
AN	IBD	MR-PRESSO	8	0.0040	36.5780	1.0872	0.9114	1.2969	3.84×10^{-1}	-
IBS	MDD	MR-PRESSO	5	0.0003	32.8922	1.3522	1.1783	1.5517	1.27×10^{-2}	-
MDD	IBS	MR-PRESSO	48	0.0036	37.7522	1.5193	1.3742	1.6797	1.43×10^{-10}	-
IBS	PTSD	MR-PRESSO	6	0.0004	34.3919	1.7176	1.1580	2.5478	4.33×10^{-2}	-
PTSD	IBS	MR-PRESSO	14	0.0054	27.3724	1.0284	0.9959	1.0621	1.11×10^{-1}	-
IBS	SCZ	MR-PRESSO	6	0.0004	34.3919	1.1449	0.7685	1.7056	5.35×10^{-1}	-
SCZ	IBS	MR-PRESSO	119	0.0485	45.0464	1.0301	1.0028	1.0582	3.24×10^{-2}	-
IBS	ADHD	MR-PRESSO	5	0.0003	32.8922	1.4285	0.9929	2.0553	1.27×10^{-1}	-
ADHD	IBS	MR-PRESSO	11	0.0171	36.0333	0.9497	0.8679	1.0392	2.87×10^{-1}	-
IBS	BIP	MR-PRESSO	6	0.0004	34.3919	1.1879	0.7441	1.8966	5.03×10^{-1}	-
BIP	IBS	MR-PRESSO	51	0.0051	41.6786	1.0004	0.9548	1.0482	9.85×10^{-1}	-
IBS	AN	MR-PRESSO	5	0.0003	32.8922	1.3611	0.7200	2.5733	3.96×10^{-1}	-
AN	IBS	MR-PRESSO	8	0.0040	36.5780	1.0077	0.9498	1.0692	8.06×10^{-1}	-
PUD	MDD	MR-PRESSO	7	0.0007	47.2585	0.9848	0.9338	1.0387	5.94×10^{-1}	-
MDD	PUD	MR-PRESSO	47	0.0035	37.5924	1.2698	1.1135	1.4480	8.64×10^{-4}	-
PUD	PTSD	MR-PRESSO	7	0.0007	47.2585	1.0077	0.9224	1.1009	8.70×10^{-1}	-
PTSD	PUD	MR-PRESSO	12	0.0048	28.1393	0.9834	0.8628	1.1208	8.07×10^{-1}	-
PUD	SCZ	MR-PRESSO	7	0.0007	47.2585	1.0439	0.8553	1.2739	6.87×10^{-1}	-
SCZ	PUD	MR-PRESSO	112	0.0463	45.5883	1.0248	0.9835	1.0679	2.46×10^{-1}	-
PUD	ADHD	MR-PRESSO	7	0.0007	47.2585	0.8469	0.7297	0.9829	7.14×10^{-2}	-

©2023 Gong W et al. *JAMA Psychiatry*.

Exposure	Outcome	Method	IV num	PVE	*F* statistic	OR	OR.lower	OR.upper	*P* value	*FDR adjusted P*
ADHD	PUD	MR-PRESSO	9	0.0138	35.4358	1.2031	1.1018	1.3138	3.34×10^{-3}	-
PUD	BIP	MR-PRESSO	7	0.0007	47.2585	1.0075	0.9001	1.1277	9.01×10^{-1}	-
BIP	PUD	MR-PRESSO	49	0.0050	42.0306	0.9556	0.8972	1.0178	1.65×10^{-1}	-
PUD	AN	MR-PRESSO	7	0.0007	47.2585	0.8713	0.7636	0.9941	8.66×10^{-2}	-
AN	PUD	MR-PRESSO	8	0.0040	36.5780	1.1889	1.1199	1.2621	7.56×10^{-4}	-
GORD	MDD	MR-PRESSO	5	0.0004	33.0918	1.1787	1.0974	1.2660	1.07×10^{-2}	-
MDD	GORD	MR-PRESSO	47	0.0035	37.5924	1.3721	1.2707	1.4815	2.26×10^{-10}	-
GORD	PTSD	MR-PRESSO	5	0.0004	33.0918	1.3219	0.9240	1.8910	2.01×10^{-1}	-
PTSD	GORD	MR-PRESSO	12	0.0048	28.1393	1.0985	1.0421	1.1579	5.01×10^{-3}	-
GORD	SCZ	MR-PRESSO	5	0.0004	33.0918	1.0258	0.7449	1.4127	8.83×10^{-1}	-
SCZ	GORD	MR-PRESSO	112	0.0463	45.5883	0.9955	0.9720	1.0195	7.09×10^{-1}	-
GORD	ADHD	MR-PRESSO	5	0.0004	33.0918	1.7518	1.2946	2.3704	2.21×10^{-2}	-
ADHD	GORD	MR-PRESSO	9	0.0138	35.4358	1.1096	1.0430	1.1804	1.10×10^{-2}	-
GORD	BIP	MR-PRESSO	5	0.0004	33.0918	1.3485	1.0516	1.7292	7.80×10^{-2}	-
BIP	GORD	MR-PRESSO	49	0.0050	42.0306	0.9576	0.9193	0.9976	4.32×10^{-2}	-
GORD	AN	MR-PRESSO	5	0.0004	33.0918	1.3669	1.0136	1.8433	1.10×10^{-1}	-
AN	GORD	MR-PRESSO	8	0.0040	36.5780	1.0118	0.9553	1.0717	7.01×10^{-1}	-
IBD	SCZ	MR-PRESSO outlier-corrected	23	0.0022	43.8652	0.9706	0.9271	1.0162	2.16×10^{-1}	-
IBD	BIP	MR-PRESSO outlier-corrected	23	0.0023	46.1520	0.9668	0.9270	1.0082	1.29×10^{-1}	-
IBS	SCZ	MR-PRESSO outlier-corrected	4	0.0003	34.5434	1.1145	0.7619	1.6302	6.15×10^{-1}	-
SCZ	IBS	MR-PRESSO outlier-corrected	117	0.0473	44.6444	1.0304	1.0041	1.0574	2.49×10^{-2}	-
ADHD	IBS	MR-PRESSO outlier-corrected	10	0.0157	36.3595	0.9761	0.9036	1.0545	5.55×10^{-1}	-
IBS	BIP	MR-PRESSO outlier-corrected	3	0.0002	32.0981	1.4967	1.0045	2.2301	1.86×10^{-1}	-

©2023 Gong W et al. *JAMA Psychiatry*.

Exposure	Outcome	Method	IV num	PVE	*F* statistic	OR	OR.lower	OR.upper	*P* value	*FDR adjusted P*
BIP	IBS	MR-PRESSO outlier-corrected	50	0.0050	41.8406	0.9906	0.9496	1.0334	6.64×10^{-1}	-
IBS	AN	MR-PRESSO outlier-corrected	3	0.0002	32.9882	1.3413	1.2045	1.4936	3.32×10^{-2}	-
PUD	SCZ	MR-PRESSO outlier-corrected	5	0.0005	41.7615	1.0108	0.9340	1.0939	8.02×10^{-1}	-
SCZ	GORD	MR-PRESSO outlier-corrected	111	0.0456	45.3214	0.9894	0.9674	1.0119	3.54×10^{-1}	-

Abbreviations: NA, not applicable; IV num, the number of instrumental SNPs selected for Mendelian randomization analysis; PVE, proportion of variance of phenotype explained by variants; OR, odds ratio; FDR, false discovery rate.

Significant results with FDR adjusted *P* < 0.05 are highlighted in bold. Trait pairs without MR-PRESSO outlier-corrected results indicated that no outliers were detected.

©2023 Gong W et al. *JAMA Psychiatry*.

eTable 19. Results of Bidirectional Mendelian Randomization Analysis From Main Analysis and Alternative Methods for Negative Control Analysis[a]

Exposure	Outcome	Method	IV num	PVE	*F* statistic	OR	OR.lower	OR.upper	*P* value	*FDR adjusted P*
CAT	IBD	Inverse-variance weighted	14	0.0030	27.6889	1.0385	0.8849	1.2186	0.6439	0.9352
IBD	CAT	Inverse-variance weighted	24	0.0024	46.2685	0.9704	0.9246	1.0184	0.2225	0.8424
CAT	IBS	Inverse-variance weighted	14	0.0030	27.6889	1.0007	0.9465	1.0579	0.9816	0.9816
IBS	CAT	Inverse-variance weighted	5	0.0003	32.8922	0.8529	0.5389	1.3498	0.4969	0.9332
CAT	PUD	Inverse-variance weighted	14	0.0030	27.6889	1.0084	0.9173	1.1085	0.8630	0.9645
PUD	CAT	Inverse-variance weighted	7	0.0007	47.2585	0.8879	0.7476	1.0545	0.1754	0.8424
CAT	GORD	Inverse-variance weighted	14	0.0030	27.6889	0.9653	0.9176	1.0154	0.1716	0.8424
GORD	CAT	Inverse-variance weighted	5	0.0004	33.0918	1.3141	0.8424	2.0500	0.2286	0.8424
CAT	MDD	Inverse-variance weighted	14	0.0030	27.6889	0.9907	0.9602	1.0223	0.5599	0.9332
MDD	CAT	Inverse-variance weighted	44	0.0033	37.8070	1.0033	0.8516	1.1820	0.9689	0.9816
CAT	PTSD	Inverse-variance weighted	14	0.0030	27.6889	0.9369	0.8312	1.0561	0.2861	0.8424
PTSD	CAT	Inverse-variance weighted	11	0.0036	23.2560	0.9393	0.8237	1.0713	0.3507	0.8424
CAT	SCZ	Inverse-variance weighted	14	0.0030	27.6889	0.9559	0.8819	1.0361	0.2724	0.8424
SCZ	CAT	Inverse-variance weighted	104	0.0433	45.7778	0.9747	0.9302	1.0213	0.2820	0.8424
CAT	ADHD	Inverse-variance weighted	14	0.0030	27.6889	0.9533	0.8226	1.1049	0.5257	0.9332
ADHD	CAT	Inverse-variance weighted	9	0.0138	35.4358	1.0829	0.9625	1.2183	0.1854	0.8424
CAT	BIP	Inverse-variance weighted	14	0.0030	27.6889	0.9480	0.8645	1.0395	0.2563	0.8424
BIP	CAT	Inverse-variance weighted	50	0.0050	41.9266	1.0671	0.9726	1.1708	0.1699	0.8424
CAT	AN	Inverse-variance weighted	14	0.0030	27.6889	1.0619	0.9268	1.2167	0.3867	0.8594
AN	CAT	Inverse-variance weighted	7	0.0036	37.3932	1.0129	0.8418	1.2187	0.8922	0.9645
early AMD	IBD	Inverse-variance weighted	7	0.0118	178.7356	0.9540	0.8628	1.0548	0.3580	0.8424
IBD	early AMD	Inverse-variance weighted	26	0.0026	45.6128	1.0114	0.9649	1.0600	0.6374	0.9352

©2023 Gong W et al. *JAMA Psychiatry*.

Exposure	Outcome	Method	IV num	PVE	*F* statistic	OR	OR.lower	OR.upper	*P* value	*FDR adjusted P*
early AMD	IBS	Inverse-variance weighted	7	0.0118	178.7356	0.9979	0.9706	1.0261	0.8837	0.9645
IBS	early AMD	Inverse-variance weighted	6	0.0004	34.3919	0.9429	0.7058	1.2596	0.6906	0.9405
early AMD	PUD	Inverse-variance weighted	7	0.0118	178.7356	0.9909	0.9452	1.0389	0.7054	0.9405
PUD	early AMD	Inverse-variance weighted	7	0.0007	47.2585	0.9844	0.8428	1.1498	0.8431	0.9645
early AMD	GORD	Inverse-variance weighted	7	0.0118	178.7356	0.9715	0.9465	0.9971	0.0293	0.8424
GORD	early AMD	Inverse-variance weighted	5	0.0004	33.0918	1.0527	0.7502	1.4773	0.7663	0.9579
early AMD	MDD	Inverse-variance weighted	6	0.0114	202.7909	0.9917	0.9744	1.0094	0.3569	0.8424
MDD	early AMD	Inverse-variance weighted	48	0.0036	37.7522	0.9353	0.7867	1.1120	0.4486	0.8971
early AMD	PTSD	Inverse-variance weighted	7	0.0118	178.7356	1.0235	0.9640	1.0866	0.4469	0.8971
PTSD	early AMD	Inverse-variance weighted	14	0.0054	27.3724	1.0357	0.8882	1.2076	0.6547	0.9352
early AMD	SCZ	Inverse-variance weighted	7	0.0118	178.7356	1.0127	0.9388	1.0923	0.7444	0.9579
SCZ	early AMD	Inverse-variance weighted	119	0.0485	45.0464	1.0329	0.9871	1.0809	0.1613	0.8424
early AMD	ADHD	Inverse-variance weighted	7	0.0118	178.7356	0.9861	0.9332	1.0419	0.6172	0.9352
ADHD	early AMD	Inverse-variance weighted	11	0.0171	36.0333	0.9401	0.8342	1.0594	0.3107	0.8424
early AMD	BIP	Inverse-variance weighted	7	0.0118	178.7356	1.0401	0.9944	1.0878	0.0863	0.8424
BIP	early AMD	Inverse-variance weighted	51	0.0051	41.6786	1.0261	0.9426	1.1171	0.5511	0.9332
early AMD	AN	Inverse-variance weighted	7	0.0118	178.7356	1.0106	0.9171	1.1136	0.8314	0.9645
AN	early AMD	Inverse-variance weighted	8	0.0040	36.5780	0.9956	0.8601	1.1525	0.9534	0.9816
CAT	IBD	MR-Egger	14	0.0030	27.6889	0.9293	0.4908	1.7595	0.8219	-
IBD	CAT	MR-Egger	24	0.0024	46.2685	0.9679	0.8271	1.1326	0.6837	-
CAT	IBS	MR-Egger	14	0.0030	27.6889	1.0545	0.8463	1.3139	0.6361	-
IBS	CAT	MR-Egger	5	0.0003	32.8922	2.2439	0.0004	13635.0740	0.8557	-
CAT	PUD	MR-Egger	14	0.0030	27.6889	1.0472	0.7167	1.5302	0.8115	-

©2023 Gong W et al. *JAMA Psychiatry*.

Exposure	Outcome	Method	IV num	PVE	*F* statistic	OR	OR.lower	OR.upper	*P* value	*FDR adjusted P*
PUD	CAT	MR-Egger	7	0.0007	47.2585	1.0410	0.3207	3.3785	0.9467	-
CAT	GORD	MR-Egger	14	0.0030	27.6889	0.9180	0.7507	1.1227	0.4050	-
GORD	CAT	MR-Egger	5	0.0004	33.0918	0.0801	0.0087	0.7339	0.0255	-
CAT	MDD	MR-Egger	14	0.0030	27.6889	1.0368	0.9173	1.1718	0.5631	-
MDD	CAT	MR-Egger	44	0.0033	37.8070	0.5277	0.1600	1.7405	0.2938	-
CAT	PTSD	MR-Egger	14	0.0030	27.6889	1.0715	0.6669	1.7218	0.7752	-
PTSD	CAT	MR-Egger	11	0.0036	23.2560	0.6025	0.2753	1.3184	0.2048	-
CAT	SCZ	MR-Egger	14	0.0030	27.6889	0.9473	0.6869	1.3065	0.7414	-
SCZ	CAT	MR-Egger	104	0.0433	45.7778	1.0100	0.8037	1.2693	0.9321	-
CAT	ADHD	MR-Egger	14	0.0030	27.6889	0.9189	0.5093	1.6580	0.7789	-
ADHD	CAT	MR-Egger	9	0.0138	35.4358	0.8434	0.4804	1.4804	0.5529	-
CAT	BIP	MR-Egger	14	0.0030	27.6889	1.3505	0.9966	1.8300	0.0526	-
BIP	CAT	MR-Egger	50	0.0050	41.9266	0.9391	0.5472	1.6115	0.8195	-
CAT	AN	MR-Egger	14	0.0030	27.6889	1.2303	0.7204	2.1010	0.4479	-
AN	CAT	MR-Egger	7	0.0036	37.3932	1.8470	1.0592	3.2206	0.0306	-
early AMD	IBD	MR-Egger	7	0.0118	178.7356	0.9079	0.7579	1.0876	0.2945	-
IBD	early AMD	MR-Egger	26	0.0026	45.6128	1.2133	1.0380	1.4182	0.0151	-
early AMD	IBS	MR-Egger	7	0.0118	178.7356	1.0073	0.9605	1.0564	0.7631	-
IBS	early AMD	MR-Egger	6	0.0004	34.3919	0.0746	0.0021	2.6977	0.1562	-
early AMD	PUD	MR-Egger	7	0.0118	178.7356	1.0343	0.9574	1.1174	0.3922	-
PUD	early AMD	MR-Egger	7	0.0007	47.2585	1.0097	0.3333	3.0595	0.9863	-
early AMD	GORD	MR-Egger	7	0.0118	178.7356	0.9858	0.9428	1.0307	0.5283	-
GORD	early AMD	MR-Egger	5	0.0004	33.0918	0.3724	0.0410	3.3836	0.3803	-

©2023 Gong W et al. *JAMA Psychiatry*.

Exposure	Outcome	Method	IV num	PVE	*F* statistic	OR	OR.lower	OR.upper	*P* value	*FDR adjusted P*
early AMD	MDD	MR-Egger	6	0.0114	202.7909	0.9866	0.9561	1.0181	0.4015	-
MDD	early AMD	MR-Egger	48	0.0036	37.7522	0.8640	0.2443	3.0556	0.8206	-
early AMD	PTSD	MR-Egger	7	0.0118	178.7356	0.9621	0.8684	1.0658	0.4596	-
PTSD	early AMD	MR-Egger	14	0.0054	27.3724	0.7657	0.2768	2.1178	0.6070	-
early AMD	SCZ	MR-Egger	7	0.0118	178.7356	0.9513	0.8393	1.0782	0.4344	-
SCZ	early AMD	MR-Egger	119	0.0485	45.0464	0.9376	0.7533	1.1670	0.5640	-
early AMD	ADHD	MR-Egger	7	0.0118	178.7356	0.9713	0.8830	1.0684	0.5495	-
ADHD	early AMD	MR-Egger	11	0.0171	36.0333	0.6928	0.3727	1.2876	0.2458	-
early AMD	BIP	MR-Egger	7	0.0118	178.7356	1.0479	0.9635	1.1397	0.2751	-
BIP	early AMD	MR-Egger	51	0.0051	41.6786	0.8677	0.5346	1.4085	0.5659	-
early AMD	AN	MR-Egger	7	0.0118	178.7356	0.9205	0.7916	1.0705	0.2823	-
AN	early AMD	MR-Egger	8	0.0040	36.5780	0.8917	0.4845	1.6410	0.7126	-
CAT	IBD	MR-Egger intercept	14	0.0030	27.6889	1.0101	0.9551	1.0684	0.7241	-
IBD	CAT	MR-Egger intercept	24	0.0024	46.2685	1.0004	0.9800	1.0212	0.9728	-
CAT	IBS	MR-Egger intercept	14	0.0030	27.6889	0.9952	0.9762	1.0147	0.6284	-
IBS	CAT	MR-Egger intercept	5	0.0003	32.8922	0.9618	0.6776	1.3651	0.8274	-
CAT	PUD	MR-Egger intercept	14	0.0030	27.6889	0.9966	0.9639	1.0303	0.8396	-
PUD	CAT	MR-Egger intercept	7	0.0007	47.2585	0.9864	0.8926	1.0901	0.7885	-
CAT	GORD	MR-Egger intercept	14	0.0030	27.6889	1.0046	0.9870	1.0225	0.6129	-
GORD	CAT	MR-Egger intercept	5	0.0004	33.0918	1.1315	1.0273	1.2463	0.0122	-
CAT	MDD	MR-Egger intercept	14	0.0030	27.6889	0.9959	0.9852	1.0066	0.4512	-
MDD	CAT	MR-Egger intercept	44	0.0033	37.8070	1.0190	0.9843	1.0550	0.2868	-
CAT	PTSD	MR-Egger intercept	14	0.0030	27.6889	0.9879	0.9476	1.0299	0.5657	-

©2023 Gong W et al. *JAMA Psychiatry*.

**Exposure**	**Outcome**	**Method**	**IV num**	**PVE**	***F* statistic**	**OR**	**OR.lower**	**OR.upper**	***P* value**	***FDR adjusted P***
PTSD	CAT	MR-Egger intercept	11	0.0036	23.2560	1.0443	0.9685	1.1261	0.2597	-
CAT	SCZ	MR-Egger intercept	14	0.0030	27.6889	1.0008	0.9730	1.0295	0.9546	-
SCZ	CAT	MR-Egger intercept	104	0.0433	45.7778	0.9974	0.9810	1.0140	0.7553	-
CAT	ADHD	MR-Egger intercept	14	0.0030	27.6889	1.0034	0.9526	1.0568	0.8994	-
ADHD	CAT	MR-Egger intercept	9	0.0138	35.4358	1.0234	0.9726	1.0768	0.3732	-
CAT	BIP	MR-Egger intercept	14	0.0030	27.6889	0.9683	0.9427	0.9945	0.0181	-
BIP	CAT	MR-Egger intercept	50	0.0050	41.9266	1.0086	0.9732	1.0453	0.6377	-
CAT	AN	MR-Egger intercept	14	0.0030	27.6889	0.9866	0.9411	1.0344	0.5766	-
AN	CAT	MR-Egger intercept	7	0.0036	37.3932	0.9427	0.8943	0.9937	0.0283	-
early AMD	IBD	MR-Egger intercept	7	0.0118	178.7356	1.0122	0.9764	1.0493	0.5081	-
IBD	early AMD	MR-Egger intercept	26	0.0026	45.6128	0.9757	0.9563	0.9955	0.0165	-
early AMD	IBS	MR-Egger intercept	7	0.0118	178.7356	0.9977	0.9882	1.0073	0.6334	-
IBS	early AMD	MR-Egger intercept	6	0.0004	34.3919	1.1104	0.9580	1.2872	0.1644	-
early AMD	PUD	MR-Egger intercept	7	0.0118	178.7356	0.9895	0.9744	1.0049	0.1804	-
PUD	early AMD	MR-Egger intercept	7	0.0007	47.2585	0.9978	0.9086	1.0958	0.9638	-
early AMD	GORD	MR-Egger intercept	7	0.0118	178.7356	0.9964	0.9876	1.0053	0.4277	-
GORD	early AMD	MR-Egger intercept	5	0.0004	33.0918	1.0468	0.9510	1.1522	0.3503	-
early AMD	MDD	MR-Egger intercept	6	0.0114	202.7909	1.0014	0.9946	1.0082	0.6974	-
MDD	early AMD	MR-Egger intercept	48	0.0036	37.7522	1.0023	0.9660	1.0401	0.9012	-
early AMD	PTSD	MR-Egger intercept	7	0.0118	178.7356	1.0154	0.9948	1.0364	0.1446	-
PTSD	early AMD	MR-Egger intercept	14	0.0054	27.3724	1.0299	0.9337	1.1361	0.5558	-
early AMD	SCZ	MR-Egger intercept	7	0.0118	178.7356	1.0160	0.9901	1.0427	0.2283	-
SCZ	early AMD	MR-Egger intercept	119	0.0485	45.0464	1.0071	0.9914	1.0231	0.3753	-

©2023 Gong W et al. *JAMA Psychiatry*.

Exposure	Outcome	Method	IV num	PVE	*F* statistic	OR	OR.lower	OR.upper	*P* value	*FDR adjusted P*
early AMD	ADHD	MR-Egger intercept	7	0.0118	178.7356	1.0038	0.9845	1.0234	0.7028	-
ADHD	early AMD	MR-Egger intercept	11	0.0171	36.0333	1.0284	0.9726	1.0874	0.3253	-
early AMD	BIP	MR-Egger intercept	7	0.0118	178.7356	0.9981	0.9813	1.0153	0.8304	-
BIP	early AMD	MR-Egger intercept	51	0.0051	41.6786	1.0112	0.9796	1.0439	0.4906	-
early AMD	AN	MR-Egger intercept	7	0.0118	178.7356	1.0235	0.9928	1.0552	0.1348	-
AN	early AMD	MR-Egger intercept	8	0.0040	36.5780	1.0107	0.9548	1.0698	0.7140	-
CAT	IBD	MR-Robust	14	0.0030	27.6889	1.0511	0.8981	1.2301	0.5347	-
IBD	CAT	MR-Robust	24	0.0024	46.2685	0.9743	0.9256	1.0255	0.3185	-
CAT	IBS	MR-Robust	14	0.0030	27.6889	0.9883	0.9463	1.0322	0.5950	-
IBS	CAT	MR-Robust	5	0.0003	32.8922	0.9975	0.6063	1.6410	0.9922	-
CAT	PUD	MR-Robust	14	0.0030	27.6889	1.0032	0.8954	1.1239	0.9563	-
PUD	CAT	MR-Robust	7	0.0007	47.2585	0.9632	0.8081	1.1481	0.6755	-
CAT	GORD	MR-Robust	14	0.0030	27.6889	0.9522	0.9157	0.9901	0.0140	-
GORD	CAT	MR-Robust	5	0.0004	33.0918	1.3219	0.8696	2.0094	0.1915	-
CAT	MDD	MR-Robust	14	0.0030	27.6889	0.9896	0.9581	1.0222	0.5273	-
MDD	CAT	MR-Robust	44	0.0033	37.8070	1.0239	0.8674	1.2087	0.7801	-
CAT	PTSD	MR-Robust	14	0.0030	27.6889	0.8561	0.7305	1.0033	0.0550	-
PTSD	CAT	MR-Robust	11	0.0036	23.2560	0.9414	0.8168	1.0851	0.4048	-
CAT	SCZ	MR-Robust	14	0.0030	27.6889	0.9596	0.8814	1.0448	0.3423	-
SCZ	CAT	MR-Robust	104	0.0433	45.7778	0.9755	0.9285	1.0250	0.3260	-
CAT	ADHD	MR-Robust	14	0.0030	27.6889	0.9689	0.8082	1.1615	0.7327	-
ADHD	CAT	MR-Robust	9	0.0138	35.4358	1.0836	0.9853	1.1918	0.0981	-
CAT	BIP	MR-Robust	14	0.0030	27.6889	0.9268	0.8479	1.0130	0.0940	-

©2023 Gong W et al. *JAMA Psychiatry*.

Exposure	Outcome	Method	IV num	PVE	*F* statistic	OR	OR.lower	OR.upper	*P* value	*FDR adjusted P*
BIP	CAT	MR-Robust	50	0.0050	41.9266	1.0899	0.9908	1.1989	0.0767	-
CAT	AN	MR-Robust	14	0.0030	27.6889	1.0644	0.9391	1.2065	0.3286	-
AN	CAT	MR-Robust	7	0.0036	37.3932	0.9715	0.7743	1.2188	0.8024	-
early AMD	IBD	MR-Robust	7	0.0118	178.7356	0.9425	0.9034	0.9833	0.0061	-
IBD	early AMD	MR-Robust	26	0.0026	45.6128	1.0026	0.9284	1.0828	0.9464	-
early AMD	IBS	MR-Robust	7	0.0118	178.7356	0.9966	0.9553	1.0396	0.8748	-
IBS	early AMD	MR-Robust	6	0.0004	34.3919	0.9423	0.7696	1.1538	0.5651	-
early AMD	PUD	MR-Robust	7	0.0118	178.7356	0.9916	0.9588	1.0255	0.6239	-
PUD	early AMD	MR-Robust	7	0.0007	47.2585	0.9878	0.8464	1.1527	0.8759	-
early AMD	GORD	MR-Robust	7	0.0118	178.7356	0.9724	0.9563	0.9887	0.0010	-
GORD	early AMD	MR-Robust	5	0.0004	33.0918	1.0273	0.7012	1.5051	0.8901	-
early AMD	MDD	MR-Robust	6	0.0114	202.7909	0.9920	0.9760	1.0083	0.3333	-
MDD	early AMD	MR-Robust	48	0.0036	37.7522	0.9479	0.7946	1.1308	0.5525	-
early AMD	PTSD	MR-Robust	7	0.0118	178.7356	1.0221	0.9898	1.0555	0.1818	-
PTSD	early AMD	MR-Robust	14	0.0054	27.3724	1.0446	0.9005	1.2117	0.5649	-
early AMD	SCZ	MR-Robust	7	0.0118	178.7356	1.0114	0.9263	1.1043	0.8001	-
SCZ	early AMD	MR-Robust	119	0.0485	45.0464	1.0301	0.9820	1.0805	0.2240	-
early AMD	ADHD	MR-Robust	7	0.0118	178.7356	0.9844	0.9546	1.0151	0.3151	-
ADHD	early AMD	MR-Robust	11	0.0171	36.0333	0.9414	0.8322	1.0650	0.3372	-
early AMD	BIP	MR-Robust	7	0.0118	178.7356	1.0378	1.0069	1.0695	0.0160	-
BIP	early AMD	MR-Robust	51	0.0051	41.6786	1.0314	0.9411	1.1304	0.5082	-
early AMD	AN	MR-Robust	7	0.0118	178.7356	1.0142	0.8832	1.1646	0.8413	-
AN	early AMD	MR-Robust	8	0.0040	36.5780	0.9943	0.8805	1.1228	0.9264	-

©2023 Gong W et al. *JAMA Psychiatry*.

Exposure	Outcome	Method	IV num	PVE	*F* statistic	OR	OR.lower	OR.upper	*P* value	*FDR adjusted P*
CAT	IBD	Weighted median	14	0.0030	27.6889	1.0739	0.8993	1.2823	0.4308	-
IBD	CAT	Weighted median	24	0.0024	46.2685	0.9894	0.9257	1.0576	0.7549	-
CAT	IBS	Weighted median	14	0.0030	27.6889	0.9902	0.9259	1.0590	0.7749	-
IBS	CAT	Weighted median	5	0.0003	32.8922	1.0154	0.6558	1.5721	0.9454	-
CAT	PUD	Weighted median	14	0.0030	27.6889	0.9726	0.8623	1.0970	0.6514	-
PUD	CAT	Weighted median	7	0.0007	47.2585	0.9857	0.8225	1.1812	0.8756	-
CAT	GORD	Weighted median	14	0.0030	27.6889	0.9540	0.8969	1.0148	0.1350	-
GORD	CAT	Weighted median	5	0.0004	33.0918	1.5521	0.9492	2.5380	0.0797	-
CAT	MDD	Weighted median	14	0.0030	27.6889	0.9845	0.9428	1.0280	0.4789	-
MDD	CAT	Weighted median	44	0.0033	37.8070	0.9645	0.7682	1.2109	0.7556	-
CAT	PTSD	Weighted median	14	0.0030	27.6889	0.8721	0.7481	1.0168	0.0805	-
PTSD	CAT	Weighted median	11	0.0036	23.2560	0.9636	0.8131	1.1419	0.6686	-
CAT	SCZ	Weighted median	14	0.0030	27.6889	0.9820	0.8873	1.0868	0.7253	-
SCZ	CAT	Weighted median	104	0.0433	45.7778	0.9999	0.9374	1.0665	0.9964	-
CAT	ADHD	Weighted median	14	0.0030	27.6889	0.9396	0.8042	1.0979	0.4329	-
ADHD	CAT	Weighted median	9	0.0138	35.4358	1.0738	0.9242	1.2476	0.3521	-
CAT	BIP	Weighted median	14	0.0030	27.6889	0.9410	0.8541	1.0367	0.2186	-
BIP	CAT	Weighted median	50	0.0050	41.9266	1.1551	1.0402	1.2828	0.0070	-
CAT	AN	Weighted median	14	0.0030	27.6889	1.1150	0.9677	1.2848	0.1323	-
AN	CAT	Weighted median	7	0.0036	37.3932	0.9731	0.8087	1.1709	0.7727	-
early AMD	IBD	Weighted median	7	0.0118	178.7356	0.9393	0.8651	1.0200	0.1366	-
IBD	early AMD	Weighted median	26	0.0026	45.6128	1.0065	0.9396	1.0781	0.8545	-
early AMD	IBS	Weighted median	7	0.0118	178.7356	0.9845	0.9497	1.0206	0.3948	-

©2023 Gong W et al. *JAMA Psychiatry*.

Exposure	Outcome	Method	IV num	PVE	*F* statistic	OR	OR.lower	OR.upper	*P* value	*FDR adjusted P*
IBS	early AMD	Weighted median	6	0.0004	34.3919	0.9383	0.6591	1.3357	0.7236	-
early AMD	PUD	Weighted median	7	0.0118	178.7356	1.0010	0.9481	1.0568	0.9725	-
PUD	early AMD	Weighted median	7	0.0007	47.2585	0.9813	0.8132	1.1842	0.8440	-
early AMD	GORD	Weighted median	7	0.0118	178.7356	0.9752	0.9463	1.0050	0.1022	-
GORD	early AMD	Weighted median	5	0.0004	33.0918	1.0116	0.6568	1.5582	0.9582	-
early AMD	MDD	Weighted median	6	0.0114	202.7909	0.9910	0.9700	1.0125	0.4104	-
MDD	early AMD	Weighted median	48	0.0036	37.7522	0.9546	0.7654	1.1905	0.6800	-
early AMD	PTSD	Weighted median	7	0.0118	178.7356	1.0090	0.9413	1.0816	0.8007	-
PTSD	early AMD	Weighted median	14	0.0054	27.3724	1.0883	0.9305	1.2729	0.2897	-
early AMD	SCZ	Weighted median	7	0.0118	178.7356	1.0037	0.9423	1.0691	0.9092	-
SCZ	early AMD	Weighted median	119	0.0485	45.0464	1.0202	0.9593	1.0849	0.5241	-
early AMD	ADHD	Weighted median	7	0.0118	178.7356	0.9816	0.9204	1.0468	0.5708	-
ADHD	early AMD	Weighted median	11	0.0171	36.0333	0.9661	0.8271	1.1283	0.6628	-
early AMD	BIP	Weighted median	7	0.0118	178.7356	1.0374	0.9915	1.0853	0.1118	-
BIP	early AMD	Weighted median	51	0.0051	41.6786	1.0579	0.9518	1.1759	0.2966	-
early AMD	AN	Weighted median	7	0.0118	178.7356	1.0534	0.9627	1.1526	0.2577	-
AN	early AMD	Weighted median	8	0.0040	36.5780	0.9432	0.7972	1.1160	0.4959	-
CAT	IBD	Weighted mode-based	14	0.0030	27.6889	1.0740	0.8156	1.4141	0.6113	-
IBD	CAT	Weighted mode-based	24	0.0024	46.2685	1.0016	0.9002	1.1144	0.9773	-
CAT	IBS	Weighted mode-based	14	0.0030	27.6889	0.9804	0.8901	1.0798	0.6872	-
IBS	CAT	Weighted mode-based	5	0.0003	32.8922	1.0413	0.6341	1.7102	0.8729	-
CAT	PUD	Weighted mode-based	14	0.0030	27.6889	0.8875	0.6867	1.1469	0.3616	-
PUD	CAT	Weighted mode-based	7	0.0007	47.2585	0.9982	0.8054	1.2372	0.9872	-

©2023 Gong W et al. *JAMA Psychiatry*.

Exposure	Outcome	Method	IV num	PVE	*F* statistic	OR	OR.lower	OR.upper	*P* value	*FDR adjusted P*
CAT	GORD	Weighted mode-based	14	0.0030	27.6889	0.9606	0.8801	1.0485	0.3687	-
GORD	CAT	Weighted mode-based	5	0.0004	33.0918	1.7792	0.8684	3.6451	0.1154	-
CAT	MDD	Weighted mode-based	14	0.0030	27.6889	0.9812	0.9112	1.0567	0.6164	-
MDD	CAT	Weighted mode-based	44	0.0033	37.8070	0.8577	0.5232	1.4060	0.5427	-
CAT	PTSD	Weighted mode-based	14	0.0030	27.6889	0.8369	0.6263	1.1182	0.2284	-
PTSD	CAT	Weighted mode-based	11	0.0036	23.2560	1.0758	0.7822	1.4797	0.6530	-
CAT	SCZ	Weighted mode-based	14	0.0030	27.6889	1.0131	0.8597	1.1938	0.8769	-
SCZ	CAT	Weighted mode-based	104	0.0433	45.7778	1.0279	0.8588	1.2304	0.7640	-
CAT	ADHD	Weighted mode-based	14	0.0030	27.6889	1.1041	0.7952	1.5330	0.5543	-
ADHD	CAT	Weighted mode-based	9	0.0138	35.4358	0.9684	0.7547	1.2426	0.8005	-
CAT	BIP	Weighted mode-based	14	0.0030	27.6889	0.9140	0.7884	1.0596	0.2332	-
BIP	CAT	Weighted mode-based	50	0.0050	41.9266	1.2061	0.9452	1.5389	0.1318	-
CAT	AN	Weighted mode-based	14	0.0030	27.6889	1.1159	0.9212	1.3519	0.2624	-
AN	CAT	Weighted mode-based	7	0.0036	37.3932	0.9530	0.7046	1.2892	0.7550	-
early AMD	IBD	Weighted mode-based	7	0.0118	178.7356	0.9372	0.8679	1.0120	0.0976	-
IBD	early AMD	Weighted mode-based	26	0.0026	45.6128	0.9608	0.8331	1.1080	0.5825	-
early AMD	IBS	Weighted mode-based	7	0.0118	178.7356	0.9993	0.9648	1.0351	0.9706	-
IBS	early AMD	Weighted mode-based	6	0.0004	34.3919	0.7991	0.4654	1.3722	0.4163	-
early AMD	PUD	Weighted mode-based	7	0.0118	178.7356	1.0047	0.9549	1.0572	0.8556	-
PUD	early AMD	Weighted mode-based	7	0.0007	47.2585	1.0564	0.8058	1.3850	0.6912	-
early AMD	GORD	Weighted mode-based	7	0.0118	178.7356	0.9753	0.9472	1.0043	0.0940	-
GORD	early AMD	Weighted mode-based	5	0.0004	33.0918	0.9227	0.5065	1.6809	0.7926	-
early AMD	MDD	Weighted mode-based	6	0.0114	202.7909	0.9901	0.9690	1.0115	0.3620	-

©2023 Gong W et al. *JAMA Psychiatry*.

Exposure	Outcome	Method	IV num	PVE	*F* statistic	OR	OR.lower	OR.upper	*P* value	*FDR adjusted P*
MDD	early AMD	Weighted mode-based	48	0.0036	37.7522	0.9887	0.6102	1.6020	0.9632	-
early AMD	PTSD	Weighted mode-based	7	0.0118	178.7356	1.0073	0.9423	1.0768	0.8305	-
PTSD	early AMD	Weighted mode-based	14	0.0054	27.3724	1.1194	0.9022	1.3889	0.3053	-
early AMD	SCZ	Weighted mode-based	7	0.0118	178.7356	0.9811	0.9339	1.0307	0.4485	-
SCZ	early AMD	Weighted mode-based	119	0.0485	45.0464	1.0211	0.8604	1.2119	0.8112	-
early AMD	ADHD	Weighted mode-based	7	0.0118	178.7356	0.9810	0.9220	1.0437	0.5438	-
ADHD	early AMD	Weighted mode-based	11	0.0171	36.0333	1.0727	0.8262	1.3926	0.5984	-
early AMD	BIP	Weighted mode-based	7	0.0118	178.7356	1.0384	0.9949	1.0838	0.0843	-
BIP	early AMD	Weighted mode-based	51	0.0051	41.6786	1.0722	0.8425	1.3646	0.5710	-
early AMD	AN	Weighted mode-based	7	0.0118	178.7356	0.9939	0.9213	1.0721	0.8733	-
AN	early AMD	Weighted mode-based	8	0.0040	36.5780	0.9582	0.7682	1.1952	0.7047	-
CAT	IBD	MR-RAPS	14	0.0030	27.6889	1.0557	0.9010	1.2369	0.5029	-
IBD	CAT	MR-RAPS	24	0.0024	46.2685	0.9710	0.9227	1.0217	0.2573	-
CAT	IBS	MR-RAPS	14	0.0030	27.6889	0.9896	0.9390	1.0429	0.6950	-
IBS	CAT	MR-RAPS	5	0.0003	32.8922	0.9218	0.6255	1.3584	0.6806	-
CAT	PUD	MR-RAPS	14	0.0030	27.6889	1.0038	0.9093	1.1081	0.9402	-
PUD	CAT	MR-RAPS	7	0.0007	47.2585	0.9137	0.7875	1.0601	0.2341	-
CAT	GORD	MR-RAPS	14	0.0030	27.6889	0.9566	0.9109	1.0046	0.0756	-
GORD	CAT	MR-RAPS	5	0.0004	33.0918	1.2266	0.7498	2.0066	0.4160	-
CAT	MDD	MR-RAPS	14	0.0030	27.6889	0.9893	0.9560	1.0238	0.5393	-
MDD	CAT	MR-RAPS	44	0.0033	37.8070	1.0165	0.8589	1.2031	0.8487	-
CAT	PTSD	MR-RAPS	14	0.0030	27.6889	0.9000	0.8030	1.0088	0.0703	-
PTSD	CAT	MR-RAPS	11	0.0036	23.2560	0.9309	0.7998	1.0835	0.3552	-

©2023 Gong W et al. *JAMA Psychiatry*.

Exposure	Outcome	Method	IV num	PVE	*F* statistic	OR	OR.lower	OR.upper	*P* value	*FDR adjusted P*
CAT	SCZ	MR-RAPS	14	0.0030	27.6889	0.9624	0.8763	1.0570	0.4227	-
SCZ	CAT	MR-RAPS	104	0.0433	45.7778	0.9742	0.9277	1.0229	0.2937	-
CAT	ADHD	MR-RAPS	14	0.0030	27.6889	0.9743	0.8348	1.1371	0.7409	-
ADHD	CAT	MR-RAPS	9	0.0138	35.4358	1.0841	0.9559	1.2295	0.2086	-
CAT	BIP	MR-RAPS	14	0.0030	27.6889	0.9408	0.8561	1.0339	0.2052	-
BIP	CAT	MR-RAPS	50	0.0050	41.9266	1.0850	0.9850	1.1952	0.0981	-
CAT	AN	MR-RAPS	14	0.0030	27.6889	1.0657	0.9368	1.2123	0.3332	-
AN	CAT	MR-RAPS	7	0.0036	37.3932	0.9907	0.8295	1.1833	0.9183	-
early AMD	IBD	MR-RAPS	7	0.0118	178.7356	0.9441	0.8709	1.0235	0.1629	-
IBD	early AMD	MR-RAPS	26	0.0026	45.6128	1.0042	0.9559	1.0549	0.8681	-
early AMD	IBS	MR-RAPS	7	0.0118	178.7356	0.9979	0.9696	1.0270	0.8871	-
IBS	early AMD	MR-RAPS	6	0.0004	34.3919	0.9421	0.6913	1.2840	0.7058	-
early AMD	PUD	MR-RAPS	7	0.0118	178.7356	0.9917	0.9447	1.0410	0.7360	-
PUD	early AMD	MR-RAPS	7	0.0007	47.2585	0.9890	0.8386	1.1665	0.8959	-
early AMD	GORD	MR-RAPS	7	0.0118	178.7356	0.9714	0.9455	0.9979	0.0349	-
GORD	early AMD	MR-RAPS	5	0.0004	33.0918	1.0463	0.7295	1.5007	0.8057	-
early AMD	MDD	MR-RAPS	6	0.0114	202.7909	0.9917	0.9738	1.0100	0.3708	-
MDD	early AMD	MR-RAPS	48	0.0036	37.7522	0.9446	0.7983	1.1176	0.5062	-
early AMD	PTSD	MR-RAPS	7	0.0118	178.7356	1.0236	0.9622	1.0889	0.4606	-
PTSD	early AMD	MR-RAPS	14	0.0054	27.3724	1.0336	0.8879	1.2031	0.6702	-
early AMD	SCZ	MR-RAPS	7	0.0118	178.7356	1.0070	0.9312	1.0890	0.8614	-
SCZ	early AMD	MR-RAPS	119	0.0485	45.0464	1.0308	0.9821	1.0819	0.2189	-
early AMD	ADHD	MR-RAPS	7	0.0118	178.7356	0.9841	0.9297	1.0417	0.5811	-

©2023 Gong W et al. *JAMA Psychiatry*.

Exposure	Outcome	Method	IV num	PVE	*F* statistic	OR	OR.lower	OR.upper	*P* value	*FDR adjusted P*
ADHD	early AMD	MR-RAPS	11	0.0171	36.0333	0.9310	0.8143	1.0645	0.2959	-
early AMD	BIP	MR-RAPS	7	0.0118	178.7356	1.0373	0.9950	1.0814	0.0849	-
BIP	early AMD	MR-RAPS	51	0.0051	41.6786	1.0337	0.9429	1.1333	0.4801	-
early AMD	AN	MR-RAPS	7	0.0118	178.7356	0.9985	0.9045	1.1023	0.9764	-
AN	early AMD	MR-RAPS	8	0.0040	36.5780	0.9947	0.8719	1.1347	0.9368	-
CAT	IBD	MR-PRESSO	14	0.0030	27.6889	1.0385	0.8849	1.2186	0.6515	-
IBD	CAT	MR-PRESSO	24	0.0024	46.2685	0.9704	0.9377	1.0042	0.0984	-
CAT	IBS	MR-PRESSO	14	0.0030	27.6889	1.0007	0.9465	1.0579	0.9819	-
IBS	CAT	MR-PRESSO	5	0.0003	32.8922	0.8529	0.5389	1.3498	0.5342	-
CAT	PUD	MR-PRESSO	14	0.0030	27.6889	1.0084	0.9173	1.1085	0.8657	-
PUD	CAT	MR-PRESSO	7	0.0007	47.2585	0.8879	0.7476	1.0545	0.2242	-
CAT	GORD	MR-PRESSO	14	0.0030	27.6889	0.9653	0.9176	1.0154	0.1947	-
GORD	CAT	MR-PRESSO	5	0.0004	33.0918	1.3141	0.8424	2.0500	0.2950	-
CAT	MDD	MR-PRESSO	14	0.0030	27.6889	0.9907	0.9602	1.0223	0.5699	-
MDD	CAT	MR-PRESSO	44	0.0033	37.8070	1.0033	0.8516	1.1820	0.9691	-
CAT	PTSD	MR-PRESSO	14	0.0030	27.6889	0.9369	0.8312	1.0561	0.3055	-
PTSD	CAT	MR-PRESSO	11	0.0036	23.2560	0.9393	0.8237	1.0713	0.3727	-
CAT	SCZ	MR-PRESSO	14	0.0030	27.6889	0.9559	0.8819	1.0361	0.2923	-
SCZ	CAT	MR-PRESSO	104	0.0433	45.7778	0.9747	0.9302	1.0213	0.2845	-
CAT	ADHD	MR-PRESSO	14	0.0030	27.6889	0.9533	0.8226	1.1049	0.5367	-
ADHD	CAT	MR-PRESSO	9	0.0138	35.4358	1.0829	0.9934	1.1805	0.1081	-
CAT	BIP	MR-PRESSO	14	0.0030	27.6889	0.9480	0.8645	1.0395	0.2768	-
BIP	CAT	MR-PRESSO	50	0.0050	41.9266	1.0671	0.9726	1.1708	0.1762	-

©2023 Gong W et al. *JAMA Psychiatry*.

Exposure	Outcome	Method	IV num	PVE	*F* statistic	OR	OR.lower	OR.upper	*P* value	*FDR adjusted P*
CAT	AN	MR-PRESSO	14	0.0030	27.6889	1.0619	0.9268	1.2167	0.4024	-
AN	CAT	MR-PRESSO	7	0.0036	37.3932	1.0129	0.8418	1.2188	0.8966	-
early AMD	IBD	MR-PRESSO	7	0.0118	178.7356	0.9540	0.8628	1.0548	0.3935	-
IBD	early AMD	MR-PRESSO	26	0.0026	45.6128	1.0114	0.9661	1.0587	0.6327	-
early AMD	IBS	MR-PRESSO	7	0.0118	178.7356	0.9979	0.9760	1.0203	0.8606	-
IBS	early AMD	MR-PRESSO	6	0.0004	34.3919	0.9429	0.7653	1.1616	0.6044	-
early AMD	PUD	MR-PRESSO	7	0.0118	178.7356	0.9909	0.9452	1.0389	0.7184	-
PUD	early AMD	MR-PRESSO	7	0.0007	47.2585	0.9844	0.8428	1.1498	0.8496	-
early AMD	GORD	MR-PRESSO	7	0.0118	178.7356	0.9715	0.9538	0.9895	0.0215	-
GORD	early AMD	MR-PRESSO	5	0.0004	33.0918	1.0527	0.7797	1.4214	0.7542	-
early AMD	MDD	MR-PRESSO	6	0.0114	202.7909	0.9917	0.9767	1.0070	0.3370	-
MDD	early AMD	MR-PRESSO	48	0.0036	37.7522	0.9353	0.7867	1.1120	0.4523	-
early AMD	PTSD	MR-PRESSO	7	0.0118	178.7356	1.0235	0.9785	1.0706	0.3503	-
PTSD	early AMD	MR-PRESSO	14	0.0054	27.3724	1.0357	0.8882	1.2076	0.6620	-
early AMD	SCZ	MR-PRESSO	7	0.0118	178.7356	1.0127	0.9388	1.0923	0.7555	-
SCZ	early AMD	MR-PRESSO	119	0.0485	45.0464	1.0329	0.9871	1.0809	0.1639	-
early AMD	ADHD	MR-PRESSO	7	0.0118	178.7356	0.9861	0.9365	1.0382	0.6126	-
ADHD	early AMD	MR-PRESSO	11	0.0171	36.0333	0.9401	0.8342	1.0594	0.3346	-
early AMD	BIP	MR-PRESSO	7	0.0118	178.7356	1.0401	0.9944	1.0879	0.1371	-
BIP	early AMD	MR-PRESSO	51	0.0051	41.6786	1.0261	0.9426	1.1171	0.5538	-
early AMD	AN	MR-PRESSO	7	0.0118	178.7356	1.0106	0.9171	1.1136	0.8385	-
AN	early AMD	MR-PRESSO	8	0.0040	36.5780	0.9956	0.8601	1.1525	0.9551	-
CAT	ADHD	MR-PRESSO outlier-corrected	13	0.0028	27.9317	0.9922	0.8673	1.1351	0.9109	-

©2023 Gong W et al. *JAMA Psychiatry*.

Exposure	Outcome	Method	IV num	PVE	*F* statistic	OR	OR.lower	OR.upper	*P* value	*FDR adjusted P*
CAT	BIP	MR-PRESSO outlier-corrected	13	0.0028	27.5457	0.9148	0.8543	0.9796	0.0255	-
BIP	CAT	MR-PRESSO outlier-corrected	48	0.0049	42.0811	1.1029	1.0156	1.1978	0.0243	-

Abbreviations: IV num, the number of instrumental SNPs selected for Mendelian randomization analysis; PVE, proportion of variance of phenotype explained by variants; OR, odds ratio; FDR, false discovery rate.

[a] Significant threshold was set at FDR adjusted $P < 0.05$. As expected, no significant causal relationships were detected in this negative control analysis. Trait pairs without MR-PRESSO outlier-corrected results indicated that no outliers were detected.

©2023 Gong W et al. *JAMA Psychiatry*.

eTable 20. Results of Mendelian Randomization Analysis Using LHC-MR Method[a]

Trait pair	confound_x	confound_y	beta_xy	beta_yx	intercept_xy	*P*.confound_x	*P*.confound_y	*P*.beta_xy	*P*.beta_yx	*P*.intercept_xy
IBS–MDD	0.0077	–0.0001	0.4471	0.2958	0.1057	8.79×10^{-1}	9.97×10^{-1}	**1.65×10^{-5}**	**1.61×10^{-10}**	1.54×10^{-19}
IBS–PTSD	0.0019	–0.1253	0.5621	0.1430	0.0426	9.48×10^{-1}	3.22×10^{-4}	**6.73×10^{-3}**	3.51×10^{-1}	1.73×10^{-6}
IBS–BIP	0.0444	0.0748	–0.5110	0.1498	0.0445	7.84×10^{-1}	7.31×10^{-1}	4.35×10^{-1}	2.65×10^{-1}	3.12×10^{-2}
PUD–MDD	0.0026	–0.0353	–0.0825	0.2040	0.0543	8.91×10^{-1}	2.55×10^{-1}	2.80×10^{-1}	**2.97×10^{-57}**	3.07×10^{-28}
GORD–MDD	0.0013	0.0232	0.2361	0.2898	0.0948	6.82×10^{-1}	4.99×10^{-1}	**4.23×10^{-6}**	**1.51×10^{-48}**	6.26×10^{-37}

Abbreviations: LHC-MR, Latent Heritable Confounder Mendelian randomization**;** confound_x and confound_y represent confounder effects on trait 1 and trait 2, respectively; beta_xy and beta_yx represent causal effect of trait 1 on trait 2, causal effect of trait 2 on trait 1, resprectively. LHC-MR, Latent Heritable Confounder Mendelian randomization; i_xy.cross-trait intercept calculated by GenomicSEM::ldsc() function.

[a] LHC-MR methods was applied to estimate the causal relationships between 5 pairwise trait, which was suggested to show potential sample overlap in pairwise LDSC analysis, to further validate the MR results. Significant results were highlighted in bold.

eTable 21. Comparisons of Data Sources of Psychiatric Disorders-Related GWAS in Genetic Correlation and Mendelian Randomization Analysis

Phenotype	Abbreviation	PMID	Year	N_cases	N_controls	N_total	Ancestry
Wu et al. (2021)							
Anxiety disorders	ANX	26754954	2016	\	\	17310	European
Attention deficit/hyperactivity disorder	ADHD	30478444	2019	19099	34194	53293	European
Autism spectrum disorder	ASD	30804558	2019	18381	27969	46350	European
Biploar disorder	BIP	31043756	2019	20352	31358	51710	European
Major depressive disorder*	MDD	29700475	2018	59851	113154	173005	European
Post-traumatic stress disorder	PTSD	28439101	2017	2489	7465	9954	European
Schizophrenia	SCZ	29483656	2018	40675	64643	105318	European
Eijsbouts C et al. (2021)							
**Genetic correlation analysis**							
Anxiety or panic attacks	ANX	0	\	\	\	\	European
Bipolar disorder	BIP	21926972	2011	7481	9250	16731	European
Depressive symptoms	DS	27089181	2016	\	\	161460	European
Neuroticism	NEU	27089181	2016	\	\	170910	European
Schizophrenia	SCZ	25056061	2014	36989	113075	150064	Mixed
**Mendelian randomization analysis**							
Anxiety	ANX	31906708	2020	\	\	175163	European
Bipolar disorder	BIP	31043756	2019	20352	31358	51710	European
Major depressive disorder	MDD	29700475	2018	59851	113154	173005	European
Neuroticism	NEU	29255261	2018	\	\	329821	European
Schizophrenia	SCZ	25056061	2014	36989	113075	150064	Mixed
Gong et al. (current study)							
Anorexia nervosa*	AN	31308545	2019	16992	55525	72517	European
Attention deficit hyperactivity disorder*	ADHD	30478444	2019	19099	34194	53293	European
Bipolar disorder*	BIP	34002096	2021	41917	371549	413466	European
Major depressive disorder*	MDD	30718901	2019	170756	329443	500199	European
Post-traumatic stress disorder*	PTSD	31594949	2019	23212	151447	174659	European
Schizophrenia*	SCZ	29483656	2018	40675	64643	105318	European

Note: The GWASs performed on multi-ancestry were highlighted in grey. The GWAS data commonly used in Wu et al. (2021) and in the current study were highlighted in light

©2023 Gong W et al. *JAMA Psychiatry*.

yellow. Note that no identical psychiatric disorders-related GWAS were used in Eijsbouts C et al. (2021) and in the current study.
* The corresponding GWAS data simultaneously used in both genetic correlation analysis and Mendelian randomization analysis in each study.

©2023 Gong W et al. *JAMA Psychiatry*.

eTable 22. Summary of Genetic Correlation Results Between 4 Gastrointestinal Tract Diseases and Psychiatric Disorders

Trait 1	Trait 2	LDSC.rg	LDSC.se	LDSC.p	rg	se	p
IBD *Wu et al. (2021)*	MDD	0.17	0.041	2.82E-05	0.15	0.043	3.0E-04
	PTSD	0.17	0.038	7.39E-02	0.20	0.161	2.1E-01
	SCZ	0.04	0.040	3.73E-01	0.04	0.040	3.7E-01
	ADHD	0.00	0.060	9.40E-01	0.00	0.059	9.6E-01
	BIP	0.02	0.048	6.45E-01	0.02	0.053	7.6E-01
	AN	-0.03	0.063	6.38E-01	/	/	/
	ANX	/	/	/	0.23	0.148	1.2E-01
	ASD	/	/	/	-0.05	0.059	3.8E-01
PUD *Wu et al. (2021)*	MDD	0.44	0.044	3.31E-24	0.37	0.045	2.1E-16
	PTSD	0.54	0.112	1.15E-06	0.30	0.164	7.2E-02
	SCZ	0.13	0.040	1.20E-03	0.13	0.040	1.0E-03
	ADHD	0.48	0.058	1.63E-16	0.48	0.057	9.1E-17
	BIP	0.07	0.044	1.14E-01	0.01	0.046	8.0E-01
	AN	0.04	0.054	4.97E-01	/	/	/
	ANX	/	/	/	0.26	0.179	1.5E-01
	ASD	/	/	/	0.00	0.066	9.7E-01
GORD *Wu et al. (2021)*	MDD	0.46	0.027	3.50E-66	0.37	0.027	3.3E-41
	PTSD	0.42	0.069	1.24E-09	0.23	0.119	4.8E-02
	SCZ	0.03	0.027	2.51E-01	0.03	0.027	2.4E-01
	ADHD	0.49	0.037	3.32E-40	0.49	0.036	3.1E-42
	BIP	0.03	0.031	2.81E-01	-0.04	0.034	2.5E-01
	AN	0.02	0.039	6.43E-01	/	/	/
	ANX	/	/	/	0.30	0.127	1.7E-02
	ASD	/	/	/	-0.01	0.043	8.6E-01
IBS *Eijsbouts C et al. (2021)*	MDD	0.57	0.026	1.14E-109	0.53	0.047	3.6E-30
	PTSD	0.47	0.080	2.87E-09	/	/	/
	SCZ	0.17	0.029	1.85E-09	0.15	0.035	1.5E-05
	ADHD	0.21	0.041	3.73E-07	/	/	/
	BIP	0.13	0.031	3.12E-05	0.15	0.047	1.8E-03
	AN	0.15	0.041	2.00E-04	/	/	/
	ANX	/	/	/	0.58	0.096	1.6E-09
	NEU	/	/	/	0.54	0.035	5.2E-53

Note: The LDSC.rg, LDSC.se, LDSC.p represent the genetic correlation in the current study, and rg, se, p represent the genetic correlation between IBD, PUD, GORD and psychiatric disorders reported in Wu et al. (2021) and between IBS and psychiatric disorders reported in Eijsbouts C et al. (2021). The significant results reported in corresponding studies were highlighted in light yellow. See Table 1 above for the full names of the abbreviations of the GIT diseases (Trait 1) and PSY disorders (Trait 2).

©2023 Gong W et al. *JAMA Psychiatry*.

eTable 23. Summary of Associations Between Gastrointestinal Tract Diseases and Psychiatric Disorders in Mendelian Randomization Analysis

Trait 1	Trait 2	Direction Trait 1→Trait 2					Direction Trait 2→Trait 1				
		b_{xy}	se	*P*	No. of SNPs	*FDR adjusted P*	b_{xy}	se	*P*	No. of SNPs	*FDR adjusted P*
Wu et al (2021)											
GORD	MDD	0.18	0.036	7.30E-07	17	\	0.21	0.038	2.20E-08	32	\
IBS							0.39	0.050	6.40E-15	33	\
Eijsbouts C et al. (2021)											
IBS	ANX	0.098	\	0.00E+00	6	\	0.205	\	3.56E-02	5	\
	MDD	0.522	\	9.03E-09	6	\	0.341	\	1.65E-10	40	\
	NEU	0.184	\	1.14E-03	6	\	0.918	\	9.34E-07	15	\
	SCZ						0.044	\	5.90E-03	94	\
Gong et al. (current study)											
PUD	MDD						0.265	0.062	1.90E-05	54	2.28E-04
	ADHD						0.161	0.041	1.00E-04	12	9.64E-04
	AN						0.152	0.042	3.45E-04	11	2.59E-03
GORD	MDD	0.137	0.049	5.11E-03	6	2.69E-02	0.347	0.037	1.42E-20	54	6.82E-19
	ADHD	0.518	0.152	6.78E-04	6	4.07E-03	0.105	0.030	3.78E-04	12	2.59E-03
	BIP	0.297	0.107	5.60E-03	6	2.69E-02					
IBS	MDD	0.302	0.070	1.51E-05	5	2.28E-04	0.433	0.050	8.80E-18	54	2.11E-16
	PTSD	0.543	0.199	6.45E-03	6	2.82E-02					

Note: The bidirectional causal relationships were highlighted in light yellow, while the unidirectional causal relationships (either causal effect of GIT disease on PSY or vice versa) were highlighted in light green. See Table 1 above for the full names of the abbreviations of the GIT diseases (Trait 1) and PSY disorders (Trait 2). FDR, false discovery rate.

©2023 Gong W et al. *JAMA Psychiatry*.

eReferences.

1. Demontis D, Walters RK, Martin J, et al. Discovery of the first genome-wide significant risk loci for attention deficit/hyperactivity disorder. *Nat Genet*. Jan 2019;51(1):63-75. doi:10.1038/s41588-018-0269-7

2. Eijsbouts C, Zheng T, Kennedy NA, et al. Genome-wide analysis of 53,400 people with irritable bowel syndrome highlights shared genetic pathways with mood and anxiety disorders. *Nat Genet*. Nov 2021;53(11):1543-1552. doi:10.1038/s41588-021-00950-8

3. Howard DM, Adams MJ, Clarke TK, et al. Genome-wide meta-analysis of depression identifies 102 independent variants and highlights the importance of the prefrontal brain regions. *Nat Neurosci*. Mar 2019;22(3):343-352. doi:10.1038/s41593-018-0326-7

4. Mullins N, Forstner AJ, O'Connell KS, et al. Genome-wide association study of more than 40,000 bipolar disorder cases provides new insights into the underlying biology. *Nat Genet*. Jun 2021;53(6):817-829. doi:10.1038/s41588-021-00857-4

5. Nievergelt CM, Maihofer AX, Klengel T, et al. International meta-analysis of PTSD genome-wide association studies identifies sex- and ancestry-specific genetic risk loci. *Nat Commun*. Oct 8 2019;10(1):4558. doi:10.1038/s41467-019-12576-w

6. Pardinas AF, Holmans P, Pocklington AJ, et al. Common schizophrenia alleles are enriched in mutation-intolerant genes and in regions under strong background selection. *Nat Genet*. Mar 2018;50(3):381-389. doi:10.1038/s41588-018-0059-2

7. Watson HJ, Yilmaz Z, Thornton LM, et al. Genome-wide association study identifies eight risk loci and implicates metabo-psychiatric origins for anorexia nervosa. *Nat Genet*. Aug 2019;51(8):1207-1214. doi:10.1038/s41588-019-0439-2

8. Wu Y, Murray GK, Byrne EM, Sidorenko J, Visscher PM, Wray NR. GWAS of peptic ulcer disease implicates Helicobacter pylori infection, other gastrointestinal disorders and depression. *Nat Commun*. Feb 19 2021;12(1):1146. doi:10.1038/s41467-021-21280-7

9. Watanabe K, Stringer S, Frei O, et al. A global overview of pleiotropy and genetic architecture in complex traits. *Nat Genet*. Sep 2019;51(9):1339-1348. doi:10.1038/s41588-019-0481-0

10. Winkler TW, Grassmann F, Brandl C, et al. Genome-wide association meta-analysis for early age-related macular degeneration highlights novel loci and insights for advanced disease. *BMC Med Genomics*. Aug 26 2020;13(1):120. doi:10.1186/s12920-020-00760-7

11. Bulik-Sullivan BK, Loh PR, Finucane HK, et al. LD Score regression distinguishes confounding from polygenicity in genome-wide association studies. *Nat Genet*. Mar 2015;47(3):291-5. doi:10.1038/ng.3211

12. Ning Z, Pawitan Y, Shen X. High-definition likelihood inference of genetic correlations across human complex traits. *Nat Genet*. Aug 2020;52(8):859-864. doi:10.1038/s41588-020-0653-y

©2023 Gong W et al. *JAMA Psychiatry*.

13. Chung D, Yang C, Li C, Gelernter J, Zhao H. GPA: a statistical approach to prioritizing GWAS results by integrating pleiotropy and annotation. *Plos Genet*. Nov 2014;10(11):e1004787. doi:10.1371/journal.pgen.1004787
14. Ray D, Chatterjee N. A powerful method for pleiotropic analysis under composite null hypothesis identifies novel shared loci between Type 2 Diabetes and Prostate Cancer. *Plos Genet*. Dec 2020;16(12):e1009218. doi:10.1371/journal.pgen.1009218
15. Giambartolomei C, Vukcevic D, Schadt EE, et al. Bayesian test for colocalisation between pairs of genetic association studies using summary statistics. *Plos Genet*. May 2014;10(5):e1004383. doi:10.1371/journal.pgen.1004383
16. de Leeuw CA, Mooij JM, Heskes T, Posthuma D. MAGMA: generalized gene-set analysis of GWAS data. *PLoS Comput Biol*. Apr 2015;11(4):e1004219. doi:10.1371/journal.pcbi.1004219
17. Blake JA, Baldarelli R, Kadin JA, et al. Mouse Genome Database (MGD): Knowledgebase for mouse-human comparative biology. *Nucleic Acids Res*. Jan 8 2021;49(D1):D981-D987. doi:10.1093/nar/gkaa1083
18. Pei G, Dai Y, Zhao Z, Jia P. deTS: tissue-specific enrichment analysis to decode tissue specificity. *Bioinformatics*. Oct 1 2019;35(19):3842-3845. doi:10.1093/bioinformatics/btz138
19. Gerring ZF, Mina-Vargas A, Gamazon ER, Derks EM. E-MAGMA: an eQTL-informed method to identify risk genes using genome-wide association study summary statistics. *Bioinformatics*. Feb 24 2021;doi:10.1093/bioinformatics/btab115
20. Zhou D, Jiang Y, Zhong X, Cox NJ, Liu C, Gamazon ER. A unified framework for joint-tissue transcriptome-wide association and Mendelian randomization analysis. *Nat Genet*. Nov 2020;52(11):1239-1246. doi:10.1038/s41588-020-0706-2
21. Sey NYA, Hu B, Mah W, et al. A computational tool (H-MAGMA) for improved prediction of brain-disorder risk genes by incorporating brain chromatin interaction profiles. *Nat Neurosci*. Apr 2020;23(4):583-593. doi:10.1038/s41593-020-0603-0
22. Yu G, Wang LG, Han Y, He QY. clusterProfiler: an R package for comparing biological themes among gene clusters. *OMICS*. May 2012;16(5):284-7. doi:10.1089/omi.2011.0118
23. Subramanian A, Tamayo P, Mootha VK, et al. Gene set enrichment analysis: a knowledge-based approach for interpreting genome-wide expression profiles. *Proc Natl Acad Sci U S A*. Oct 25 2005;102(43):15545-50. doi:10.1073/pnas.0506580102
24. Foley CN, Staley JR, Breen PG, et al. A fast and efficient colocalization algorithm for identifying shared genetic risk factors across multiple traits. *Nat Commun*. Feb 3 2021;12(1):764. doi:10.1038/s41467-020-20885-8
25. Ruhlemann MC, Hermes BM, Bang C, et al. Genome-wide association study in 8,956 German individuals identifies influence of ABO histo-blood groups on gut microbiome. *Nat Genet*. Feb 2021;53(2):147-155. doi:10.1038/s41588-020-00747-1
26. Bowden J, Davey Smith G, Burgess S. Mendelian randomization with invalid instruments: effect estimation and bias detection through Egger regression. *Int J Epidemiol*. Apr 2015;44(2):512-25. doi:10.1093/ije/dyv080

©2023 Gong W et al. *JAMA Psychiatry*.

27. Bowden J, Davey Smith G, Haycock PC, Burgess S. Consistent Estimation in Mendelian Randomization with Some Invalid Instruments Using a Weighted Median Estimator. *Genet Epidemiol*. May 2016;40(4):304-14. doi:10.1002/gepi.21965
28. Hartwig FP, Davey Smith G, Bowden J. Robust inference in summary data Mendelian randomization via the zero modal pleiotropy assumption. *Int J Epidemiol*. Dec 1 2017;46(6):1985-1998. doi:10.1093/ije/dyx102
29. Slob EAW, Burgess S. A comparison of robust Mendelian randomization methods using summary data. *Genet Epidemiol*. Jun 2020;44(4):313-329. doi:10.1002/gepi.22295
30. Zhao Q, Wang J, Hemani G, Bowden J, Small DS. Statistical inference in two-sample summary-data Mendelian randomization using robust adjusted profile score. *The Annals of Statistics*. 2020;48(3):1742-1769, 28.
31. Verbanck M, Chen CY, Neale B, Do R. Detection of widespread horizontal pleiotropy in causal relationships inferred from Mendelian randomization between complex traits and diseases. *Nat Genet*. May 2018;50(5):693-698. doi:10.1038/s41588-018-0099-7
32. Rivas MA, Beaudoin M, Gardet A, et al. Deep resequencing of GWAS loci identifies independent rare variants associated with inflammatory bowel disease. *Nat Genet*. Oct 9 2011;43(11):1066-73. doi:10.1038/ng.952
33. Anderson CA, Boucher G, Lees CW, et al. Meta-analysis identifies 29 additional ulcerative colitis risk loci, increasing the number of confirmed associations to 47. *Nat Genet*. Mar 2011;43(3):246-52. doi:10.1038/ng.764
34. Mohanan V, Nakata T, Desch AN, et al. C1orf106 is a colitis risk gene that regulates stability of epithelial adherens junctions. *Science*. Mar 9 2018;359(6380):1161-1166. doi:10.1126/science.aan0814
35. Manzanillo P, Mouchess M, Ota N, et al. Inflammatory Bowel Disease Susceptibility Gene C1ORF106 Regulates Intestinal Epithelial Permeability. *Immunohorizons*. May 30 2018;2(5):164-171. doi:10.4049/immunohorizons.1800027
36. Azad MB, Wade KH, Timpson NJ. FUT2 secretor genotype and susceptibility to infections and chronic conditions in the ALSPAC cohort. *Wellcome Open Res*. 2018;3:65. doi:10.12688/wellcomeopenres.14636.2
37. Uhlen M, Fagerberg L, Hallstrom BM, et al. Proteomics. Tissue-based map of the human proteome. *Science*. Jan 23 2015;347(6220):1260419. doi:10.1126/science.1260419
38. Togashi H, Sakisaka T, Takai Y. Cell adhesion molecules in the central nervous system. *Cell Adh Migr*. Jan-Mar 2009;3(1):29-35. doi:10.4161/cam.3.1.6773
39. Aonurm-Helm A, Jurgenson M, Zharkovsky T, et al. Depression-like behaviour in neural cell adhesion molecule (NCAM)-deficient mice and its reversal by an NCAM-derived peptide, FGL. *Eur J Neurosci*. Oct 2008;28(8):1618-28. doi:10.1111/j.1460-9568.2008.06471.x
40. Weeber EJ, Beffert U, Jones C, et al. Reelin and ApoE receptors cooperate to enhance hippocampal synaptic plasticity and learning. *J Biol Chem*. Oct 18 2002;277(42):39944-52. doi:10.1074/jbc.M205147200

©2023 Gong W et al. *JAMA Psychiatry*.

©2023 Gong W et al. *JAMA Psychiatry*.
