# Supplementary material for: Role of the Gut-Brain Axis in the Shared Genetic Etiology Between Gastrointestinal Tract Diseases and Psychiatric Disorders: A Genome-Wide Pleiotropic Analysis
Source: JAMA Psychiatry. 2023 Feb 8;80(4):360–70. doi: 10.1001/jamapsychiatry.2022.4974 (PMC9909581; doi:10.1001/jamapsychiatry.2022.4974)
Supplement: Supplement 2. — Data Sharing Statement [file jamapsychiatry-e224974-s002.pdf]

Data Sharing Statement

Gong. Role of the Gut-Brain Axis in the Shared Genetic Etiology Between Gastrointestinal Tract Diseases and Psychiatric Disorders. *JAMA Psychiatry*. Published February 08, 2023. doi:10.1001/jamapsychiatry.2022.4974

Data
Data available: No
